# A mixed-methods analysis of the implementation of a new community long-COVID service during the 2020 pandemic: Learning from practice

**DOI:** 10.1371/journal.pone.0313367

**Published:** 2026-06-26

**Authors:** Stefanie L. Williams, Emily Beadle, Paul Williams, Harsha Master, Annalisa Casarin

**Affiliations:** 1 School of Health, Medicine and Life Sciences, University of Hertfordshire, Hatfield, Hertfordshire, United Kingdom; 2 Hertfordshire Community NHS Trust, Welwyn Garden City, United Kingdom; Public Library of Science, UNITED KINGDOM OF GREAT BRITAIN AND NORTHERN IRELAND

## Abstract

**Introduction:**

The rapidly increasing prevalence of long-COVID (LC), a condition characterised by multisystem complexity and high patient symptom burden, posed an immediate need to develop new clinics for assessment and management. This article reports on the rapid implementation of a reactive and responsive LC care pathway. We mapped patients’ journeys through this pathway, identifying the services that were activated according to prevalent symptoms, and used the Theoretical Domains Framework (TDF) to assess the barriers and facilitators to its implementation and delivery, from the perspective of health care professionals (HCPs) and LC patients.

**Methods:**

Mixed methods study, including retrospective quantitative cross-sectional analysis of patient data and semi-structured qualitative interviews. One hundred and sixteen patients who attended the long-COVID clinic in Hertfordshire, UK, in the first 5 months of its existence and consented for their data to be analysed. Six HCPs and five patients participated in semi-structured interviews.

**Results:**

Patients were referred into the service an average of 5.75 months post initial COVID-19 infection. 82% of patients required onward referral to other HCPs, most commonly pulmonary rehabilitation, chronic fatigue specialists, and a specialist COVID-19 rehab general practitioner embedded within the service. Patients reported having rehabilitation needs, moderate depression and anxiety, and difficulties performing usual activities for daily living. The TDF domains most relevant to the implementation of the LC pathway were *beliefs about capabilities, environmental context* and *resources, knowledge, and reinforcement.*

**Discussion:**

Our study provides novel insight into the development of a reactive multidisciplinary care pathway. Key drivers for successful implementation of LC services were identified, such as leadership, multidisciplinary teamwork, transferable skills, and knowledge exchange. Barriers to rapid set up of the service included funding constraints and the rapid evolution of an emergency context.

## Introduction

### Long-COVID

Following the recovery from an acute COVID-19 infection, some patients experience a range of persistent and debilitating physical and psychological symptoms such as fatigue, breathlessness, and cognitive impairment. Such symptoms, of which over 200 have been reported, are indicative of a multiphasic, cyclical condition, which has become known by the patient-made term ‘long-COVID’ (LC) [1,2]. The World Health Organization (WHO) defines LC, otherwise known as Post-COVID-19 Syndrome and Post-Acute Sequelae of COVID-19 (PASC), as *“the continuation or development of new symptoms three months after the initial SARS-CoV-2 infection, with these symptoms lasting for at least two months with no other explanation*” [2]. LC can manifest in multiple forms, with recent phenotyping studies clustering symptoms by organs and functions [3], deeply affecting individuals’ quality of life in different ways, with variability of patterns (relapsing-remitting versus constant manifestation) and severity. The condition is estimated to affect 1.9 million people in the UK [4] and at least 65 million individuals worldwide [5]. The foundation for managing people affected by LC is holistic and supportive care coupled with detection of treatable complications [3]. Evidence from patients attending specialist LC clinics demonstrates substantial heterogeneity in symptom burden, functional limitations and recovery trajectories, with many experiencing fluctuating or relapsing and remitting patterns of illness [6,7]. These findings highlight LC as a complex, multi-system condition requiring flexible and multidisciplinary models of care, rather than single-discipline or protocol-driven approaches.

### Long-COVID services

Due to the rapidly increasing prevalence of LC internationally, the need for the development of new care pathways and multidisciplinary clinics to assess and manage symptomatic patients was recognised, and advocated for by patient groups, clinicians, and researchers as early as April 2020 [8,9]. Furthermore, the WHO emphasised the need for multidisciplinary approaches to the assessment and management of LC, which are contextually appropriate and tailored to the specific complexities of the multisystem condition [10]. In 2021, the NHS provided £10 million for the creation of a network of clinics to help assess and treat those in the community exhibiting symptoms of LC, with the target of creating more than 60 centres across England [11]. This was subsequently followed by an additional £90 million investment in specialist LC clinics in England [12]. Prior to the availability of additional resources, community services adapted their care provision in order to absorb the high demand of caring for people with LC. With very limited national guidance or relative research, there was an immediate need to develop new pathways in response to clinical need. The new clinics needed to address the evolving nature of LC and the demand for multi-disciplinary assessment and management delivered in a short period of time.

The LOCOMOTION study, a national multi-site programme, has demonstrated both the scale of efforts to optimise LC services and the considerable variation in clinic models, staffing and pathways across the NHS [13,14]. Findings from the LOCOMOTION quality improvement collaborative further show that clinics have had to iteratively adapt referral processes, assessment models and follow-up pathways in response to emerging evidence and local constraints [14]. This variability underscores the importance of examining how locally developed pathways function in practice.

Despite the expansion of specialist services, evidence suggests that many patients continue to experience persistent symptoms and functional impairment following discharge from LC pathways. Evaluation of outcomes from specialist community services indicates that recovery is often partial and slow, with a substantial proportion of individuals remaining symptomatic months after service contact [7,15]. These findings highlight the limitations of existing models and reinforce the need for continued service development, adaptation and evaluation to better meet patient needs.

### Barriers to accessing care for long-COVID

Access to specific LC support can validate patients’ experiences of the condition and legitimising their symptoms [16–18]. However, multiple barriers to access and delivery persist, including unclear referral pathways, workforce constraints and limited integration between services, limited awareness, poor coverage, inconsistency, disconnect between healthcare systems and funding uncertainties have been identified as structural barriers to the provision of specialised integrated care for LC [12,19,20]. Healthcare professionals (HCPs) have reported a lack of existing referral pathways, service capacity pressures, lack of medical staff within LC service provision, resource issues, gaps in knowledge and lack of confidence in managing the condition as barriers to the effective implementation of community rehabilitation for LC [21,22]. However, these findings were reflective of the challenges of adapting and delivering existing integrated services, to absorb LC patients, rather than a dedicated LC clinic. Furthermore, existing research examining the implementation of LC services, from the perspective of those involved in their delivery, is limited to two UK regions, and does not utilise frameworks of implementation science [20,21]. Specific barriers identified by groups disproportionately affected by COVID-19, such as ethnic minority backgrounds, socioeconomically deprived areas and other underserved groups experiencing under-referral and delayed diagnosis despite higher disease burden [23,24], included mistrust in health professionals and fear [25]. A finding which may explain poorer uptake of multidisciplinary LC clinics by these groups [25,26]. Addressing these barriers requires attention not only to service outcomes, but also to how pathways are implemented and experienced by both patients and providers.

### Implementation science

The rapid establishment of LC services required substantial behavioural and organisational change, often within existing community rehabilitation structures. Qualitative studies of LC rehabilitation models highlight that many services were adapted from pre-existing pathways, frequently without formal implementation frameworks to guide change [27]. Given the significant health service utilisation and economic burden associated with LC [28] there is a clear need to understand the barriers and facilitators influencing implementation. Applying an implementation science framework offers a systematic approach to examining how multidisciplinary pathways are introduced, adapted and sustained in real-world settings.

The implementation, and subsequent ongoing delivery, of a new multidisciplinary healthcare service requires substantial behavioural changes at different levels within the organisation responsible for its inception. The Theoretical Domains Framework (TDF) is a theory-based approach to implementation, derived from a synthesis of psychological theories describing the possible barriers and facilitators individuals may encounter when implementing a service [29]. It can be used to provide a granular understanding of the psychological capability and reflective motivational processes underpinning behaviour change within this context [29,30]**.** The TDF consists of 14 theoretical domains, indicating factors relevant to and determinants of a specific behaviour: knowledge; skills; memory, attention and decision processes; behavioural regulation; social/professional role and identity; beliefs about capabilities; optimism; beliefs about consequences; intentions; goals; reinforcement; emotion, environmental context and resources; and social influences. The TDF has been used extensively across a wide range of healthcare settings to identify barriers and facilitators to the implementation of guidelines and services [31,32]. In relation to COVID-19, the TDF has been used to understand COVID-19 disease prevention behaviours, including vaccine uptake [33–37], hand hygiene [33,37] and adherence to guidelines [38]. It has also been applied to the development of an intervention to support doctors well-being and resilience during COVID-19 [39]. However, to our knowledge this is the first study to apply the TDF to understand the barriers and facilitators to the effective implementation of a LC service aimed at being reactive to people’s need, in the context of a disease with multiple manifestations which requires a holistic approach. Identifying the features underpinning the adaptation of services will provide valuable insights for future development of similar integrated care pathways.

### Aims of the study

The present study reports on the rapid implementation of a reactive and responsive multidisciplinary pathway in a community setting, developed to help manage demands associated with the long-term complications of COVID-19. The service was jointly led by specialist COVID-19 Rehab General Practitioner (GP) and Applied Health Professional (AHP). The pathway was first set up in August 2020, prior to the National Institute for Health and Care Excellence (NICE) publication of case definition in November 2020 and clinical guidance in December 2020 [40]. Referrals into the service came from both primary and secondary care. In addition to examining the demographic and symptom characteristics of the patients accessing the pathway and mapping their journey to provide comparison to other LC pathways [26], we also aimed to explore the experiences of service users and providers. In particular, we sought to assess the barriers and facilitators to the implementation of the clinic from the perspective of both LC clinic HCPs, and LC patients receiving care via the pathway.

## Materials and methods

### Study design and setting

The study took place within a Hertfordshire LC service, which began in August 2020. Covering a population of 600,000 people in East and North Hertfordshire, the multi-disciplinary team (MDT) initiated and developed a new COVID-19 pathway in response to patient need and demand underpinned by an integrated approach to care, i.e., to provide coordinated, holistic care involving both medical assessment and rehabilitation. A COVID-19 rehabilitation register was developed to identify and map patients in need of support and to track patient numbers and outcomes across the NHS trust. An Allied Health Professional COVID-19 Coordinator was recruited to facilitate the coordination and triage patients across pathways and systems from August 2020 onwards. The pathway was an entirely virtual community-based clinic jointly led by a COVID-19 GP and an Occupational Therapist, with a large MDT of allied health professionals. These included Physiotherapists, Speech and Language Therapists, Dietitians, Pulmonary Rehab, Chronic Fatigue specialists, and a Clinical Psychologist. Complex patients were reviewed by the clinic’s GP and referred on to secondary care specialists as needed. The team also worked closely with colleagues in acute hospitals, social care, and the voluntary sector.

A mixed-methods study design was used for this evaluation. The quantitative component consisted of a retrospective cross-sectional analysis of data collected from patients admitted to the LC service between 1st August 2020 and 31st December 2020. The qualitative component consisted of semi-structured interviews with 1) a subsample of these patients (five), and 2) six HCPs working within the service during the same time period.

### Participants and data collection procedures

Patients and HCPs were eligible to participate if they were referred to or worked within the service between the dates specified above. There were no limitations on eligibility to take part according to HCP type. Where possible, purposive sampling was used for participant selection for the qualitative interviews to ensure inclusion of participants with diverse characteristics. The recruitment period for this study was between 14^th^ April 2022 and 31^st^ July 2023.

Quantitative data were collected by staff within the clinic (HM) during the day-to-day running of the service. Upon entering the clinic, a clinical assessment of LC signs and symptoms was performed, a post COVID-19 rehabilitation form filled in and patients were asked to complete questionnaires including the measures detailed below. Of 218 patients referred into the clinic, 116 (53.2%) provided consent by phone for their data to be analysed for research purposes. Their data were transferred to the data recording system run by the Trust. Cases were selected according to participants’ consent, retrieved by Trust’s personnel and anonymised, then recorded (EB) using an electronic data collection form developed specifically for this study (S1 Table). This process may have introduced selection bias [41]. Since, the study only included patients who were referred to and attended the Hertfordshire Long COVID clinic during its first five months, it excludes people with Long COVID who did not seek help, those who could not get a GP referral, or those who were treated in different regions. Participants may have been more motivated, more dissatisfied, or more engaged with the service than the average patient/staff member, over- or under-estimating the results of the analysis.

Retrospective quantitative data were accessed on 14^th^ April 2022. Quantitative data were directly accessed by EB prior to analysis to identify eligible cases, EB therefore had access to information that could identify individual participants. However, quantitative data were anonymised and encrypted by the NHS Trust Information Manager prior to analysis.

When approached for consent to use their data for research purposes, five patients agreed also to participate in a virtual semi-structured interview. Patients were approached to participate in the research via email and/or telephone and their consent was recorded. In addition, six clinicians were invited to participate in a virtual semi-structured interview to collect their perspective. All participants were provided with information sheets and provided written consent by signing consent forms prior to participation in the interview.

All qualitative interviews were conducted by the same male researcher (PW), an Academic Research Fellow (PhD) at the time of the interview. This enabled consistency with regards to interview schedules (reliability). PW knew all clinicians because of working within the clinic in a research capacity, therefore there is a risk of Social Desirability Bias, where interviewees (especially staff) may give more positive answers to avoid criticizing their colleagues or their own workplace [41].

PW did not have a relationship with any of the patients prior to study commencement. Participants were provided with the researcher’s name and contact details in the information sheets, as well as reasons for doing the research. No other details relating to the interviewer were provided. Interviews took place online and were recorded. Only the interviewer and participants were present during the interviews. Patients and HCP interviews lasted approximately 60 minutes. Field notes were taken during the interview. No repeat interviews were undertaken. After interviewing five patients and six clinicians, the research team were satisfied that data saturation had been reached, recruitment was then ceased. Interview recordings and verbatim transcript were stored securely for analysis by the authors (AC, EB, PW, SLW). Transcripts were not returned to participants for comment and/or correction. Analysis and selection of the quotes were made by AC and SLW, not the interviewer, to avoid interviewer bias. While the researchers strove to maintain objectivity, they may have inadvertently introduced observer bias by selecting quotes that primarily supported the success of the service [41]. Recall bias (it occurs when participants look back at their past experience) may have had an impact on the responses, since the study happened several months after participants accessed the service [41].

### Measures

Demographic data were collected (i.e., age, gender, ethnicity, smoking status, BMI, deprivation status using postcode); as well as data relating to pre-existing comorbidities and disability (physical, learning). Referral data included date of referral, primary reason for referral, source of referral, discharge data, intervention (rejected for referral or discharged), referrals on to individual services and date of onwards referral. COVID-19-related test/ history data included i) hospitalisation, ii) current/past treatment, iii) onset, iv) test results, v) initial symptoms, and vi) ongoing problems.

In addition, the following measures, whose validity and reliability have been established, were used: Yorkshire Rehabilitation Screening (C19-YRS) [42,43]; clinical frailty scale; Malnutrition Universal Screening Tool (by BAPEN); UCLH loneliness assessment; PHQ-9 [44]; GAD-7 [45]; Patient functional scale and self-management plan; EuroQol five-dimension health questionnaire (EQ5D) [46]. The PHQ-9 [44] and GAD-7 [45] were only completed when deemed necessary by LC clinic staff. EQ5D [46] data were not initially collected upon clinic set up. Thus, completion rates for these measures were lower than other included measures.

Qualitative data were collected through semi-structured interviews. Patient interview schedules developed by the protocol authors (AC, EB) focused on: patients overall experience of the LC clinic, perceived clarity of their assessment and treatment pathway through the clinic, interactions with staff/clinicians, clarity of communication with clinicians/staff, satisfaction with received care, potential improvements of care and pathway, preference for virtual or in-person services. HCP interview schedules included questions on their experiences of setting up a reactive LC service, of working in a multi-disciplinary team, the organisational structure of the service, issues experienced delivering care in time of a pandemic, virtual services, interactions with patients, interactions with other staff/clinicians, adaptations to service based on patient needs/wants. Topic guides for patient and HCP interviews are available as supplementary material (S2 File).

### Data analysis

#### Quantitative data analysis.

Data were analysed in SPSS (version 28) by EB. Descriptive statistics were used to identify frequencies and percentages. Patients’ demographic variables are reported using frequencies and/or percentages for categorical variables and means ± SD for continuous variables. Descriptive data on service utilisation and referral pathways are reported using frequencies and/or percentages, including i) where patients were referred from, ii) primary reason for being referred into to the service, iii) which services were used by which patients, iv) number of services accessed by each patient, v) referrals on to other services. Pearson’s chi-square/Fisher’s exact was used to explore the relationships between demographic and service-related variables (e.g., sex and primary reason for referral).

#### Qualitative data analysis.

Anonymised interview data were coded using NVivo (version 12). Data were analysed using thematic analysis [47] to find and display recurrent themes and patterns in data. Inductive open coding, creating codes based on the qualitative data, using a line-by-line process, was used to analyse the transcripts. To ensure integrity of the analysis process, two coders (PW, AC) independently coded the first two interviews and discrepancies were discussed in order to reach agreement. Following this, the rest of the interviews were analysed by three coders (PW, AC, EB) using a single codebook, with opportunities to create new codes as needed.

Quotes were deductively allocated to one of 14-domains specified within the TDF using a spreadsheet developed for the current study. The domains were used as a guide to group codes into themes. To prevent omitting important data, subdomains were added where appropriate. Discrepancies in allocating quotes to a relevant TDF domains were resolved by discussion. A framework matrix was developed to reduce and organise the data into themes, cases, sets, and connections between categories were mapped to explore relationships between themes. Data triangulation was used to gain a greater understanding of multiple perspectives; similarities and differences between domains related to patients’ and healthcare professionals’ views were identified. Finally, codes related to barriers and/or facilitators were extracted and used to create a list (Fig 4). Participants did not provide feedback on the findings.

**Fig 1:**
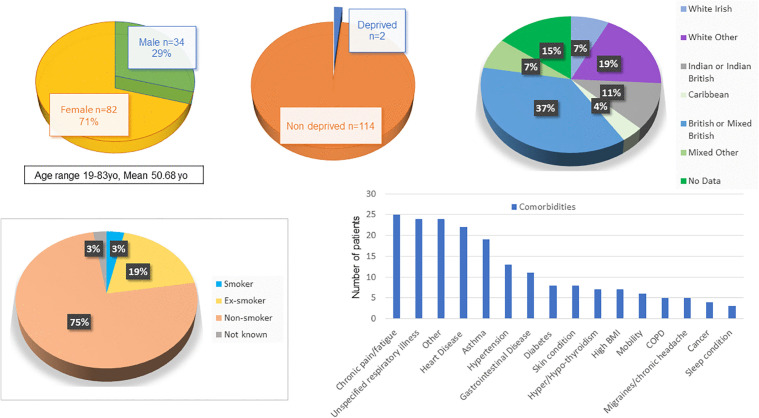
Infographics of LC clinic patients’ characteristics. Patients admitted between 1st August 2020 and 31st December 2020 to the Hertfordshire LC clinic.

**Fig 2:**
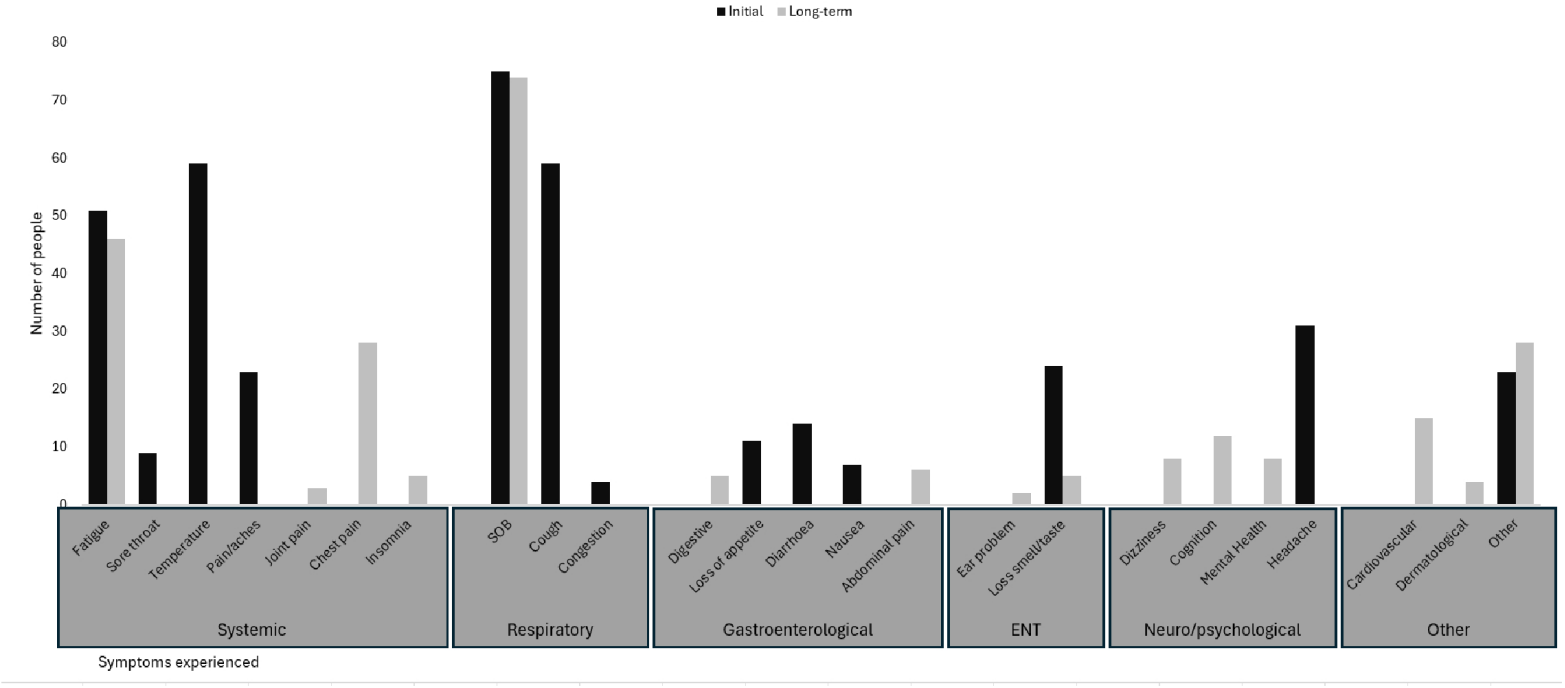
COVID and long-COVID symptoms of patients referred into the service.

**Fig 3:**
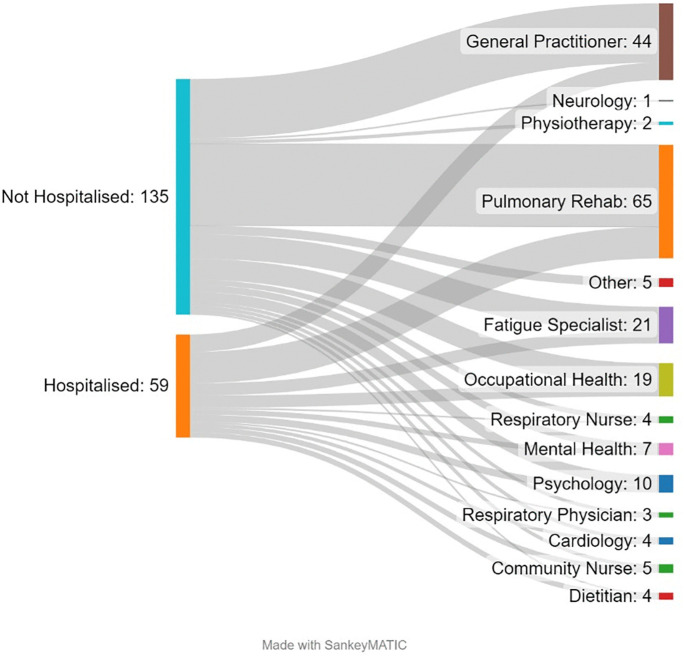
Referrals out to other services. The figure shows the number of patients referred to specialist services between August and December 2020.

**Fig 4:**
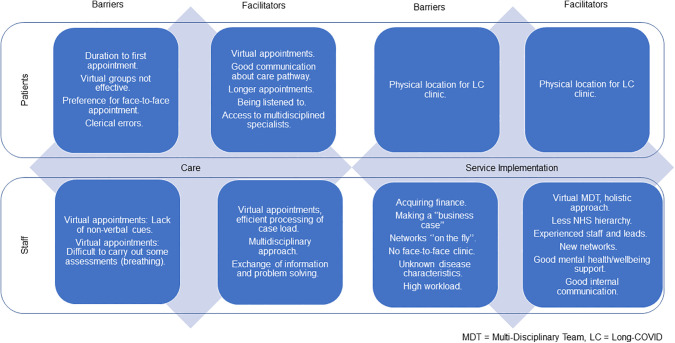
Barriers and facilitators to care and service implementation for care delivery according to patients and professionals.

### Ethics approval

Ethical approval for the study was obtained through Health Research Authority (HRA) and Health and Care Research Wales (HCRW) Research Ethics Committee (REC reference: 21/PR/0987). All participants provided informed consent prior to participating in the study. University of Hertfordshire and institutional ethics guidelines were followed.

### Patient and public involvement

The design of this project was rooted in the necessity of aligning clinical efforts with the lived experiences of those affected by LC. We prioritized three elements of the patient journey that those who attended clinic appointments identified as challenging: first assessment, management, and navigating care within a newly established system.

#### Alignment with patient priorities.

We believe the fundamental aims of this study directly reflect the research priorities identified by the LC community. To ensure this alignment:

- Direct Consultation: The decision to explore the specific barriers to accessing and navigating the care pathway was informed by direct discussions with patients attending clinic appointments.
- Patient Validation: Feedback from the public underscored the urgency of this work, with respondents specifically welcoming the study as a critical step in addressing the systemic gaps in LC knowledge and service delivery.

#### Healthcare professionals collaboration.

In addition to patient input, we actively sought the involvement of the healthcare professionals responsible for the day-to-day delivery of the clinic. Their frontline expertise was instrumental in:

- Refining the study aims to ensure they were grounded in clinical reality.
- Shaping the study plan to accurately capture the multidisciplinary nature of the service.

#### Commitment to dissemination.

To ensure the insights gained from this study contribute to the wider understanding of LC care, this study is made publicly available via open-access platforms and to study participants in a format of their choice (paper, verbal communication, poster). We believe that transparency is essential for the rapid dissemination of learning within the global medical and patient communities.

## Results

Between August and December 2020, 218 patients were referred to the clinic, of which 116 (53.2%) gave consent to take part in the current study, plus six HCPs. All patients were diagnosed with LC, had suspected LC, or had COVID-19 associated rehabilitation needs.

### Patient characteristics

Patients attending the service (n = 116) were aged between 19 and 83 years (mean = 50.68, SD = 14.40) and the majority were female (70.7%). Most were classified as living in non-deprived conditions (98.3%) and were White British (76.7%). The majority were non-smokers (75%) or ex-smokers (19%), only 3.4% were current smokers. The mean number of comorbidities patients reported ranged between 0–6 (mean = 1.95). The most common were chronic pain/fatigue (21.55%), unspecified respiratory illness (20.68%), heart disease (18.97%), asthma (16.37%) and hypertension (11.2%) Fig 1.

Patients within the LC service reported having ongoing rehabilitation needs (C19-YRS mean 7.99, SD = 6.31). Patients also reported having moderate depression (mean = 13.21, SD = 7.22) and moderate anxiety (mean = 9.39, SD = 6.76) associated with onset of COVID-19 and reported difficulties with performing usual activities of daily living (mean = 1.95, SD = 1.01).

There were no significant differences shown between month of referral into the service and C19-YRS, PHQ-9, and GAD-7 outcomes. However, there was an increase in the average score of the C19-YRS in November 2020. Patients reporting gastro-intestinal comorbidities reported significantly lower scores on EQ5D self-care measured at access to the service (mean = 1.0), versus those without (mean = 1.30), t (98) = 4.10, p < .001. No other significant differences observed regarding comorbidity type or frequency and EQ5D.

### Referrals into the service

There were between 15 and 30 patients (mean = 23.2) referred into the service per month, during the five-month study period. The number of patients referred into the service during August, September, October, November and December was 30, 20, 27, 24 and 15, respectively.

Patients were admitted to the LC clinic on average 5.75 months post initial-infection/symptoms of COVID-19. The majority of patients were admitted 7 months post-COVID (N = 28), reflecting an absence of LC clinics prior to August 2020, e.g., a person being affected by the infection in the early part of the year (Jan-April) and being admitted to the clinic between Aug and Nov. One patient was admitted in the same month as they experienced COVID-19 symptoms.

The majority of patients were referred to the service by their General Practitioner (GP: 72.4%). Initial symptoms of COVID-19 were treated at home by 63.8% of patients and the majority did not have a test (41.4%), or were too early for testing (18.1%), to confirm their COVID-19 status. In total, 33.6% had a positive test result confirmed on referral to the service. Most patients were referred to the service for respiratory conditions (50.9%) or chronic fatigue issues (18.1%). The journey of patients through the service pathway can be found in S3 Fig.

### COVID and long-COVID symptoms of patients referred into the service

Temperature changes (increased, reduced or both; n = 59, 50.86%), shortness of breath (SOB; n = 76, 65.5%), cough (n = 59, 50.86%), and fatigue (n = 51, 43.97%) were the most reported symptoms for initial acute COVID infection. There was a significant association between shortness of breath (SOB) at initial infection and smoking status, with non-smokers (never smoked) more likely to report SOB than current smokers and ex-smokers (p = 0.022) Fig 2.

Breathing difficulties, primarily continued shortness of breath (n = 74,63.79%), and fatigue (n = 46, 39.66%) were the most reported LC symptoms at admittance to the service.

Fatigue and breathing difficulties were present in both primary COVID-19 infection and LC, while cardiac (n = 16, 13.79%), concentration (n = 12, 10.34%) and mental health issues (n = 8, 6.89%) started only after the primary infection.

There was a significant difference in primary COVID-19 infection symptoms between patients who were hospitalised and those that were not hospitalised (i.e., homecare, A&E access but not admitted, self-referral). People who were treated at home or were admitted to A&E and subsequently discharged were more likely to report pain/aches (p = .011), headache (p = .043), changes in smell/taste (p = .008) and fatigue (p = .002). Patients referred into the service from primary care were significantly more likely to report pain/aches (p = .039) and changes in smell/taste (p = .040) at primary infection than patients referred to the service from secondary care.

### Referrals out of the service

There was an average of 1.59 unique referrals out of the service on to other services and health professionals, per patient (range 0–5). In total, 82% of patients had an onward specialist referral. The majority of patients required referral on to pulmonary rehabilitation (n = 65, 56.03%). The other services patients were referred to were; Specialist COVID-19 Rehab General Practitioner (n = 44, 37.93%), Community Chronic Fatigue Specialist (n = 21, 18.1%), Occupational Health (n = 19, 16.37%), Psychologist (n = 10, 8.62%), Mental Health Team (n = 7, 6.03%), Community Nurse (n = 5, 4.31%), Dietitian (n = 4, 3.45%), Respiratory Nurse(n = 3, 2.59%), Cardiology (n = 4, 3.45%), Respiratory Physician (n = 3, 2.59%), Physiotherapy (n = 2, 1.72%), and Neurology (n = 1, 0.86%).

Patients who were hospitalised were more likely be referred to further specialist advice, e.g., cardiology (dietitian, psychology), whilst patients who were not hospitalised were more likely to be referred to the specialist COVID-19 Rehab GP and pulmonary rehabilitation for further care (Fig 3).

Patients with higher EQ5D pain scores had significantly more outward referrals than those with less pain (F(5, 104) = 2.64, p = .027). Patients with anxiety and/or depression symptoms, measured using EQ5D, had significantly more unique outward referrals than patients without anxiety and/or depression (F(5, 104) = 2.58, p = .030). There was a significant association between gender and outward primary care referrals (e.g., those referred to the specialist COVID-19 Rehab GP, community nursing, community chronic fatigue syndrome specialist, and pulmonary rehab). Females were significantly more likely to be referred to the specialist COVID-19 Rehab GP than males [X2(1) = 5.88, p = .020]. There were no association between gender and outward referrals to mental health specialists (p = .78), allied health professionals services (p = .30), or secondary care (p = .40).

### Discharge from long COVID service

In total, 90 patients (77.59%) were discharged from the service. Of these, 62% (n = 56) had significantly improved symptoms or were asymptomatic, whilst 38% (n-34) were still symptomatic. One patient was discharged but requested to be re-admitted to the service. Twenty-five patients remained within the service. There was a significant association between long-term (LC) associated self-reported fatigue and discharge status (p = .002), with those without fatigue significantly more likely to have significantly improved symptoms of LC and were discharged, while those with fatigue were more likely to remain in the service.

### Qualitative data

In total, eleven qualitative interviews were conducted, with five patients and six HCPs which include two doctors, one physiotherapist, two occupational therapists, and one coordinator). TDF domains indicated as most important and relevant to the implementation of a new service to address LC patient needs, based on our analysis of patient and HCP interview data, are presented in Table 1. The number of participants who mentioned, and the number of references which related to, a specific domain is also presented. Where appropriate, sub-domains were added to further capture important features of some TDF domains.

Due to the extensive list of domains and sub-themes, the most frequently referenced domains across both patients and HCPs (*beliefs about capabilities, environmental context* *and* *resources, knowledge* and *reinforcement*) are presented in coding trees (S4 Text). A separate coding tree is presented for patients and HCPs. The other domains were also referenced by study participants, and it was found they roughly overlapped with the four dominant domains.

**Table 1:** TDF [29] domains and subdomains across patients and HCPs, Hertfordshire LC service, 2023.

TDF ([29] domains (blue) and additional sub-domains (black).	Number of patients	Number of references	TDF domains definitions.
**Behavioural regulation**	4	18	Anything aimed at managing or changing objectively observed or measured actions.
**Beliefs about capabilities**	11	79*	Acceptance of the truth, reality or validity about an ability, talent or facility that a person can put to constructive use.
Trust	1	3	
**Beliefs about consequences**	9	55	Acceptance of the truth, reality or validity about outcomes of a behaviour in a given situation.
Communication	3	18	
**Emotions**	10	78	A complex reaction pattern, involving experiential, behavioural and psychological elements, by which individual attempts to deal with a personally significant matter or event.
**Environmental context and resources**	11	253*	Any circumstance of a person’s situation or environment that discourages or encourages the development of skills and abilities, independence, social competence and adaptive behaviour.
Team working	2	19	
Expectations	3	23	
Experience of referral and service	3	22	
Adaptation	2	24	
**Goals**	6	32	Mental representations of outcomes or end states that an individual wants to achieve.
**Intentions**	7	40	A conscious decision to perform a behaviour or a resolve to act in a certain way.
**Knowledge**	9	81*	An awareness of the existence of something.
**Memory, attention and decision processes**	10	66	The ability to retain information, focus selectively on aspects of the environment and choose between two or more alternatives.
**Optimism & pessimism**	4	17	The confidence that things will happen for the best or that desired goals will be attained.
**Reinforcement**	11	88*	Increasing the probability of a response by arranging a dependent relationship, or contingency, between the response and a given stimulus.
**Skills**	8	46	An ability or proficiency acquired through practice.
Skills transfer _Learning	2	7	
**Social influences**	8	39	Those interpersonal processes that can cause individuals to change their thoughts, feelings or behaviours.
**Social or professional role and identity**	6	67	A coherent set of behaviours and displayed personal qualities of an individual in a social or work setting.
**Social-professional role and identity**	4	27	
Commitment	2	11	
Leadership	2	4	

*Most frequently identified domains. Social-professional: social behaviours and qualities of an individual in a work setting.

The most dominant TDF domains for both patients and HCPs were *beliefs about capabilities* (of staff), *environmental context and resources* (for creating a new care pathway), *knowledge* (of new conditions, its consequences, and new pathway), and *reinforcement* (when a positive experience creates reassurance and confidence on the system).

#### Beliefs about capabilities.

Patients stated that the provision of a specific LC service made them feel confident they were well looked after:

‘’ I was thrilled to be honest because I just felt quite desperate at the time.” P003

‘’…you felt confident in the process of diagnosis and then subsequent treatment.” P008

Patients acknowledged the new condition was a challenge, but the new service was important, and its existence was valuable:

*‘’ it was a relief to know that, actually, I wasn’t just gonna be forgotten.”* P005

*‘’There wasn’t another route I could take. Speak to somebody else about it*.” P009

For staff members, the new condition represented a challenge because of the uncertainty of what they were dealing with and the new pathway to be established:

‘’ *…had to create a pathway where patients could get referred in you know having… making sure that we haven’t missed anything else.*” S001

‘’ …*there was the first scoping meetings, I kind of said, because I’d come from a very systems approach in my previous jobs… I said: you’re gonna have to… have lots of systems involved with this patient cohort because we don’t know what’s needed*. … *I’d kind of have that confidence of working in pathways of care, not in an organizational silo, you know which a lot of people were still very, you know, still office.*” S002

‘’… *quite quickly you realize that you… you didn’t feel like you were as effective in your care. You know, so much of the things that we can ascertain about a patient, we obviously do visually*… *when we started to see post COVID patients face to face. I remember being quite surprised because over the phone they would have a number of symptoms that would be concerning and then they would come into clinic and you would be reassured that actually well, no, you don’t look too bad.” S004*

‘’ …*there was a bit of apprehension because it’s a change and it’s like how is this gonna work from you know various team members but actually within the space of perhaps about six weeks then that waiting list just shrunk then*.” S005

‘’*So, I think I was fairly confident that we could provide a good standard of care in terms of we could do a full assessment on them, and we could direct them to appropriate resources.*” S006

#### Environmental context and resources.

Patients reflected on the importance of providing the necessary time for staff to assess their conditions and options:

*‘’ It wasn’t like just a quick 10-minute conversation. Not like I had a bit longer, you know, to properly go into things.” P003*

*‘’ there’s this thing called long COVID. That’s some people are still suffering with effects afterwards and I don’t think those people would if they haven’t been in hospital. I think they’ve missed. They’re missed out.” P009*

Capacity of the service was referred to as a barrier to access, and as dependent on resources during a pandemic, when many people needed it:

*‘’I feel like I was quite lucky. Actually, when I got referred when I sort of been in touch with my GP, I seemed to manage to get referred over quite relatively quickly. I know a lot of people subsequently had quite long waits just because of the sheer numbers.’’ P003*

Experience and the attitude of the professionals was also mentioned as an important feature:

*‘’ And the lady was just... I seem to remember, being easy to talk to, but she was also... a no-nonsense lady and I liked that, she was very sort of direct and to the point and she knew what she was doing and you felt that this was somebody... in amongst all of this craziness and madness, here was somebody that actually knows... what’s going on because she’d obviously, I’m guessing, drawn on her previous backgrounds.’’ P008*

The pandemic restrictions didn’t affect the quality of the service. Moreover, according to the HCP interviewed, team working excelled. Appreciation was expressed towards leadership; the leaders were firm but approachable.

*‘’We haven’t had anyone that’s come back and moaned that we haven’t seen them face to face and… and you know, again, when I was a bit confused or if I was stuck or if I was, I would do a video call. And then sometimes I would get like, say, calling rehab… I’d be like, can you see them face to face and let me know what you think.” S001*

*‘’A lot of work from a therapeutic point of view is making sure that people access the right management.” S003*

*‘’ half a day, a week that they had funding for an OT, so very limited when you consider sort of what the need was and what the scope was… So, I was drafted into sort of say, can you take this on because literally there was nobody else.” S005*

*‘’ …had to think about everything in terms of how well we’re gonna gather the data? What was the new system? How the template gonna look like for this service and what? What things were we gonna need to do? And that had to change quite rapidly as we learned more about it because none of us knew really.” S006*

The need for integration of care was quite evident from the beginning because different patients had different care needs, so different services needed to be activated or engaged with.

*‘’when I did get into post, I was put in contact with respiratory consultant at the [redacted]… The COVID recovery lead. So, we what we did is near the beginning, you know when we started to see the patient, we started to work together a bit more and say well, let’s think about a pathway. … And I started doing that with cardiology. I started to do that with neurology. So, I started to create new pathways.” S001*

*‘’we tried to connect them to other services which were already running within the trust, but also try to connect them to other clinicians within the system.” S006*

#### Knowledge.

The new condition was still unknown to patients and professionals, each patient was different, and the array of symptoms was puzzling. The service provided an opportunity to develop and disseminate knowledge, which had a reassuring impact on some patients:

‘’ *It was scary territory at the time because we did we we just didn’t know enough about it* …*we needed to find out more about it. The only way we could do that was by …developing these long COVID clinics that actually you can then monitor that person, post COVID*.” P005

‘’ *And I think because I have knowledge, I wasn’t over worried about that*.” P009

‘’*You know, no one told you this was what’s gonna happen. So, you it’s kind of letting you know in advance. What could happen. I … I … I was quite happy to have that. I do feel that it’s needed*.” P011

The new condition was affecting patients in several ways, but the symptoms were all recognisable and issues staff members were used to treating. The uncertainty in the early days was soon replaced with the knowledge transfer from previous roles.

*‘’I first was asked to join, it was very much like, you know, we don’t, we don’t really know what is out there…” S001*

*‘’You, kind of like most clinical jobs that you do after you’ve been embedded into the ward or the service after a few months, it all comes …you have a confidence about what to do. But this job you don’t. Yeah, you just… It’s just 1/2 year later I still feel like a schoolgirl in this job.” S002*

Knowledge by the team, regarding the condition, the care needed and as shown to the patients, was a source of reassurance in a period when media messages created confusion.

*‘’ There’s something about being able to reassure people in the context of the problems that they’re having, because we all built up our knowledge about some of the symptoms that people were having.” S003*

*‘’I think we were at the start, we were slightly… You can’t know what you don’t know. … you were learning more about COVID, long COVID that then you’d think. … there was quite a lot of stuff that was coming out in the media about long COVID as well.” S006*

#### Reinforcement.

Knowing about the service and feeling they were looked after, made an impact of patients’ wellbeing:

‘’ *I think we just had a chat on the phone instead of went through all of my symptoms and umm, just kind of talked, talked it all through everything that was going on as far as I remember, which I found very reassuring... Umm, so I guess it made me feel, you know, hopeful*.”P003

”*…hundred, 100% satisfied like I say. Just because it was such a new virus that we knew nothing about, it was just nice to know. That basically I’d had probably the best MOT in my life*.” P005

Positive feedback and patient outcomes reinforced the belief that staff members were heading in the right direction. Also, it was another source of reassurance for the patients that there was a way out of the uncertainty of their conditions. Reinforcing positive outcomes was also important to provide evidence to commissioners for more resources to be invested.

*‘’You know that initial assessment and that review that I do is so thorough that they’re so they’re really delighted that someone has actually finally taken that whole story and is looking at that bigger picture.” S001*

*‘’You still have to, have this, you know, kind of confidence of working in unknown uncertainty, but also the ability to kind of you know have confidence in like what we do know so far and what we have done so far has had a good effect.” S002*

*‘’We had to move to a more generalist type approach with then specialist input… we kind of developed the offer based on what the patients were telling us.” S003*

#### Summary of principal findings.

The number of patients that accessed the LC clinic during the first five months of implementation ranged between 15 and 30 per month, and patients were on average 5.75 months post initial COVID-19 infection when they were admitted. Patients attending the service were predominantly female, White British and were from non-deprived areas. The most commonly reported LC symptoms reported by patients were shortness of breath and fatigue. On average there were 1.59 referrals out of the service to other specialists, with the majority of referrals going to pulmonary rehabilitation, primary care and community chronic fatigue specialists.

The service went through a series of adaptations (i.e., implementation of data collection tools, inclusion of additional specialities) to deliver care based on the evolving understanding of the condition as well as patients’ needs. There was a substantial time lag between initial evidence of LC diagnosis and the ability to access care via the LC pathway, with some patients required to wait up to seven months for care. The delay in both the identification of LC as a distinct condition and acknowledgment of the need to invest resources for delivery of LC services at that time likely contributed to this. Nonetheless, the LC clinic delivered a high performing service that met patient needs during challenging times. When people entered the pathway, the journey was unpredictable and often included a reiteration of assessments and further referrals (S3 Fig). It demonstrates the cross-disciplinary collaborations that staff members established, or reinforced if already present, to provide high quality multidisciplinary care for complex LC care. Based on patients’ recollection and HCPs learning from the experience of accessing and delivering a new care pathway, barriers and facilitators of implementing a new service were identified, which are presented in Fig 4.

## Discussion

The emergence of a new virus with multiple post infective complications was one of the biggest health challenges in 2020. On a background of considerable uncertainty around pathology, optimum treatment and with very limited research and national guidance or research and in the face of increasing numbers of patients requiring support for LC symptoms, there was an immediate need to develop new pathways in response to clinical need. Learning from that experience, current NICE Guidance recommends that people with LC should be offered integrated and coordinated care, referral for multidisciplinary assessment or, for specific complications, referral to specialist care [40]. Several studies have reported on the implementation of support for LC within the UK health service context [21,26,48,49]. A prospective study of 1325 patients attending a dedicated LC assessment and management service set up in April 2020 [26], demonstrated the significant prolonged functional impairment of patients and the extensive need for onward referral to other specialists. The development of an integrated rehabilitation pathway for community LC patients has been investigated by a group of clinicians in Leeds, UK [49]. The authors report the referral criteria used to manage LC patients through the system and the professionals involved. This study instead shows the potential barriers and facilitators of implementing such services in a local setting.

Healthcare professionals within the East and North Hertfordshire NHS Trust had no preparation time or any opportunity to draw upon principles of established implementation or improvement science frameworks for creation of a pathway. Instead, staff acted rapidly according to patients’ needs and feedback and the resources available, as well as their previous experience, to meet patients’ needs. The establishment of a MDT within the LC pathway, including a Specialist COVID-19 Rehab General Practitioner, Occupational Therapists, Physiotherapists, Speech and Language Therapists, Dietitians, Pulmonary Rehab, Chronic Fatigue specialists, and a Clinical Psychologist, enabled knowledge exchange across specialities. This contributed to increased knowledge of the condition, effective problem solving and the provision of a holistic approach to LC care. Furthermore, due to their existing skills and capabilities HCPs were able to rapidly develop and establish further pathways into secondary care for patients that required more specialised investigations and review, which included onwards referral to respiratory, cardiology, ENT and neurology. Professionals also felt positive about collaborative working, despite the challenging context. The pandemic gave HCPs the opportunity to work interactively with other services and deliver practical, integrated care pathways. HCPs expressed positive feelings around working in a multi-disciplinary team, collaborative working was perceived as satisfying and leadership was strong, staff members acknowledged the efforts and experience each person contributed to the creation of something new during exceptional times.

Prior studies of UK LC clinics have identified several organisational and individual-level factors influencing their implementation and uptake, including the novelty of the condition, resource and capacity-related issues, knowledge limitations and lack of existing referral pathways [18,21]. However, to date there has been limited use of implementation science frameworks to explore this within this context. The TDF was used to formulate interview questions for LC Leads in one recent study [21]. However, the framework wasn’t used to guide analysis and was not applied to the interviews with patients. In contrast to previous research, the current study employed a mixed-methods research design which has facilitated a greater depth and breadth of understanding of the context within the LC service was implemented and led to the following findings.

### Barriers and facilitators to implementation and delivery

Several barriers and facilitators to the implementation and delivery of the LC pathway, from the perspective of both patients and HCPs, were identified using the TDF (Fig 4). Patients and professionals, recalling the use and creation of the service, implied that the main areas to focus on, when implementing a new service for a novel condition, are the **environmental context and resources**, to facilitate the initial implementation and ongoing delivery of a new care pathway; the **capabilities of staff members**, specifically knowledge and expertise of HCPs delivering the service; **knowledge** and prompt dissemination of information about the new condition, its consequences, and the new service; and positive **reinforcement**, when positive experiences and good patients’ outcomes creates reassurance and confidence in the way the system is adapted [29].

#### Environmental context and resources*.*

Overall, the experience of setting up a reactive and responsive service in an emergency was exceptional for all the people involved. Nevertheless, the already established organisational structure of the service supported the new pathway implementation well. Barriers to the initial set-up of the LC pathway included budgetary constraints and the need to make a LC ‘business case’ to commissioners, which may suggest the need for new funding strategies or the creation of emergency funds. Lack of central face-to-face clinic location, lack of administrative and clinical processes and high workload were indicated as challenges by HCPs. Virtual delivery presented some challenges for HCPs, including difficulties in completing necessary breathing assessments and a reduction in non-verbal cues. Nevertheless, virtual delivery did facilitate efficient processing of heavy caseloads, was well accepted by patients and perceived as a safe mode of delivery within this context. Lack of knowledge around the disease characteristics of LC created uncertainty regarding which specialities were required within the pathway and multidisciplinary networks were therefore created rapidly with limited prior planning. Difficulties setting up the service and subsequent access for patients may have contributed to the increase in average LC symptom severity in November 2020, as shown by the C19-YRS [42,43], although it is also possible that this reflected the timing of the second wave and lockdown, which created a barrier to access in-person facilities.

Although the socio-demographic characteristics of those attending the LC service reflect that of the local population [50], patients from ethnic minority groups and areas of high deprivation were underrepresented, which reflects the findings of a previous study [26]. Patients from these groups were disproportionately affected by acute COVID-19 infection [51,52] and arguably should therefore have been seen more frequently within LC services. This finding, however, may reflect lack of trust in health services, and experiences of inequalities of access of minority ethnic groups living with LC, a key barrier to uptake of services amongst this population [25]. Barriers to inclusive care were explored in a study delivered by the LOCOMOTION consortium, a group of investigators, funded by the National Institute of Health and Social Care Research in UK, exploring optimal treatment for people affected by LC [14]. The study found that having to battle for recognition in the face of structural inequalities can be a taxing experience, which may contribute to the low number of members of ethnically marginalised populations that visited the LC clinic. Additionally, this process is followed with misdiagnosing symptoms and feelings of not being recognised as affected by a chronic condition. It’s also evident that the various levels of tenacity needed are related to the social and cultural health capital of individuals with LC. Some were able to obtain their own information on LC through other sources or rely on family members who were employed by the NHS. However, some people lacked the social and cultural support necessary for taking care of their health, and coupled with their disadvantage circumstances, they hardly sought support [53].

#### Capabilities and knowledge of healthcare professionals*.*

The present study has identified the value of existing transferable capabilities and expertise of HCPs, beyond their experience of specifically treating LC. In particular, the leadership experience from previous roles contributed to successful service implementation. Effective teamwork within the pathway and across different specialities was reported as a facilitator to both clinic set-up and delivery. HCPs felt positive about collaborative working, despite the challenging context. The pandemic gave HCPs the opportunity to work interactively with other services and deliver practical, integrated care pathways. HCPs also expressed positive feelings around working in a multi-disciplinary team, collaborative working was perceived as satisfying and leadership was strong, staff members acknowledged the efforts and experience each person contributed to the creation of something new during exceptional times.

The existence of a clinic tailored specifically to LC, and access to HCPs with specific knowledge of and expertise in treating the condition, provided reassurance for patients who were anxious and reported that they were “desperate”. HCPs understood patients needed dedicated time and acknowledgement of their condition, beyond that provided by usual care, and this served to validate patients’ experiences of LC. This was a finding which has been reported previously by patients receiving care in another multidisciplinary LC service [21]. In addition, patients reported excellent interactions with clinic staff and communication was reported as being good. Patients felt looked after and in capable hands, confirming the importance of the implementation of the new service for the patients’ physical and mental wellbeing.

### Strengths and weaknesses of the study

This study is the first, to our knowledge, to utilise a mixed methods analysis in conjunction with the TDF to understand which barriers and facilitators exist in relation to the implementation of a LC service. This led to evidence-based suggestions for the implementation of both future LC clinics and reactive services during critical public health emergencies. However, in the present study the TDF was used retrospectively to guide analysis and was not used in the development of the qualitative topic guides.

The study extends our knowledge of the lived experience of patients receiving care from multidisciplinary LC clinics as well as those involved in its delivery. The current paper reports on the implementation of a single LC pathway, therefore it is likely that national and regional variations exist in the implementation and delivery of LC services within the UK health service context. As such, whilst four dominant TDF domains were identified as relevant to service implementation in the current study, there are likely to be other barriers and facilitators to the implementation of other LC pathways that haven’t been identified here. Findings from our study complement the results of the LOCOMOTION consortium by integrating the lived experiences of patients and the frontline insights of healthcare professionals into the study’s structural framework [14].

In total, 102 people didn’t consent to share their data for analysis, which may have created bias in this respect. A gender split where females outnumber males (70.69% vs. 29.31%) introduces significant gender bias. While this ratio actually mirrors the global epidemiological trend for LC, which consistently shows a higher prevalence in women, it still impacts how results primarily describe the female experience of LC. This should be considered when service adaptations based on our findings will be applied to practice. Moreover, of those eligible, very few patients agreed to participate in the interviews, which may be due to patient reluctance to recall difficult experiences related to LC or perceived additional burden at a time when they had competing demands in relation to managing their condition. Furthermore, interviews with patients and staff were conducted several months after the implementation of the service, contributing to the possibility of recall bias. Quantitative data collection took time to be optimised when the LC clinic opened, the C19-YRS patient-reported outcome measure [41,42] was not yet available, and measures of anxiety and depression were initially only completed if there was perceived need to do so by clinic staff. A such, data for some measures were incomplete. However, adaptations to data collection processes later on during service delivery, further demonstrates the responsiveness of the service.

We were unable to collect data on the impact of the service on patients’ recovery, due to pragmatic reasons. A follow-up analysis is underway as part of another project. Despite these limitations, the triangulation of both the quantitative and qualitative data, alongside the inclusion of multiple perspectives, allowed for a deep understanding of the implementation of the LC pathway. This further increases the validity and reliability of the findings [54].

### Recommendations for the implementation and delivery of new LC clinics

This study findings provide evidence-based recommendations to inform the set-up, implementation, and delivery of multidisciplinary LC clinics. Effective integrated care requires early mapping of essential services based on patient need, assessment of existing clinical knowledge, and development of context-sensitive multidisciplinary referral pathways. Use of implementation and improvement science frameworks, supported by collaboration with academic experts, can help identify barriers and facilitators to successful service delivery and support patient-centred care.

Involving multiple stakeholders—including managers, clinicians, and patients—in planning can ensure services meet diverse needs. Meaningful inclusion of experts by experience in planning and commissioning processes can improve representation and mitigate access inequalities.

Pathways should remain flexible, with contingency plans to accommodate changes in the condition and patient needs, alongside early identification of opportunities to streamline referrals. Embedding collaborative working frameworks, exploring virtual and digital care options, and prioritising staff wellbeing through dedicated support and development opportunities can strengthen team functioning, retention, and service sustainability.

## Conclusion

This study contributes important insights into the development and delivery of multidisciplinary LC services, identifying key drivers of successful implementation, including strong clinical leadership, effective multidisciplinary teamwork, transferable professional skills, and active knowledge exchange. At the same time, the findings highlight persistent barriers to rapid service set-up, particularly funding constraints and the challenges posed by a rapidly evolving emergency context. Further qualitative and longitudinal research is needed to capture patients’ lived experiences and the longer-term impact of multidisciplinary care on health and wellbeing. Priority should be given to research with underrepresented yet disproportionately affected groups to identify barriers and facilitators to accessing care, enabling services to be better tailored and uptake improved. Greater attention is also required to the follow-up needs of patients discharged while still symptomatic, as well as to the influence of social determinants of health on outcomes, carer wellbeing, and resource use.

## Supporting information

S1 Table COVID rehab screening form. (PDF)

S2 File Patient and HCP Interview topic guides. (PDF)

S3 Fig Long-COVID pathway infographic flow diagram. (TIF)

S4 Text Coding trees for patient and HCP interviews. (PDF)

S5 Table COREQ checklist. (PDF)

S6 File Quantitative data collected during the study. (PDF)

S7 File Patient interview transcripts. (PDF)
