# Supplementary material for: A mixed-methods analysis of the implementation of a new community long-COVID service during the 2020 pandemic: Learning from practice
Source: PLoS One. 2026 Jun 26;21(6):e0313367. doi: 10.1371/journal.pone.0313367 (PMC13308792; doi:10.1371/journal.pone.0313367)
Supplement: S1 Table — (PDF) [file pone.0313367.s001.pdf]

Post Covid-19 Rehabilitation Patient Questionnaire

Dimension	Question	Delete & Comment Where Appropriate
Basic Information	Name: M/F	
	Address:	
	Date of Birth:	
	NHS number if known	
	Tel numbers: Home:<br>Mobile:	
	Ethnicity	**White**<br>English / Welsh / Scottish / Northern Irish / British<br>Irish<br>Gypsy or Irish Traveller<br>Any other White background<br><br>**Mixed / Multiple ethnic groups**<br>White and Black Caribbean<br>White and Black African<br>White and Asian<br>Any other Mixed / Multiple<br>Asian / Asian British<br>Indian<br>Pakistani<br>Bangladeshi<br>Chinese<br>Any other Asian background<br><br>**Black / African / Caribbean /**<br>Black British<br>African<br>Caribbean<br>Any other Black / African / Caribbean background<br><br>**Other ethnic group**<br>Arab<br>Any other ethnic group
Consent	Consent to –<br>• This data being stored on your record<br>• This data being shared with other teams, GP etc<br>• This team viewing your medical record	Yes / No<br><br>Yes / No<br>Yes / No

		Sign ………………………
Onset and history of Covid 19	Date of onset of Covid symptoms:<br><br>Date and result of swab:<br><br>Were you admitted to hospital because of Covid19?<br>Date of admission:<br>Date of discharge:<br>Did you go to ITU (intensive care unit)?<br>Number of days on ITU:<br>Did you require ventilation on a machine?<br>Number of days on ventilation machine:<br><br>Have you had a Chest X-ray?<br>Echocardiogram?<br>CT chest scan / angiogram	 <br>Positive / Negative<br><br>Yes /No<br><br><br><br>Yes / No<br><br>Yes / No<br><br><br>Yes / No / Date :<br>Yes / No / Date:<br>Yes / No / Date:
General health	In general, prior to this illness how would you rate your general health on a scale of 0 to 10 (with 10 being the best health you can imagine and 0 being the worst health you can imagine)<br><br>How would you rate your health today on the same scale?	……..<br><br>……...
Understanding?	How would you rate your understanding of what`s going on?<br>Do you have any concerns?	Good / Ok / Limited
Smoking / Alcohol	Do you smoke tobacco or other drugs or vape/use electronic cigarettes?<br><br>Alcohol consumption: number of units per week	Yes / No<br>Details:<br><br>…………
Breathing	Did you have any form of breathing problem prior to developing COVID-19 such as COPD, asthma, bronchiectasis, lung fibrosis or other/not sure?<br><br>Were you prescribed oxygen for another condition prior to developing COVID-19<br><br>Has your breathing changed for the worse since	Yes / No / Unsure<br><br><br>Yes / No<br><br><br>Yes / No

	developing COVID-19?<br><br>If yes, do you have new or worsened:<br>Shortness of breath:<br><br>At rest?<br>On exertion?<br>Cough?<br>Phlegm/mucus on your chest?<br>Wheeze?<br>Other breathing problem that you are worried about?<br><br>Have you been given oxygen to use at home since developing COVID-19?<br><br>If you have been prescribed inhalers, are you having any difficulties managing them?	 <br><br><br><br>Yes / No<br>Yes / No<br>Yes / No<br>Yes / No<br>Yes / No<br>Yes / No<br><br><br>Yes / No<br><br><br>Yes / No / N/A
Fatigue	Prior to this illness, did you have any issues with fatigue?<br><br>How would you rate your fatigue prior to this illness (with 1 being no fatigue at all and 10 being the most fatigue you can imagine)<br><br>What did you find helpful in managing this?<br><br>Thinking about the past week, how would you rate your level of fatigue on the same scale of 1 – 10<br><br>Have you noticed any patterns to this fatigue?<br><br>Or anything that helps you to manage your fatigue more effectively?<br><br>Is your fatigue impacting on your mobility, personal care activities and or ability to do the things that bring you enjoyment?	Yes / No<br><br><br>…….<br><br><br><br><br>……...<br><br>Yes /No<br><br>Yes / No<br><br><br>Yes /No
Mobility / Physical	How was your mobility prior to your illness?<br><br><br>Did you require any mobility aids or support?<br><br><br>Do you have any mobility issues now?	Details :<br><br><br>Yes / No<br>Details:<br><br>Yes/ No<br>Details:

	Since your illness do you have any new issues with your joints, muscles, sensation or strength?<br><br>Have you had any falls in the last 2 weeks?<br><br>Have you been feeling dizzy or light headed, especially when you first stand up?	Yes / No<br>Details:<br><br>Yes/ No<br>Details:<br><br>Yes/ No<br>Details:
Self-Care	Were you able to care for yourself independently prior to your illness (washing, dressing, toileting, meal preparation)?<br><br>Please describe any difficulties you were having and equipment or support already in place<br><br>Are you able to care for yourself now (washing and dressing, toileting, meal preparation)? Please circle:	Yes / No<br><br>• No problem<br>• Slight difficulties<br>• Moderate difficulties<br>• Severe difficulties<br>• Unable to wash or dress
Usual activities	Thinking about the activities you carried out routinely before your illness (work, study, housework, leisure)<br><br>Were you able to take part in these activities before you were unwell? What support did you require?<br><br>How possible is it for you to take part in these activities now? Please circle:	• No problem<br>• Slight difficulties<br>• Moderate difficulties<br>• Severe difficulties<br>• Unable to take part<br>• Not applicable (if still in hospital)
Stress	Since this episode of illness, have you noticed flashbacks, nightmares, anger or jumpiness?<br><br>Is this getting in the way of you managing your day to day life?<br><br>In the last 2 weeks have you had any periods of re-living your experience of being unwell?	Yes / No<br><br>Yes /No / N/A<br><br>Yes / No

	In the last 2 weeks have you had any hallucinations?<br><br>How is your sleep compared to how it used to be?	Yes / No
Anxiety	Did you have any issues with anxiety prior to this illness? If so, what strategies and support did you have in place?<br><br>Thinking about how you've been feeling in the last 7 days, rate your response to each question :<br><br>**I feel nervous, anxious or on edge:**<br><br><br><br><br>**Worrying thoughts go through my mind:**<br><br><br><br><br>**I have trouble relaxing:**	Yes / No<br><br><br><br><br>3 Nearly every day<br>2 More than half the days<br>1 Several days<br>0 Not at all<br><br>3 Nearly every day<br>2 More than half the days<br>1 Several days<br>0 Not at all<br><br>3 Nearly every day<br>2 More than half the days<br>1 Several days<br>0 Not at all
Mood and Depression	Did you have any issues with depression prior to this illness? If so, what strategies and support did you have in place?<br><br>Thinking about how you have been feeling in the last 7 days, rate your response to each question:<br><br>**I have been feeling down, depressed or hopeless**<br><br><br><br>**I have poor appetite / overeating:**<br><br><br><br><br>**I have been feeling bad about myself :**	Yes / No<br><br><br><br><br>3 Nearly every day<br>2 More than half the days<br>1 Several days<br>0 Not at all<br><br>3 Nearly every day<br>2 More than half the days<br>1 Several days<br>0 Not at all<br><br>3 Nearly every day<br>2 More than half the days<br>1 Several days<br>0 Not at all

	**I feel like I have let my family down:**	3 Nearly every day<br>2 More than half the days<br>1 Several days<br>0 Not at all
Pain / Discomfort	Did you have pain or discomfort prior to this illness?<br><br>How did you manage this?<br><br>Thinking about your pain / discomfort now, Please rate on a scale of 1 to 10 (with 1 being no pain and 10 being the worst pain you can imagine)	Yes / No<br><br><br><br>……….
Thinking / Memory	Do you feel that your memory is worse than it was before you were unwell?<br><br>Do you feel that your concentration is worse than it was before you were unwell<br><br>Specific issues re memory or concentration :	Yes / No<br><br>Yes / No
Communication	**Have you or your family noticed any change in the way you talk and communicate with people?**<br><br>Do people say it is difficult to understand you (new problem)?<br><br>Is it taking longer to communicate your thoughts and feelings when you are speaking or writing (new problem)?<br><br>Do you have problems understanding others or with reading (new problem)?<br><br>Is starting or keeping a conversation going or concentrating difficult (new problem)?	Yes / No (if no move to next section on swallowing)<br><br>Yes / No<br><br>Yes / No<br><br>Yes / No<br><br>Yes / No
Swallowing	**Since this episode of illness, have you noticed if chewing / swallowing is more difficult?**<br><br>• Is coughing when drinking a new problem?<br>• Is coughing when eating a new problem?<br>• Is struggling to swallow medication a new	Yes /No (if no move to next section on voice)<br><br>Yes / No<br>Yes / No

Voice	problem?<br>• Are you now taking longer to eat or out of breath chewing (new problem)?<br><br>**Since this episode of illness, have you noticed any of the following?**<br>• Voice is hoarse or weak but slowly improving<br>• Voice is hoarse or weak with no improvement (new problem)<br>• Any episodes of feeling like you can't get your breath?<br>• Any episodes of noisy breathing when you inhale?	Yes / No<br><br>Yes / No<br><br><br><br>Yes / No<br><br>Yes / No<br><br>Yes / No<br><br>Yes /No
Dietary	What was your weight before COVID?<br><br>What is your weight now?<br><br>Have you noticed any unintentional weight loss since your illness?<br><br>Is breathlessness or taste changes impacting the quantity of food you can eat?	 <br><br><br>Yes / No<br><br>Yes / No
Oral Health	Are you having issues with your oral health as a result of your illness? (e.g. dryness, soreness, bleeding, pain, ulcers, problems with teeth or dentures)	Yes / No<br>Details:
Skin	Do you find any areas of your skin are sore from the pressure of sitting or lying for long periods?<br><br>Have you noticed any changes in your skin since having Covid 19 ?	Yes / No<br>Details:<br><br>Yes / No<br>Details:
Medication	Are you managing your medication at home (even if this is with the support of others)?<br><br>Since becoming unwell or since discharge from hospital:<br><br>Have any changes been made to your medications?<br><br>Have there been any changes in the way you are managing your medication?	Yes / No<br><br><br><br><br>Yes / No<br>Details:<br><br>Yes / No<br>Details:

Social	Current Job<br><br>If unemployed, was job lost due to COVID-19?<br><br>Do you feel you have adequate advice to manage your daily tasks and financial matters?<br><br>Has a family member become a new carer for you?<br><br>Do any of your close family members or household need emotional support or advice as a result of COVID-19?<br><br>Do you have any learning disabilities?	Details:<br><br>Yes / No<br><br>Yes / No<br><br>Yes / No<br><br>Yes / No<br><br>Yes / No<br>Details:
Diabetes	Do you have diabetes?<br><br>Have you had any high or low blood glucose levels that have been difficult to control and are frequent in their occurrence and are those new?<br><br>Have you contacted your diabetes Care provider?<br><br>Would you like any further support for your diabetes?	Yes / No (If No, move onto next section Medical)<br><br>Yes / No<br><br>Yes / No<br><br>Yes / No
Medical	Do you have ongoing symptoms that are not yet under control and that are not being overseen by a medical professional?	Yes / No<br>Details:
Rehabilitation preferences	If you are offered rehabilitation, which of these formats are you happy to use:<br><br>Individual<br>Group<br>Telephone<br>Video calling<br>App<br>Paper work	 <br><br>Yes / No<br>Yes / No<br>Yes / No<br>Yes / No<br>Yes / No<br>Yes / No

	All the above	Yes / No
