# Supplementary material for: A mixed-methods analysis of the implementation of a new community long-COVID service during the 2020 pandemic: Learning from practice
Source: PLoS One. 2026 Jun 26;21(6):e0313367. doi: 10.1371/journal.pone.0313367 (PMC13308792; doi:10.1371/journal.pone.0313367)
Supplement: S2 File — (PDF) [file pone.0313367.s002.pdf]

Topic guide – patients and HCPs
IRAS ID: 299032

Version 1 (15.06.2021)

Patients

Before beginning the interview: *the interviewer states the participants study number and asks if the participant give consent to recording the interview*

General Introduction
Thank you for agreeing to take part in this study. Before we begin the discussion, we would like to remind you that there are no right or wrong answers to the questions, and any information you tell us today will be kept confidentially and quoted anonymously.
You can let us know at any time if:

- You do not wish to answer a question
- You would like the audio recording to be switched off
- You would like to leave the discussion

Start recording once any questions answered

1. How was the experience of being referred to the long-COVID clinic?
   How easy was it to get an appointment, how long did it take, how did it make you feel, how did you know about the clinic?

2. Tell me about your first appointment with the clinic
   What did it involve, how was it conducted, how long did you spend speaking with staff?

3. What was your experience of virtual/online services received in the clinic?
   Was this the majority of your care or did you have more in-person interactions? How did you find the online/virtual [compared to in-person appointments/interactions]? Did you prefer online/virtual or in-person appointments/interactions?

4. Was the process and pathway through assessment and treatment of long-COVID made clear?
   Was the process explained to you, were treatment options made clear to you?

5. How were the interactions you had with staff at the clinic?

6. Was communication of your assessment and treatment clear? Was communication from staff generally clear?

7. Were you/are you satisfied with the care received at the clinic? And why?

8. Is there anything you think could have been better?
   Any improvements that could be made? Any changes you would like to see made? Any service to be implemented?

9. Do you have any other comment?

Thank you very much for taking part to this project, we will make results available to participants.

Topic guide – patients and HCPs
IRAS ID: 299032

Version 1 (15.06.2021)

HCPs

Before beginning the interview: *the interviewer states the participants study number and asks if the participant give consent to recording the interview*

General Introduction
Thank you for agreeing to take part in this study. Before we begin the discussion, we would like to remind you that there are no right or wrong answers to the questions, and any information you tell us today will be kept confidentially and quoted anonymously.
You can let us know at any time if:

- You do not wish to answer a question
- You would like the audio recording to be switched off
- You would like to leave the discussion

Start recording once any questions answered

1. Tell me a bit about your role in the long-COVID clinic

2. Can you describe the organisational structure of the service when it opened and now? Was it changed from inception according to organisational or patients' needs?

3. What was the experience like of setting up the long-COVID clinic?

At what point did you join the team (if you were not there at the beginning)? What were the challenges of setting up the clinic? And facilitators?

4. What were the challenges of delivering care during the pandemic?

5. How were your interactions with patients?

6. How were your interactions with other staff?

7. What was your experience of virtual services provider?

How did they compare to in-person services? Did you experience these directly?

8. What was it like working in a multi-disciplinary team?

9. What do you think did work well? Or not so well?

10. What changes/adaptations would you like to see made to the service?

Any adaptations based on patient needs/wants, any feedback from patients on this?

11. Do you have any other comment?

Thank you very much for taking part to this project, we will make results available to participants.
