# Supplementary figures and images for: A mixed-methods analysis of the implementation of a new community long-COVID service during the 2020 pandemic: Learning from practice
Source: PLoS One. 2026 Jun 26;21(6):e0313367. doi: 10.1371/journal.pone.0313367 (PMC13308792; doi:10.1371/journal.pone.0313367)

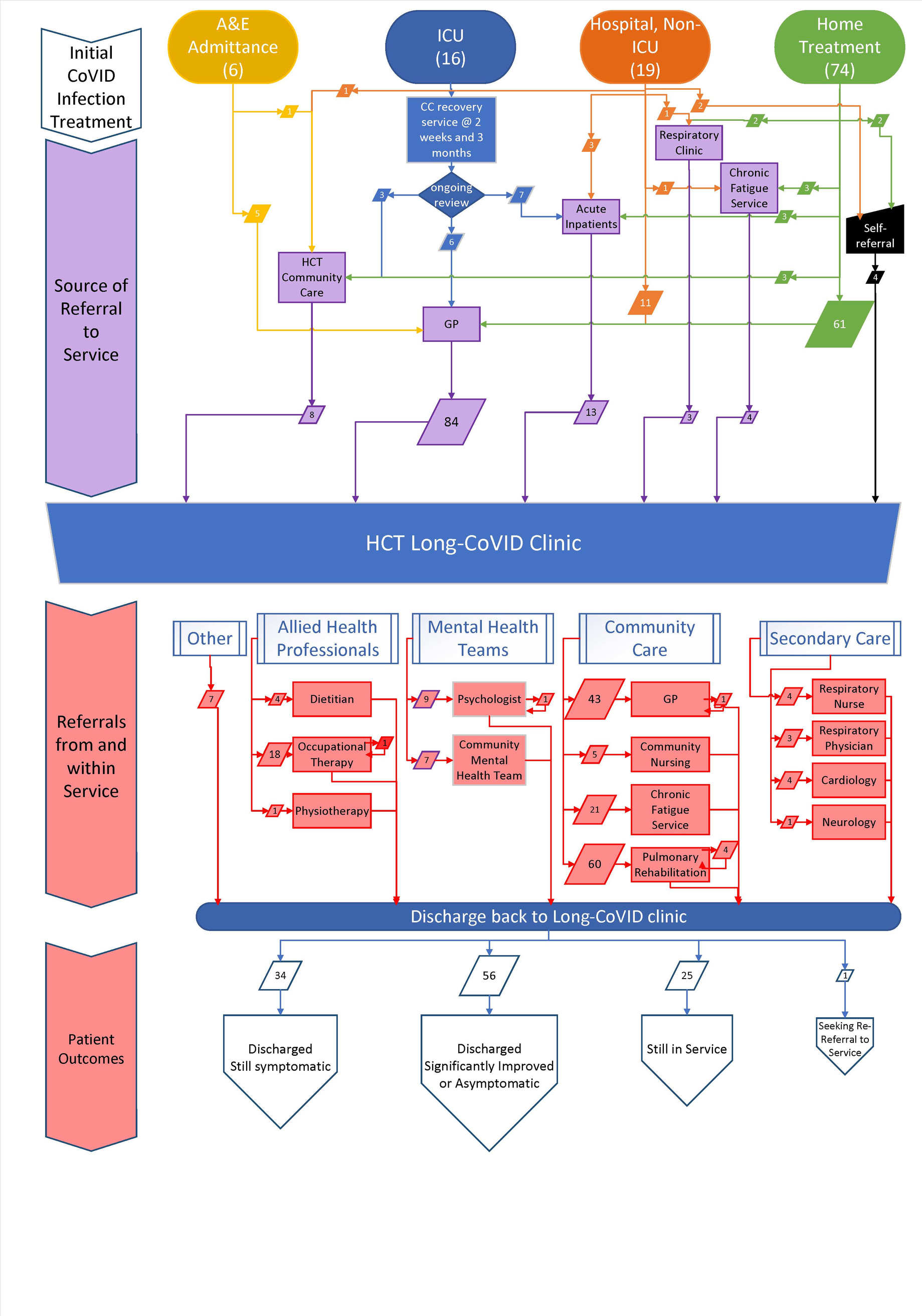

Supplement: S3 Fig — (TIF) [file pone.0313367.s003.tif]
