# Supplementary material for: A mixed-methods analysis of the implementation of a new community long-COVID service during the 2020 pandemic: Learning from practice
Source: PLoS One. 2026 Jun 26;21(6):e0313367. doi: 10.1371/journal.pone.0313367 (PMC13308792; doi:10.1371/journal.pone.0313367)

Simplified coding tree showing the four salient domains with connected subthemes emerging from patients' interviews.

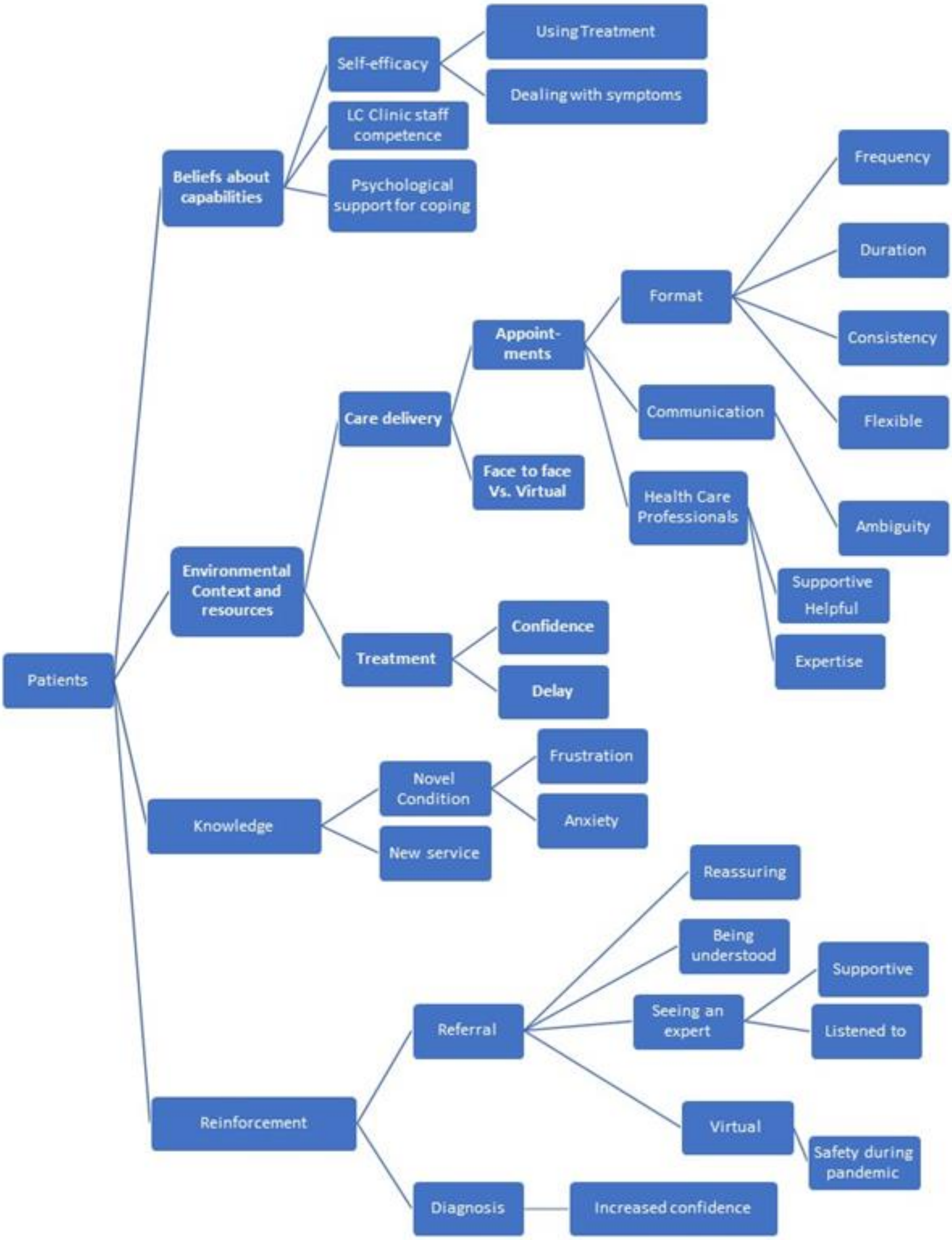

Simplified coding tree showing the four salient domains with connected subthemes emerging from staff members' interviews.

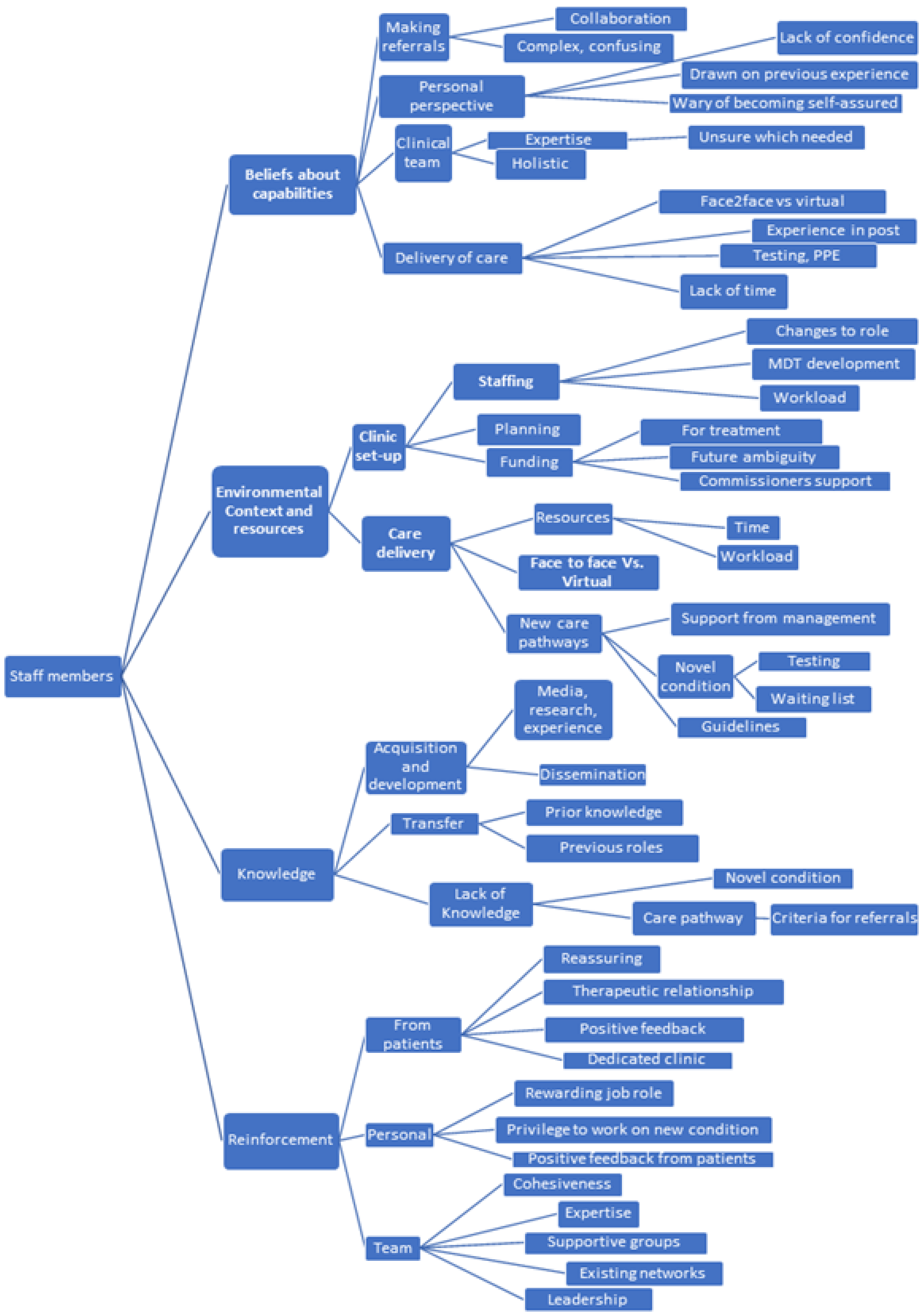

Supplement: S4 Text — (PDF) [file pone.0313367.s004.pdf]
