# Supplementary material for: A mixed-methods analysis of the implementation of a new community long-COVID service during the 2020 pandemic: Learning from practice
Source: PLoS One. 2026 Jun 26;21(6):e0313367. doi: 10.1371/journal.pone.0313367 (PMC13308792; doi:10.1371/journal.pone.0313367)
Supplement: S5 Table — (PDF) [file pone.0313367.s005.pdf]

Consolidated criteria for reporting qualitative studies (COREQ): 32-item checklist

No. Item	Guide questions/description	Description	Reported on Page #
**Domain 1: Research team and reflexivity**			
Personal Characteristics			
1. Interviewer/facilitator	Which author/s conducted the interview or focus group?	PW conducted the interviews	9
2. Credentials	What were the researcher's credentials? E.g. PhD, MD	PW has a PhD	9
3. Occupation	What was their occupation at the time of the study?	Academic Research Fellow	9
4. Gender	Was the researcher male or female?	Male	9
5. Experience and training	What experience or training did the researcher have?	PW had experience with qualitative methods including conducting interviews.	9
Relationship with participants			
6. Relationship established	Was a relationship established prior to study commencement?	PW was working within the clinic in a research capacity at the time of the study and therefore knew all participating clinicians. PW did not have a relationship with any of the patients prior to study commencement.	9

7. Participant knowledge of the interviewer	What did the participants know about the researcher? e.g. personal goals, reasons for doing the research	The reasons for PW doing the research were stated in the participant information sheet.	9
8. Interviewer characteristics	What characteristics were reported about the inter viewer/facilitator? e.g. Bias, assumptions, reasons and interests in the research topic	None	9

**Domain 2: study design**			
Theoretical framework			
9. Methodological orientation and Theory	What methodological orientation was stated to underpin the study? e.g. grounded theory, discourse analysis, ethnography, phenomenology, content analysis	Data were analysed using thematic analysis. Inductive open coding using a line-by-line process was first used to analyse the transcripts. Quotes were then deductively allocated to one of 14-domains specified within the Theoretical Domains Framework.	12
Participant selection			
10. Sampling	How were participants selected? e.g. purposive, convenience, consecutive, snowball	A combination of purposive and convenience sampling was used, the latter was used predominantly for healthcare professionals due to the availability of HCPs present within the clinic at the time of the study. Where possible, purposive sampling was used to ensure both males and females, different ages and different professions were represented.	9
11. Method of approach	How were participants approached? e.g. face-to-face, telephone, mail, email	Participants were approached to participate in the research via email and/or telephone	9
12. Sample size	How many participants were in the study?	5 patients.<br>6 healthcare professionals.	9
13. Non-participation	How many people refused to participate or dropped out? Reasons?	Of 218 patients referred into the clinic, five patients agreed to participate in the interview. 116 patients agreed for their quantitative data to be analysed for the study. All HCPs approached agreed to participate in the interview.	8-9
Setting			

14. Setting of data collection	Where was the data collected? e.g. home, clinic, workplace	The data were collected within a long-COVID clinic. Interviews took place online.	7-8
15. Presence of non-participants	Was anyone else present besides the participants and researchers?	No	9-10
16. Description of sample	What are the important characteristics of the sample? e.g. demographic data, date	Participant characteristics are included in table 1.	14-15
Data collection			
17. Interview guide	Were questions, prompts, guides provided by the authors? Was it pilot tested?	A topic guide for HCPs and patients are provided as supplemental material.	-
18. Repeat interviews	Were repeat interviews carried out? If yes, how many?	No repeat interviews were conducted.	10
19. Audio/visual recording	Did the research use audio or visual recording to collect the data?	The interviews were conducted online and were recorded.	9
20. Field notes	Were field notes made during and/or after the inter view or focus group?	Field notes were made during the interviews.	10
21. Duration	What was the duration of the inter views or focus group?	HCP and patient interviews lasted for approximately 60 minutes.	10
22. Data saturation	Was data saturation discussed?	After interviewing five patients and six clinicians, the research team were satisfied that data saturation had been reached, recruitment was then ceased	10
23. Transcripts returned	Were transcripts returned to participants for comment and/or correction?	No.	10
**Domain 3: analysis and findings**			

Data analysis			
24. Number of data coders	How many data coders coded the data?	Two coders (PW, AC) independently coded the first two interviews. Following this, the rest of the interviews were analysed by three coders (PW, AC, EB).	12
25. Description of the coding tree	Did authors provide a description of the coding tree?	Coding trees for HCP and patient interviews are provided as supplemental material.	-
26. Derivation of themes	Were themes identified in advance or derived from the data?	Inductive open coding using a line-by-line process was first used to analyse the transcripts to derive themes. Quotes were then deductively allocated to one of 14-domains specified within the Theoretical Domains Framework.	12
27. Software	What software, if applicable, was used to manage the data?	NVivo version 12.	12
28. Participant checking	Did participants provide feedback on the findings?	No.	12
Reporting			
29. Quotations presented	Were participant quotations presented to illustrate the themes/findings? Was each quotation identified? e.g. participant number	Key findings of this study are supported with selected quotes in text. Participant numbers are assigned to individual quotes.	21-26
30. Data and findings consistent	Was there consistency between the data presented and the findings?	Yes. The findings, including themes and subthemes, were derived from the data. This is supported in the text by illustrative quotes.	Results: 18-26<br>Discussion:28-34

31. Clarity of major themes	Were major themes clearly presented in the findings?	Major themes are clearly presented in the results section.	18-26
32. Clarity of minor themes	Is there a description of diverse cases or discussion of minor themes?	Description of diverse cases are presented in the discussion, see table 3 (barriers and facilitators)	28-34
