# Supplementary material for: A mixed-methods analysis of the implementation of a new community long-COVID service during the 2020 pandemic: Learning from practice
Source: PLoS One. 2026 Jun 26;21(6):e0313367. doi: 10.1371/journal.pone.0313367 (PMC13308792; doi:10.1371/journal.pone.0313367)
Supplement: S6 File — (PDF) [file pone.0313367.s006.pdf]

anonID	age	sex	Depriveda	deprivatio	ethnicity	ethnicity	Ethnicity	smokingst	smoking	Dateofrefe	month re	discharge	primaryreasonforreferral	sourceofreferral	source	referralson	totaluniqu	totalreferr	ReferraltoGP	Referralto	Referralto	Referralto	Referralto	Referralto	Referralto	Referralto	Referralto	Referralto	Neurology	Physio	cardiology	otherRefe	COVID19h	hospitalisa	COVID19o	year onse	month or	COVID19testresults
patient 0007	74	1.00	Non Depri	1.00	White Brit	1.00	1.00	Ex-smoker	2.00	03/08/202	8.00		Respiratory Conditions	General Practitioner	3.00	Referral to community-based nurse, dietitian, occupational therapist, psyc	4	4	0	0	1	1	0	1	0	0	0	1	0	0	0	0	yes	1.00	28.03.202	2.00	3.00	not availabe suspected covid
Patient 0008	45	1.00	Non Depri	1.00	White Brit	1.00	1.00	Never sm	3.00	04/08/202	8.00		Chronic Fatigue Syndrome	General Practitioner	3.00	referral to GP	1	1	1	0	0	0	0	0	0	0	0	0	0	0	0	0	yes - non I	2.00	21-04-202	2.00	4.00	yes
Patient 0009	39	1.00	Non Depri	1.00	White Brit	1.00	1.00	Never sm	3.00	04/08/202	8.00		Respiratory Conditions	General Practitioner	3.00	other referral, referral to GP, referral to pulmonary rehabilitation	3	3	1	1	0	0	0	0	0	0	0	0	0	0	0	1	no	3.00	20-03-20	2.00	3.00	too early for testing
Patient 0011	55	1.00	Non Depri	1.00	White Brit	1.00	1.00	Never sm	3.00	44047.790	8.00		Respiratory Conditions	General Practitioner	3.00	pulmonary rehabilitation,	1	1	0	1	0	0	0	0	0	0	0	0	0	0	0	0	yes - non I	2.00	43922	2.00	4.00	
Patient 0013	44	1.00	Non Depri	1.00	White Brit	1.00	1.00	Never sm	3.00	44052.396	8.00		Respiratory Conditions	General Practitioner	3.00	referral to GP, pulmonary rehabilitation	1	1	1	1	0	0	0	0	0	0	0	0	0	0	0	0	yes - non I	2.00	28/03/202	2.00	3.00	positive test - 02 April
Patient 0014	41	1.00	Non Depri	1.00	White Brit	1.00	1.00	Never sm	3.00	44052.404	8.00		Respiratory Conditions	General Practitioner	3.00	Referral to GP, referral to pulmonary rehabilitation	2	2	1	1	0	0	0	0	0	0	0	0	0	0	0	0	no	3.00	43862	2.00	2.00	antibody test - 07.2020
Patient 0016	48	1.00	Non Depri	1.00	White Brit	1.00	1.00	Never sm	3.00	44055.402	8.00		Respiratory Conditions	General Practitioner	3.00	other referral, referral to pulmonary rehabilitation	2	2	0	1	0	0	0	0	0	0	0	0	0	0	0	1	no	3.00	20.03.202	2.00	3.00	
Patient 0017	52	2.00	Non Depri	1.00	White Brit	1.00	1.00	Never sm	3.00	44055.403	8.00		Respiratory Conditions	General Practitioner	3.00	referral to chronic fatigue syndrome specialist team, pulmonary rehabilita	2	2	0	1	0	0	0	0	0	0	1	0	0	0	0	0	yes - non I	2.00	43878	2.00	2.00	too early for testing
patient 0018	53	2.00	Non Depri	1.00	White Brit	1.00	1.00	Smoker	1.00	12/08/202	8.00		Respiratory Conditions	Self referral	6.00	referral to GP, pulmonary rehabilitation	2	2	1	1	0	0	0	0	0	0	0	0	0	0	0	0		3.00	23.03.202	2.00	3.00	09.07.2020
patient 0019	43	2.00	Non Depri	1.00	White Brit	1.00	1.00	Never sm	3.00	44056.557	8.00		Respiratory Conditions	General Practitioner	3.00	other	1	1	0	0	0	0	0	0	0	0	0	0	0	0	0	1	no	3.00	43831	2.00	1.00	too early for testing
Patient 0020	42	1.00	Non Depri	1.00	White Brit	1.00	1.00	Never sm	3.00	13/08/202	8.00		Respiratory Conditions	General Practitioner	3.00	pulmonary rehabilitation,	1	1	0	1	0	0	0	0	0	0	0	0	0	0	0	0	yes - non I	2.00	43865	2.00	2.00	positive test
Patient 0028	40	1.00	Non Depri	1.00	British or	6.00	0.00	Ex-smoker	2.00	17/08/202	8.00		Emotional/Behavioural Problems	Acute Hospital Inpatients	1.00	Referral to GP	1	1	1	0	0	0	0	0	0	0	0	0	0	0	0	0	yes	1.00	43922	2.00	4.00	
Patient 0029	44	1.00	Non Depri	1.00	White Brit	1.00	1.00	Never sm	3.00	17/08/202	8.00		Respiratory Conditions	Acute Hospital Inpatients	1.00	pulmonary rehabilitation,	1	1	0	1	0	0	0	0	0	0	0	0	0	0	0	0	yes	1.00	16-03-202	2.00	3.00	not tested
patient 0030	48	2.00	Non Depri	1.00	Ethnic cat	8.00	0.00	Never sm	3.00	44060.544	8.00		Triage	Acute Hospital Inpatients	1.00	none	0	0	0	0	0	0	0	0	0	0	0	0	0	0	0	0	yes-non IC	2.00		#NULL!	#NULL!	
Patient 0032	55	1.00	Non Depri	1.00	White Brit	1.00	1.00	Never sm	3.00	17/08/202	8.00		Respiratory Conditions	General Practitioner	3.00	pulmonary rehabilitation,	1	1	0	1	0	0	0	0	0	0	0	0	0	0	0	0	no	3.00	43922	2.00	4.00	
Patient 0033	64	2.00	Non Depri	1.00	Ethnic cat	8.00	0.00	Never sm	3.00	17/08/202	8.00		Problems with activities of daily living	Acute Hospital Inpatients	1.00	none	0	0	0	0	0	0	0	0	0	0	0	0	0	0	0	0	yes	1.00	44640	2.00	3.00	
Patient 0035	75	2.00	Non Depri	1.00	White Brit	1.00	1.00	Never sm	3.00	17/08/202	8.00		Respiratory Conditions	Acute Hospital Inpatients	1.00	none	0	0	0	0	0	0	0	0	0	0	0	0	0	0	0	0	yes	1.00	14-03-202	2.00	3.00	positive swab
Patient 0036	31	1.00	Non Depri	1.00	White Brit	1.00	1.00	Never sm	3.00	44064	8.00		Respiratory Conditions	General Practitioner	3.00	pulmonary rehabilitation,	1	1	0	1	0	0	0	0	0	0	0	0	0	0	0	0	no	3.00	16-04-202	2.00	4.00	
Patient 0037	39	2.00	Deprived	2.00	Indian or	4.00	0.00	Never sm	3.00	44064.606	8.00		Respiratory Conditions	General Practitioner	3.00	other referral	1	1	0	0	0	0	0	0	0	0	0	0	0	0	0	1	no	3.00	43922	2.00	4.00	
Patient 0038	61	1.00	Non Depri	1.00	White Brit	1.00	1.00	Never sm	3.00	44064.610	8.00		Chronic Fatigue Syndrome	General Practitioner	3.00	referral to GP, pulmonary	1	1	1	1	0	0	0	0	0	0	0	0	0	0	0	0	no	3.00	43949	2.00	4.00	postive test
Patient 0039	40	2.00	Non Depri	1.00	White Brit	1.00	1.00	Never sm	3.00	44064.612	8.00		Respiratory Conditions	General Practitioner	3.00	pulmonary rehabilitation,	1	1	0	1	0	0	0	0	0	0	0	0	0	0	0	0	no	3.00	20-03-20	2.00	3.00	
Patient 0042	29	1.00	Non Depri	1.00	White Brit	1.00	1.00	Ex-smoker	2.00	44064.629	8.00		Respiratory Conditions	General Practitioner	3.00	pulmonary rehabilitation, chronic fatigue syndrome specialist team	2	2	0	1	0	0	0	0	0	0	1	0	0	0	0	0	no	3.00	14.04.202	2.00	4.00	negative test on 14th April
Patient 0043	64	1.00	Non Depri	1.00	Other Mix	7.00	0.00	Never sm	3.00	44064.635	8.00		Respiratory Conditions	General Practitioner	3.00	Referral to mental health team	1	1	0	0	0	0	1	0	0	0	0	0	0	0	0	0	yes	1.00		#NULL!	#NULL!	
Patient 0045	54	2.00	Non Depri	1.00	White Brit	1.00	1.00	Never sm	3.00	44069.390	8.00		Respiratory Conditions	General Practitioner	3.00	referral to GP, pulmonary rehabilitation, respiratory physician	3	3	1	1	0	0	0	0	0	1	0	0	0	0	0	0	no	3.00		#NULL!	#NULL!	
Patient 0046	43	1.00	Non Depri	1.00	White Brit	1.00	1.00	Never sm	3.00	44069.392	8.00		Respiratory Conditions	General Practitioner	3.00	referral to pulmonary rehabilitation	1	1	0	1	0	0	0	0	0	0	0	0	0	0	0	0	no	3.00	18.04.202	2.00	4.00	
Patient 0047	67	1.00	Non Depri	1.00	White Brit	1.00	1.00	Never sm	3.00	44069.436	8.00		Respiratory Conditions	General Practitioner	3.00	referral to pulmonary rehabilitation	1	1	0	1	0	0	0	0	0	0	0	0	0	0	0	0	no	3.00		#NULL!	#NULL!	
Patient 0049	59	1.00	Non Depri	1.00	White Brit	1.00	1.00	Never sm	3.00	44070.536	8.00		Respiratory Conditions	HCT Community Service	4.00	referral to GP, pulmonary rehab, chronic fatigue syndrome, respiratory nu	4	4	1	1	0	0	0	0	1	0	1	0	0	0	0	0	no	3.00	16.03.202	2.00	3.00	
Patient 0050	67	2.00	Non Depri	1.00	White Brit	1.00	1.00	Never sm	3.00	44071.370	8.00		Mobility problems	HCT Community Service	4.00	CFS, GP	2	2	1	0	0	0	0	0	0	0	1	0	0	0	0	0	yes	1.00	25.03.202	2.00	3.00	positive test - 25.03
Patient 0052	62	2.00	Non Depri	1.00	White Brit	1.00	1.00	Never sm	3.00	44071.484	8.00		Respiratory Conditions	Acute Hospital Inpatients	1.00	pulmonary rehabilitation,	1	1	0	1	0	0	0	0	0	0	0	0	0	0	0	0	yes	1.00	03.04.202	2.00	4.00	swabbed at hospital
Patient 0054	61	1.00	Non Depri	1.00	White Brit	1.00	1.00	Never sm	3.00	44071.490	8.00		Respiratory Conditions	Acute Hospital Inpatients	1.00	pulmonary rehabilitation,	1	1	0	1	0	0	0	0	0	0	0	0	0	0	0	0	yes - non I	2.00		#NULL!	#NULL!	
Patient 0059	57	2.00	Non Depri	1.00	White Brit	1.00	1.00	Never sm	3.00	44076.622	9.00		Chronic Fatigue Syndrome	General Practitioner	3.00	occupatonal therapy, pulmonary, cfs	2	2	0	1	0	0	0	1	0	0	1	0	0	0	0	0	yes	1.00	43922	2.00	4.00	43925
Patient 0063	54	2.00	Non Depri	1.00	White Brit	1.00	1.00	Never sm	3.00	44078.477	9.00		Respiratory Conditions	General Practitioner	3.00	respiratory nurse, respiratory physician	2	2	0	0	0	0	0	0	1	1	0	0	0	0	0	0	no	3.00		#NULL!	#NULL!	
Patient 0065	33	1.00	Non Depri	1.00	White Brit	1.00	1.00	Never sm	3.00	44078.482	9.00		Neurological problems	General Practitioner	3.00	other referral	1	1	0	0	0	0	0	0	0	0	0	0	0	0	0	1	no	3.00	24.04.202	2.00	4.00	no test
Patient 0066	35	1.00	Non Depri	1.00	Indian or	4.00	0.00	Never sm	3.00	44082.362	9.00		Respiratory Conditions	General Practitioner	3.00	referral to pulmonary rehabilitation	1	1	0	1	0	0	0	0	0	0	0	0	0	0	0	0	no	3.00	43922	2.00	4.00	
Patient 0068	50	1.00	Non Depri	1.00	White Brit	1.00	1.00	Ex-smoker	2.00	44082.366	9.00		Respiratory Conditions	General Practitioner	3.00	referral to GP, occupational therapy, pulmonary rehabilitation, psychologi	4	4	1	1	0	0	0	1	0	0	0	1	0	0	0	0	no	3.00	29.03.202	2.00	3.00	antibody test - 05.08.2020
Patient 0069	59	2.00	Non Depri	1.00	White Brit	1.00	1.00	Ex-smoker	2.00	44082.370	9.00		Respiratory Conditions	General Practitioner	3.00	referral to pulmonary rehabilitation	1	1	0	1	0	0	0	0	0	0	0	0	0	0	0	0	no	3.00		#NULL!	#NULL!	
Patient 0072	70	1.00	Non Depri	1.00	White Brit	1.00	1.00	Never sm	3.00	44084.396	9.00		Respiratory Conditions	Self referral	6.00	pulmonary rehabilitation,	1	1	0	1	0	0	0	0	0	0	0	0	0	0	0	0	yes - non I	2.00	24.03.202	2.00	3.00	no positive test
Patient 0075	32	2.00	Non Depri	1.00	Other Mix	7.00	0.00	Never sm	3.00	44085.379	9.00		Respiratory Conditions	General Practitioner	3.00	mental health team, pulmonary team, other	3	3	0	1	0	0	1	0	0	0	0	0	0	0	0	1	no	3.00		#NULL!	#NULL!	
Patient 0076	68	1.00	Non Depri	1.00	White Brit	1.00	1.00	Ex-smoker	2.00	44085.506	9.00		Chronic Fatigue Syndrome	General Practitioner	3.00	gp	1	1	1	0	0	0	0	0	0	0	0	0	0	0	0	0	no	3.00	23.03.202	2.00	3.00	too early for testing
Patient 0077	25	1.00	Non Depri	1.00	White Brit	1.00	1.00	Never sm	3.00	44088.646	9.00		Chronic Fatigue Syndrome	General Practitioner	3.00	gp, occupational therapy,	2	2	1	0	0	0	0	1	0	0	0	0	0	0	0	0	no	3.00	05.04.202	2.00	4.00	02.05.2020
Patient 0078	26	1.00	Non Depri	1.00	Other Whi	3.00	1.00	Never sm	3.00	44090.402	9.00		Respiratory Conditions	General Practitioner	3.00	pulmonary	1	1	0	1	0	0	0	0	0	0	0	0	0	0	0	0	no	3.00	43891	2.00	3.00	
Patient 0083	76	2.00	Non Depri	1.00	White Brit	1.00	1.00	Never sm	3.00	44092.502	9.00		Respiratory Conditions	HCT Community Service	4.00	pulmonary	1	1	0	1	0	0	0	0	0	0	0	0	0	0	0	0	yes	1.00		#NULL!	#NULL!	
Patient 0084	44	2.00	Non Depri	1.00	White Brit	1.00	1.00	Never sm	3.00	44093.799	9.00		Chronic Fatigue Syndrome	General Practitioner	3.00	referral to GP, pulmonary rehabilitation	2	2	1	1	0	0	0	0	0	0	0	0	0	0	0	0	no	3.00	01.04.202	2.00	4.00	
Patient 0086	65	1.00	Non Depri	1.00	White Brit	1.00	1.00	Never sm	3.00	44097.65	9.00		Cardiac Conditions	General Practitioner	3.00	gp, cardiology, respiratory physician	3	3	1	0	0	0	0	0	0	1	0	0	0	0	1	0	yes - non I	2.00	14.03.202	2.00	3.00	too early for testing
Patient 0087	51	1.00	Non Depri	1.00	White Brit	1.00	1.00	Never sm	3.00	44097.654	9.00		Chronic Fatigue Syndrome	General Practitioner	3.00	gp	1	1	1	0	0	0	0	0	0	0	0	0	0	0	0	0	no	3.00	09.04.202	2.00	4.00	12.04.2020 and 10.08.2020
Patient 0088	51	1.00	Non Depri	1.00	White Brit	1.00	1.00	Ex-smoker	2.00	44098.542	9.00		Chronic Fatigue Syndrome	General Practitioner	3.00	cfs, gp, occupational therapy	3	3	1	0	0	0	0	1	0	0	1	0	0	0	0	0	no	3.00	20.03.202	2.00	3.00	
Patient 0089	48	1.00	Non Depri	1.00	White Brit	1.00	1.00	Never sm	3.00	44099.498	9.00		Respiratory Conditions	General Practitioner	3.00	gp, occupational therapy, pulmonary	3	3	1	1	0	0	0	1	0	0	0	0	0	0	0	0	no	3.00	17.03.202	2.00	3.00	too early for testing
Patient 0090	56	1.00	Non Depri	1.00	White Brit	1.00	1.00	Never sm	3.00	44102.654	9.00		Chronic Fatigue Syndrome	General Practitioner	3.00	cfs, gp	2	2	1	0	0	0	0	0	0	0	1	0	0	0	0	0	no	3.00	03.03.202	2.00	3.00	too early for testing
Patient 0096	42	1.00	Non Depri	1.00	White Brit	1.00	1.00	Ex-smoker	2.00	44104.448	9.00		Chronic Fatigue Syndrome	General Practitioner	3.00	cfs, gp, pulmonary	3	3	1	1	0	0	0	0	0	0	1	0	0	0	0	0	no	3.00	03.08.202	2.00	8.00	negative - 03/08/2020
Patient 0097	57	1.00	Non Depri	1.00	White Brit	1.00	1.00	Never sm	3.00	44104.562	9.00		Respiratory Conditions	Acute Hospital Inpatients	1.00	gp, occupational therapy, pulmonary	3	3	1	1	0	0	0	1	0	0	0	0	0	0	0	0	yes	1.00	43920	2.00	3.00	negative - 27.04.2020
Patient 0098	54	1.00	Non Depri	1.00	White Brit	1.00	1.00	Never sm	3.00	44105.456	10.00		Respiratory Conditions	General Practitioner	3.00	cfs, gp, occupational therapy	3	3	1	0	0	0	0	1	0	0	1	0	0	0	0	0	no - para	3.00	24.03.202	2.00	3.00	too early for testing
Patient 0103	32	1.00	Non Depri	1.00	White Brit	1.00	1.00	Never sm	3.00	44111.470	10.00		Covid-19 Rehab	Chronic Fatigue Syndrome	2.00	cfs, pulmonary	2	2	0	1	0	0	0	0	0	0	1	0	0	0	0	0	no	3.00	22.03.202	2.00	3.00	too early for testing
Patient 0105	74	1.00	Non Depri	1.00	White Brit	1.00	1.00	Ex-smoker	2.00	44111.651	10.00		Covid-19 Rehab	Chronic Fatigue Syndrome	2.00	cfs, community-based nurse, dietitian, occupational therapist	4	4	0	0	1	1	0	1	0	0	1	0	0	0	0	0	yes - not i	2.00	not record	#NULL!	#NULL!	none
Patient 0106	56	1.00	Non Depri	1.00	British or	6.00	0.00	Ex-smoker	2.00	44111.748	10.00		Covid-19 Rehab	Chronic Fatigue Syndrome	2.00	cfs	1	1	0	0	0	0	0	0	0	0	1	0	0	0	0	0	no - para	3.00	43891	2.00	3.00	none
Patient 0107	55	2.00	Non Depri	1.00	White Brit	1.00	1.00	Ex-smoker	2.00	44113.552	10.00		Covid-19 Rehab	Respiratory Conditions	5.00	none	0	0	0	0	0	0	0	0	0	0	0	0	0	0	0	0	no	3.00	43891	2.00	3.00	none
Patient 0108	80	1.00	Non Depri	1.00	White Brit	1.00	1.00	Never sm	3.00	44118.478	10.00		Covid-19 Rehab	Chronic Fatigue Syndrome	2.00	cfs	1	1	0	0	0	0	0	0	0	0	1	0	0	0	0	0	no	3.00	feb/mar 2	2.00	2.00	none
Patient 0110	52	2.00	Non Depri	1.00	White Brit	1.00	1.00	Never sm	3.00	44118.488	10.00		Covid-19 Rehab	Respiratory Conditions	5.00	cfs	1	1	0	0	0	0	0	0	0	0	1	0	0	0	0	0	yes - non i	2.00	43891	2.00	3.00	none
Patient 0111	52	1.00	Non Depri	1.00	White Brit	1.00	1.00	Never sm	3.00	44118.497	10.00		Covid-19 Rehab	Respiratory Conditions	5.00	gp	1	1	1	0	0	0	0	0	0	0	0	0	0	0	0	0	no	3.00	25.03.202	2.00	3.00	too early for testing
Patient 0114	39	1.00	Non Depri	1.00	Other Whi	3.00	1.00	Never sm	3.00	44119.640	10.00		Respiratory Conditions	General Practitioner	3.00	occupational therapy, pulmonary, occupational therapy, pulmonary, comr	3	5	0	2	1	0	0	2	0	0	0	0	0	0	0	0	no	3.00	43891	2.00	3.00	none
Patient 0116	70	1.00	Non Depri	1.00	White Brit	1.00	1.00	Never sm	3.00	44120.691	10.00		Respiratory Conditions	General Practitioner	3.00	pulmonary	1	1	0	1	0	0	0	0	0	0	0	0	0	0	0	0	no	3.00	15.03.20	2.00	3.00	none
Patient 0120	25	1.00	Non Depri	1.00	White Brit	1.00	1.00	Never sm	3.00	44120.736	10.00		Respiratory Conditions	General Practitioner	3.00	gp	1	1	1	0	0	0	0	0	0	0	0	0	0	0	0	0	no	3.00	15.03.202	2.00	3.00	06.06.20 positive test
Patient 0121	77	2.00	Non Depri	1.00	White Brit	1.00	1.00	Ex-smoker	2.00	44123.606	10.00		Chronic Fatigue Syndrome	Self referral	6.00	pulmonary	1	1	0	1	0	0	0	0	0	0	0	0	0	0	0	0	yes - non I	2.00	44097	2.00	9.00	positive test
Patient 0122	25	2.00	Non Depri	1.00	White Brit	1.00	1.00	Smoker	1.00	44123.620	10.00		Respiratory Conditions	General Practitioner	3.00	dietitian, pulmonary	2	2	0	1	0	1	0	0	0	0	0	0	0	0	0	0	yes - PAHT	1.00	01.03.202	2.00	3.00	none
Patient 0123	31	1.00	Non Depri	1.00	White Brit	1.00	1.00	Never sm	3.00	44123.622	10.00		Triage	General Practitioner	3.00	pulmonary	1	1	0	1	0	0	0	0	0	0	0	0	0	0	0	0	no	3.00	43922	2.00	4.00	
Patient 0124	65	1.00	Non Depri	1.00	Indian or	4.00	0.00	Never sm	3.00	44123.623	10.00		Respiratory Conditions	General Practitioner	3.00	gp	1	1	1	0	0	0	0	0	0	0	0	0	0	0	0	0	no	3.00	43922	2.00	4.00	
Patient 0125	54	1.00	Non Depri	1.00	White Brit	1.00	1.00	Ex-smoker	2.00	44123.625	10.00		Triage	General Practitioner	3.00	pulmonary	1	1	0	1	0	0	0	0	0	0	0	0	0	0	0	0	no	3.00	43922	2.00	4.00	+ in may (antibody
Patient 0127	47	1.00	Non Depri	1.00	Ethnic cat	8.00	0.00	Refusal to	4.00	44123.635	10.00		Respiratory Conditions	Acute Hospital Inpatients	1.00	mental health team, ocupational therapy	2	2	0	0	0	0	1	1	0	0	0	0	0	0	0	0	yes - ICU	1.00	21.03.202	2.00	3.00	31.03.2020 negative. early april neg
Patient 0128	39	1.00	Non Depri	1.00	White Brit	1.00	1.00	Never sm	3.00	44124.411	10.00		Respiratory Conditions	Acute Hospital Inpatients	1.00	gp, mental health team, occupational therapy	3	3	1	0	0	0	1	1	0	0	0	0	0	0	0	0	outpatient	2.00	43891	2.00	3.00	later test (May) negative
Patient 0130	52	1.00	Non Depri	1.00	White Brit	1.00	1.00	Never sm	3.00	44127.495	10.00		Respiratory Conditions	HCT Community Service	4.00	gp, pulmonary, physiotherapy	3	3	1	1	0	0	0	0	0	0	0	0	0	1	0	0	no	3.00	19.03.202	2.00	3.00	no
Patient 0131	42	1.00	Non Depri	1.00	White Brit	1.00	1.00	Never sm	3.00	44127.749	10.00		Respiratory Conditions	HCT Community Service	4.00	GP, psychologist, pulmonary, gp, psychologist, pulmonary, cardiology, resp	5	8	2	2	0	0	0	0	1	0	0	2	0	0	1	0	no - rang 1	4.00	14.03.202	2.00	3.00	too early
Patient 0134	47	1.00	Non Depri	1.00	British or	6.00	0.00	Ex-smoker	2.00	44130.650	10.00		Chronic Fatigue Syndrome	General Practitioner	3.00	mental health team	1	1	0	0	0	0	1	0	0	0	0	0	0	0	0	0	no	3.00	43891	2.00	3.00	positive - 11.6.20. Negative - 30.04.
Patient 0135	78	2.00	Non Depri	1.00	British or	6.00	0.00	Ex-smoker	2.00	44130.654	10.00	Jan 2021	Problems with activities of daily living	General Practitioner	3.00	none	0	0	0	0	0	0	0	0	0	0	0	0	0	0	0	0	no data re	0.00		#NULL!	#NULL!	
Patient 0136	75	1.00	Non Depri	1.00	White Brit	1.00	1.00	Smoker	1.00	44130.659	10.00		Chronic Fatigue Syndrome	General Practitioner	3.00	occupational therapist, physiotherapist	2	2	0	0	0	0	0	1	0	0	0	0	0	1	0	0	no	3.00	43891	2.00	3.00	none
Patient 0137	64	1.00	Non Depri	1.00	White Brit	1.00	1.00	Never sm	3.00	44130.660	10.00		Chronic Fatigue Syndrome	General Practitioner	3.00	cfs	1	1	0	0	0	0	0	0	0	0	1	0	0	0	0	0	yes - non I	2.00	30.03.202	2.00	3.00	positive
Patient 0138	66	1.00	Non Depri	1.00	Not State	8.00	0.00	Never sm	3.00	44132.647	10.00	Dec 2020	Chronic Fatigue Syndrome	General Practitioner	3.00	gp, occupational therapy	2	2	1	0	0	0	0	1	0	0	0	0	0	0	0	0	no	3.00	23.03.202	2.00	3.00	too early for testing
Patient 0140	49	1.00	Non Depri	1.00	White Brit	1.00	1.00	Ex-smoker	2.00	44133.382	10.00		Triage	General Practitioner	3.00	gp	1	1	1	0	0	0	0	0	0	0	0	0	0	0	0	0	no - refuse	3.00	01.03.202	2.00	3.00	01.03.2020
Patient 0142	43	1.00	Non Depri	1.00	White Brit	1.00	1.00	Never sm	3.00	44133.606	10.00		Respiratory Conditions	Acute Hospital Inpatients	1.00	psychologist, pulmonary	2	2	0	1	0	0	0	0	0	0	0	1	0	0	0	0	no	3.00	43922	2.00	4.00	no
Patient 0144	55	1.00	Non Depri	1.00	White Brit	1.00	1.00	Never sm	3.00	44137.573	11.00		Respiratory Conditions	General Practitioner	3.00	occupational, psychologist, pulmonary	3	3	0	1	0	0	0	1	0	0	0	1	0	0	0	0	yes	1.00	43556	2.00	4.00	positive test
Patient 0147	82	2.00	Non Depri	1.00	British or	6.00	0.00	Smoking S	4.00	44139.351	11.00		Triage	General Practitioner	3.00	none	0	0	0	0	0	0	0	0	0	0	0	0	0	0	0	0	no	3.00	43898	2.00	3.00	psotive test
Patient 0148	51	1.00	Non Depri	1.00	White Brit	1.00	1.00	Never sm	3.00	44139.360	11.00		Respiratory Conditions	General Practitioner	3.00	pulmonary	1	1	0	1	0	0	0	0	0	0	0	0	0	0	0	0	no	3.00	43927	2.00	4.00	no
Patient 0149	57	1.00	Non Depri	1.00	White Brit	1.00	1.00	Never sm	3.00	44139.441	11.00		Respiratory Conditions	General Practitioner	3.00	cfs, pulmonary	2	2	0	1	0	0	0	0	0	0	1	0	0	0	0	0	no	3.00	43891	2.00	2.00	too early
Patient 0150	45	1.00	Non Depri	1.00	White Brit	1.00	1.00	Never sm	3.00	44139.682	11.00		Respiratory Conditions	General Practitioner	3.00	pulmonary	1	1	0	1	0	0	0	0	0	0	0	0	0	0	0	0	no	3.00	5.4.20	2.00	4.00	positive
Patient 0153	50	1.00	Non Depri	1.00	White Brit	1.00	1.00	Never sm	3.00	44140.683	11.00		Respiratory Conditions	General Practitioner	3.00	gp, mental health team, pulmonary, pulmonary, dietitian, pulmonary	4	6	1	3	0	1	1	0	0	0	0	0	0	0	0	0	no	3.00	43891	2.00	3.00	june-20 positive
Patient 0156	41	1.00	Non Depri	1.00	Other Whi	3.00	1.00	Never sm	3.00	44146.514	11.00		Cardiac Conditions	General Practitioner	3.00	cfs, gp, mental health, cardiology	4	4	1	0	0	0	1	0	0	0	1	0	0	0	1	0	yes - non I	2.00	22.10.20	2.00	10.00	25.10.20 positve
Patient 0157	58	1.00	Non Depri	1.00	White Iris	2.00	1.00	Ex-smoker	2.00	44146.651	11.00		Chronic Fatigue Syndrome	General Practitioner	3.00	cfs, gp, pulmonary	3	3	1	1	0	0	0	0	0	0	1	0	0	0	0	0	a&e no ad	4.00	12.12.201	1.00	12.00	too early
Patient 0159	19	1.00	Non Depri	1.00	White Brit	1.00	1.00	Never sm	3.00	44147.448	11.00		Chronic Fatigue Syndrome	General Practitioner	3.00	gp	1	1	1	0	0	0	0	0	0	0	0	0	0	0	0	0	yes - a&e t	2.00	28.9.20	2.00	9.00	1.10.20
Patient 0164	54	1.00	Non Depri	1.00	White Brit	1.00	1.00	Never sm	3.00	44151.624	11.00		Problems with activities of daily living	General Practitioner	3.00	gp, pulmonary	2	2	1	1	0	0	0	0	0	0	0	0	0	0	0	0	no	3.00	43908	2.00	3.00	
Patient 0165	66	1.00	Non Depri	1.00	White Iris	2.00	1.00	Never sm	3.00	44151.637	11.00		Cardiac Conditions	General Practitioner	3.00	gp	1	1	1	0	0	0	0	0	0	0	0	0	0	0	0	0	a&e rushe	4.00	09.09.20	2.00	9.00	pos
Patient 0166	39	1.00	Non Depri	1.00	White Brit	1.00	1.00	Never sm	3.00	44151.789	11.00		Problems with activities of daily living	General Practitioner	3.00	none	0	0	0	0	0	0	0	0	0	0	0	0	0	0	0	0	home	3.00	1.4.20	2.00	4.00	
Patient 0167	71	2.00	Non Depri	1.00	White Brit	1.00	1.00	Ex-smoker	2.00	44152.518	11.00		Respiratory Conditions	General Practitioner	3.00	pulmonary, gp, pulmonary, psychologist, respiratory nurse	4	5	1	2	0	0	0	0	1	0	0	1	0	0	0	0	yes - given	2.00	19.10.20	2.00	4.00	psoitve
Patient 0168	28	2.00	Non Depri	1.00	White Brit	1.00	1.00	Never sm	3.00	44153.574	11.00		Triage	General Practitioner	3.00	none	0	0	0	0	0	0	0	0	0	0	0	0	0	0	0	0	no	3.00	43891	2.00	10.00	psotive
Patient 0169	30	1.00	Non Depri	1.00	White Brit	1.00	1.00	Never sm	3.00	44153.615	11.00		Triage	General Practitioner	3.00	cfs, pulmonary	2	2	0	1	0	0	0	0	0	0	1	0	0	0	0	0	no	3.00	43891	2.00	3.00	
Patient 0172	56	2.00	Non Depri	1.00	White Brit	1.00	1.00	Never sm	3.00	44155.470	11.00		Chronic Fatigue Syndrome	General Practitioner	3.00	occupational therapist, psychologist, pulmonary, community-based nurse	4	4	0	1	1	0	0	1	0	0	0	1	0	0	0	0	no	3.00	43891	2.00	3.00	neg aug 20
Patient 0173	58	1.00	Non Depri	1.00	White Brit	1.00	1.00	Never sm	3.00	44155.778	11.00		Respiratory Conditions	General Practitioner	3.00	psychologist, pulmonary	2	2	0	1	0	0	0	0	0	0	0	1	0	0	0	0	yes -	1.00	43922	2.00	4.00	positive
Patient 0174	48	1.00	Non Depri	1.00	White Brit	1.00	1.00	Ex-smoker	2.00	44158.525	11.00		Respiratory Conditions	Self referral	6.00	GP, pulmonary	2	2	1	1	0	0	0	0	0	0	0	0	0	0	0	0	no	3.00	11.04.20	2.00	4.00	pos 14.04
Patient 0179	43	1.00	Non Depri	1.00	White Brit	1.00	1.00	Never sm	3.00	44160.512	11.00		Respiratory Conditions	General Practitioner	3.00	other	1	1	0	0	0	0	0	0	0	0	0	0	0	0	0	1	no	3.00	13.03.20	2.00	3.00	too earlu
Patient 0180	69	2.00	Non Depri	1.00	British or	6.00	0.00	Never sm	3.00	44160.559	11.00	Nov 2020	Problems with activities of daily living	Acute Hospital Inpatients	1.00	none	0	0	0	0	0	0	0	0	0	0	0	0	0	0	0	0	no data	3.00		#NULL!	#NULL!	
Patient 0181	42	2.00	Non Depri	1.00	White Brit	1.00	1.00	Never sm	3.00	44160.598	11.00		Medication Management/Administration	General Practitioner	3.00	none	0	0	0	0	0	0	0	0	0	0	0	0	0	0	0	0	no	3.00	18.03.20	2.00	3.00	too early.2nd june positive antibod
Patient 0183	27	2.00	Non Depri	1.00	White Brit	1.00	1.00	Never sm	3.00	44161.501	11.00		Respiratory Conditions	General Practitioner	3.00	gp, psychologist, pulmonary, cardiology, cfs	5	5	1	1	0	0	0	0	0	0	1	1	0	0	1	0	a&e but n	4.00	13.10.20	2.00	10.00	16.10.20
Patient 0184	40	1.00	Non Depri	1.00	White Brit	1.00	1.00	Never sm	3.00	44161.746	11.00		Triage	General Practitioner	3.00	none	0	0	0	0	0	0	0	0	0	0	0	0	0	0	0	0	no	3.00	43891	2.00	3.00	no
Patient 0186	83	2.00	Non Depri	1.00	White Brit	1.00	1.00	Never sm	3.00	44165.499	11.00		Problems with activities of daily living	HCT Community Service	4.00	none	0	0	0	0	0	0	0	0	0	0	0	0	0	0	0	0	yes -	1.00	17.11	2.00	11.00	pos - 17.11
Patient 0189	28	2.00	Non Depri	1.00	White Brit	1.00	1.00	Never sm	3.00	44166.668	12.00		Triage	General Practitioner	3.00	pulmonary, gp	2	2	1	1	0	0	0	0	0	0	0	0	0	0	0	0	no	3.00	1.3.20	2.00	3.00	too early
Patient 0192	81	1.00	Non Depri	1.00	White Brit	1.00	1.00	Ex-smoker	2.00	44167.643	12.00		Problems with activities of daily living	HCT Community Service	4.00	none	0	0	0	0	0	0	0	0	0	0	0	0	0	0	0	0	yes but no	2.00	30.11	2.00	11.00	contracted in hospital
Patient 0193	44	1.00	Deprived	2.00	White Brit	1.00	1.00	Smoker	1.00	44167.743	12.00		Triage	General Practitioner	3.00	none	0	0	0	0	0	0	0	0	0	0	0	0	0	0	0	0	no	3.00	44105	2.00	10.00	3rdoct
Patient 0194	43	1.00	Non Depri	1.00	Other whi	3.00	1.00	Never sm	3.00	44168.574	12.00		Triage	General Practitioner	3.00	none	0	0	0	0	0	0	0	0	0	0	0	0	0	0	0	0	no	3.00	1.4.20	2.00	4.00	too early
Patient 0195	52	1.00	Non Depri	1.00	White Brit	1.00	1.00	Never sm	3.00	44168.729	12.00		Respiratory Conditions	General Practitioner	3.00	none	0	0	0	0	0	0	0	0	0	0	0	0	0	0	0	0	no	3.00	43930	2.00	4.00	5/5/20 negative
Patient 0200	43	1.00	Non Depri	1.00	British or	6.00	0.00	Smoking S	4.00	44173.431	12.00		Triage	General Practitioner	3.00	none	0	0	0	0	0	0	0	0	0	0	0	0	0	0	0	0	a&e but n	4.00	1.03	2.00	3.00	too early. pos test jan 21, may 202
Patient 0201	48	1.00	Non Depri	1.00	Other Whi	3.00	1.00	Never sm	3.00	44173.437	12.00		Chronic Fatigue Syndrome	HCT Community Service	4.00	GP, neurology	2	2	1	0	0	0	0	0	0	0	0	0	1	0	0	0	no	3.00	8.3.20	2.00	3.00	too earlu
Patient 0202	41	1.00	Non Depri	1.00	White Brit	1.00	1.00	Never sm	3.00	44174.580	12.00		Chronic Fatigue Syndrome	General Practitioner	3.00	none	0	0	0	0	0	0	0	0	0	0	0	0	0	0	0	0	no	3.00	44136	2.00	11.00	positive
Patient 0204	43	2.00	Non Depri	1.00	British or	6.00	0.00	Ex-smoker	2.00	44175.527	12.00		Respiratory Conditions	General Practitioner	3.00	none	0	0	0	0	0	0	0	0	0	0	0	0	0	0	0	0	no	3.00	6.11	2.00	11.00	
Patient 0208	46	1.00	Non Depri	1.00	Caribbean	5.00	0.00	Never sm	3.00	44179.552	12.00		Respiratory Conditions	Acute Hospital Inpatients	1.00	pulmonary	1	1	0	1	0	0	0	0	0	0	0	0	0	0	0	0	no	3.00	mar/apr 2	2.00	3.00	no
Patient 0211	48	2.00	Non Depri	1.00	White Brit	1.00	1.00	Never sm	3.00	44182.562	12.00		Chronic Fatigue Syndrome	General Practitioner	3.00	gp, pulmonary	2	2	1	1	0	0	0	0	0	0	0	0	0	0	0	0	not admitt	4.00	16.04	2.00	4.00	too earlu, 16.july pos
Patient 0212	45	1.00	Non Depri	1.00	British or	6.00	0.00	Never sm	3.00	44186.561	12.00		Triage	General Practitioner	3.00	cfs, ocupational therapist, pulmonary	3	3	0	1	0	0	0	1	0	0	1	0	0	0	0	0	no	3.00	16.11	2.00	11.00	17.11
Patient 0214	50	2.00	Non Depri	1.00	White Brit	1.00	1.00	Never sm	3.00	44187.462	12.00		Respiratory Conditions	General Practitioner	3.00	gp, occupational therapist, pulmonary, community-based nurse	4	4	1	1	1	0	0	1	0	0	0	0	0	0	0	0	yes - IV	2.00	44626	2.00	3.00	too early
Patient 0215	29	1.00	Non Depri	1.00	White Brit	1.00	1.00	Never sm	3.00	44187.507	12.00		Triage	General Practitioner	3.00	none	0	0	0	0	0	0	0	0	0	0	0	0	0	0	0	0	no	3.00	12.08	2.00	8.00	no
Patient 0218	45	1.00	Non Depri	1.00	British or	6.00	0.00	Ex-smoker	2.00	44187.601	12.00		Triage	General Practitioner	3.00	none	0	0	0	0	0	0	0	0	0	0	0	0	0	0	0	0	no	3.00	6.3	2.00	3.00	1.5

test confi	COVID19ir	referral re	other initi	temperatu	cough	change sr	SOB	fatigue	pain ache	headache	sore throa	congestior	loss appet	diarrhoea	nausea	LC dizzine	LC abdo	LC chest	LC memo	LC insomn	LC cardio	LC joint	LC ear pr	LC menta	LC digesti	LC breath	LC fatigue	LC skin	LC tasteo	LC other	COVID19o	post disch	Heartprob	hypertens	breathing	diabetes	mentalhea	othercom	anxiety	depressior	stress	other MH	addiction	learning d	respirator	Asthma	COPD	Diabetes	hypertens	heart con	hyper hyp	cancer	skin cond	migranes
1.00	low appet	1.00	0.00	0.00	1.00	0.00	0.00	0.00	0.00	0.00	0.00	0.00	1.00	1.00	0.00	0.00	0.00	0.00	0.00	0.00	0.00	0.00	0.00	1.00	0.00	1.00	0.00	0.00	0.00	0.00		3.00	fast af with covid		ards, pulmonary fibrosis			breast can	0.00	0.00	0.00	0.00	0.00	0.00	1.00	0.00	0.00	0.00	0.00	1.00	0.00	1.00	0.00	0.00
3.00	temperatu	6.00	0.00	1.00	1.00	0.00	1.00	0.00	0.00	0.00	0.00	0.00	0.00	0.00	0.00	0.00	0.00	0.00	0.00	0.00	0.00	0.00	0.00	0.00	0.00	1.00	1.00	0.00	0.00	0.00		3.00							0.00	0.00	0.00	0.00	0.00	0.00	0.00	0.00	0.00	0.00	0.00	0.00	0.00	0.00	0.00	0.00
1.00	cough, unv	1.00	0.00	0.00	1.00	0.00	1.00	0.00	0.00	0.00	0.00	0.00	0.00	0.00	0.00	0.00	0.00	1.00	0.00	1.00	1.00	0.00	0.00	0.00	0.00	0.00	1.00	0.00	0.00	0.00	fatigue, sle	2.00			Asthma			Erlos-Danl	0.00	0.00	0.00	0.00	0.00	0.00	1.00	1.00	0.00	0.00	0.00	0.00	0.00	0.00	1.00	0.00
0.00	cough and	1.00	0.00	1.00	1.00	0.00	0.00	0.00	0.00	0.00	0.00	0.00	0.00	0.00	0.00	0.00	0.00	0.00	0.00	0.00	0.00	0.00	0.00	0.00	0.00	1.00	0.00	0.00	0.00	0.00		3.00							0.00	0.00	0.00	0.00	0.00	0.00	0.00	0.00	0.00	0.00	0.00	0.00	0.00	0.00	0.00	0.00
3.00	SOB, chest	1.00	0.00	1.00	0.00	0.00	1.00	0.00	0.00	0.00	0.00	0.00	0.00	0.00	0.00	0.00	0.00	1.00	0.00	0.00	0.00	0.00	0.00	0.00	0.00	1.00	0.00	0.00	0.00	0.00		3.00							0.00	0.00	0.00	0.00	0.00	0.00	0.00	0.00	0.00	0.00	0.00	0.00	0.00	0.00	0.00	0.00
3.00	breathless	1.00	0.00	1.00	1.00	1.00	1.00	1.00	1.00	0.00	0.00	0.00	0.00	0.00	0.00	0.00	0.00	1.00	0.00	1.00	0.00	0.00	0.00	1.00	0.00	1.00	0.00	0.00	0.00	0.00	stress and	2.00						migranes	0.00	0.00	0.00	0.00	0.00	0.00	0.00	0.00	0.00	0.00	0.00	0.00	0.00	0.00	0.00	1.00
0.00	sore throa	1.00	0.00	0.00	0.00	1.00	0.00	1.00	0.00	0.00	1.00	0.00	0.00	0.00	0.00	0.00	0.00	0.00	1.00	0.00	1.00	0.00	0.00	0.00	0.00	1.00	0.00	0.00	0.00	1.00	memory a	2.00						trigeminal	0.00	0.00	0.00	0.00	0.00	0.00	0.00	0.00	0.00	0.00	0.00	0.00	0.00	0.00	0.00	0.00
1.00	SOB on ex	1.00	0.00	0.00	1.00	0.00	1.00	1.00	0.00	0.00	0.00	0.00	0.00	0.00	0.00	1.00	0.00	0.00	1.00	0.00	0.00	0.00	0.00	0.00	0.00	1.00	0.00	0.00	0.00	0.00	still gets S(	2.00							0.00	0.00	0.00	0.00	0.00	0.00	0.00	0.00	0.00	0.00	0.00	0.00	0.00	0.00	0.00	0.00
3.00	loss of tast	1.00	0.00	0.00	0.00	1.00	0.00	1.00	1.00	1.00	0.00	0.00	0.00	1.00	0.00	0.00	1.00	0.00	0.00	0.00	0.00	0.00	0.00	0.00	0.00	1.00	0.00	0.00	0.00	0.00		3.00							0.00	0.00	0.00	0.00	0.00	0.00	0.00	0.00	0.00	0.00	0.00	0.00	0.00	0.00	0.00	0.00
1.00	sob, cough	1.00	0.00	1.00	1.00	0.00	1.00	0.00	0.00	0.00	0.00	0.00	0.00	0.00	0.00	0.00	0.00	0.00	0.00	0.00	0.00	0.00	0.00	0.00	0.00	1.00	0.00	0.00	0.00	0.00		3.00							0.00	0.00	0.00	0.00	0.00	0.00	0.00	0.00	0.00	0.00	0.00	0.00	0.00	0.00	0.00	0.00
3.00	short of br	1.00	0.00	1.00	1.00	0.00	1.00	0.00	0.00	0.00	0.00	0.00	0.00	0.00	1.00	1.00	1.00	1.00	0.00	0.00	0.00	0.00	0.00	0.00	0.00	1.00	0.00	0.00	0.00	0.00		3.00	PE		asthma		depressior	chronic sy	0.00	1.00	0.00	0.00	0.00	0.00	1.00	1.00	0.00	0.00	0.00	1.00	0.00	0.00	0.00	0.00
0.00	ventilated	4.00	0.00	0.00	0.00	0.00	0.00	0.00	0.00	0.00	0.00	0.00	0.00	0.00	0.00	0.00	0.00	0.00	0.00	0.00	0.00	0.00	0.00	1.00	0.00	0.00	0.00	0.00	0.00	1.00	intermitte	2.00						high BMI,	0.00	0.00	0.00	0.00	0.00	0.00	0.00	0.00	0.00	1.00	0.00	0.00	0.00	0.00	0.00	0.00
0.00	shortness	1.00	0.00	0.00	1.00	0.00	1.00	1.00	0.00	0.00	0.00	0.00	0.00	0.00	0.00	0.00	0.00	0.00	0.00	0.00	0.00	0.00	0.00	0.00	0.00	1.00	1.00	0.00	0.00	1.00		3.00			asthma		treated for	depressior	0.00	1.00	0.00	0.00	0.00	0.00	1.00	1.00	0.00	0.00	0.00	0.00	0.00	0.00	0.00	0.00
0.00	not record	5.00	0.00	0.00	0.00	0.00	0.00	0.00	0.00	0.00	0.00	0.00	0.00	0.00	0.00	0.00	0.00	0.00	0.00	0.00	0.00	0.00	0.00	0.00	0.00	0.00	0.00	0.00	0.00	0.00		3.00							0.00	0.00	0.00	0.00	0.00	0.00	0.00	0.00	0.00	0.00	0.00	0.00	0.00	0.00	0.00	0.00
0.00	cough and	1.00	0.00	0.00	1.00	0.00	1.00	0.00	0.00	0.00	0.00	0.00	0.00	0.00	0.00	0.00	0.00	0.00	0.00	0.00	0.00	0.00	0.00	0.00	0.00	1.00	0.00	0.00	0.00	0.00		3.00							0.00	0.00	0.00	0.00	0.00	0.00	0.00	0.00	0.00	0.00	0.00	0.00	0.00	0.00	0.00	0.00
0.00	fever, coug	3.00	0.00	1.00	1.00	0.00	0.00	0.00	0.00	0.00	0.00	0.00	0.00	0.00	0.00	0.00	0.00	0.00	0.00	0.00	0.00	0.00	0.00	0.00	0.00	0.00	0.00	0.00	0.00	0.00		3.00							0.00	0.00	0.00	0.00	0.00	0.00	0.00	0.00	0.00	0.00	0.00	0.00	0.00	0.00	0.00	0.00
3.00	not record	1.00	0.00	0.00	0.00	0.00	0.00	0.00	0.00	0.00	0.00	0.00	0.00	0.00	0.00	0.00	0.00	0.00	0.00	0.00	0.00	0.00	0.00	0.00	0.00	0.00	0.00	0.00	0.00	0.00		3.00						COPD	0.00	0.00	0.00	0.00	0.00	0.00	1.00	0.00	1.00	0.00	0.00	0.00	0.00	0.00	0.00	0.00
0.00	breathless	1.00	0.00	0.00	1.00	0.00	1.00	0.00	0.00	0.00	0.00	0.00	0.00	0.00	0.00	0.00	0.00	0.00	0.00	0.00	0.00	0.00	0.00	0.00	0.00	1.00	0.00	0.00	0.00	0.00		3.00							0.00	0.00	0.00	0.00	0.00	0.00	0.00	0.00	0.00	0.00	0.00	0.00	0.00	0.00	0.00	0.00
0.00	fatigue, SC	1.00	0.00	1.00	1.00	1.00	1.00	0.00	0.00	0.00	0.00	0.00	0.00	0.00	0.00	0.00	0.00	1.00	0.00	0.00	0.00	0.00	0.00	0.00	0.00	1.00	0.00	0.00	0.00	0.00		3.00							0.00	0.00	0.00	0.00	0.00	0.00	0.00	0.00	0.00	0.00	0.00	0.00	0.00	0.00	0.00	0.00
3.00	lethagy, fe	6.00	0.00	1.00	1.00	1.00	0.00	1.00	0.00	0.00	0.00	0.00	0.00	1.00	1.00	0.00	0.00	0.00	0.00	0.00	0.00	1.00	0.00	0.00	0.00	0.00	1.00	0.00	0.00	0.00		3.00			asthma			hypothyro	0.00	0.00	0.00	0.00	0.00	0.00	1.00	1.00	0.00	0.00	0.00	1.00	1.00	0.00	0.00	0.00
0.00	fatigue, m	1.00	0.00	1.00	0.00	0.00	0.00	1.00	1.00	0.00	0.00	0.00	0.00	0.00	0.00	0.00	0.00	1.00	0.00	0.00	0.00	0.00	0.00	0.00	0.00	1.00	0.00	0.00	0.00	0.00		3.00							0.00	0.00	0.00	0.00	0.00	0.00	0.00	0.00	0.00	0.00	0.00	0.00	0.00	0.00	0.00	0.00
2.00	fever, eara	1.00	0.00	1.00	0.00	1.00	1.00	0.00	1.00	1.00	0.00	0.00	1.00	0.00	1.00	0.00	0.00	0.00	0.00	0.00	0.00	0.00	1.00	0.00	1.00	1.00	0.00	0.00	0.00	0.00		3.00			asthma - well controlled				0.00	0.00	0.00	0.00	0.00	0.00	1.00	1.00	0.00	0.00	0.00	0.00	0.00	0.00	0.00	0.00
0.00		1.00	0.00	0.00	0.00	0.00	0.00	0.00	0.00	0.00	0.00	0.00	0.00	0.00	0.00	0.00	0.00	0.00	0.00	0.00	0.00	0.00	0.00	0.00	0.00	1.00	0.00	0.00	0.00	0.00		3.00					depressior	previous c	0.00	1.00	0.00	0.00	0.00	0.00	0.00	0.00	0.00	0.00	0.00	0.00	0.00	1.00	0.00	0.00
0.00	SOB, chest	1.00	0.00	1.00	0.00	0.00	1.00	1.00	0.00	1.00	0.00	0.00	0.00	0.00	0.00	0.00	1.00	1.00	0.00	0.00	0.00	0.00	0.00	0.00	0.00	1.00	0.00	0.00	0.00	0.00		3.00						psoriasis	0.00	0.00	0.00	0.00	0.00	0.00	0.00	0.00	0.00	0.00	0.00	0.00	0.00	0.00	1.00	0.00
0.00	worsening	1.00	0.00	1.00	1.00	0.00	1.00	0.00	0.00	0.00	0.00	1.00	1.00	0.00	1.00	0.00	0.00	1.00	0.00	0.00	0.00	0.00	1.00	0.00	0.00	1.00	0.00	0.00	0.00	0.00	fatigue, str	2.00							0.00	0.00	0.00	0.00	0.00	0.00	0.00	0.00	0.00	0.00	0.00	0.00	0.00	0.00	0.00	0.00
0.00	SOB and fa	1.00	0.00	0.00	0.00	0.00	1.00	1.00	0.00	0.00	0.00	0.00	0.00	0.00	0.00	0.00	0.00	0.00	0.00	0.00	0.00	0.00	0.00	0.00	0.00	1.00	0.00	0.00	0.00	0.00		3.00					anxiety and depressio		1.00	1.00	0.00	0.00	0.00	0.00	0.00	0.00	0.00	0.00	0.00	0.00	0.00	0.00	0.00	0.00
0.00	severe SOI	1.00	0.00	1.00	1.00	1.00	1.00	1.00	1.00	1.00	0.00	0.00	0.00	0.00	0.00	0.00	0.00	1.00	1.00	0.00	0.00	1.00	0.00	0.00	0.00	1.00	1.00	0.00	0.00	1.00		3.00			asthma				0.00	0.00	0.00	0.00	0.00	0.00	1.00	1.00	0.00	0.00	0.00	0.00	0.00	0.00	0.00	0.00
3.00	cough, fev	10.00	0.00	1.00	1.00	0.00	1.00	0.00	0.00	0.00	0.00	0.00	0.00	0.00	0.00	0.00	0.00	0.00	0.00	0.00	0.00	0.00	0.00	0.00	0.00	0.00	1.00	0.00	0.00	1.00	fatigue	2.00	previously high BMI but lost 30kg through covid. AKI						0.00	0.00	0.00	0.00	0.00	0.00	0.00	0.00	0.00	0.00	0.00	0.00	0.00	0.00	0.00	0.00
3.00	cough, fati	1.00	0.00	1.00	1.00	0.00	1.00	1.00	0.00	0.00	0.00	0.00	0.00	0.00	0.00	0.00	0.00	0.00	0.00	0.00	0.00	0.00	0.00	0.00	0.00	1.00	0.00	0.00	0.00	0.00		3.00		yes		type 2		alcohol ab	0.00	0.00	0.00	0.00	1.00	0.00	1.00	0.00	1.00	1.00	1.00	0.00	0.00	0.00	0.00	0.00
0.00	SOB, cone	1.00	0.00	0.00	0.00	0.00	1.00	0.00	0.00	0.00	0.00	0.00	0.00	0.00	0.00	0.00	0.00	0.00	1.00	0.00	0.00	0.00	0.00	0.00	0.00	0.00	1.00	0.00	0.00	1.00		3.00							0.00	0.00	0.00	0.00	0.00	0.00	0.00	0.00	0.00	0.00	0.00	0.00	0.00	0.00	0.00	0.00
3.00	covid pneu	6.00	0.00	0.00	1.00	0.00	1.00	0.00	0.00	0.00	0.00	0.00	0.00	0.00	0.00	0.00	0.00	0.00	0.00	0.00	0.00	0.00	0.00	0.00	0.00	1.00	1.00	0.00	0.00	0.00		3.00							0.00	0.00	0.00	0.00	0.00	0.00	0.00	0.00	0.00	0.00	0.00	0.00	0.00	0.00	0.00	0.00
0.00	cough, sob	1.00	0.00	1.00	1.00	0.00	1.00	1.00	0.00	0.00	0.00	0.00	0.00	0.00	0.00	0.00	0.00	0.00	0.00	0.00	0.00	0.00	0.00	0.00	0.00	1.00	0.00	0.00	0.00	1.00	ongoing c	2.00					anxiety and depressio		1.00	1.00	0.00	0.00	0.00	0.00	0.00	0.00	0.00	0.00	0.00	0.00	0.00	0.00	0.00	0.00
0.00	tight chest	7.00	0.00	1.00	0.00	0.00	1.00	1.00	1.00	0.00	0.00	0.00	0.00	0.00	0.00	0.00	0.00	1.00	0.00	0.00	0.00	0.00	0.00	0.00	0.00	0.00	0.00	0.00	0.00	1.00		3.00			mild asthma as child				0.00	0.00	0.00	0.00	0.00	0.00	1.00	1.00	0.00	0.00	0.00	0.00	0.00	0.00	0.00	0.00
0.00	SOBOE, fat	1.00	0.00	1.00	1.00	0.00	1.00	1.00	0.00	0.00	0.00	0.00	0.00	0.00	0.00	0.00	0.00	0.00	0.00	0.00	0.00	0.00	0.00	1.00	0.00	1.00	0.00	0.00	0.00	0.00		3.00							0.00	0.00	0.00	0.00	0.00	0.00	0.00	0.00	0.00	0.00	0.00	0.00	0.00	0.00	0.00	0.00
3.00	tiredness,	1.00	1.00	1.00	0.00	0.00	0.00	1.00	0.00	0.00	1.00	0.00	0.00	0.00	0.00	0.00	0.00	0.00	0.00	0.00	0.00	0.00	0.00	0.00	0.00	1.00	1.00	0.00	0.00	0.00	SOB and fa	2.00							0.00	0.00	0.00	0.00	0.00	0.00	0.00	0.00	0.00	0.00	0.00	0.00	0.00	0.00	0.00	0.00
0.00	fever, coug	1.00	0.00	1.00	1.00	0.00	1.00	0.00	0.00	0.00	0.00	0.00	0.00	0.00	0.00	0.00	0.00	0.00	1.00	0.00	0.00	0.00	0.00	0.00	0.00	1.00	0.00	0.00	0.00	0.00		3.00						high BMI,	0.00	0.00	0.00	0.00	0.00	0.00	0.00	0.00	0.00	0.00	0.00	0.00	0.00	0.00	0.00	1.00
2.00	fatigue, fe	1.00	1.00	1.00	0.00	1.00	0.00	1.00	0.00	0.00	0.00	0.00	0.00	1.00	1.00	0.00	1.00	0.00	0.00	0.00	0.00	0.00	0.00	0.00	0.00	1.00	1.00	0.00	0.00	0.00	SOB and fa	2.00						HTN, GORI	0.00	0.00	0.00	0.00	0.00	0.00	0.00	0.00	0.00	0.00	1.00	0.00	0.00	0.00	0.00	0.00
0.00	cough, sob	1.00	0.00	1.00	1.00	0.00	1.00	1.00	0.00	0.00	0.00	0.00	0.00	0.00	0.00	0.00	0.00	0.00	0.00	0.00	0.00	0.00	0.00	1.00	0.00	0.00	1.00	0.00	0.00	1.00	mental he	2.00						back and h	0.00	0.00	0.00	0.00	0.00	0.00	1.00	1.00	0.00	0.00	1.00	1.00	0.00	0.00	0.00	0.00
1.00	deep achir	6.00	1.00	1.00	1.00	1.00	0.00	1.00	1.00	1.00	0.00	0.00	1.00	0.00	1.00	0.00	0.00	0.00	0.00	0.00	0.00	0.00	0.00	0.00	0.00	0.00	1.00	0.00	1.00	1.00	mouth ulc	2.00					depressior	oa	0.00	1.00	0.00	0.00	0.00	0.00	0.00	0.00	0.00	0.00	0.00	0.00	0.00	0.00	0.00	0.00
3.00	high fever	6.00	0.00	1.00	0.00	0.00	1.00	1.00	0.00	1.00	0.00	0.00	0.00	0.00	0.00	0.00	0.00	0.00	0.00	0.00	0.00	0.00	0.00	0.00	0.00	0.00	1.00	0.00	0.00	0.00		3.00						asthma.	0.00	0.00	0.00	0.00	0.00	0.00	0.00	1.00	0.00	0.00	0.00	0.00	0.00	0.00	0.00	0.00
0.00	sob, fatigu	1.00	0.00	0.00	0.00	0.00	1.00	1.00	0.00	0.00	0.00	0.00	0.00	0.00	0.00	0.00	0.00	1.00	0.00	0.00	0.00	0.00	0.00	0.00	0.00	1.00	0.00	0.00	0.00	0.00		3.00							0.00	0.00	0.00	0.00	0.00	0.00	0.00	0.00	0.00	0.00	0.00	0.00	0.00	0.00	0.00	0.00
0.00	cough, sob	1.00	0.00	0.00	1.00	0.00	1.00	0.00	0.00	0.00	0.00	0.00	0.00	0.00	0.00	0.00	0.00	0.00	0.00	0.00	0.00	0.00	0.00	0.00	0.00	1.00	0.00	0.00	0.00	1.00	sob while	2.00							0.00	0.00	0.00	0.00	0.00	0.00	0.00	0.00	0.00	0.00	0.00	0.00	0.00	0.00	0.00	0.00
0.00	generally u	6.00	1.00	1.00	1.00	1.00	1.00	1.00	1.00	0.00	0.00	0.00	1.00	0.00	0.00	0.00	0.00	0.00	0.00	1.00	0.00	0.00	0.00	1.00	0.00	0.00	1.00	0.00	0.00	0.00		3.00							0.00	0.00	0.00	0.00	0.00	0.00	0.00	0.00	0.00	0.00	0.00	0.00	0.00	0.00	0.00	0.00
1.00	sob, severe	8.00	0.00	1.00	0.00	0.00	1.00	1.00	0.00	1.00	0.00	0.00	0.00	0.00	0.00	0.00	0.00	0.00	0.00	0.00	1.00	0.00	0.00	0.00	0.00	1.00	1.00	0.00	0.00	1.00	still in serv	1.00					depressior	ulcerative	0.00	1.00	0.00	0.00	0.00	0.00	0.00	0.00	0.00	0.00	0.00	0.00	0.00	0.00	0.00	0.00
3.00	loss of tast	6.00	0.00	0.00	0.00	1.00	0.00	1.00	0.00	1.00	0.00	0.00	0.00	0.00	0.00	0.00	0.00	1.00	0.00	0.00	0.00	0.00	0.00	0.00	0.00	0.00	1.00	0.00	0.00	0.00		3.00					anxiety	fatigue and	1.00	0.00	1.00	0.00	0.00	0.00	0.00	0.00	0.00	0.00	0.00	0.00	0.00	0.00	0.00	0.00
0.00	fatigue, ch	6.00	0.00	1.00	1.00	1.00	1.00	1.00	1.00	0.00	0.00	0.00	0.00	0.00	0.00	1.00	0.00	1.00	1.00	0.00	0.00	0.00	0.00	0.00	0.00	0.00	1.00	0.00	0.00	0.00	still in serv	1.00		new onset hypertension					0.00	0.00	0.00	0.00	0.00	0.00	0.00	0.00	0.00	0.00	1.00	0.00	0.00	0.00	0.00	0.00
1.00	cough, los	1.00	0.00	0.00	1.00	1.00	1.00	1.00	1.00	1.00	1.00	0.00	0.00	0.00	0.00	0.00	0.00	0.00	0.00	0.00	0.00	0.00	0.00	0.00	0.00	1.00	0.00	0.00	1.00	1.00	voice and	2.00						overweigh	0.00	0.00	0.00	0.00	0.00	0.00	1.00	0.00	0.00	0.00	0.00	1.00	0.00	0.00	0.00	0.00
1.00	severe hac	6.00	0.00	0.00	1.00	0.00	0.00	0.00	0.00	0.00	0.00	1.00	0.00	0.00	0.00	0.00	0.00	0.00	0.00	0.00	0.00	0.00	0.00	0.00	0.00	0.00	1.00	0.00	0.00	0.00		3.00					anxiety	back op., a	1.00	0.00	0.00	0.00	0.00	0.00	0.00	0.00	0.00	0.00	0.00	0.00	0.00	0.00	0.00	0.00
2.00	fever, sob,	6.00	0.00	1.00	0.00	0.00	1.00	1.00	1.00	0.00	0.00	0.00	0.00	0.00	0.00	0.00	0.00	1.00	0.00	0.00	0.00	0.00	0.00	0.00	0.00	0.00	1.00	0.00	0.00	0.00	low mood	2.00			asthma			hypothyro	0.00	0.00	0.00	0.00	0.00	0.00	1.00	1.00	0.00	0.00	0.00	0.00	1.00	0.00	0.00	1.00
2.00	continuou	1.00	0.00	1.00	1.00	0.00	0.00	0.00	0.00	1.00	0.00	0.00	1.00	0.00	0.00	1.00	0.00	1.00	0.00	1.00	1.00	0.00	0.00	0.00	0.00	1.00	1.00	0.00	0.00	1.00	still in serv	1.00						new onset	0.00	0.00	0.00	0.00	0.00	0.00	0.00	0.00	0.00	0.00	1.00	0.00	0.00	0.00	0.00	0.00
1.00	sore throa	1.00	0.00	1.00	1.00	1.00	1.00	0.00	0.00	1.00	1.00	0.00	0.00	0.00	0.00	0.00	0.00	0.00	1.00	0.00	0.00	0.00	0.00	0.00	0.00	1.00	1.00	0.00	0.00	0.00	still active	1.00					depressior	suspected	0.00	1.00	0.00	0.00	0.00	0.00	0.00	0.00	0.00	0.00	0.00	0.00	0.00	0.00	0.00	0.00
1.00	chills and	2.00	1.00	1.00	1.00	0.00	1.00	1.00	1.00	1.00	0.00	0.00	1.00	1.00	0.00	0.00	0.00	1.00	0.00	0.00	1.00	0.00	0.00	1.00	0.00	1.00	1.00	0.00	0.00	0.00	heart - tac	2.00					depressior	cfs as teen	0.00	1.00	0.00	0.00	0.00	0.00	0.00	0.00	0.00	0.00	0.00	0.00	0.00	0.00	0.00	0.00
0.00	not record	2.00	0.00	0.00	0.00	0.00	0.00	0.00	0.00	0.00	0.00	0.00	0.00	0.00	0.00	0.00	0.00	0.00	1.00	0.00	0.00	0.00	0.00	0.00	0.00	0.00	0.00	0.00	0.00	1.00	not record	3.00						sciatica, m	0.00	0.00	0.00	0.00	0.00	0.00	0.00	0.00	0.00	0.00	0.00	0.00	0.00	0.00	0.00	0.00
0.00	tiredness,	2.00	1.00	1.00	1.00	0.00	0.00	0.00	0.00	0.00	0.00	0.00	0.00	0.00	0.00	0.00	0.00	0.00	0.00	0.00	0.00	0.00	0.00	0.00	0.00	1.00	1.00	0.00	0.00	0.00	not record	3.00						none reco	0.00	0.00	0.00	0.00	0.00	0.00	0.00	0.00	0.00	0.00	0.00	0.00	0.00	0.00	0.00	0.00
0.00	not record	2.00	0.00	0.00	0.00	0.00	0.00	0.00	0.00	0.00	0.00	0.00	0.00	0.00	0.00	0.00	0.00	0.00	0.00	0.00	0.00	0.00	0.00	0.00	0.00	0.00	0.00	0.00	0.00	0.00	recovering	3.00						none reco	0.00	0.00	0.00	0.00	0.00	0.00	0.00	0.00	0.00	0.00	0.00	0.00	0.00	0.00	0.00	0.00
0.00	stomach p	2.00	1.00	0.00	0.00	0.00	1.00	1.00	0.00	0.00	0.00	0.00	0.00	0.00	0.00	0.00	1.00	0.00	0.00	0.00	0.00	0.00	0.00	0.00	0.00	1.00	0.00	0.00	1.00	1.00	"constant	2.00	cardiac history						0.00	0.00	0.00	0.00	0.00	0.00	0.00	0.00	0.00	0.00	0.00	1.00	0.00	0.00	0.00	0.00
0.00	fever, coug	2.00	0.00	1.00	1.00	0.00	1.00	0.00	0.00	0.00	0.00	0.00	0.00	0.00	0.00	0.00	0.00	0.00	0.00	0.00	0.00	0.00	0.00	0.00	0.00	0.00	0.00	0.00	0.00	0.00		3.00							0.00	0.00	0.00	0.00	0.00	0.00	0.00	0.00	0.00	0.00	0.00	0.00	0.00	0.00	0.00	0.00
1.00	hot and cc	2.00	1.00	1.00	1.00	0.00	1.00	0.00	0.00	0.00	1.00	0.00	0.00	0.00	0.00	0.00	0.00	1.00	0.00	0.00	0.00	0.00	0.00	0.00	0.00	1.00	0.00	1.00	0.00	0.00	slight coug	2.00						gatsritis nc	0.00	0.00	0.00	0.00	0.00	0.00	0.00	0.00	0.00	0.00	0.00	0.00	0.00	0.00	0.00	0.00
0.00	sob, fatigu	1.00	1.00	0.00	0.00	0.00	1.00	1.00	0.00	0.00	0.00	0.00	0.00	0.00	0.00	0.00	0.00	0.00	0.00	0.00	0.00	0.00	0.00	0.00	0.00	1.00	1.00	0.00	0.00	0.00	still in serv	1.00							0.00	0.00	0.00	0.00	0.00	0.00	0.00	0.00	0.00	0.00	0.00	0.00	0.00	0.00	0.00	0.00
0.00	sob	1.00	0.00	0.00	0.00	0.00	1.00	0.00	0.00	0.00	0.00	0.00	0.00	0.00	0.00	0.00	0.00	0.00	0.00	0.00	0.00	0.00	0.00	0.00	0.00	1.00	0.00	0.00	0.00	1.00	still in serv	1.00					ptsd in pa	spinna bifi	0.00	0.00	0.00	1.00	0.00	0.00	0.00	0.00	0.00	0.00	0.00	0.00	0.00	0.00	0.00	0.00
3.00	cold and fe	1.00	0.00	1.00	0.00	0.00	1.00	0.00	0.00	1.00	0.00	0.00	0.00	0.00	0.00	0.00	0.00	0.00	0.00	0.00	1.00	0.00	0.00	0.00	0.00	1.00	0.00	0.00	0.00	1.00	headaches	2.00			asthma as child			DEPO injec	0.00	0.00	0.00	0.00	0.00	0.00	1.00	1.00	0.00	0.00	0.00	0.00	0.00	0.00	0.00	1.00
3.00	sob, fever	6.00	0.00	1.00	0.00	0.00	1.00	0.00	0.00	0.00	0.00	0.00	0.00	0.00	0.00	0.00	0.00	0.00	0.00	0.00	0.00	0.00	0.00	0.00	0.00	0.00	1.00	0.00	0.00	0.00		3.00						hypertensi	0.00	0.00	0.00	0.00	0.00	0.00	0.00	0.00	0.00	1.00	1.00	1.00	0.00	0.00	0.00	0.00
0.00	cough, col	1.00	0.00	1.00	1.00	0.00	0.00	1.00	0.00	0.00	0.00	0.00	1.00	0.00	1.00	0.00	0.00	0.00	0.00	0.00	0.00	0.00	0.00	0.00	0.00	1.00	0.00	0.00	0.00	0.00		3.00						recurrent	1.00	1.00	0.00	1.00	1.00	1.00	1.00	1.00	0.00	0.00	0.00	0.00	0.00	0.00	0.00	0.00
0.00	not report	5.00	0.00	0.00	0.00	0.00	0.00	0.00	0.00	0.00	0.00	0.00	0.00	0.00	0.00	0.00	0.00	0.00	0.00	0.00	0.00	0.00	0.00	0.00	0.00	0.00	0.00	0.00	0.00	0.00		3.00						postural o	0.00	0.00	0.00	0.00	0.00	0.00	0.00	0.00	0.00	0.00	1.00	0.00	0.00	0.00	0.00	0.00
0.00	fever, fatig	1.00	0.00	1.00	0.00	0.00	0.00	1.00	0.00	1.00	0.00	0.00	1.00	0.00	0.00	0.00	0.00	0.00	0.00	0.00	0.00	0.00	0.00	0.00	0.00	1.00	0.00	0.00	0.00	0.00		3.00				type 2 diabetes		high chole	0.00	0.00	0.00	0.00	0.00	0.00	0.00	0.00	0.00	1.00	0.00	1.00	0.00	0.00	0.00	0.00
3.00	not record	5.00	0.00	0.00	0.00	0.00	0.00	0.00	0.00	0.00	0.00	0.00	0.00	0.00	0.00	0.00	0.00	0.00	0.00	0.00	0.00	0.00	0.00	0.00	0.00	0.00	1.00	0.00	0.00	0.00	still in serv	1.00						none repo	0.00	0.00	0.00	0.00	0.00	0.00	0.00	0.00	0.00	0.00	0.00	0.00	0.00	0.00	0.00	0.00
2.00	fever, coug	1.00	0.00	1.00	1.00	0.00	1.00	0.00	0.00	0.00	0.00	0.00	0.00	0.00	0.00	0.00	0.00	0.00	0.00	0.00	0.00	0.00	0.00	0.00	0.00	1.00	0.00	0.00	0.00	0.00	none	3.00					depression		0.00	1.00	0.00	0.00	0.00	0.00	0.00	0.00	0.00	0.00	0.00	0.00	0.00	0.00	0.00	0.00
2.00	fever, coug	1.00	0.00	1.00	1.00	0.00	1.00	1.00	0.00	0.00	0.00	0.00	0.00	0.00	0.00	0.00	0.00	0.00	0.00	0.00	0.00	0.00	0.00	0.00	0.00	1.00	0.00	0.00	0.00	0.00	discharged	4.00							0.00	0.00	0.00	0.00	0.00	0.00	0.00	0.00	0.00	0.00	0.00	0.00	0.00	0.00	0.00	0.00
0.00	cough, SOI	1.00	0.00	0.00	1.00	0.00	1.00	0.00	0.00	0.00	0.00	0.00	0.00	0.00	0.00	0.00	0.00	0.00	0.00	0.00	0.00	0.00	0.00	0.00	0.00	1.00	0.00	0.00	0.00	1.00	still in serv	1.00						osteoarthr	0.00	0.00	0.00	0.00	0.00	0.00	1.00	1.00	0.00	0.00	1.00	0.00	0.00	0.00	0.00	0.00
1.00	achy body	1.00	0.00	1.00	1.00	0.00	1.00	0.00	1.00	0.00	0.00	0.00	0.00	1.00	0.00	0.00	0.00	1.00	0.00	0.00	1.00	0.00	0.00	0.00	0.00	1.00	1.00	0.00	0.00	0.00	still in serv	1.00						migraines	0.00	1.00	0.00	0.00	0.00	0.00	0.00	0.00	0.00	0.00	0.00	0.00	0.00	0.00	0.00	1.00
3.00	anosmia	6.00	0.00	0.00	0.00	1.00	0.00	0.00	0.00	0.00	0.00	0.00	0.00	0.00	0.00	0.00	0.00	0.00	0.00	0.00	0.00	0.00	0.00	0.00	0.00	0.00	1.00	0.00	0.00	0.00	still in serv	1.00						back pain,	0.00	0.00	0.00	0.00	0.00	0.00	0.00	0.00	0.00	0.00	0.00	0.00	0.00	0.00	0.00	0.00
0.00		3.00	0.00	0.00	0.00	0.00	0.00	0.00	0.00	0.00	0.00	0.00	0.00	0.00	0.00	0.00	0.00	0.00	0.00	0.00	0.00	0.00	0.00	0.00	0.00	0.00	0.00	0.00	0.00	0.00		3.00							0.00	0.00	0.00	0.00	0.00	0.00	0.00	0.00	0.00	0.00	0.00	0.00	0.00	0.00	0.00	0.00
0.00	fever, coug	6.00	1.00	1.00	1.00	0.00	0.00	0.00	0.00	1.00	0.00	0.00	0.00	0.00	0.00	0.00	0.00	0.00	0.00	0.00	0.00	0.00	0.00	0.00	0.00	1.00	0.00	1.00	1.00	0.00	still in serv	1.00						psoriasis,	0.00	1.00	0.00	0.00	0.00	0.00	0.00	0.00	0.00	0.00	1.00	1.00	1.00	0.00	1.00	0.00
3.00	sob	6.00	0.00	0.00	0.00	0.00	1.00	0.00	0.00	0.00	0.00	0.00	0.00	0.00	0.00	0.00	0.00	0.00	0.00	0.00	0.00	0.00	0.00	0.00	0.00	0.00	1.00	0.00	0.00	0.00		3.00						rheumatic	0.00	0.00	0.00	0.00	0.00	0.00	0.00	0.00	0.00	0.00	0.00	1.00	0.00	1.00	1.00	0.00
1.00	dry cough,	6.00	1.00	0.00	1.00	1.00	1.00	0.00	0.00	1.00	0.00	0.00	1.00	0.00	0.00	0.00	0.00	1.00	1.00	0.00	0.00	0.00	0.00	0.00	0.00	1.00	1.00	0.00	0.00	0.00	all improv	3.00						infected sa	0.00	0.00	0.00	0.00	0.00	0.00	1.00	1.00	0.00	0.00	0.00	0.00	0.00	0.00	0.00	0.00
3.00	cough, fev	5.00	1.00	1.00	1.00	0.00	0.00	1.00	1.00	0.00	0.00	0.00	0.00	1.00	0.00	0.00	0.00	1.00	0.00	1.00	0.00	0.00	0.00	0.00	0.00	1.00	0.00	0.00	0.00	1.00	intermitte	2.00							0.00	0.00	0.00	0.00	0.00	0.00	0.00	0.00	0.00	0.00	0.00	0.00	0.00	0.00	0.00	0.00
0.00	fever, coug	1.00	0.00	1.00	1.00	0.00	1.00	0.00	0.00	0.00	0.00	0.00	0.00	0.00	0.00	0.00	0.00	0.00	0.00	0.00	0.00	0.00	0.00	0.00	0.00	1.00	1.00	0.00	0.00	0.00	still in serv	1.00						bronciecta	0.00	0.00	0.00	0.00	0.00	0.00	1.00	0.00	0.00	0.00	0.00	0.00	0.00	0.00	0.00	0.00
3.00		1.00	0.00	0.00	0.00	0.00	0.00	0.00	0.00	0.00	0.00	0.00	0.00	0.00	0.00	1.00	0.00	0.00	0.00	0.00	1.00	0.00	0.00	0.00	0.00	1.00	0.00	0.00	0.00	1.00	still in serv	1.00						pulmonary	0.00	0.00	0.00	0.00	0.00	0.00	0.00	0.00	0.00	0.00	1.00	0.00	0.00	0.00	0.00	0.00
3.00	tinnutis (r)	5.00	1.00	0.00	1.00	0.00	1.00	1.00	1.00	0.00	0.00	0.00	0.00	0.00	0.00	0.00	0.00	0.00	0.00	0.00	0.00	0.00	0.00	0.00	0.00	0.00	1.00	0.00	0.00	0.00	fatigue.	2.00						angina, hy	0.00	0.00	0.00	0.00	0.00	0.00	0.00	0.00	0.00	0.00	1.00	1.00	0.00	0.00	0.00	0.00
0.00	cough, sob	1.00	0.00	0.00	0.00	1.00	0.00	1.00	0.00	0.00	0.00	0.00	0.00	0.00	0.00	0.00	0.00	0.00	0.00	0.00	1.00	0.00	0.00	0.00	0.00	1.00	0.00	0.00	0.00	0.00		3.00						IBS,	0.00	0.00	0.00	0.00	0.00	0.00	0.00	0.00	0.00	0.00	0.00	0.00	0.00	0.00	0.00	0.00
1.00	cough, bre	1.00	0.00	0.00	1.00	0.00	1.00	0.00	0.00	0.00	0.00	0.00	0.00	0.00	0.00	0.00	0.00	0.00	0.00	0.00	0.00	0.00	0.00	0.00	0.00	1.00	1.00	0.00	0.00	0.00	still under	1.00						HRT, scarri	1.00	0.00	1.00	1.00	0.00	0.00	1.00	1.00	0.00	0.00	0.00	1.00	0.00	0.00	1.00	0.00
3.00	not record	1.00	0.00	0.00	0.00	0.00	0.00	0.00	0.00	0.00	0.00	0.00	0.00	0.00	0.00	0.00	0.00	0.00	0.00	0.00	0.00	0.00	0.00	0.00	0.00	1.00	0.00	0.00	0.00	0.00	sob	2.00							0.00	0.00	0.00	0.00	0.00	0.00	0.00	0.00	0.00	0.00	0.00	0.00	0.00	0.00	0.00	0.00
3.00	fever, ano	1.00	1.00	1.00	0.00	1.00	1.00	0.00	1.00	0.00	0.00	0.00	0.00	0.00	0.00	0.00	0.00	0.00	0.00	0.00	1.00	0.00	0.00	0.00	0.00	1.00	0.00	0.00	0.00	0.00		3.00						sjogren's s	0.00	0.00	0.00	0.00	0.00	0.00	0.00	0.00	0.00	0.00	0.00	0.00	0.00	0.00	0.00	0.00
3.00	fever, ano	8.00	0.00	1.00	0.00	1.00	0.00	0.00	1.00	1.00	0.00	0.00	0.00	0.00	0.00	0.00	0.00	1.00	0.00	0.00	1.00	0.00	0.00	0.00	0.00	0.00	0.00	0.00	0.00	0.00		3.00							0.00	0.00	0.00	0.00	0.00	0.00	0.00	0.00	0.00	0.00	0.00	0.00	0.00	0.00	0.00	0.00
1.00	breathless	6.00	1.00	0.00	0.00	0.00	1.00	1.00	0.00	1.00	0.00	0.00	0.00	1.00	0.00	1.00	0.00	0.00	0.00	0.00	0.00	0.00	0.00	0.00	0.00	0.00	1.00	0.00	0.00	0.00		3.00						rhemattoi	0.00	0.00	0.00	0.00	0.00	0.00	1.00	0.00	1.00	0.00	0.00	0.00	0.00	0.00	1.00	0.00
3.00	no initial s	6.00	0.00	0.00	0.00	0.00	1.00	0.00	0.00	1.00	0.00	0.00	0.00	1.00	1.00	0.00	1.00	1.00	0.00	0.00	0.00	0.00	0.00	0.00	0.00	1.00	1.00	0.00	0.00	0.00	fatigue,	2.00						PCOS, ade	1.00	0.00	0.00	0.00	0.00	0.00	0.00	0.00	0.00	0.00	0.00	0.00	0.00	0.00	0.00	0.00
0.00	breathless	3.00	0.00	1.00	0.00	1.00	1.00	1.00	1.00	1.00	1.00	0.00	0.00	0.00	0.00	0.00	0.00	0.00	0.00	0.00	0.00	0.00	0.00	0.00	0.00	1.00	1.00	0.00	0.00	1.00		3.00						fibromyalg	0.00	0.00	0.00	0.00	0.00	0.00	0.00	0.00	0.00	0.00	0.00	0.00	0.00	0.00	1.00	0.00
3.00	headache,	8.00	0.00	0.00	0.00	0.00	0.00	0.00	0.00	1.00	1.00	1.00	0.00	0.00	0.00	0.00	0.00	0.00	0.00	0.00	1.00	0.00	0.00	0.00	0.00	0.00	1.00	1.00	0.00	0.00	fatigue - se	2.00						soliosis, H	0.00	0.00	0.00	0.00	0.00	0.00	0.00	0.00	0.00	0.00	1.00	1.00	0.00	0.00	0.00	0.00
0.00	fever, coug	3.00	0.00	1.00	1.00	1.00	1.00	0.00	0.00	0.00	0.00	0.00	0.00	0.00	0.00	0.00	0.00	0.00	0.00	0.00	1.00	0.00	0.00	0.00	0.00	1.00	0.00	0.00	0.00	0.00	still in serv	1.00							0.00	0.00	0.00	0.00	0.00	0.00	0.00	0.00	0.00	0.00	0.00	0.00	0.00	0.00	0.00	0.00
3.00	fatigue, so	1.00	1.00	1.00	0.00	0.00	1.00	1.00	0.00	0.00	0.00	0.00	0.00	0.00	0.00	0.00	0.00	0.00	1.00	0.00	0.00	1.00	0.00	0.00	0.00	1.00	0.00	0.00	0.00	0.00	pain when	2.00						severe cop	0.00	0.00	0.00	0.00	0.00	0.00	1.00	0.00	1.00	0.00	0.00	1.00	0.00	0.00	0.00	0.00
3.00	fever, coug	5.00	0.00	1.00	1.00	0.00	1.00	0.00	0.00	0.00	0.00	0.00	0.00	0.00	0.00	0.00	0.00	0.00	0.00	0.00	0.00	0.00	0.00	0.00	0.00	0.00	0.00	0.00	0.00	0.00		3.00						dyslexia, fr	0.00	0.00	0.00	0.00	0.00	1.00	0.00	0.00	0.00	0.00	0.00	0.00	0.00	0.00	0.00	0.00
0.00	sob, fatigu	5.00	0.00	0.00	0.00	0.00	1.00	1.00	0.00	0.00	0.00	0.00	0.00	0.00	0.00	0.00	0.00	0.00	0.00	0.00	0.00	0.00	0.00	0.00	0.00	0.00	0.00	0.00	0.00	0.00	discharged	3.00						pregnant a	0.00	0.00	0.00	0.00	0.00	0.00	0.00	0.00	0.00	0.00	0.00	0.00	0.00	0.00	0.00	0.00
2.00	cough, sob	6.00	0.00	0.00	1.00	0.00	1.00	0.00	0.00	0.00	0.00	0.00	0.00	0.00	0.00	0.00	0.00	0.00	0.00	0.00	0.00	0.00	0.00	0.00	0.00	1.00	1.00	0.00	0.00	0.00	sob and cc	2.00						obstructiv	0.00	0.00	0.00	0.00	0.00	0.00	0.00	0.00	0.00	0.00	0.00	0.00	0.00	0.00	0.00	0.00
3.00	balance iss	1.00	1.00	0.00	0.00	0.00	1.00	0.00	0.00	0.00	0.00	0.00	0.00	0.00	0.00	0.00	0.00	0.00	0.00	0.00	0.00	0.00	0.00	1.00	0.00	1.00	0.00	0.00	0.00	0.00	still in serv	1.00						menorrhag	0.00	0.00	0.00	0.00	0.00	0.00	0.00	0.00	0.00	1.00	1.00	1.00	1.00	0.00	0.00	0.00
3.00	small chok	1.00	1.00	0.00	1.00	0.00	1.00	1.00	1.00	1.00	0.00	1.00	0.00	0.00	0.00	0.00	0.00	0.00	0.00	0.00	0.00	0.00	0.00	0.00	0.00	1.00	0.00	0.00	1.00	0.00	cough, nas	2.00						overweigh	0.00	0.00	0.00	0.00	0.00	0.00	0.00	1.00	0.00	0.00	0.00	0.00	0.00	0.00	0.00	0.00
1.00	achy, fatig	1.00	1.00	0.00	0.00	1.00	1.00	1.00	1.00	0.00	1.00	0.00	0.00	0.00	0.00	0.00	0.00	1.00	0.00	0.00	0.00	0.00	0.00	0.00	0.00	1.00	0.00	0.00	0.00	1.00	sleep apnc	3.00						endometri	0.00	0.00	0.00	0.00	0.00	0.00	1.00	1.00	0.00	0.00	1.00	0.00	0.00	0.00	1.00	0.00
0.00		3.00	0.00	0.00	0.00	0.00	0.00	0.00	0.00	0.00	0.00	0.00	0.00	0.00	0.00	0.00	0.00	0.00	0.00	0.00	0.00	0.00	0.00	0.00	0.00	0.00	0.00	0.00	0.00	0.00		3.00							0.00	0.00	0.00	0.00	0.00	0.00	0.00	0.00	0.00	0.00	0.00	0.00	0.00	0.00	0.00	0.00
3.00	fever, chill	9.00	0.00	1.00	0.00	0.00	1.00	1.00	0.00	1.00	0.00	0.00	0.00	1.00	0.00	0.00	0.00	0.00	0.00	0.00	0.00	0.00	0.00	0.00	0.00	0.00	0.00	0.00	0.00	0.00	improved	3.00						type 1 DM	0.00	0.00	0.00	0.00	0.00	0.00	0.00	0.00	0.00	1.00	0.00	0.00	0.00	0.00	0.00	0.00
3.00	fever, hea	1.00	1.00	1.00	0.00	1.00	0.00	0.00	0.00	1.00	0.00	0.00	0.00	0.00	0.00	1.00	0.00	0.00	0.00	1.00	0.00	0.00	0.00	0.00	0.00	1.00	0.00	0.00	0.00	1.00	symptoms	2.00						asthma, ha	0.00	0.00	0.00	0.00	0.00	0.00	0.00	1.00	0.00	0.00	0.00	0.00	0.00	0.00	0.00	0.00
0.00	not report	5.00	0.00	0.00	0.00	0.00	0.00	0.00	0.00	0.00	0.00	0.00	0.00	0.00	0.00	0.00	0.00	0.00	0.00	0.00	0.00	0.00	0.00	0.00	0.00	0.00	1.00	0.00	0.00	0.00	still under	1.00						Polycystic	1.00	1.00	0.00	0.00	0.00	0.00	0.00	0.00	0.00	0.00	0.00	0.00	0.00	0.00	0.00	0.00
3.00	sob	3.00	0.00	0.00	0.00	0.00	1.00	0.00	0.00	0.00	0.00	0.00	0.00	0.00	0.00	0.00	0.00	0.00	0.00	0.00	0.00	0.00	0.00	0.00	0.00	1.00	0.00	0.00	0.00	1.00	still in serv	1.00						none repo	0.00	0.00	0.00	0.00	0.00	0.00	0.00	0.00	0.00	0.00	0.00	0.00	0.00	0.00	0.00	0.00
1.00	extreme fa	5.00	0.00	1.00	0.00	0.00	1.00	1.00	1.00	1.00	0.00	0.00	1.00	1.00	0.00	0.00	0.00	0.00	1.00	0.00	0.00	0.00	0.00	0.00	1.00	0.00	1.00	0.00	0.00	0.00	bowel irrit	2.00						chronic sir	0.00	0.00	0.00	0.00	0.00	0.00	1.00	0.00	1.00	1.00	1.00	1.00	1.00	0.00	0.00	0.00
3.00	contracted	3.00	0.00	0.00	0.00	0.00	0.00	0.00	0.00	0.00	0.00	0.00	0.00	0.00	0.00	0.00	0.00	0.00	0.00	0.00	0.00	0.00	0.00	0.00	0.00	0.00	0.00	0.00	0.00	1.00	swollen le	2.00						type 2 dial	0.00	0.00	0.00	0.00	0.00	0.00	0.00	0.00	0.00	0.00	0.00	0.00	0.00	0.00	0.00	0.00
3.00	breathless	5.00	1.00	0.00	0.00	0.00	1.00	1.00	0.00	1.00	0.00	0.00	0.00	0.00	0.00	0.00	0.00	0.00	0.00	0.00	0.00	0.00	0.00	0.00	1.00	0.00	0.00	0.00	0.00	0.00	gastric disc	2.00						hypothyro	0.00	0.00	0.00	0.00	0.00	0.00	0.00	0.00	0.00	1.00	0.00	0.00	1.00	0.00	0.00	0.00
1.00	fatigue, co	5.00	0.00	0.00	1.00	0.00	1.00	1.00	0.00	0.00	0.00	0.00	0.00	0.00	0.00	0.00	0.00	1.00	0.00	0.00	0.00	0.00	0.00	0.00	0.00	1.00	0.00	0.00	0.00	0.00	chest tight	2.00						depressior	0.00	1.00	0.00	0.00	0.00	0.00	0.00	0.00	0.00	0.00	0.00	0.00	0.00	0.00	0.00	0.00
2.00	fever, coug	1.00	0.00	1.00	1.00	0.00	1.00	0.00	0.00	0.00	0.00	0.00	0.00	0.00	0.00	0.00	0.00	0.00	0.00	0.00	0.00	0.00	0.00	0.00	0.00	1.00	0.00	0.00	0.00	0.00	sob,	2.00							0.00	0.00	0.00	0.00	0.00	0.00	0.00	0.00	0.00	0.00	0.00	0.00	0.00	0.00	0.00	0.00
1.00	cough, sob	5.00	0.00	1.00	1.00	0.00	1.00	1.00	1.00	1.00	0.00	0.00	0.00	0.00	0.00	0.00	0.00	1.00	0.00	0.00	1.00	0.00	0.00	0.00	0.00	1.00	1.00	0.00	0.00	1.00	still in serv	1.00						hypothyro	1.00	0.00	0.00	0.00	0.00	0.00	0.00	0.00	0.00	0.00	0.00	1.00	0.00	0.00	0.00	0.00
1.00	cough, pai	6.00	1.00	0.00	1.00	0.00	1.00	1.00	0.00	0.00	0.00	0.00	0.00	0.00	0.00	0.00	0.00	1.00	0.00	0.00	0.00	0.00	0.00	0.00	0.00	1.00	1.00	0.00	0.00	0.00	still in serv	1.00						hysterectc	0.00	0.00	0.00	0.00	0.00	0.00	0.00	0.00	0.00	0.00	0.00	0.00	0.00	0.00	0.00	0.00
3.00	not report	6.00	0.00	0.00	0.00	0.00	0.00	0.00	0.00	0.00	0.00	0.00	0.00	0.00	0.00	0.00	0.00	0.00	0.00	0.00	0.00	0.00	0.00	0.00	0.00	1.00	0.00	0.00	0.00	1.00	pain and b	2.00							0.00	0.00	0.00	0.00	0.00	0.00	0.00	0.00	0.00	0.00	0.00	0.00	0.00	0.00	0.00	0.00
0.00	sob, cough	1.00	0.00	0.00	1.00	0.00	1.00	0.00	0.00	0.00	0.00	0.00	0.00	0.00	0.00	0.00	0.00	1.00	0.00	0.00	0.00	0.00	0.00	0.00	0.00	1.00	0.00	0.00	0.00	0.00		3.00							0.00	0.00	0.00	0.00	0.00	0.00	0.00	0.00	0.00	0.00	0.00	0.00	0.00	0.00	0.00	0.00
0.00	sob, breat	1.00	0.00	0.00	0.00	0.00	1.00	1.00	0.00	0.00	0.00	0.00	0.00	0.00	0.00	0.00	0.00	0.00	0.00	0.00	0.00	0.00	0.00	0.00	0.00	1.00	1.00	0.00	0.00	0.00	still in serv	1.00							0.00	0.00	0.00	0.00	0.00	0.00	0.00	0.00	0.00	0.00	0.00	0.00	0.00	0.00	0.00	0.00
3.00	chills, hea	6.00	0.00	1.00	1.00	0.00	1.00	1.00	0.00	1.00	0.00	0.00	0.00	1.00	0.00	0.00	0.00	1.00	1.00	0.00	0.00	0.00	0.00	0.00	1.00	0.00	1.00	0.00	0.00	0.00	still in serv	1.00						hypertensi	0.00	0.00	0.00	0.00	0.00	0.00	0.00	0.00	0.00	0.00	1.00	1.00	0.00	0.00	0.00	0.00
3.00	fatigue, dr	5.00	1.00	0.00	0.00	0.00	0.00	1.00	0.00	1.00	0.00	0.00	0.00	1.00	0.00	0.00	0.00	0.00	0.00	0.00	0.00	0.00	0.00	1.00	0.00	0.00	0.00	1.00	0.00	0.00	still in serv	1.00						adhd	0.00	0.00	0.00	0.00	0.00	1.00	0.00	0.00	0.00	0.00	0.00	0.00	0.00	0.00	0.00	0.00
1.00	cough, sor	1.00	0.00	0.00	1.00	0.00	0.00	0.00	1.00	1.00	1.00	0.00	0.00	0.00	0.00	0.00	0.00	0.00	0.00	0.00	0.00	0.00	0.00	0.00	0.00	1.00	1.00	0.00	0.00	0.00	still in serv	1.00						hiatus her	0.00	0.00	0.00	1.00	0.00	0.00	1.00	1.00	0.00	0.00	0.00	0.00	0.00	0.00	0.00	0.00
0.00	fever, coug	5.00	0.00	1.00	1.00	0.00	0.00	0.00	0.00	0.00	0.00	0.00	0.00	0.00	0.00	0.00	0.00	0.00	0.00	0.00	0.00	0.00	0.00	0.00	0.00	0.00	0.00	0.00	0.00	0.00		3.00						suggested	1.00	1.00	0.00	1.00	0.00	1.00	0.00	0.00	0.00	0.00	0.00	1.00	0.00	0.00	0.00	0.00
3.00	headache,	5.00	0.00	1.00	1.00	1.00	1.00	1.00	0.00	1.00	0.00	0.00	0.00	1.00	0.00	0.00	0.00	0.00	0.00	0.00	0.00	0.00	0.00	0.00	0.00	1.00	1.00	0.00	0.00	0.00	still in serr	1.00						acute brea	1.00	0.00	0.00	1.00	0.00	0.00	0.00	0.00	0.00	0.00	0.00	1.00	0.00	1.00	0.00	0.00

sleep_con	high_BMI	mobility	chronic_pa	gastric_co	other_con	number_c	YRS19final	HCTfrailty	Malnutriti	HCTUCLHl	PHQ9	GAD7	Patientfun	EQ5Dmob	EQ5Dselfc	EQ5Dusua	EQ5Dpain	EQ5Danxietydepression
0.00	0.00	0.00	0.00	0.00	0.00	3.00	#NULL!	#NULL!	#NULL!	#NULL!	#NULL!	#NULL!	#NULL!	1	1	2	1	1
0.00	0.00	0.00	0.00	0.00	0.00	0.00	7	#NULL!	#NULL!	#NULL!	#NULL!	#NULL!	#NULL!	1	1	1	1	1
0.00	0.00	0.00	0.00	0.00	0.00	2.00	2	#NULL!	#NULL!	#NULL!	#NULL!	#NULL!	#NULL!	2	1	2	3	2
0.00	0.00	0.00	0.00	0.00	0.00	0.00	7	#NULL!	#NULL!	#NULL!	#NULL!	#NULL!	#NULL!	3	1	3	1	2
0.00	0.00	0.00	0.00	0.00	0.00	0.00	11	#NULL!	#NULL!	#NULL!	#NULL!	#NULL!	#NULL!	2	1	2	1	2
0.00	0.00	0.00	0.00	0.00	0.00	1.00	6	#NULL!	#NULL!	#NULL!	#NULL!	#NULL!	#NULL!	2	1	1	2	2
0.00	0.00	0.00	1.00	0.00	0.00	1.00	6	#NULL!	#NULL!	#NULL!	#NULL!	#NULL!	#NULL!	2	2	3	3	1
0.00	0.00	0.00	0.00	0.00	0.00	0.00	0	#NULL!	#NULL!	#NULL!	#NULL!	#NULL!	#NULL!	2	1	3	2	1
0.00	0.00	0.00	0.00	0.00	0.00	0.00	12	#NULL!	#NULL!	#NULL!	#NULL!	#NULL!	#NULL!	1	1	1	2	2
0.00	0.00	0.00	0.00	0.00	0.00	0.00	2	#NULL!	#NULL!	#NULL!	#NULL!	#NULL!	#NULL!	1	1	3	1	1
1.00	1.00	0.00	1.00	0.00	1.00	6.00	3	#NULL!	#NULL!	#NULL!	#NULL!	#NULL!	#NULL!	3	3	3	2	2
0.00	1.00	0.00	0.00	0.00	0.00	2.00	0	#NULL!	#NULL!	#NULL!	#NULL!	#NULL!	#NULL!	3	1	2	3	3
0.00	0.00	0.00	0.00	0.00	0.00	2.00	14	#NULL!	#NULL!	#NULL!	#NULL!	#NULL!	#NULL!	1	1	1	1	1
0.00	0.00	0.00	0.00	0.00	0.00	0.00	#NULL!	#NULL!	#NULL!	#NULL!	#NULL!	#NULL!	#NULL!	#NULL!	#NULL!	#NULL!	#NULL!	#NULL!
0.00	0.00	0.00	0.00	0.00	0.00	0.00	4	#NULL!	#NULL!	#NULL!	#NULL!	#NULL!	#NULL!	1	1	1	2	2
0.00	0.00	0.00	0.00	0.00	0.00	0.00	#NULL!	#NULL!	#NULL!	#NULL!	#NULL!	#NULL!	#NULL!	1	1	1	1	1
0.00	0.00	0.00	0.00	0.00	0.00	1.00	0	#NULL!	#NULL!	#NULL!	#NULL!	#NULL!	#NULL!	1	1	1	1	1
0.00	0.00	0.00	0.00	0.00	0.00	0.00	11	#NULL!	#NULL!	#NULL!	#NULL!	#NULL!	#NULL!	2	1	2	1	2
0.00	0.00	0.00	0.00	0.00	0.00	0.00	#NULL!	#NULL!	#NULL!	#NULL!	#NULL!	#NULL!	#NULL!	1	1	1	1	1
0.00	0.00	0.00	0.00	0.00	1.00	5.00	7	#NULL!	#NULL!	#NULL!	#NULL!	#NULL!	#NULL!	2	1	2	2	1
0.00	0.00	0.00	0.00	0.00	0.00	0.00	2	#NULL!	#NULL!	#NULL!	#NULL!	#NULL!	#NULL!	1	1	1	1	2
0.00	0.00	0.00	0.00	0.00	0.00	1.00	5	#NULL!	#NULL!	#NULL!	13	3	#NULL!	2	1	1	2	2
0.00	0.00	0.00	1.00	0.00	0.00	4.00	#NULL!	#NULL!	#NULL!	#NULL!	#NULL!	#NULL!	#NULL!	2	2	2	3	2
0.00	0.00	0.00	0.00	0.00	0.00	1.00	1	#NULL!	#NULL!	#NULL!	#NULL!	#NULL!	#NULL!	2	1	1	1	1
0.00	0.00	0.00	0.00	0.00	0.00	0.00	7	#NULL!	#NULL!	#NULL!	#NULL!	#NULL!	#NULL!	2	1	3	2	3
0.00	0.00	0.00	0.00	0.00	0.00	2.00	8	#NULL!	#NULL!	#NULL!	#NULL!	#NULL!	#NULL!	1	1	1	1	1
0.00	0.00	0.00	0.00	0.00	0.00	1.00	1	#NULL!	#NULL!	#NULL!	#NULL!	#NULL!	#NULL!	2	1	2	1	2
0.00	0.00	0.00	0.00	0.00	0.00	0.00	10	#NULL!	#NULL!	#NULL!	#NULL!	#NULL!	#NULL!	1	1	1	1	1
0.00	0.00	0.00	0.00	0.00	1.00	5.00	14	#NULL!	#NULL!	#NULL!	12	8	#NULL!	3	3	3	1	2
0.00	0.00	0.00	0.00	0.00	0.00	0.00	18	#NULL!	#NULL!	#NULL!	#NULL!	#NULL!	#NULL!	2	2	2	1	2
0.00	0.00	0.00	0.00	0.00	0.00	0.00	0	#NULL!	#NULL!	#NULL!	#NULL!	#NULL!	#NULL!	#NULL!	#NULL!	#NULL!	#NULL!	#NULL!
0.00	0.00	0.00	0.00	0.00	0.00	2.00	1	#NULL!	#NULL!	#NULL!	#NULL!	#NULL!	#NULL!	1	1	1	1	1
0.00	0.00	0.00	0.00	0.00	0.00	1.00	5	#NULL!	#NULL!	#NULL!	3	2	#NULL!	3	1	3	3	2
0.00	0.00	0.00	0.00	0.00	0.00	0.00	8	#NULL!	#NULL!	#NULL!	0	0	#NULL!	1	2	2	2	3
0.00	0.00	0.00	0.00	0.00	0.00	0.00	5	#NULL!	#NULL!	#NULL!	10	6	#NULL!	#NULL!	#NULL!	#NULL!	#NULL!	#NULL!
0.00	1.00	0.00	0.00	0.00	0.00	2.00	#NULL!	#NULL!	#NULL!	#NULL!	#NULL!	#NULL!	#NULL!	1	1	1	1	1
0.00	0.00	0.00	0.00	1.00	0.00	4.00	4	#NULL!	#NULL!	#NULL!	5	3	#NULL!	1	1	2	1	2
0.00	0.00	0.00	1.00	0.00	0.00	6.00	21	#NULL!	#NULL!	#NULL!	#NULL!	#NULL!	#NULL!	1	1	2	1	3
0.00	0.00	0.00	1.00	0.00	0.00	2.00	18	#NULL!	#NULL!	#NULL!	#NULL!	#NULL!	#NULL!	1	1	1	1	2
0.00	0.00	0.00	0.00	0.00	0.00	1.00	2	#NULL!	#NULL!	#NULL!	#NULL!	#NULL!	#NULL!	1	1	3	3	2
0.00	0.00	0.00	0.00	0.00	0.00	0.00	5	#NULL!	#NULL!	#NULL!	#NULL!	#NULL!	#NULL!	1	1	1	1	1
0.00	0.00	0.00	0.00	0.00	0.00	0.00	0	#NULL!	#NULL!	#NULL!	#NULL!	#NULL!	#NULL!	1	2	2	1	1
0.00	0.00	0.00	0.00	0.00	0.00	0.00	12	#NULL!	#NULL!	#NULL!	#NULL!	#NULL!	#NULL!	1	1	1	2	3
0.00	0.00	1.00	0.00	1.00	0.00	3.00	10	#NULL!	#NULL!	#NULL!	#NULL!	#NULL!	#NULL!	1	1	2	1	1
0.00	0.00	0.00	1.00	0.00	0.00	3.00	#NULL!	#NULL!	#NULL!	#NULL!	#NULL!	#NULL!	#NULL!	1	1	1	2	1
0.00	0.00	0.00	0.00	0.00	0.00	1.00	#NULL!	#NULL!	#NULL!	#NULL!	#NULL!	#NULL!	#NULL!	1	1	2	1	1
0.00	1.00	0.00	0.00	0.00	0.00	3.00	17	#NULL!	#NULL!	#NULL!	#NULL!	#NULL!	#NULL!	1	1	3	1	3
0.00	0.00	0.00	1.00	0.00	0.00	2.00	8	#NULL!	#NULL!	#NULL!	#NULL!	#NULL!	#NULL!	1	1	1	3	2
0.00	0.00	0.00	1.00	0.00	1.00	5.00	11	#NULL!	#NULL!	#NULL!	#NULL!	#NULL!	#NULL!	1	1	2	1	2
1.00	0.00	0.00	0.00	0.00	0.00	2.00	9	#NULL!	#NULL!	#NULL!	#NULL!	#NULL!	#NULL!	3	3	3	3	2
0.00	0.00	0.00	1.00	0.00	0.00	2.00	7	#NULL!	#NULL!	#NULL!	#NULL!	#NULL!	#NULL!	1	3	3	3	2
0.00	0.00	0.00	1.00	0.00	0.00	2.00	#NULL!	#NULL!	#NULL!	#NULL!	#NULL!	#NULL!	#NULL!	2	1	1	2	2
0.00	0.00	1.00	1.00	0.00	0.00	2.00	17	#NULL!	#NULL!	#NULL!	#NULL!	#NULL!	#NULL!	2	5	3	3	1
0.00	0.00	0.00	0.00	0.00	0.00	0.00	#NULL!	#NULL!	#NULL!	#NULL!	#NULL!	#NULL!	#NULL!	1	1	1	1	1
0.00	0.00	0.00	0.00	0.00	0.00	0.00	#NULL!	#NULL!	#NULL!	#NULL!	#NULL!	#NULL!	#NULL!	1	1	1	1	2
0.00	0.00	0.00	0.00	0.00	0.00	1.00	#NULL!	#NULL!	#NULL!	#NULL!	#NULL!	#NULL!	#NULL!	1	1	1	1	2
0.00	0.00	0.00	0.00	0.00	0.00	0.00	#NULL!	#NULL!	#NULL!	#NULL!	#NULL!	#NULL!	#NULL!	1	2	3	3	2
0.00	0.00	0.00	1.00	1.00	0.00	2.00	3	#NULL!	#NULL!	#NULL!	#NULL!	#NULL!	#NULL!	1	1	1	2	2
0.00	0.00	0.00	0.00	0.00	0.00	0.00	0	#NULL!	#NULL!	#NULL!	#NULL!	#NULL!	#NULL!	3	1	3	2	1
0.00	0.00	0.00	0.00	0.00	1.00	2.00	18	#NULL!	#NULL!	#NULL!	#NULL!	#NULL!	#NULL!	3	1	3	1	2
0.00	0.00	0.00	0.00	0.00	0.00	2.00	8	#NULL!	#NULL!	#NULL!	#NULL!	#NULL!	#NULL!	1	1	1	1	1
0.00	1.00	0.00	1.00	0.00	1.00	6.00	0	#NULL!	#NULL!	#NULL!	#NULL!	#NULL!	#NULL!	1	1	1	1	2
0.00	0.00	0.00	0.00	1.00	0.00	6.00	12	#NULL!	#NULL!	#NULL!	17	18	#NULL!	1	1	3	3	3
0.00	0.00	0.00	0.00	1.00	0.00	2.00	17	#NULL!	#NULL!	#NULL!	#NULL!	#NULL!	#NULL!	1	1	1	1	2
0.00	0.00	0.00	0.00	0.00	0.00	2.00	#NULL!	#NULL!	#NULL!	#NULL!	#NULL!	#NULL!	#NULL!	1	1	2	2	2
0.00	0.00	0.00	0.00	0.00	0.00	0.00	0	#NULL!	#NULL!	#NULL!	12	10	#NULL!	3	1	3	2	3
0.00	0.00	0.00	0.00	0.00	0.00	1.00	5	#NULL!	#NULL!	#NULL!	9	5	#NULL!	1	1	1	1	3
0.00	0.00	0.00	0.00	0.00	0.00	0.00	17	#NULL!	#NULL!	#NULL!	18	15	#NULL!	1	1	1	3	2
0.00	0.00	0.00	1.00	0.00	1.00	5.00	3	#NULL!	#NULL!	#NULL!	#NULL!	#NULL!	#NULL!	1	1	1	2	1
0.00	0.00	0.00	0.00	0.00	0.00	2.00	15	#NULL!	#NULL!	#NULL!	26	21	#NULL!	1	1	2	2	3
0.00	0.00	0.00	1.00	0.00	0.00	1.00	#NULL!	#NULL!	#NULL!	#NULL!	#NULL!	#NULL!	#NULL!	1	1	1	2	3
0.00	0.00	0.00	0.00	0.00	0.00	0.00	#NULL!	#NULL!	#NULL!	#NULL!	#NULL!	#NULL!	#NULL!	#NULL!	#NULL!	#NULL!	#NULL!	#NULL!
0.00	0.00	0.00	1.00	1.00	0.00	6.00	0	#NULL!	#NULL!	#NULL!	#NULL!	#NULL!	#NULL!	2	1	1	3	1
0.00	0.00	1.00	1.00	0.00	1.00	6.00	#NULL!	#NULL!	#NULL!	#NULL!	#NULL!	#NULL!	#NULL!	1	1	1	2	2
0.00	0.00	0.00	0.00	0.00	1.00	2.00	0	#NULL!	#NULL!	#NULL!	#NULL!	#NULL!	#NULL!	1	1	1	1	1
0.00	0.00	0.00	0.00	0.00	0.00	0.00	#NULL!	#NULL!	#NULL!	#NULL!	#NULL!	#NULL!	#NULL!	1	1	1	1	4
0.00	0.00	0.00	0.00	0.00	0.00	1.00	#NULL!	#NULL!	#NULL!	#NULL!	#NULL!	#NULL!	#NULL!	1	1	2	2	3
0.00	0.00	0.00	0.00	0.00	0.00	1.00	21	#NULL!	#NULL!	#NULL!	18	14	#NULL!	1	1	3	2	4
0.00	0.00	0.00	0.00	1.00	1.00	6.00	#NULL!	#NULL!	#NULL!	#NULL!	#NULL!	#NULL!	#NULL!	2	1	3	2	2
0.00	0.00	0.00	0.00	1.00	0.00	1.00	3	#NULL!	#NULL!	#NULL!	#NULL!	#NULL!	#NULL!	1	1	1	1	1
0.00	0.00	0.00	1.00	1.00	1.00	6.00	13	#NULL!	#NULL!	#NULL!	#NULL!	#NULL!	#NULL!	1	1	1	2	2
0.00	0.00	0.00	0.00	0.00	0.00	0.00	17	#NULL!	#NULL!	#NULL!	#NULL!	#NULL!	#NULL!	1	1	1	1	1
0.00	0.00	0.00	0.00	0.00	1.00	3.00	20	#NULL!	#NULL!	#NULL!	#NULL!	#NULL!	#NULL!	1	1	1	2	3
0.00	0.00	0.00	0.00	0.00	0.00	0.00	7	#NULL!	#NULL!	#NULL!	#NULL!	#NULL!	#NULL!	2	1	3	3	3
0.00	0.00	0.00	1.00	0.00	0.00	3.00	#NULL!	4	#NULL!	#NULL!	#NULL!	#NULL!	#NULL!	2	1	3	1	2
0.00	0.00	0.00	0.00	0.00	1.00	3.00	18	#NULL!	#NULL!	#NULL!	#NULL!	#NULL!	#NULL!	1	1	1	3	3
0.00	0.00	0.00	1.00	0.00	1.00	4.00	18	#NULL!	#NULL!	#NULL!	21	12	#NULL!	2	1	2	3	2
0.00	0.00	0.00	1.00	0.00	1.00	4.00	#NULL!	#NULL!	#NULL!	#NULL!	#NULL!	#NULL!	#NULL!	1	1	1	1	1
0.00	0.00	0.00	0.00	0.00	0.00	0.00	2	#NULL!	#NULL!	#NULL!	#NULL!	#NULL!	#NULL!	1	1	2	1	2
0.00	0.00	0.00	0.00	0.00	0.00	3.00	11	#NULL!	#NULL!	#NULL!	15	#NULL!	#NULL!	2	1	3	2	4
0.00	0.00	0.00	1.00	0.00	0.00	2.00	#NULL!	#NULL!	#NULL!	#NULL!	#NULL!	#NULL!	#NULL!	1	1	1	1	1
0.00	0.00	0.00	0.00	0.00	1.00	4.00	11	#NULL!	#NULL!	#NULL!	#NULL!	9	#NULL!	2	1	2	2	2
1.00	0.00	0.00	0.00	0.00	0.00	1.00	9	#NULL!	#NULL!	#NULL!	17	7	#NULL!	1	1	3	1	3
0.00	0.00	0.00	1.00	1.00	0.00	6.00	12	#NULL!	#NULL!	#NULL!	#NULL!	#NULL!	#NULL!	1	1	1	1	3
0.00	1.00	0.00	0.00	0.00	1.00	5.00	#NULL!	#NULL!	#NULL!	#NULL!	#NULL!	#NULL!	#NULL!	1	1	1	1	1
0.00	0.00	1.00	0.00	0.00	1.00	6.00	#NULL!	#NULL!	#NULL!	#NULL!	#NULL!	#NULL!	#NULL!	1	1	2	1	2
0.00	0.00	0.00	0.00	0.00	0.00	0.00	#NULL!	#NULL!	#NULL!	#NULL!	#NULL!	#NULL!	#NULL!	#NULL!	#NULL!	#NULL!	#NULL!	#NULL!
0.00	0.00	0.00	0.00	0.00	0.00	1.00	#NULL!	#NULL!	#NULL!	#NULL!	#NULL!	#NULL!	#NULL!	1	1	1	1	1
0.00	0.00	0.00	0.00	0.00	0.00	1.00	11	#NULL!	#NULL!	#NULL!	19	16	#NULL!	4	4	4	4	4
0.00	0.00	0.00	1.00	0.00	1.00	4.00	#NULL!	#NULL!	#NULL!	#NULL!	#NULL!	#NULL!	#NULL!	1	1	4	3	2
0.00	0.00	0.00	0.00	0.00	1.00	1.00	0	#NULL!	#NULL!	#NULL!	#NULL!	#NULL!	#NULL!	2	1	3	1	2
0.00	0.00	1.00	1.00	0.00	0.00	6.00	15	#NULL!	#NULL!	#NULL!	11	#NULL!	#NULL!	1	1	1	1	1
0.00	0.00	0.00	0.00	0.00	0.00	0.00	#NULL!	#NULL!	#NULL!	#NULL!	2	0	#NULL!	4	2	3	2	3
0.00	0.00	0.00	0.00	0.00	0.00	2.00	#NULL!	#NULL!	#NULL!	#NULL!	#NULL!	#NULL!	#NULL!	2	2	4	2	3
0.00	0.00	0.00	0.00	0.00	0.00	1.00	#NULL!	#NULL!	#NULL!	#NULL!	#NULL!	#NULL!	#NULL!	1	1	1	2	2
0.00	0.00	0.00	0.00	0.00	0.00	0.00	10	#NULL!	#NULL!	#NULL!	#NULL!	#NULL!	#NULL!	1	1	2	1	2
0.00	0.00	1.00	0.00	0.00	1.00	5.00	#NULL!	#NULL!	#NULL!	#NULL!	#NULL!	#NULL!	#NULL!	2	2	4	2	3
0.00	0.00	0.00	0.00	0.00	1.00	2.00	9	#NULL!	#NULL!	#NULL!	#NULL!	#NULL!	#NULL!	#NULL!	#NULL!	#NULL!	#NULL!	#NULL!
0.00	0.00	0.00	0.00	0.00	0.00	0.00	#NULL!	#NULL!	#NULL!	#NULL!	#NULL!	#NULL!	#NULL!	1	1	1	2	1
0.00	0.00	0.00	0.00	0.00	0.00	0.00	#NULL!	#NULL!	#NULL!	#NULL!	#NULL!	#NULL!	#NULL!	1	1	3	2	2
0.00	0.00	0.00	0.00	0.00	0.00	0.00	#NULL!	#NULL!	#NULL!	#NULL!	#NULL!	#NULL!	#NULL!	1	1	1	1	1
0.00	0.00	0.00	0.00	0.00	1.00	3.00	#NULL!	#NULL!	#NULL!	#NULL!	#NULL!	#NULL!	#NULL!	1	2	3	2	2
0.00	0.00	0.00	0.00	0.00	0.00	1.00	#NULL!	#NULL!	#NULL!	#NULL!	#NULL!	#NULL!	#NULL!	2	2	4	2	2
0.00	0.00	0.00	0.00	1.00	1.00	4.00	0	#NULL!	#NULL!	#NULL!	23	20	#NULL!	3	1	3	3	3
0.00	0.00	0.00	0.00	0.00	0.00	5.00	0	#NULL!	#NULL!	#NULL!	#NULL!	#NULL!	#NULL!	4	4	5	3	3
0.00	1.00	0.00	0.00	0.00	0.00	5.00	#NULL!	#NULL!	#NULL!	#NULL!	#NULL!	#NULL!	#NULL!	3	2	4	3	2
