# Supplementary material for: A mixed-methods analysis of the implementation of a new community long-COVID service during the 2020 pandemic: Learning from practice
Source: PLoS One. 2026 Jun 26;21(6):e0313367. doi: 10.1371/journal.pone.0313367 (PMC13308792; doi:10.1371/journal.pone.0313367)
Supplement: S7 File — (PDF) [file pone.0313367.s007.pdf]

Transcript of patients' interviews.

P003

0:0:0.0 --> 0:0:1.600
PW (LC CLINIC)
OK, good. I think it's working.

0:0:2.790 --> 0:0:5.960
PW (LC CLINIC)
Yes. Does that does that sort of like give you a notification at your end?

0:0:5.510 --> 0:0:7.0
P003
Yeah. Yeah, I can see that.

0:0:6.660 --> 0:0:15.420
PW(LC CLINIC)
Yeah, OK. Brilliant. OK, allocate just for the purpose of the recording. Just wanted to make sure that you're happy for me to be recording this conversation today.

0:0:15.780 --> 0:0:18.330
P003
Yeah, absolutely fine to be recorded, no problems.

0:0:18.760 --> 0:0:35.270
PW(LC CLINIC)
OK. Thank you so much. And also for the purpose of the .. of the tape, my name is PW. I'm here today with participant P003. It's the 4th of May. And the time is just coming up to 20 to 4.

0:0:37.300 --> 0:0:52.490
PW(LC CLINIC)
OK. Thank you so much for taking part in this today. And as mentioned it's, you know an interview which is gonna focus entirely on your kind of experiences of going through the long COVID clinic back in 2020. It wasn't, I think, yeah.

0:0:52.540 --> 0:0:52.910
P003
Yeah.

0:0:53.880 --> 0:1:2.650
PW(LC CLINIC)
Yeah. So just start with like a very kind of broad question and OK, what was your experience like of being referred to the long COVID clinic?

0:1:5.60 --> 0:1:7.390
P003
It was largely really positive.

0:1:12.10 --> 0:1:12.370
PW(LC CLINIC)
Umm.

0:1:7.560 --> 0:1:31.240
P003
And I mean, after it, I felt a bit desperate at the time, sort of suddenly being unwell with a whole slew of symptoms, and I feel like I was quite lucky. Actually, when I got referred when I sort of been in touch with my GP, I seemed to manage to get referred over quite relatively quickly. I know a lot of people subsequently had quite long waits just because of the sheer numbers.

0:1:31.970 --> 0:1:32.440
PW(LC CLINIC)
Yeah.

0:1:32.520 --> 0:1:35.540
P003
So no, it was it felt like a very straightforward process.

0:1:37.140 --> 0:1:37.760
P003
Long enough.

0:1:36.400 --> 0:1:43.670
PW(LC CLINIC)
OK, great. So it's it was fairly fairly easy to get an appointment and it didn't take too long to get that sort of first appointment. Would that be a fair thing to say?

0:1:42.760 --> 0:1:48.300
P003
No. Yeah. And then it was all, I think every appointment I had was was just over the phone.

0:1:49.350 --> 0:1:49.740
PW(LC CLINIC)
Right.

0:1:49.420 --> 0:1:51.310
P003
But I found it extremely helpful.

0:1:52.0 --> 0:1:57.830
PW(LC CLINIC)
OK. And how did you in the 1st place find out about the clinic?

0:1:59.390 --> 0:1:59.800
PW(LC CLINIC)
Yeah.

0:1:59.350 --> 0:2:29.260
P003
I'm I'd got in touch with my GP. I I was unwell, started to feel unwell with COVID symptoms and in March 2020, just days before the 1st lockdown started, and then just wasn't well and didn't get better and didn't get better and it just kept going on. So I touched in a few times with my GP and then I think it was towards the summer of that year. I got in touch and she, she said, look there, there's this new clinic started up which you can refer.

0:2:28.860 --> 0:2:29.280
PW(LC CLINIC)
Yeah.

0:2:30.60 --> 0:2:32.890
P003
So that was the first I I heard about it from the GP.

0:2:33.420 --> 0:2:44.610
PW(LC CLINIC)
OK. And if it's possible to say no, we got obviously cast your mind back to 2020, which seems like an eternity ago now, doesn't it? But how did getting referred to the clinic make you feel if that's possible to say?

0:2:47.190 --> 0:2:47.560
PW(LC CLINIC)
Right.

0:2:45.240 --> 0:2:50.630
P003
I was thrilled to be honest, because I just felt quite desperate at the time. I just seemed to be.

0:3:1.110 --> 0:3:1.440
PW(LC CLINIC)
Hmm.

0:3:3.420 --> 0:3:3.930
PW(LC CLINIC)
Yeah.

0:2:51.400 --> 0:3:14.780
P003
Unwell with a whole slew of symptoms and not getting better, and I think obviously you know, seeing what was going on globally at the time it it all quite alarming. So for me, I felt so relieved actually just to feel like there's anything I might be able to do to, you know, to try and help deal with my symptoms and understand things a bit better.

0:3:15.410 --> 0:3:17.50
PW(LC CLINIC)
Absolutely, yeah.

0:3:17.940 --> 0:3:27.360
PW(LC CLINIC)
So again, you know, appreciate that it is a good while ago now, but I just wanted, you know if you can remember sort of what the first your your first appointment involved?

0:3:28.670 --> 0:3:28.840
PW(LC CLINIC)
The.

0:3:29.720 --> 0:3:32.730
P003
Well, First off, it was a big questionnaire to fill in, I think.

0:3:32.930 --> 0:3:33.460
PW(LC CLINIC)
OK.

0:3:34.310 --> 0:3:46.430
P003
And then the first telephone appointment with. So it was with the the GP who was running the long COVID clinic. I think we just had a chat on the phone instead of went through all of my symptoms and.

0:3:52.710 --> 0:3:53.140
PW(LC CLINIC)
Yeah.

0:3:46.960 --> 0:3:59.70
P003
Umm, just kind of talked, talked it all through everything that was going on as far as I remember, which I found very reassuring. And then and then I can't remember it was that appointment or subsequent one and it was this case of sort of.

0:4:0.260 --> 0:4:3.720
P003
Triaging me and putting various referrals into.

0:4:3.380 --> 0:4:3.740
PW(LC CLINIC)
Right.

0:4:5.150 --> 0:4:5.610
PW(LC CLINIC)
Yeah.

0:4:6.680 --> 0:4:7.20
P003
Yeah.

0:4:7.730 --> 0:4:19.470
PW(LC CLINIC)
OK. And all of these, I totally appreciate that we're talking, you know, a a good good while ago, but you remember approximately how long you might have spent on that first appointment.

0:4:21.150 --> 0:4:24.120
P003
Oh gosh, that's a good question, I'm remember.

0:4:22.860 --> 0:4:26.130
PW(LC CLINIC)
Yeah. So, sorry. Sorry. OK. Yeah, it's a difficult one.

0:4:30.260 --> 0:4:30.650
PW(LC CLINIC)
Yeah.

0:4:25.760 --> 0:4:34.180
P003
It didn't feel like it. I mean, it wasn't. It wasn't like just a quick 10 minute conversation. Not like I had a bit longer, you know, to properly go into things.

0:4:34.760 --> 0:4:39.890
P003
Umm, so I don't. I don't know. It might have been 20 minutes. It might have been half an hour.

0:4:40.80 --> 0:4:46.900
PW(LC CLINIC)
Yeah. Yeah, OK. And that was just with the the GP, as you say, it wasn't it over the phone, is that right? The GP of the from the long COVID service?

0:4:47.0 --> 0:4:47.930
P003
That's it, yeah.

0:4:47.820 --> 0:4:53.90
PW(LC CLINIC)
Yeah. And again, is it possible to say kind of like how you felt after your first appointment?

0:4:54.10 --> 0:4:54.760
P003
Umm.

0:4:55.490 --> 0:4:59.240
P003
I think just probably quite reassured and quite listened to.

0:4:59.700 --> 0:5:0.100
PW(LC CLINIC)
Yeah.

0:4:59.630 --> 0:5:4.440
P003
And I mean the whole all of the symptoms I was having and just the whole picture, everything felt a bit.

0:5:8.120 --> 0:5:8.510
PW(LC CLINIC)
Yeah.

0:5:5.620 --> 0:5:15.160
P003
Confusing and a bit nebulous and hard to define at the time, so to actually speak to someone who just totally got it and understood the issues for such a relief.

0:5:15.670 --> 0:5:16.20
PW(LC CLINIC)
Yep.

0:5:15.530 --> 0:5:18.980
P003
Umm, so I guess it made me feel, you know, hopeful that.

0:5:19.640 --> 0:5:23.480
P003
You know might be able to do something about some of these symptoms.

0:5:23.150 --> 0:5:28.440
PW(LC CLINIC)
That's our symptoms. Yeah. So quite kind of reassured, I suppose. After after your first appointment.

0:5:28.680 --> 0:5:30.120
P003
That's it. Yeah, yeah.

0:5:30.220 --> 0:5:44.430
PW(LC CLINIC)
Yeah. And you sort of said that you sort of had an ongoing contact with the clinic, you know, is it possible to say approximately how long you were, you know you had a points with a clinic over what sort of time span you were within their service, if that makes sense?

0:5:44.490 --> 0:5:46.690
P003
Yeah, I'm just trying to think. I think it was a.

0:5:47.380 --> 0:5:49.580
P003
I think it was about a year perhaps.

0:5:49.860 --> 0:5:50.330
PW(LC CLINIC)
OK.

0:5:50.760 --> 0:5:53.840
P003
On and off appointments, I mean sort of more, more.

0:5:54.830 --> 0:6:1.860
P003
More thankless than appointments to get things started at the beginning, you know, and deal with various referrals and things, but I.

0:6:3.10 --> 0:6:5.180
P003
I definitely remember speaking to.

0:6:6.740 --> 0:6:8.70
P003
To the lady there.

0:6:9.120 --> 0:6:10.640
P003
In 2021.

0:6:11.670 --> 0:6:12.140
PW(LC CLINIC)
Yeah.

0:6:11.180 --> 0:6:17.630
P003
I'm sort of, I think probably to in the summer of 21. So I think I was definitely sort of still.

0:6:19.500 --> 0:6:20.820
PW(LC CLINIC)
Yeah, yeah.

0:6:18.680 --> 0:6:22.310
P003
You know in their system, I think it. Yeah, yeah.

0:6:22.380 --> 0:6:30.420
PW(LC CLINIC)
OK. And again, probably difficult to say, but approximately how often would you say that you had contact with the clinic?

0:6:32.440 --> 0:6:36.350
P003
As I said, it was probably more frequently to start with, so I don't know probably.

0:6:40.100 --> 0:6:41.220
PW(LC CLINIC)
Every couple months, yeah.

0:6:37.620 --> 0:6:47.980
P003
I don't know. Maybe if we couple of months or something what direct contact with them or you know an onward appointment that to come out of my conversations. But yeah, I think I.

0:6:47.640 --> 0:6:48.10
PW(LC CLINIC)
Yeah.

0:6:48.740 --> 0:6:51.770
P003
We had the sort of initial conversation, then some things have been in place and then.

0:6:52.490 --> 0:6:55.780
P003
And then I think they sort of checked in every now and again.

0:6:57.630 --> 0:7:3.110
P003

Yeah, for for a while. And then that sort of tapered out towards the end, I guess I didn't need it quite so much.

0:7:3.510 --> 0:7:11.780
PW(LC CLINIC)
Yeah. And what do you would you have had contact with the with the same member of staff or did it tends to be kind of different members of staff? I suppose depending on?

0:7:11.420 --> 0:7:15.80
P003
No, it was. It was always the same, the same GP.

0:7:15.380 --> 0:7:16.630
PW(LC CLINIC)
OK. OK, great.

0:7:17.530 --> 0:7:26.930
PW(LC CLINIC)
And again, I appreciate so allocate you know if you in contact with the clinic for that period of time, this might be fairly difficult question to answer but.

0:7:27.910 --> 0:7:33.100
PW(LC CLINIC)
Are you kind of? Have you got any recollection of kind of what kind of assessments and treatments that you received over that period?

0:7:34.340 --> 0:7:44.340
P003
Yeah, so Ohr, let me think. So I was so I was having chest pains, so I got referred to I had lung function tests.

0:7:45.120 --> 0:7:52.370
P003
And then I am again through phone appointments, had appointments with a pulmonary rehab physio.

0:7:53.400 --> 0:7:58.0
P003
Umm I had ohhh let me think.

0:7:59.970 --> 0:8:2.340
P003
Oh gosh, sorry. I'm trying to remember all these scans were cool.

0:8:2.10 --> 0:8:9.190
PW(LC CLINIC)
Now, honestly, honestly, no, no problem at all. Allocator, because it's, you know, I totally appreciate the the time that's lapsed in between. So yeah.

0:8:8.130 --> 0:8:11.820
P003
Yeah, I had a I think it was a.

0:8:15.260 --> 0:8:15.710
PW(LC CLINIC)
Yeah.

0:8:12.480 --> 0:8:18.650
P003
Would it be a CT scan of my lungs? They they like put a sort of dye in to have a look.

0:8:19.200 --> 0:8:19.710
PW(LC CLINIC)
OK.

0:8:19.930 --> 0:8:21.950
P003
That be, I think I had a CT scan of my lungs.

0:8:23.250 --> 0:8:24.800
P003
I had.

0:8:26.670 --> 0:8:30.910
P003
I started getting palpitations so I had a sort of.

0:8:31.800 --> 0:8:32.480
P003
And.

0:8:38.420 --> 0:8:38.890
PW(LC CLINIC)
Right.

0:8:33.740 --> 0:8:40.360
P003
Oh God, I sort of overnight 24 or 48 hour monitor. Put on me to see if there was funny going on there.

0:8:41.60 --> 0:8:48.660
P003
Umm, what else did I have? I I got referred to a service called activate that was a chronic fatigue service.

0:8:48.970 --> 0:8:49.780
PW(LC CLINIC)
Yeah. OK.

0:8:49.890 --> 0:8:52.680
P003
They they were absolutely fantastic. They were brilliant.

0:8:53.460 --> 0:8:53.940
PW(LC CLINIC)
That's great.

0:8:54.430 --> 0:9:9.540
P003
And what else? And I also got referred to. I also had insomnia start with all of this. So I was referred to a service called Sleep Station, which was a sort of app based sleep training offering.

0:9:10.60 --> 0:9:14.40
PW(LC CLINIC)
OK. And was was, was that via activate as well, was it or was that something?

0:9:13.930 --> 0:9:15.880
P003
No, that was actually.

0:9:17.400 --> 0:9:18.990
P003
Ohh how was I referred to that?

0:9:21.740 --> 0:9:25.890
P003
Oh, good to know. I'm just trying to think was that actually through my normal DP or was that through that so?

0:9:27.470 --> 0:9:27.770
P003
They.

0:9:25.660 --> 0:9:28.610
PW(LC CLINIC)
Ah, OK, OK. That that, yeah, that would make sense.

0:9:28.700 --> 0:9:30.970
P003
No. Do you know what that was? Through my normal GP?

0:9:30.950 --> 0:9:32.740
PW(LC CLINIC)
And would you pay? Yeah. OK.

0:9:32.680 --> 0:9:36.350
P003
I had that at the same time as the the activate stuff.

0:9:36.950 --> 0:9:40.440
PW(LC CLINIC)
OK. And both, did you find both beneficial or?

0:9:40.0 --> 0:9:43.250
P003
The Anything was an absolute nightmare, I've gotta say.

0:9:43.770 --> 0:9:44.900
PW(LC CLINIC)
The app, yeah.

0:9:43.570 --> 0:9:45.30
P003
And it just.

0:9:45.630 --> 0:9:52.440
P003
And I mean I it was. It was weird. It was about several months after I started the long COVID.

0:10:3.890 --> 0:10:4.180
PW(LC CLINIC)
Art.

0:10:4.700 --> 0:10:9.410
P003
Sleep problems in my life. So then I got referred to it and.

0:10:10.220 --> 0:10:15.910
P003
It it was, it was. I mean, I followed every bit of advice to the letter because I was just desperate.

0:10:16.260 --> 0:10:16.630
PW(LC CLINIC)
Yeah.

0:10:25.610 --> 0:10:26.70
PW(LC CLINIC)
Yeah.

0:10:17.500 --> 0:10:35.100
P003
And it just didn't help at all. They give you a sort of sleep training, no sort of sleep hygiene score at the beginning. I mean mine was. And then at the end, I think it was like a 12 wep003 program where you put all this stuff into place. I think it had barely gone up by, you know, a few percent.

0:10:35.860 --> 0:10:42.270
P003
And they said have concluded, oh, I think there's something else going on here. And so I was like, yes, I think it's long COVID.

0:10:42.710 --> 0:10:43.210
P003
I'm.

0:10:43.0 --> 0:10:45.690
PW(LC CLINIC)
Oh, right, OK. So you went full circle kind of thing.

0:10:46.50 --> 0:10:53.160
P003
Yeah, it just didn't help and I've got I think I'd also, you know, it's a kind of paradox of sleep. The more you think about it, the more you can't do it. But.

0:10:53.170 --> 0:10:53.620
PW(LC CLINIC)
Yeah.

0:10:56.640 --> 0:10:57.10
PW(LC CLINIC)
Yeah.

0:11:6.180 --> 0:11:6.410
PW(LC CLINIC)
Yeah.

0:11:17.210 --> 0:11:17.510
PW(LC CLINIC)
Umm.

0:10:53.860 --> 0:11:25.990
P003
Can't sleep? That's kind of all you can think about. So UMI think I I followed all the advice and it didn't really help me and honest. It just dominated my entire life. I felt really awful. And the whole time. And then a little while after I finished it, I was still trying to carry on with the advice. And then I think I just was so tired. I just thought I'm just gonna try and get back to my normal pattern. And I think I just relaxed a bit and then I had to sleep a bit and it did. It did get better. I I have to say it's of all the long cable things. All my symptoms have cleared up now apart from.

0:11:27.410 --> 0:11:27.890
PW(LC CLINIC)
You're right.

0:11:26.90 --> 0:11:37.420
P003
The insomnia and I go long periods where I I go to. Well not not. I go through periods when I can't sleep and then we sleep is still a really weak area for me. So the slightest interruption to my routine.

0:11:37.980 --> 0:11:45.940
P003
I'm going somewhere and sleeping in a different bed or a different room just knocks my sleep out and it takes me a while to get it back again.

0:11:53.130 --> 0:11:53.600
PW(LC CLINIC)
Yeah.

0:11:47.400 --> 0:11:54.770
P003
And I started a new job at the beginning of this year, and I didn't sleep for 2 1/2 months, which was just awful. So unless.

0:11:54.400 --> 0:11:57.330
PW(LC CLINIC)
Perfect for a new job, right? Yeah.

0:11:55.850 --> 0:11:59.870
P003
Yeah, which sounds really hard to retain your information when you can't sleep.

0:12:1.250 --> 0:12:1.740
P003
Umm.

0:11:59.720 --> 0:12:4.350
PW(LC CLINIC)
Yeah, absolutely. Ohr wow. Is it something you still getting support for, P003?

0:12:12.890 --> 0:12:13.280
PW(LC CLINIC)
Hmm.

0:12:4.640 --> 0:12:23.730
P003
Well, I I did. I did at the beginning of the year. Get in touch with my GP again saying, oh God, it's it's come back, you know, really badly. And they put much with a local well-being service and I'm doing a sort of, I mean it was it was a bit similar to the sleep program did before sort of I think it's a CBT CBT's other word.

0:12:23.860 --> 0:12:24.660
PW(LC CLINIC)
Yeah.

0:12:24.720 --> 0:12:45.850
P003
Umm style thing where it's all through an app you you follow different modules where you get different advice of sort of things to do and you know monitor your amount of sleep you're getting each night. It didn't enormously help me to be honest. I just feel like those sorts of things don't seem to work. It was more just I just started to relax and I got my feet a bit more under the table in my new job and.

0:12:45.570 --> 0:12:52.280
PW(LC CLINIC)
OK, so like you saw the the parts of the the jigsaw sort of fell into the place and that was probably more the reason for the improvement in sleep.

0:12:53.640 --> 0:12:54.40
PW(LC CLINIC)
Umm.

0:12:51.480 --> 0:13:7.480
P003
I think I that's it. You know, it was need my other half was about to start a new job and he, you know, was gonna be working away a few nights a wep003. And I think I just had that in my mind. And as soon as we kind of got to that, it's like oh, actually this is fine. I can cope. And so it's more kind of life we've got in the way.

0:13:7.550 --> 0:13:8.550
P003
Yeah, yeah.

0:13:7.380 --> 0:13:9.890
PW(LC CLINIC)
Yeah, yeah, get in the way of a lot of things.

0:13:12.40 --> 0:13:13.170
P003
But it seemed to be.

0:13:15.40 --> 0:13:24.800
P003
Yeah, it's it's just a bit of a wep003 area for me now, which is frustrating because I've never had sleep issues before at all and it it feels a bit like the COVID sort of set something in motion. And now I can't quite.

0:13:27.820 --> 0:13:28.110
PW(LC CLINIC)
Umm.

0:13:25.770 --> 0:13:34.810
P003
Philly get on top of it. But you know it's it's it's largely OK for for wep003s at a time. So that's, you know, that's enough.

0:13:37.10 --> 0:13:37.440
P003
Yeah.

0:13:35.90 --> 0:13:38.340
PW(LC CLINIC)
Yeah, it's enough to get by, I suppose, yeah.

0:13:38.490 --> 0:13:46.610
P003
So yeah, so that the sleep services were just didn't really work for me. That's it's more. I just needed to do it in my own time.

0:13:48.50 --> 0:13:54.790
P003
The activate service was absolutely fantastic, so that was for chronic fatigue.

0:13:58.360 --> 0:13:58.630
PW(LC CLINIC)
Umm.

0:14:9.770 --> 0:14:10.130
PW(LC CLINIC)
Right.

0:13:55.0 --> 0:14:12.170
P003
And which I developed from the long COVID and that was brilliant. So that was seeing an occupational therapist and a psychologist. So that was. I went in in person for an initial assessment with them, which was probably the conversation for about an hour.

0:14:12.630 --> 0:14:17.690
P003
Umm. And then it was sort of sessions online sessions with them.

0:14:18.290 --> 0:14:27.140
P003
It's coming through different sort of techniques and things doing some relaxation exercises, you know, having some sessions with the psychologist just about, you know.

0:14:30.210 --> 0:14:30.520
PW(LC CLINIC)
Mm-hmm.

0:14:38.750 --> 0:14:39.100
PW(LC CLINIC)
Umm.

0:14:28.80 --> 0:14:48.810
P003
Just about my history and stuff and and then they plugged me into a group, A sort of a a group. They were running online. All people who had long COVID, and that was absolutely brilliant. That was such to help. So I think it was sort of wep003ly sessions we had with a sort of different theme each wep003 going through different sort of.

0:14:58.190 --> 0:14:59.520
PW(LC CLINIC)
Yeah, yeah.

0:15:9.610 --> 0:15:9.860
PW(LC CLINIC)
Yeah.

0:14:49.860 --> 0:15:11.180
P003
Things we can do to to sort of, you know, pace yourself and different ways to sort of, you know, challenge your thinking on various things. It was it was so helpful. I felt so supportive through that. And I think just also being in a group with other people who are going through the same things and just you know just got it was in reassuring.

0:15:12.210 --> 0:15:24.290
PW(LC CLINIC)
Fantastic. So yeah, so the online sort of even though was online, so you know no face to face in regards to the group, but you know, even though it was online, it still worked really well and allocate. Would that be sort of a fair reflection here?

0:15:23.70 --> 0:15:34.360
P003
Yeah, it really did. And they sort of put us, we'd sort of, you know, talk through a lot of things and then they'd put us into little breakout sessions of sort of two or three people. So it was a bit more comfortable to.

0:15:34.750 --> 0:15:35.260
PW(LC CLINIC)
Yeah.

0:15:35.420 --> 0:15:40.50
P003
Talk about staff and that was great. And yeah, it just it just felt like a.

0:15:39.690 --> 0:15:40.130
PW(LC CLINIC)
Brilliant.

0:15:40.710 --> 0:15:46.340
P003
Important proper to have to sort of, you know, hold me up when it was going through quite difficult time.

0:15:46.460 --> 0:16:9.830
PW(LC CLINIC)
Yeah, absolutely. OK. And you know, in terms of sort of like a, you know, going through what we say sort of like, you know your care pathway, did you feel as if the process was always explained to you and you had a good idea of what was happening and why you were having certain being referred to certain places for support with your symptoms?

0:16:10.350 --> 0:16:24.60
P003
Yeah, yeah, no, it it always made sense. Everywhere I was being referred to and it was very much in response to, you know, everything I was raising that was going on to me. It's like, right, OK. Well then we'll plug you into this service or these tests or things.

0:16:37.240 --> 0:16:37.720
PW(LC CLINIC)
OK.

0:16:25.500 --> 0:16:54.120
P003
I mean, this is kind of I I don't feel like all of it particularly helped me, but I think that's more the nature of long COVID than anything to do with the clinic itself. You know, the sort of some of the, the sort of palpitations and chest pains and things I was having. I think this is an experience a lot of people on COVID, do you have all the tests and everything says everything's absolutely fine and it was still sort of unclear what on Earth is going on, but.

0:16:56.490 --> 0:16:56.830
PW(LC CLINIC)
Umm.

0:16:54.270 --> 0:17:9.820
P003
I think that was the nature of the beast, you know? You know nobody nobody quite knew. And I suppose it was case of being able to rule out certain things. So in in some sense I found that quite frustrating. But, you know, that's that was the nature of long, long COVID when I had it.

0:17:10.200 --> 0:17:10.740
PW(LC CLINIC)
Yeah.

0:17:11.300 --> 0:17:18.670
P003
So yeah, but no, that it it definitely was always explained to me what what we were gonna be investigating or or trying.

0:17:20.190 --> 0:17:20.590
P003
Yeah.

0:17:21.270 --> 0:17:49.720
PW(LC CLINIC)
And allocate just to you know, the the frustration that was that around just the fact that you know you had to sort of go to a lot of different places and and and like you saying that was obviously due to another condition et cetera, et cetera. But that was the frustrating part of it was just the fact that there was you know would it be less frustrating obviously if it wasn't a novel situation and it could have been a bit more kind of clear cut as to you've got this. So therefore you go there, was it the kind of?

0:17:52.920 --> 0:17:53.300
P003
I think.

0:17:49.800 --> 0:17:54.630
PW(LC CLINIC)
And that sort of nebulous aspect of being referred to various different places.

0:17:54.980 --> 0:18:7.540
P003
Yeah, I mean, to be totally honest, I was happy to be referred to anyone and everyone that I just thought anything that's on offer. I'm gonna do it if it might help me, great or, you know, define a bit more what's going on. So I was very happy with that.

0:18:8.180 --> 0:18:30.10
P003
Umm, I think it was just, you know, it was so new and unknown. And I I just didn't seem to get better for months and months and then, you know, some new some symptoms started with the beginning and then some other symptoms like the palpitations and the insomnia started a few months further down the line. And it was just being like ohhh God, am I gonna be ill forever.

0:18:30.360 --> 0:18:30.760
PW(LC CLINIC)
Hmm.

0:18:31.820 --> 0:18:41.690
P003
I think that was really the the frustration and the worry in all of it that, you know, if I keep having tests and being told everything's fine, then why do I not feel fine? It was sort of trying to.

0:18:42.530 --> 0:18:43.880
P003
Manage that really.

0:18:46.490 --> 0:18:46.920
PW(LC CLINIC)
OK.

0:19:6.780 --> 0:19:7.130
PW(LC CLINIC)
Umm.

0:19:12.900 --> 0:19:13.410
PW(LC CLINIC)
Yeah.

0:18:45.340 --> 0:19:16.730
P003
Yeah. I mean, in, in, in the, in some ways it was, you know, it was incredibly helpful to have all these appointments and feel supported on that front. It was it was just the unknown nature of it all. You know where it was gonna lead and and actually, I mean, it took me about 2 1/2 years, but all of my symptoms just over time improved and have have got better apart from the the on insomnia that's still raises its head every now and again and and you know it seemed for me. I feel like I've been really lucky it's just.

0:19:19.780 --> 0:19:20.160
PW(LC CLINIC)
Yeah.

0:19:16.950 --> 0:19:25.100
P003
It just needed some time, you know, and a lot of people haven't got better and I still grappling with a lot of things so.

0:19:24.820 --> 0:19:38.20
PW(LC CLINIC)
Absolutely. Yeah. Yeah. And so you kind of touched on this a little bit. I just wondered, you know, if it's possibly how confident, you know you felt in your diagnosis and obviously subsequent treatment.

0:19:41.90 --> 0:19:50.480
P003
Yeah, I I think I felt very confident in the diagnosis. It was just such a relief. It sort of just made sense to explain what what was going on.

0:19:51.960 --> 0:19:56.870
P003
So that side of things was was was very straightforward.

0:19:57.10 --> 0:19:57.430
PW(LC CLINIC)
OK.

0:19:57.940 --> 0:19:59.50
P003
I actually have.

0:19:59.350 --> 0:20:28.460
P003
I'm a chronic health condition that there is unrelated to long COVID. There is quite there and lots of people haven't really heard of and it was a sort of strange thing that I suddenly had this new syndrome and all these symptoms that I didn't really understand didn't make sense. But everyone I know was like, oh, yeah, right. It's long COVID. And we're sort of understanding it. Where is my other long chronic health condition I've had for years? That's quite rare. People haven't heard of people just.

0:20:29.260 --> 0:20:36.830
P003
Don't get it and don't know how to talk to me about it and sort of their eyes sort of glaze over if I start to talk about it. So it was a slightly strange situation of.

0:20:37.270 --> 0:20:37.910
PW(LC CLINIC)
Right.

0:20:38.40 --> 0:20:38.890
P003
Managing.

0:20:39.790 --> 0:20:44.220
P003
To sort of health issues at once and.

0:20:43.890 --> 0:20:47.600
PW(LC CLINIC)
And having that sort of disparity between the two in the yeah.

0:20:46.500 --> 0:20:57.300
P003
Yeah, it was. It was pretty odd, actually. People would sort of quite openly come and ask me like ohh, how's you know, how are you doing? How is the long COVID? What sort of treatments are you having? You know, are you getting any improvements and?

0:21:5.750 --> 0:21:7.690
PW(LC CLINIC)
Right, right. Yeah.

0:20:58.130 --> 0:21:10.840
P003
Apart from with my own family who have the same syndrome as me, I've I've never had that anybody else about my long term health condition, but so slightly it just yeah, it put it into slightly stark relief, I suppose.

0:21:11.0 --> 0:21:11.510
PW(LC CLINIC)
Umm.

0:21:12.0 --> 0:21:14.30
P003
In that sense, which was just quite interesting really.

0:21:13.970 --> 0:21:15.960
PW(LC CLINIC)
Absolutely. Yeah. Yeah, very interesting.

0:21:17.770 --> 0:21:23.780
PW(LC CLINIC)
Kind of like the, you know, the novel condition, then a novel way of treating it and obviously some.

0:21:25.790 --> 0:21:37.320
PW(LC CLINIC)
Benefits to that isn't there the way that sort of like people approached the condition? It sounds like, you know from a a supportive aspect. You know, they're supportive aspects of bit, you know, receiving treatment.

0:21:38.800 --> 0:21:43.220
PW(LC CLINIC)
Yeah, very interesting. Point allocate. Yeah. So you know how how did that?

0:21:44.50 --> 0:21:53.300
PW(LC CLINIC)
You know, how did that make you feel? Would have having those two things going on, you know. How how did you feel about then? You know, in contrast to?

0:21:54.220 --> 0:21:56.410
PW(LC CLINIC)
You're kind of your your chronic condition, you know.

0:21:58.110 --> 0:22:1.930
PW(LC CLINIC)
What were the main sort of like, you know, the impacts of that on on the way that you feel towards?

0:22:3.780 --> 0:22:5.160
PW(LC CLINIC)
The clinic and and what have you.

0:22:6.150 --> 0:22:9.520
P003
Umm I suppose. I suppose it it's.

0:22:10.700 --> 0:22:20.800
P003
I think it's sort of over time made everyone more open and a bit more comfortable about talking about their health and it just being part of a conversation. So in that sense.

0:22:20.530 --> 0:22:20.910
PW(LC CLINIC)
Umm.

0:22:21.790 --> 0:22:26.360
P003
In some ways, it's because I've still got my original health condition, and that's something I'm always gonna have.

0:22:32.360 --> 0:22:32.880
PW(LC CLINIC)
Yeah.

0:22:47.680 --> 0:22:48.60
PW(LC CLINIC)
Yeah.

0:22:28.660 --> 0:22:55.0
P003
It's made me feel a little bit freer to talk about it with people and and I think if peoples eyes do start to glaze over, I think well, you know, maybe if you've heard about it from me, you might, you know be able to help someone else in the future or you might just have a little bit more understanding of people's health issues in sort of invisible health issues that people live with all the time. You never know what people are dealing with. So.

0:22:55.50 --> 0:22:55.720
PW(LC CLINIC)
Umm.

0:22:55.900 --> 0:22:57.270
P003
Common sense I sort of.

0:23:2.810 --> 0:23:3.210
PW(LC CLINIC)
Right.

0:22:58.280 --> 0:23:16.730
P003
I think people, perhaps it, made me feel a little bit more open about things and gone through all of that. And you know, people were just interested to know what what was happening on the long, you know, from the long COVID service. And most people are just really impressed to hear, you know, my friends and family are doing to sort of services. I was being plugged into.

0:23:17.430 --> 0:23:25.300
P003

Umm, so you know overall I'm I feel really you know pleased to have been plugged into all of that what it was all.

0:23:24.990 --> 0:23:25.520
PW(LC CLINIC)
Yeah.

0:23:26.450 --> 0:23:36.700
PW(LC CLINIC)
Yeah. Well, I mean, you probably kind of answered this, but I'll ask you anyway. P003, you know, so did you feel supported by staff, you know, in relation to the interactions around?

0:23:37.810 --> 0:23:41.420
PW(LC CLINIC)
You know, you're the diagnosis long COVID and any subsequent treatment.

0:23:42.460 --> 0:23:52.530
P003
Yeah, I did. I mean, I've gotta say, there were the sort of hospital tests, like the sort of lung function test and the scan and.

0:23:53.510 --> 0:23:54.80
P003
The sort of.

0:23:56.170 --> 0:24:4.990
P003
Umm, what you call it? You know you have this this thing stuck on your test to monitor your heart rate. I can't. What you call that? Those sides of things that felt quite functional.

0:24:5.770 --> 0:24:7.370
P003
That felt a little bit like.

0:24:8.50 --> 0:24:10.500
P003
You know you're in and you're out or not. If you go and.

0:24:10.280 --> 0:24:11.130
PW(LC CLINIC)
Right. OK.

0:24:18.270 --> 0:24:18.760
PW(LC CLINIC)
Right.

0:24:11.660 --> 0:24:23.470
P003
Hey, I wasn't as sort of comfortable with all of that, I suppose in terms of how it was explained or what it being it was just like no, it has to all fine off your game, it's like, OK, But what does that actually mean?

0:24:24.170 --> 0:24:24.750
PW(LC CLINIC)
Yeah.

0:24:43.270 --> 0:24:43.680
PW(LC CLINIC)
Umm.

0:24:23.910 --> 0:24:45.950
P003
And the OHR, I mean the people in the activate service were just so brilliant, they really were. And the I saw a lovely pulmonary rehab physio as well who was really supportive and you know incredibly helpful and just really sort of got it, which I mean that's that's half of it really when you're speaking to.

0:24:46.880 --> 0:24:55.880
P003
To medical people going through these things, it's just sort of feeling like you're just understood, even if you know they're able to help you or not that that it, you know, helps enormously.

0:24:56.400 --> 0:24:58.270
P003
Umm so yeah.

0:24:59.340 --> 0:25:15.230
PW(LC CLINIC)
Yeah. So quite a contrast in between sort of like the actual clinic and then places that you referred to. So it sounded like perhaps your experience of communicating, you know, communication with staff was perhaps better with the clinic than it was to subsequent sort of referrals. Would that be fair to say allocate?

0:25:15.650 --> 0:25:17.260
P003
Yeah. UM.

0:25:18.360 --> 0:25:18.680
P003
I'm.

0:25:20.780 --> 0:25:27.10
P003
I spoke to in in the long COVID service staff. Those were always really helpful conversations because.

0:25:27.850 --> 0:25:29.170
P003
You know it's.

0:25:41.60 --> 0:25:41.430
PW(LC CLINIC)
Hmm.

0:25:29.790 --> 0:25:44.450
P003

Everything I was saying was definitely, you know, falling on receptive ears. And it was sort of understood and, you know, taken seriously as well, which which felt like quite a big deal at the time. And then certain services I got plugged into subsequently.

0:25:45.870 --> 0:25:55.840
P003
Where it's, you know, very helpful and it's just, you know, speaking to kind of understanding individuals where it's sort of test side of things felt a bit more perfunctory I suppose.

0:25:56.10 --> 0:26:3.700
PW(LC CLINIC)
Yeah, yeah. OK. And, you know, at any point in this delicate, you know, did you feel able to voice any concerns?

0:26:5.130 --> 0:26:8.820
PW(LC CLINIC)
You know in, in, in relation to perhaps the you know the testing and and what have you.

0:26:12.800 --> 0:26:13.180
PW(LC CLINIC)
No.

0:26:10.620 --> 0:26:15.50
P003
I'm not really, and I suppose I never really particularly needed to.

0:26:16.350 --> 0:26:18.740
P003
I think I think sometimes with anything just.

0:26:19.360 --> 0:26:19.910
P003
Umm.

0:26:20.940 --> 0:26:30.800
P003
I didn't know. I often feel this. Unless if you're going in for any kind of medical testing or anything, it's sort of, I don't know. I find sometimes walking in hospitals. It's like this whole universe that I feel I don't quite understand.

0:26:40.440 --> 0:26:41.20
PW(LC CLINIC)
Yeah.

0:26:32.780 --> 0:26:59.210
P003
I yeah. You know you're there. You're there for that little bit and like, sort of, you know, I don't quite understand how it all fits in with everything, but yeah, I felt like, you know, I was, I was getting this whole load of appointments and things three. So I felt like, you know, I'm just gonna. I'm just gonna do it. And, you know, I was very much able to earn time. I spoke to the the GP and the long COVID clinic able to run through things with her and be like, OK, but what does that actually mean? And.

0:26:59.510 --> 0:27:0.800
PW(LC CLINIC)
OK, OK.

0:27:1.240 --> 0:27:2.70
P003
And yeah.

0:27:2.140 --> 0:27:3.420
PW(LC CLINIC)
Yeah, OK. Brilliant.

0:27:5.360 --> 0:27:26.120
PW(LC CLINIC)
Just in relation to kind of a I tell you appreciate that obviously some of your interactions were face to face especially for sort of a diagnostic tests. I mean yeah, not something you can do virtually, but where you did have kind of virtual telephone or virtual appointments allocate, how did you find that process of sort of receiving care that way?

0:27:33.680 --> 0:27:34.180
PW(LC CLINIC)
Yeah.

0:27:27.430 --> 0:27:41.280
P003
I found it really good actually. I mean I think before of this I probably would have always preferred to do appointments in person. I just you know like person, but actually I mean partly feeling unwell and very fatigued. It just made life a lot easier.

0:27:42.600 --> 0:27:51.30
P003
And I think you know, it was over times where there was still lots of restrictions in place. And I was, you know, really worried about.

0:27:53.280 --> 0:27:53.670
PW(LC CLINIC)
Umm.

0:27:56.690 --> 0:27:57.130
PW(LC CLINIC)
OK.

0:27:51.730 --> 0:28:3.160
P003
Catching it again and what might happen, things are actually it. It felt very straightforward being able to do it home and and you know, not have to venture out necessarily. And I think it was sort of.

0:28:4.10 --> 0:28:7.940
P003
I don't know. It's sort of a whole new world, really, isn't it? It's just kind of how we got used to doing everything.

0:28:8.900 --> 0:28:9.310
PW(LC CLINIC)
Yeah.

0:28:8.420 --> 0:28:11.80
P003
And and I've rest similar time.

0:28:12.560 --> 0:28:15.550
P003
Which feels ridiculous now. I actually started a new job.

0:28:17.90 --> 0:28:20.440
P003
I've that period and and that was entirely working.

0:28:21.580 --> 0:28:36.610
P003
I've not heard everyone was entirely working from home, so I was just so I got kind of used to speaking to colleagues every day on teams and doing everything online. So I feel like quite quickly just got very used to that being you know how you how you did.

0:28:37.350 --> 0:28:37.690
P003
Things.

0:28:36.310 --> 0:28:41.860
PW(LC CLINIC)
It kind of, yeah, that's kind of quite normal. Yeah. Ohh not the dreaded new normal phrase. Yeah.

0:28:43.200 --> 0:29:7.10
PW(LC CLINIC)
Did you sort of like so in that period of time? Then P003, especially with the sort of like as you said, sort of fears around, you know, going into places like hospitals for appointments etcetera at that point in time, do you actually, would you say you preferred the sort of like online or telephone appointments rather than face to face, but would it be fair to say that if those weren't the circumstances, would you have a preference towards one or the other?

0:29:19.450 --> 0:29:19.780
PW(LC CLINIC)
Hmm.

0:29:28.740 --> 0:29:29.180
PW(LC CLINIC)
Yeah.

0:29:8.290 --> 0:29:31.490
P003
I yeah. And I think generally my preference would probably be to see people in person. I just feel like you can sort of connect bit better and have sort of slightly better conversations, but equally time and I'm very comfortable doing conversations online. And I think at the time it just it, it made much more sense at the time. Just you know thing going on with the restrictions and.

0:29:32.240 --> 0:29:41.450
P003
You know, at times I had kids home during lockdowns trying to home school and all that, so trying to fit external appointments in might have been a bit of a nightmare.

0:29:41.510 --> 0:29:44.980
PW(LC CLINIC)
Yeah, yeah. So it's quite convenient under the circumstances.

0:29:44.810 --> 0:29:46.100
P003
Yeah, yeah, absolutely.

0:29:45.780 --> 0:29:56.500
PW(LC CLINIC)
Yeah. Had you had you had, you know, prior to the COVID-19 pandemic, had you, you know, had any health related appointments carried out virtually prior to that?

0:29:56.990 --> 0:30:0.510
P003
I'm no I don't think I had actually.

0:30:2.430 --> 0:30:9.900
P003
No, I mean even just, you know, it's like even before then, just just getting a GP appointment. You know it was always sort of in person and.

0:30:10.130 --> 0:30:10.510
PW(LC CLINIC)
Hmm.

0:30:16.120 --> 0:30:16.600
PW(LC CLINIC)
Yeah.

0:30:10.920 --> 0:30:23.850
P003
Since then, you know it's a lot more phone consults than eat or an E console online, and it's really convenient, I think. So now I think prior to that, it had probably all been in person.

0:30:24.170 --> 0:30:33.520
PW(LC CLINIC)
Yeah, yeah. OK. Just got a couple more questions. OK, just checking that. Obviously the kids are still, you know, involved with Netflix. Everything's OK.

0:30:33.840 --> 0:30:35.350
P003
Is they? Don't worry, they're fine.

0:30:35.950 --> 0:30:50.660
PW(LC CLINIC)
OK. So again kind of a broad question just about, you know, would you say you were overall in

relation to the long COVID clinic, did you feel satisfied with the care that you received and if so, why?

0:30:52.340 --> 0:30:56.440
P003
Yeah, I was. I was very happy with with the care I received.

0:30:58.40 --> 0:31:4.240
P003
I think it just. I just felt listened to and taken seriously.

0:31:6.200 --> 0:31:7.110
P003
And.

0:31:9.390 --> 0:31:24.640
P003
Again, it was so to being able to say I'm presenting with all of these symptoms and you know that would almost straight away be acted on it like, OK, well, we'll plug you into this and we'll plug you into that and do all these onward referrals.

0:31:26.210 --> 0:31:29.500
P003
And and again in relation to my my other chronic health condition.

0:31:30.710 --> 0:31:35.690
P003
That can sometimes be a bit of a battle to get to see the right person or you know to.

0:31:36.0 --> 0:31:36.350
PW(LC CLINIC)
Umm.

0:31:36.570 --> 0:31:44.100
P003
No. If the doctor you're talking to has actually heard of the syndrome or knows about it, or you know, knows how to manage it.

0:31:44.550 --> 0:31:44.910
PW(LC CLINIC)
Yeah.

0:31:45.630 --> 0:31:51.480
P003
So I suppose comparing the two and that sense it it, it felt a bit like a dream actually just to sort of.

0:31:51.270 --> 0:31:52.340
PW(LC CLINIC)
Yeah. OK.

0:31:52.540 --> 0:32:0.90
P003

Maybe listen to and understood and and got. And you know, I felt very supportive through that supported through that whole period.

0:32:1.390 --> 0:32:8.580
P003
And it was just enormously reassuring, you know, I mean, there are times when I really felt like I was losing my mind just trying to grapple with all of this stuff and.

0:32:9.560 --> 0:32:17.270
P003
And you know, just try and manage all these health conditions day-to-day and and I think the worry that it is, it just gonna is this what life is just gonna be like now.

0:32:17.720 --> 0:32:18.790
PW(LC CLINIC)
Umm yeah.

0:32:18.620 --> 0:32:28.770
P003
Because it was, you know, extremely helpful to to have, you know, just have that understanding and feel like actually someone's someone supporting me here.

0:32:29.290 --> 0:32:41.240
PW(LC CLINIC)
Brilliant. OK. So educate last question you know, is there anything that you think could have been better you know from your experience of going through through the long COVID clinic?

0:32:42.730 --> 0:32:43.540
P003
I'm.

0:32:45.120 --> 0:32:46.820
P003
No, not really. I mean, I think the only thing.

0:32:53.530 --> 0:32:54.40
PW(LC CLINIC)
Right.

0:32:47.550 --> 0:32:59.150
P003
Would have, which is impossible because nobody knew at the time was just knowing what Earth was going on in my body. But that experience of everybody, wasn't it so much of it was just, you know, we didn't know about.

0:33:0.530 --> 0:33:5.810
P003
So no, in terms of the actual service itself, no, I I've you know top marks, I think I think it was.

0:33:6.610 --> 0:33:6.870
P003
Umm.

0:33:7.430 --> 0:33:28.680
PW(LC CLINIC)
OK, brilliant. And you know I totally appreciate we've covered a lot of ground allocate and it might be the case that there's been things you know have thought about about as we've gone through it that you wanted to mention. So just I did say last question before then, I I obviously lied. Have you got any kind of like final comments or anything that we didn't cover or you feel as if you wanted to highlight?

0:33:28.750 --> 0:33:35.550
PW(LC CLINIC)
Have you know that you perhaps started to talk about and we didn't get the chance to to go down a certain Ave.

0:33:39.930 --> 0:33:40.590
PW(LC CLINIC)
Have everything.

0:33:37.260 --> 0:33:40.930
P003
No, I think we probably, I think we probably covered it all as far as I'm concerned.

0:33:41.410 --> 0:33:48.240
PW(LC CLINIC)
Fantastic. OK with that then I'll just stop the recording.

-- ----- ----- -----

P005

0:0:0.0 --> 0:0:1.230
P005
Yeah, that's fine. That's fine.

0:0:-4.-980 --> 0:0:1.560
PW (LC clinic)
Sh, just cause I've I've had this before where the recording didn't work, so I've also got like a backup so hit record on that as well.

0:0:2.920 --> 0:0:10.900
PW (LC clinic)
OK. So OK, first and foremost, I'm here today with participant P005 my name is PW. It's the 28th of April 2023 and it is 11:37 AM.

0:0:24.210 --> 0:0:29.750
PW (LC clinic)
And Sh, are you just to make sure that you're happy for me to be recording this interview today?

0:0:30.100 --> 0:0:31.290
P005
Yeah, that's absolutely fine. Thank you.

0:0:31.800 --> 0:0:47.130
PW (LC clinic)

Thank you, Sh. OK, so we'll go straight into the interview questions and the first one is quite broad. So I'll just start with that. So you know how was your experience of being referred to the long COVID clinic?

0:0:47.970 --> 0:0:52.880
P005
I for me, I was. It was great because I think obviously when I caught COVID in 2020.

0:0:53.550 --> 0:0:55.300
P005
When I first bought it, it was obviously.

0:0:57.320 --> 0:0:57.690
PW (LC clinic)
Yeah.

0:0:56.110 --> 0:1:14.270
P005
You no one knew what was, you know. So actually for me, the care that I received was for me was was great. And I think having their facility of going to the long COVID clinic so early I thought was a great was great and I had obviously a lot of work colleagues that.

0:1:17.110 --> 0:1:18.280
PW (LC clinic)
Didn't have that option, yeah.

0:1:15.220 --> 0:1:21.40
P005
Didn't have that facility. So for me, I was really no. Yeah, I was really grateful that I was actually lucky enough.

0:1:28.810 --> 0:1:29.380
PW (LC clinic)
Yeah.

0:1:21.820 --> 0:1:30.260
P005
To to be referred and and live in the catchment area. Really I suppose for the long COVID clean a couple Lister. So yeah, no, really happy. Really pleased.

0:1:30.940 --> 0:1:31.540
P005
Pretty lucky.

0:1:30.650 --> 0:1:34.500
PW (LC clinic)
And did you find how? How difficult did you find it to to get an appointment?

0:1:35.610 --> 0:1:53.850
P005
And that's because of you. They they were always in contact with me. I I I I was there was there was never me having to chase them for anything. And I think I had a GP used to ring me quite freak

frequently and just to check what I was doing and I think I was under the respiratory physio as well so to be honest with you.

0:1:57.410 --> 0:1:59.460
PW (LC clinic)
No, it's quite straightforward.

0:1:54.830 --> 0:2:0.280
P005
I didn't really experience any any problems really. It was all they were all pretty pretty on it, if I'm honest.

0:2:10.860 --> 0:2:11.100
P005
Yep.

0:2:0.470 --> 0:2:13.670
PW (LC clinic)
Yeah. I mean, obviously, we cast our minds back a bit, is it can you remember approximately Sh just approached me like how long it took from you sort of you know reporting or you know being diagnosed with long COVID to getting your your first appointment with a long COVID clinic.

0:2:18.200 --> 0:2:18.570
PW (LC clinic)
Right.

0:2:22.980 --> 0:2:23.380
PW (LC clinic)
Yeah.

0:2:14.60 --> 0:2:25.950
P005
Umm it wasn't long because I end up in hospital and I think once I come out of hospital I was then referred by the list to hospitals. So I think I had a phone call probably within a wep003 to two wep003s.

0:2:26.180 --> 0:2:27.150
PW (LC clinic)
OK, OK.

0:2:26.940 --> 0:2:27.260
P005
So.

0:2:28.550 --> 0:2:39.690
PW (LC clinic)
And you know, again, it's quite a broad question, but you know, how did you feel about being referred to the long COVID clinic? Were there any sort of like a emotions that you can remember at at that time you know?

0:2:41.240 --> 0:2:48.890
PW (LC clinic)

Just that whole kind of, you know, your response, if you like to that process of you know long you've got long COVID and you're gonna be passed on to the long COVID clinic.

0:3:5.630 --> 0:3:5.980
PW (LC clinic)
Yeah.

0:2:49.360 --> 0:3:10.580
P005
I I think I think for me it was a it was a relief to know that actually I wasn't just gonna be forgotten about because like I say, we were so early on into understanding what COVID and long COVID was, that actually it was really nice to, if I'm honest, to be investigated properly to, to understand my ongoing symptoms, to make sure that you know.

0:3:11.560 --> 0:3:21.490
P005
You know, some of the symptoms are I still had a long COVID. It was nice to get further investigations done and understanding that actually it is all down to long cave and I'm not gonna be.

0:3:30.20 --> 0:3:30.490
PW (LC clinic)
OK.

0:3:34.360 --> 0:3:34.860
PW (LC clinic)
Yeah.

0:3:22.580 --> 0:3:38.290
P005
Need in any sort of like extra sort of like treatment. So for me I was, yeah, I was. I was quite relieved and I think working within the medical professional as well. I understand things a lot more. So I understood to be fair how poorly I was prior.

0:3:40.90 --> 0:3:41.470
P005
The having COVID and stuff.

0:3:40.390 --> 0:3:42.870
PW (LC clinic)
Prior to be being referred. Yeah. Yeah. OK.

0:3:42.290 --> 0:3:43.220
P005
Yeah. So.

0:3:43.770 --> 0:3:58.670
PW (LC clinic)
And was it just simply a process of, you know, you were just referred to long COVID, were you aware of, like, the long COVID clinic beforehand or, you know, a suppose essentially the question is how did you find out about the long COVID clinic was it was it purely through a referral process or OK.

0:3:57.360 --> 0:4:11.170
P005

Yeah. Yeah, it was. If if you're purely food like being being admitted into the Lister and being diagnosed with long COVID to say like, OK, we're gonna refer you to the long COVID cleaners. Like Ohh, didn't know one existed. I suppose that. Yeah, but no.

0:4:12.650 --> 0:4:12.990
P005
Yeah.

0:4:13.650 --> 0:4:13.970
P005
Yeah.

0:4:17.500 --> 0:4:18.460
P005
Yes. Yeah.

0:4:19.530 --> 0:4:20.700
P005
My admission, yeah.

0:4:9.360 --> 0:4:32.470
PW (LC clinic)
Right, right. Yeah, yeah. Why? In a way. Why? Why would you with such a new thing? Wasn't it, you know? And so you referred from the Lister. Would that be right? So after your unfortunate, you know, stay. Yeah. OK. OK. And again, Sh, sort of like a bit of a test of memory here because I don't know if you're my memory is pretty awful, if I'm honest with you. But so obviously thinking back to kind of like your first appointment, if that's.

0:4:33.490 --> 0:4:37.750
PW (LC clinic)
You know, if that's possible, you remember what the first appointment at the first appointment kind of involved?

0:4:38.440 --> 0:5:1.960
P005
Uh, yeah, I think it was. Initially it was a GP that had caught like a GP. That was a within that team called find out what ongoing symptoms I had. And one of the biggest ones for me was I was still having problems with shortness of breath. So after a discussion and sort of like it was obviously over the telephone and telephone assessment, she sort of said she wanted to refer me to like respiratory physio was my initial.

0:5:11.120 --> 0:5:11.550
PW (LC clinic)
OK.

0:5:2.620 --> 0:5:12.200
P005
And team to be referred to. So yeah, no, the GP that I used to speak to was was great. So yeah.

0:5:12.740 --> 0:5:17.20
PW (LC clinic)
And that was done. Umm, I'm presuming it was a virtual call, you know, like a.

0:5:19.700 --> 0:5:21.0
PW (LC clinic)
Over the telephone, OK.

0:5:16.280 --> 0:5:22.750
P005
It's a yeah, it's, uh. Tell just a normal telephone call. I don't think we've got. I don't think we've got to grips for teams at that point.

0:5:25.190 --> 0:5:33.780
PW (LC clinic)
And how was it? How was that, you know, like conducting, you know, that kind of appointment by telephone. How did you find that as a process?

0:5:33.960 --> 0:5:41.50
P005
Yeah, that don't bother me. I'm, you know, I'm not. I'm not one of these people that are fuss with its face to face teams or telephone.

0:5:42.370 --> 0:5:46.810
P005
But yeah, no, obviously again at that time, obviously COVID was still.

0:5:49.850 --> 0:5:50.180
PW (LC clinic)
Umm.

0:5:47.940 --> 0:5:59.190
P005
Pretty rife, so obviously the last thing I think I wanted and I you know any sort of clinician would want is to have a face to face appointment. So you know you sort of like knew that a lot of these appointments were gonna be.

0:6:2.0 --> 0:6:2.410
PW (LC clinic)
Yeah.

0:6:4.740 --> 0:6:5.240
PW (LC clinic)
Yeah.

0:6:0.430 --> 0:6:6.320
P005
So like virtual or over a telephone, you knew they weren't gonna be face to face. So that was that was fine with me.

0:6:6.980 --> 0:6:20.670
PW (LC clinic)
Umm. And you know, just in terms of preference, if you know going back to then it was it was possible would you have actually preferred a face to face appointment or you know in hindsight would would you was it was it quite quite happy with the way things were with it being kind?

0:6:20.140 --> 0:6:26.900
P005
I I suppose an issue with the GP1 probably wouldn't have been so much, maybe a little bit of a spiritual physio.

0:6:32.580 --> 0:6:32.960
PW (LC clinic)
Yeah.

0:6:44.10 --> 0:6:44.380
PW (LC clinic)
Yeah.

0:6:28.150 --> 0:6:50.280
P005
It might have been a bit easier face to face and then rather than teams, just because obviously you're, you know, you're not actually physically there in front of somebody and making sure you do everything right and getting a full understanding. But again it wasn't, it wasn't it, it was what it was. So I was as far as I was I said I was getting the help and support that I needed, so it didn't bother me.

0:6:50.530 --> 0:7:1.720
PW (LC clinic)
Yeah, and and and like, you know, up to the point of, you know, this, the outbreak of the pandemic, etcetera, how do you previously had any kind of health appointments that were carried out virtually or by telephone, etc.

0:7:1.670 --> 0:7:2.300
P005
Nope.

0:7:3.100 --> 0:7:5.190
P005
No, no. I was, yeah.

0:7:2.500 --> 0:7:8.90
PW (LC clinic)
No. OK. So it's quite a novel, quite novel situation. Novel way of delivering care. Yeah. OK.

0:7:6.90 --> 0:7:8.710
P005
Yeah, yeah, absolutely, yeah.

0:7:9.890 --> 0:7:22.490
PW (LC clinic)
And over the sort of like the your, your course of, yeah. You know, being sort of treated by the long COVID clinic. What was all of your appointments virtual? Were there any face to face interactions or?

0:7:21.630 --> 0:7:27.710
P005
My car, my cardiology ones, were face to face. Obviously I had to have scans on my.

0:7:35.840 --> 0:7:36.970
PW (LC clinic)
OK, OK.

0:7:28.910 --> 0:7:39.710
P005
My heart and I had to have a X-rays and scans on my lungs as well, so they will obviously face to face, but all the all the respiratory ones were all done virtually.

0:7:40.430 --> 0:7:47.180
PW (LC clinic)
OK, umm and and again you know just on while we're on that kind of you know that the process that you went through.

0:7:47.530 --> 0:7:59.410
PW (LC clinic)
Umm, so you know you can't really taught me through that Sh. You know like from going first of all your first appointment with the long COVID clinic and then how how things disseminate you know your path of care?

0:8:6.190 --> 0:8:6.570
P005
Yeah.

0:8:0.660 --> 0:8:7.660
PW (LC clinic)
Yeah. Do you wanna tell me a bit about how that, how that went from the moment that you, you know what what was undertaken, you know, what was the process that you went through?

0:8:7.790 --> 0:8:20.150
P005
Yes, I initially, obviously, like I say I was referred to the respiratory clinic cause of the main thing with my problem was I was still short of breath. I then started experiencing quite severe palpitations.

0:8:20.920 --> 0:8:34.980
P005
And so obviously spoke to the long COVID clinic and obviously you know I'm having an obviously I think again working in the professional world, it's a bit easier they I think they understand where I'm coming from. So obviously I explained to him about my palpitations.

0:9:2.190 --> 0:9:2.520
PW (LC clinic)
Right.

0:8:36.310 --> 0:9:5.230
P005
And yeah, obviously was referred to cardiology, no problems whatsoever. Really quick appointment. I suffered with fatigue quite a lot. So if I did anything, I'd then have to have maybe a couple of hours, like napping, because so I was referred to the fatigue clinic as well, which was done face to face. So I think every time I came to them with a issue within my long COVID problems, it was dealt with and there was a clinic available to me.

0:9:5.350 --> 0:9:5.990
P005
I think the.

0:9:7.410 --> 0:9:15.50
P005
The fatigue clinic took a little bit longer because they had a set of dates that I had to be able to attend all of them, and I think there was one.

0:9:16.890 --> 0:9:17.400
PW (LC clinic)
OK.

0:9:15.680 --> 0:9:27.460
P005
That I couldn't, uh. So that that was probably a little bit longer of a process, but again it it for me it wasn't the end of the world and so but.

0:9:25.770 --> 0:9:35.130
PW (LC clinic)
OK, OK. Yeah. And did you feel like along that route, you know, that everything that explained to you were aware of the pathway that you would that that you know the care pathway you go down that was all?

0:9:55.210 --> 0:9:55.390
PW (LC clinic)
Yeah.

0:9:56.560 --> 0:9:57.350
PW (LC clinic)
Sure. Yeah.

0:9:55.450 --> 0:10:7.960
P005
Compared to others, but yet no. At no point did I ever think I wasn't being like, listen to or looked after or supported. You know, I. Yeah, I can't fault it if I'm honest.

0:10:8.110 --> 0:10:25.590
PW (LC clinic)
Yeah. OK. Similar question, Sh, just the same sort of thing, but you know in terms of treatment and any treatment that you received, do you feel that that was kind of explained to you and that you were given options and you know you felt as if you were aware of what's going on and the treatments that you were you were?

0:10:26.590 --> 0:10:29.760
PW (LC clinic)
It was, you know, you were in the driving seat to say sort of, yeah.

0:10:28.670 --> 0:10:36.180
P005
Yeah. No, I was for, like, fully informed, fully understood. And and like I say, if it was something I wasn't, I.

0:10:36.820 --> 0:10:45.190
P005
If some I wasn't he keen on, then yeah, I I felt like I was listening to and it wasn't just a case of. Well, I'm telling you, you got to do this. You need to do it.

0:10:49.830 --> 0:10:52.80
PW (LC clinic)
Yeah, yeah.

0:10:46.370 --> 0:10:58.80
P005
They were, you know. Yeah, I I can't. Thought I need professional if I'm honest. I I really can't. I don't know. People might be quite negative about stuff, but I'm just. I'm just not. There's nothing negative to be.

0:10:59.200 --> 0:11:0.230
PW (LC clinic)
OK. Yeah.

0:10:59.120 --> 0:11:1.200
P005
About my process so.

0:11:0.920 --> 0:11:18.620
PW (LC clinic)
Fantastic. I mean, I'm. I'm kind of like can always kind of maybe guess the answer to this, but you know I will obviously ask you anyway you know with in terms of the diagnosis as well as the treatment that you received. Did you feel confident in you know what would you say sort of like?

0:11:25.360 --> 0:11:28.630
P005
Yeah. No. Yeah, yeah, definitely, definitely, definitely.

0:11:19.170 --> 0:11:33.450
PW (LC clinic)
Uh, that that they were that they were accurate and you felt confident in the process of diagnosis and and then subsequent treatment. Yeah. OK, OK. And just, you know, similar again similar kind of area but.

0:11:34.310 --> 0:11:44.850
PW (LC clinic)
Just in terms of kind of interactions with with staff and obviously clinic clinical staff, did you personally feel kind of supported within those interactions?

0:11:45.560 --> 0:11:51.870
P005
Yeah, yeah, definitely. Especially I think with like cardiology it that's obviously that was for me quite a worrying.

0:11:57.260 --> 0:11:58.180
PW (LC clinic)
Yeah, yeah.

0:12:3.730 --> 0:12:4.330
PW (LC clinic)
Absolutely.

0:12:15.90 --> 0:12:15.440
PW (LC clinic)
Yeah.

0:11:53.330 --> 0:12:18.140
P005
Process to go through cause obviously we don't. No, nobody wants anything be wrong with their hearts and you know, and I I was. I was, I was. I was. Yeah. Well, I still class. I was I was young at the time. Still I am young I suppose. And so actually I think that was that was quite a difficult time and like with history of heart problems within the family you know but it was they were all you always reassured always made aware of what was going on and.

0:12:19.60 --> 0:12:20.380
P005
You didn't wait.

0:12:32.860 --> 0:12:33.700
PW (LC clinic)
Fantastic.

0:12:21.510 --> 0:12:36.30
P005
Along time for any sort of like results, like when I had my echocardiogram, they were very good at saying, like, you know, we can't see anything wrong and it's just so, yeah, no, always fully informed and supported. So that was the biggest thing for me was for the cardiology one, I think.

0:12:36.700 --> 0:12:44.800
PW (LC clinic)
OK. And did you feel that, you know, you had the opportunity to voice any concerns that you might have had along the way? Yeah.

0:12:43.480 --> 0:13:1.40
P005
Yeah, definitely. Especially like along with the cardiology, definitely just due to some family history. I would you know, I made them aware. So I think they understood that my anxiety along though that particular area, I was probably a little bit higher than than other areas really.

0:13:1.690 --> 0:13:4.420
PW (LC clinic)
OK, OK and.

0:13:5.300 --> 0:13:9.820
PW (LC clinic)
You know, in terms of sort of like the staying in contact with with the clinic over a period of time.

0:13:10.50 --> 0:13:18.150
PW (LC clinic)

And you know, how long did you uh overall spend going through the clinical process, if that makes sense?

0:13:18.740 --> 0:13:19.800
P005
I think it was.

0:13:21.870 --> 0:13:25.320
P005
Maybe 77878 months.

0:13:27.760 --> 0:13:28.170
PW (LC clinic)
OK.

0:13:39.250 --> 0:13:39.580
PW (LC clinic)
Sure.

0:13:44.450 --> 0:13:44.880
PW (LC clinic)
Yeah.

0:13:26.370 --> 0:13:48.420
P005
I was. I was under them and and I think to towards the end it was one of those things of like they could have discharged me. But we were waiting on. We were waiting on a test result so she wanted to wait until we've had those test results before she discharged me. So again, she could just gone. Right. OK, that's it. Let's just finish it. But she she was quite adamant. No, let's just wait until we've had.

0:13:54.840 --> 0:13:54.980
PW (LC clinic)
Ah.

0:13:55.50 --> 0:13:55.250
PW (LC clinic)
Hi.

0:13:49.230 --> 0:13:57.300
P005
This particular test back and then I'll be able to discharge you if if I'm happy with it, so be it. But probably about 7 or 8 months bit in trouble.

0:14:5.210 --> 0:14:5.420
P005
Yeah.

0:14:8.20 --> 0:14:8.270
P005
Yep.

0:13:56.320 --> 0:14:8.970
PW (LC clinic)
Several months and and you've touched on this a bit, Sh, but just don't like he's clear in my head. Obviously it was you had some help with fatigue, so you had you know that you the fatigue clinic also cardiology. Were there any other?

0:14:10.310 --> 0:14:10.530
PW (LC clinic)
The.

0:14:10.270 --> 0:14:12.120
P005
But I like this respiratory.

0:14:12.290 --> 0:14:13.180
PW (LC clinic)
Respiratory yeah.

0:14:12.280 --> 0:14:14.150
P005
And I think.

0:14:14.880 --> 0:14:21.790
P005
So that was it for me. I might. Well, I had. I had to have a lung function test as well, which I don't I that that might come under.

0:14:22.570 --> 0:14:27.430
P005
The spiritual aspect? Maybe. So. Yeah. Yeah, so.

0:14:22.250 --> 0:14:33.740
PW (LC clinic)
Is that might come out of respiratory pulp, pulmonary rehab, something like that. Yeah. OK. And. And, you know, I know again, this might be tricky because you've probably had quite a lot of tests etcetera, but you know.

0:14:34.910 --> 0:14:40.560
PW (LC clinic)
Ones that you remember what? What assessments and treatments did you receive kind of in?

0:14:39.900 --> 0:14:53.900
P005
Uh, so, so we cardiology, I had an obviously 24 hour ECG monitoring. I had an echo Cardiogram and then I had a lung function test on my lungs.

0:15:2.0 --> 0:15:2.340
PW (LC clinic)
OK.

0:14:54.640 --> 0:15:4.370
P005

And then with the fatigue clinic, it was mainly to do with sort of like under the respiratory physio was all to do with more exercise type things and but.

0:15:5.170 --> 0:15:14.510
P005
Yeah, lots of CT scans, lots of xrays and things. But yeah, mainly like, say, it was the echocardiogram. I think was probably the biggest thing.

0:15:15.70 --> 0:15:26.680
PW (LC clinic)
OK. OK. And in terms of like regularity and again, I guess it was probably quite sporadic, but you know how often would you say that you were in contact with the clinic throughout that process?

0:15:27.600 --> 0:15:28.260
P005
Umm.

0:15:29.430 --> 0:15:33.970
P005
Probably with with the the GP I think you should call me at least once a month I think.

0:15:34.170 --> 0:15:34.620
PW (LC clinic)
OK.

0:15:35.240 --> 0:15:44.720
P005
I mean it would be random call I I they would just call me and I'd be like, oh, hello. You know, I was never. I was not ever told they were gonna call me. It was just one of those.

0:15:44.170 --> 0:15:47.570
PW (LC clinic)
OK, just to check up, just to see how, yeah.

0:15:45.420 --> 0:15:55.910
P005
Uh, yeah, yeah, yeah. I used to be like ohh, you know? Ohh. Hello. They'd be like, oh, yeah, GP from the long COVID clinic. So I was never sent an appointment to speak to the GP. It was just always a.

0:15:56.780 --> 0:15:57.400
P005
A random.

0:15:58.120 --> 0:15:59.600
P005
Out of the blue? Cool. Really.

0:16:0.380 --> 0:16:0.750
P005
Yeah.

0:15:57.920 --> 0:16:2.370
PW (LC clinic)
Right, right out of the blue was it? Was it always the same member of staff or?

0:16:2.160 --> 0:16:3.430
P005
Yeah, it was always GP.

0:16:3.320 --> 0:16:4.110
PW (LC clinic)
OK, OK.

0:16:4.370 --> 0:16:4.710
P005
Yeah.

0:16:11.910 --> 0:16:12.170
P005
Hi.

0:16:6.820 --> 0:16:24.110
PW (LC clinic)
And yeah, go. Sorry, I just, I'm sort of like a darting about of it here, Sh. So I do apologize, but you know, going back to the the day of your first appointment with the clinic and if it's possible to say how are you feeling on that particular day knowing that you were having your first appointment?

0:16:29.370 --> 0:16:29.690
PW (LC clinic)
Hmm.

0:16:25.60 --> 0:16:30.750
P005
Umm, I was. I was. I always think it was quite straight. It was quite stressful. I was still at that point.

0:16:42.920 --> 0:16:43.240
PW (LC clinic)
Umm.

0:16:31.800 --> 0:16:44.920
P005
Unwell. I'm still having quite a lot to deal with. The fact that I'd been so poorly that you sort of come out the other side of it where you know that there was thousands of thousands of people dying from it. So there was a lot of anxiety and apprehension.

0:16:45.50 --> 0:16:47.100
P005
And but again.

0:16:48.200 --> 0:16:50.430
P005
I knew it could only benefit me rather than.

0:16:52.710 --> 0:16:53.120
PW (LC clinic)
Yeah.

0:16:51.580 --> 0:16:57.220
P005
It not benefited me. I knew big, you know, being referred and speaking to the clinic was only gonna help me rather than.

0:17:0.270 --> 0:17:0.640
PW (LC clinic)
Yeah.

0:17:4.520 --> 0:17:5.590
PW (LC clinic)
Absolutely, yeah.

0:16:57.900 --> 0:17:7.500
P005
But yeah, I was nervous and anxious. Cause suppose it's. Yeah, it was. It was scary territory at the time because we did we we just didn't know enough about it.

0:17:8.380 --> 0:17:8.850
P005
Die.

0:17:7.770 --> 0:17:17.960
PW (LC clinic)
Yeah, absolutely. Yeah, it wasn't it. UM, and if it's possible to say, like, approximately how long do you think you spent with on that first appointment? Speaking to that, that first member of staff?

0:17:17.690 --> 0:17:20.940
P005
Uh, probably. At least a good like 45 minutes to an hour.

0:17:21.600 --> 0:17:21.930
P005
Yeah.

0:17:20.740 --> 0:17:34.960
PW (LC clinic)
5 minutes. OK and then again, Shannon, I totally appreciate this probably quite difficult questions because it's it is a long time ago. But jimber how you felt post to you know after the after you had the appointment, were there any changes in the way that you felt say compared to prior?

0:17:38.620 --> 0:17:38.910
PW (LC clinic)
Hmm.

0:17:35.580 --> 0:17:39.240
P005
Well, I I think again it's it's just that reassurance that actually.

0:17:40.730 --> 0:17:49.530
P005
It's not just something that's going to be brushed under the carpet that actually, and I suppose at the end of the day we we needed to find out more about it. The only way we could do that was by.

0:17:56.530 --> 0:17:56.930
PW (LC clinic)
Yeah.

0:17:50.520 --> 0:18:2.770
P005
Developing these long COVID clinics that actually you can then monitor that person, post COVID. And so yeah, no, I was I I was, yeah, I was quite happy that I was still being cared for and I was still.

0:18:10.660 --> 0:18:10.950
PW (LC clinic)
Yeah.

0:18:4.30 --> 0:18:11.490
P005
In the pipeline of get getting getting help and getting things checked out fully. So now I was yeah I was.

0:18:12.180 --> 0:18:13.660
P005
Relieved and happy if I'm honest.

0:18:13.820 --> 0:18:15.100
PW (LC clinic)
Yeah. OK. OK.

0:18:15.980 --> 0:18:28.330
PW (LC clinic)
And we've, we've definitely touched on this, but I I will ask you anyway. Just you know, for purposes of clarity, but you know where you satisfied with the care that you received and if so, why?

0:18:28.600 --> 0:18:33.790
P005
Yeah, yeah, 100 hundred 100% satisfied like I say.

0:18:34.810 --> 0:18:40.490
P005
Just because it was such a new virus that we knew nothing about, it was just nice to know.

0:18:41.570 --> 0:18:49.720
P005
That basically I'd had probably the best MOT in my life compared to you know, if I hadn't had cut, you know, there was just, it was I just felt like.

0:18:51.540 --> 0:18:55.740
P005
Nothing else could have could have been investigated. I I felt like I'd be fully.

0:18:59.670 --> 0:19:0.130
PW (LC clinic)
Yeah.

0:19:1.520 --> 0:19:2.230
PW (LC clinic)
Yeah, yeah.

0:18:56.400 --> 0:19:4.260
P005
Yeah, the probably like, say best body MINTER tab for my you know, so yes and I I ohr can't fault it really.

0:19:4.410 --> 0:19:13.370
PW (LC clinic)
Yeah. So you felt quite it made with those sort of you know checks being carried out you I guess would be fair to say you felt quite confident as a result of those those, yeah.

0:19:12.60 --> 0:19:18.410
P005
Yeah, I felt confident. Felt felt relieved that actually, you know what, I actually you do then think, OK, well, that's fine. It's not a.

0:19:19.570 --> 0:19:21.610
P005
So it's gonna be the rest of my life.

0:19:21.730 --> 0:19:30.20
P005
And you know, yes, OK. I still do have certain problems now, but that, that that is what it is. But I know I'll ultimately.

0:19:32.550 --> 0:19:32.880
PW (LC clinic)
Yeah.

0:19:33.600 --> 0:19:33.920
PW (LC clinic)
Yeah.

0:19:30.630 --> 0:19:36.980
P005
There's no underlying issues that are gonna, you know, everything's everybody's been looked at. So yeah, no. Yeah.

0:19:35.790 --> 0:19:37.400
PW (LC clinic)
Yeah. OK. OK.

0:19:38.540 --> 0:19:41.430
PW (LC clinic)
And you know, thinking about sort of.

0:19:42.500 --> 0:19:51.610
PW (LC clinic)
I suppose what would you say sort of moving towards sort of like the idea of like an ideal if it was ideal in your mind? You know, is there anything that you think could have been better?

0:19:55.620 --> 0:19:56.400
PW (LC clinic)
Yeah. OK.

0:19:56.460 --> 0:20:9.710
P005
I I think, yeah. No, I like say I can't. I can't. I can't fault anything, really. Every staff member were was caring. Kind were, you know, listened. And yeah, we're supportive. So yeah. Now I can't.

0:20:10.690 --> 0:20:11.130
PW (LC clinic)
OK.

0:20:10.760 --> 0:20:12.900
P005
I can't think of anything. If anything, they've changed.

0:20:13.340 --> 0:20:30.700
PW (LC clinic)
Sure. OK. And even in terms of say, you know the way that things were delivered, say like the, you know the virtual aspects of it, you know anything that you would you would change there if you know giving an ideal set of circumstances you know would there been a preference for say face to face appointments over telephone appointments or?

0:20:31.720 --> 0:20:32.90
PW (LC clinic)
Umm.

0:20:36.920 --> 0:20:37.270
PW (LC clinic)
Yeah.

0:20:29.740 --> 0:20:49.240
P005
I think there's always gonna be a preference for face to face because I think that it gives a very different sense of the appointment and but I obviously again, you understand the complications of that because of the virus we were we were experiencing. So yeah, I think face to face appointments are always gonna be better than.

0:20:51.460 --> 0:20:51.930
PW (LC clinic)
Yeah.

0:20:49.910 --> 0:20:53.420
P005
That a virtual one, but it it also just wasn't the end of the world either.

0:20:58.520 --> 0:20:58.820
P005
Yeah.

0:21:1.350 --> 0:21:2.650
P005
Yeah, absolutely.

0:21:3.390 --> 0:21:3.680
P005
Yeah.

0:20:53.640 --> 0:21:10.0
PW (LC clinic)
Yeah. So just you know, under the circumstances, that's what was safest and you know and, and I suppose possible as well and you know, yeah, OK well actually Sh, we've gone through all of the questions I can ask you anything that we touched on that you wanted to expand on.

0:21:10.240 --> 0:21:12.90
PW (LC clinic)
And in any way or.

0:21:18.150 --> 0:21:18.500
PW (LC clinic)
Ohr.

0:21:11.130 --> 0:21:22.770
P005
No, no, I thought. No, I like I say I just I'm. I'm very grateful for the long COVID clinic and all the staff that works works within that team and yeah, yeah.

0:21:26.770 --> 0:21:27.90
P005
Yeah.

0:21:28.240 --> 0:21:28.640
P005
Yeah.

0:21:22.170 --> 0:21:35.0
PW (LC clinic)
It's. Yeah, it's so good that you, you know, I can see that you've had a a positive experience of. That's. That's great, isn't it? And final question, is there any other comments that you wanted to mention Sh before I stop the recording?

0:21:35.310 --> 0:21:43.370
P005
Now I've just say thank. Thank thank you to the team and thanks for all their hard work. You know it was a it was a very trying.

0:21:47.260 --> 0:21:47.670
PW (LC clinic)
Yeah.

0:21:56.220 --> 0:21:56.860
PW (LC clinic)
That's fantastic.

0:21:59.80 --> 0:21:59.880
PW (LC clinic)
Ohr blessing.

0:21:44.80 --> 0:22:4.980
P005
Time for everybody that worked within the night. It was within NHS and you know, I suppose at the end that I couldn't be prouder that the to to work for the NHS or that, you know, at this that particular time. So yeah, and I really grateful to to everybody really and to the list of hospital for saving my life really because, you know, it could have gone the other way. So yeah, very lucky.

0:22:6.300 --> 0:22:6.740
P005
Yeah.

0:22:8.450 --> 0:22:9.0
P005
Not covered.

0:22:4.540 --> 0:22:10.20
PW (LC clinic)
Yeah. What, what an experience? Yeah, what? Sh. Thank you ever so much. I what I'll do here is just.

0:22:11.960 --> 0:22:14.640
PW (LC clinic)
… stop the recording.

-- ----- ----- -----

P008

0:0:0.0 --> 0:0:0.740
P008
Yes, it does.

0:0:-2.-980 --> 0:0:1.500
PW (LC clinic)
If it says recording, et cetera at your end, so it isn't and it does.

0:0:0.860 --> 0:0:3.980
P008
There's someone something. Transcription.

0:0:4.370 --> 0:0:5.60
PW (LC clinic)
OK.

0:0:4.310 --> 0:0:5.900
P008
Yes, totally, yeah.

0:0:5.380 --> 0:0:7.170
PW (LC clinic)
UM, perfect.

0:0:7.180 --> 0:0:7.580
PW (LC clinic)
OK.

0:0:7.590 --> 0:0:14.700
PW (LC clinic)
So just for the purpose of the tape today, I'm here with participant P008.

0:0:15.10 --> 0:0:20.640
PW (LC clinic)
It's the 11th of May, 2023 and the time is 5 minutes past 11.

0:0:21.0 --> 0:0:26.930
PW (LC clinic)
And again, just for the purposes of the recording, S, are you happy for me to be recording this conversation today?

0:0:27.610 --> 0:0:28.440
P008
I am happy, yes.

0:0:29.190 --> 0:0:29.500
PW (LC clinic)
Lovely.

0:0:29.510 --> 0:0:30.560
PW (LC clinic)
Thank you very much.

0:0:31.170 --> 0:0:35.80
PW (LC clinic)
OK, so you know, we'll start with that.

0:0:35.430 --> 0:0:42.50
PW (LC clinic)
I'd say kind of a rather broad question, but what was your experience like of being referred to the long COVID clinic?

0:0:43.710 --> 0:0:47.30
P008
I'm from what I can remember, there's a lot of it.

0:0:51.490 --> 0:0:51.770
PW (LC clinic)
OK.

0:0:47.40 --> 0:0:52.170
P008
I'll be honest with my memory issues, I I can't remember, but I do remember.

0:0:53.830 --> 0:0:59.750
P008
The we did it, it was mostly done on the phone and then I think I did have a teams.

0:1:1.490 --> 0:1:4.120
P008
Umm, what's the word appointment as well?

0:1:4.830 --> 0:1:5.40
PW (LC clinic)
Yeah.

0:1:4.310 --> 0:1:9.970
P008
And they also came out emails to me as well, which was, which was really good.

0:1:11.290 --> 0:1:11.620
PW (LC clinic)
OK.

0:1:11.630 --> 0:1:14.920
PW (LC clinic)
So you received sort of like information from them.

0:1:14.930 --> 0:1:17.820
PW (LC clinic)
It could have been, you know, across all those different mediums.

0:1:17.830 --> 0:1:19.400
PW (LC clinic)
So telephone, email.

0:1:19.700 --> 0:1:19.840
P008
Yeah.

0:1:19.590 --> 0:1:23.460
PW (LC clinic)
And did you mention did you have virtual appointments as well or?

0:1:28.910 --> 0:1:29.130
PW (LC clinic)
Yeah.

0:1:24.170 --> 0:1:31.570
P008
But did I think I can only remember 1 and that would just to look to see?

0:1:34.560 --> 0:1:39.550
P008
To look at if I was to say, if you breathe, I think it's like breathing from the stomach and things like that.

0:1:39.740 --> 0:1:40.320
PW (LC clinic)
OK.

0:1:39.660 --> 0:1:43.10
P008
And to check the model that I was that I was breathing.

0:1:43.60 --> 0:1:45.880
P008
So yeah, I I can just about remember that.

0:1:46.900 --> 0:1:47.910
PW (LC clinic)
OK, OK.

0:1:48.370 --> 0:1:52.310
PW (LC clinic)
And just into like so you know, think about like saying the first appointment.

0:1:52.320 --> 0:2:1.940
PW (LC clinic)
Is it possible to say I know S, you say you know part of the long COVID symptoms for you is obviously been this and you know, problems with the memory which you also just mentioned.

0:2:1.950 --> 0:2:7.150
PW (LC clinic)
So, umm, you know, if it's not possible to remember these these things then you know please don't worry.

0:2:7.560 --> 0:2:15.100
PW (LC clinic)
But you know, thinking back to your first appointment can remember whether it was it easy to get your first appointment or was it something that was quite difficult to get?

0:2:17.650 --> 0:2:23.810
P008
To be honest, I'm not quite sure how I was referred.

0:2:24.580 --> 0:2:24.960
PW (LC clinic)
OK.

0:2:28.790 --> 0:2:29.30
PW (LC clinic)
Yeah.

0:2:25.260 --> 0:2:34.480
P008
I don't know if it was through my GP or cause I was under quite a few departments and I was under respiratory.

0:2:34.490 --> 0:2:39.630
P008
I was under hematology and antiquariat and things like that.

0:2:39.790 --> 0:2:41.360
P008
So it's pot.

0:2:41.370 --> 0:2:43.400
P008
I'm not sure where it came from.

0:2:43.650 --> 0:2:44.300
P008
I don't.

0:2:44.310 --> 0:2:46.480
P008
I think I remember well, I'm.

0:2:46.490 --> 0:2:52.770
P008
I'm gonna assume cause I don't remember, but I'm gonna assume that I was just advised that this is.

0:2:53.210 --> 0:2:54.740
P008
This is what we've done for you.

0:2:55.490 --> 0:2:55.930
PW (LC clinic)
OK.

0:2:54.750 --> 0:2:58.320
P008
Someone would have advised me is this is what's gonna happen next.

0:2:59.0 --> 0:2:59.260
PW (LC clinic)
OK.

0:2:59.270 --> 0:3:1.290
PW (LC clinic)
And you think that was probably most likely your GP?

0:3:3.220 --> 0:3:4.730
P008
Possibly.

0:3:5.170 --> 0:3:5.430
PW (LC clinic)
Yeah.

0:3:4.740 --> 0:3:13.30
P008
I'm, as I say, I had contact with, you know, a sort of the the 18 months of it.

0:3:13.40 --> 0:3:23.490
P008
So the 1st 18 months following having COVID and I had a lot of medical contact, so it's really hard to say, but possibly my GP.

0:3:23.920 --> 0:3:24.690
PW (LC clinic)
OK.

0:3:25.420 --> 0:3:30.990
PW (LC clinic)
And you know, do you remember it taking, you know, a a length of time to get that first appointment?

0:3:32.970 --> 0:3:33.210
P008
Umm.

0:3:31.0 --> 0:3:33.570
PW (LC clinic)
You know, was it was it fairly quick or was it something that you use?

0:3:34.760 --> 0:3:40.770
P008
I actually think I don't know the issue I've got with my memory is I'm actually waiting.

0:3:40.780 --> 0:3:41.850
P008
I've had a two month.

0:3:45.740 --> 0:3:45.980
PW (LC clinic)
Hmm.

0:3:41.860 --> 0:3:49.720
P008
Well, I've been being assessed over a period of about a year for possible earnings days, dementia, so as well as the memory loss.

0:3:49.100 --> 0:3:50.120
PW (LC clinic)
I'm sorry to hear that, S.

0:3:50.680 --> 0:3:51.750
P008
That's OK.

0:3:51.840 --> 0:3:59.150
P008

I'm I've had all my assessments done and I'm just writing them to put a diagnosis together now, but I have a feeling I'm gonna.

0:3:59.160 --> 0:4:6.350
P008
I'm gonna say that I think somebody probably called me and said you've been referred to this service.

0:4:7.290 --> 0:4:8.220
PW (LC clinic)
OK, OK.

0:4:12.890 --> 0:4:13.70
P008
Yeah.

0:4:8.230 --> 0:4:16.920
PW (LC clinic)
So the yeah, that sounds like a you, you know, if you were referred and maybe not aware that you've been referred then it was it kind of a you wouldn't have a delay.

0:4:15.350 --> 0:4:17.750
P008
Probably not, no.

0:4:16.930 --> 0:4:18.820
PW (LC clinic)
You know, you wouldn't have any sort of like a waiting period.

0:4:18.830 --> 0:4:18.970
PW (LC clinic)
OK.

0:4:18.980 --> 0:4:19.600
PW (LC clinic)
That makes sense.

0:4:19.610 --> 0:4:20.570
PW (LC clinic)
Yeah. OK.

0:4:20.10 --> 0:4:28.390
P008
Yeah, because I get a lot of things that, umm, I would get called in for appointments for lots of stuff that I had no idea about.

0:4:28.460 --> 0:4:29.760
P008
So I'm I'm gonna.

0:4:29.770 --> 0:4:34.560
P008
I'm gonna say that probably someone called me to say we're from the pulmonary rehab team.

0:4:34.810 --> 0:4:36.300
P008
This is what we're gonna be doing with you.

0:4:36.880 --> 0:4:38.170
PW (LC clinic)
I'm with you, OK?

0:4:38.890 --> 0:4:46.750
PW (LC clinic)
And you know, and again I totally appreciate that I'm asking you to kind of recall things, S, I know that that's something you're you're you're having trouble with.

0:4:50.640 --> 0:4:50.800
P008
Yeah.

0:4:46.760 --> 0:4:50.810
PW (LC clinic)
So you know, if it's not possible to answer, you know, please don't worry at all.

0:4:50.820 --> 0:4:54.230
PW (LC clinic)
But I just just wondered sort of if it's possible to.

0:4:56.570 --> 0:5:3.840
PW (LC clinic)
You know, in terms of like the first appointment that you had, I are you aware of sort of like what was involved at that first appointment?

0:5:4.670 --> 0:5:6.460
PW (LC clinic)
You know how it was conducted?

0:5:6.710 --> 0:5:9.500
PW (LC clinic)
Maybe who conducted and and and kind of how you felt on the day?

0:5:10.250 --> 0:5:19.30
P008
Umm, all I can remember is is it was a lady and and she was very nice.

0:5:21.290 --> 0:5:24.990
P008
And she, from what I can remember, I think she might have been called Isabel.

0:5:26.190 --> 0:5:26.570
PW (LC clinic)
OK.

0:5:25.0 --> 0:5:27.730
P008
But I'm not sure she she was.

0:5:31.80 --> 0:5:34.580
P008
She I think I can sort of widely remember she was very good.

0:5:34.590 --> 0:5:37.470
P008
Like she got even though no one really sort of knew.

0:5:39.900 --> 0:5:40.160
PW (LC clinic)
Hmm.

0:5:37.480 --> 0:5:40.790
P008
This was obviously quite knew she's sort of.

0:5:41.160 --> 0:5:44.470
P008
You could tell think she knew what she was doing, and it was.

0:5:44.480 --> 0:5:48.380
P008
She'd already got black a plan, a basic plan plan put together and stuff like that.

0:5:49.200 --> 0:5:49.760
PW (LC clinic)
OK.

0:5:49.830 --> 0:5:50.690
PW (LC clinic)
And did you did?

0:5:50.700 --> 0:5:53.40
PW (LC clinic)
Did you feel sort of like that you were included?

0:5:53.930 --> 0:5:56.120
P008
Yes. Yeah.

0:5:53.770 --> 0:5:59.730
PW (LC clinic)
You know, in terms of the plan did and did you feel as if you had an opportunity to kind of ask questions and and things like that?

0:5:59.650 --> 0:6:1.790
P008
Yeah, I think I did, yeah.

0:6:0.940 --> 0:6:3.370
PW (LC clinic)
So OK.

0:6:3.630 --> 0:6:8.770
PW (LC clinic)
And you know, were you given any sort of treatment options at that and uh S?

0:6:9.820 --> 0:6:12.430
P008
I can't remember.

0:6:13.270 --> 0:6:13.960
PW (LC clinic)
OK.

0:6:14.460 --> 0:6:14.730
P008
Umm.

0:6:14.250 --> 0:6:23.180
PW (LC clinic)
And did you, did you know, do you have any sort of sense of how you felt at the time, you know, just the fact that you you were getting this help with with long COVID?

0:6:23.310 --> 0:6:24.170
PW (LC clinic)
How did that make you feel?

0:6:25.280 --> 0:6:27.350
P008
Umm, I would imagine.

0:6:27.360 --> 0:6:36.740
P008
Probably very good because up until that point, everybody was very much in the dark as to what was going on.

0:6:38.70 --> 0:6:38.290
PW (LC clinic)
Yeah.

0:6:39.880 --> 0:6:41.720
P008
So I would say, yeah.

0:6:40.0 --> 0:6:43.280
PW (LC clinic)
So, so yeah and.

0:6:45.380 --> 0:6:49.430
PW (LC clinic)
And this this, this lady that we think they might have been Isabel and.

0:6:49.500 --> 0:6:51.830
P008
The thing that that night 8 it's funny.

0:6:51.840 --> 0:6:53.590
P008
So I can't remember.

0:6:53.860 --> 0:6:55.340
P008
There's lots of things I can't remember.

0:6:55.350 --> 0:6:58.130
P008
What I can remember really random things, so yeah.

0:6:59.470 --> 0:7:2.170
PW (LC clinic)
And do we know if she was a GP or what?

0:7:2.180 --> 0:7:2.360
PW (LC clinic)
What?

0:7:2.370 --> 0:7:4.410
PW (LC clinic)
Her background was or were no OK.

0:7:2.830 --> 0:7:4.540
P008
No shape.

0:7:4.550 --> 0:7:19.790
P008
My think she was umm to do with the pulmonary rehab and she's like the I don't know if she'd have been and like her or what's the words.

0:7:19.800 --> 0:7:22.130
P008
UM, like a physiotherapist, like a lung?

0:7:22.240 --> 0:7:22.680
PW (LC clinic)
OK.

0:7:23.760 --> 0:7:24.830
PW (LC clinic)
Yes, that makes sense.

0:7:22.140 --> 0:7:26.300
P008
Physiotherapy, I think something like that.

0:7:27.450 --> 0:7:28.140
PW (LC clinic)
OK.

0:7:28.730 --> 0:7:40.400
PW (LC clinic)
And again, just like in terms of you know rather than sort of recalling sort of you know, what would you say sort of you know the individuals involved, do you remember sort of how you felt at that first appointment, S?

0:7:41.230 --> 0:7:42.750
P008
I don't know, no.

0:7:42.750 --> 0:7:43.220
PW (LC clinic)
No.

0:7:43.390 --> 0:7:43.690
PW (LC clinic)
OK.

0:7:44.250 --> 0:7:58.80
PW (LC clinic)
And and how about sort of like you know, just rather than sort of the initial appointment, I'm just wondering about how things went and you know how often would you say you were in contact with the clinic as time went on and you?

0:7:58.800 --> 0:8:3.260
PW (LC clinic)
Uh, you you received sort of care for for the long COVID?

0:8:9.240 --> 0:8:9.990
PW (LC clinic)
OK.

0:8:2.910 --> 0:8:11.190
P008
Then it might have been once a wep003, or every two wep003s then.

0:8:10.200 --> 0:8:11.630
PW (LC clinic)
And would that normally be?

0:8:11.740 --> 0:8:14.910
PW (LC clinic)
Would that normally be with the same member of staff or could it have been different members of staff?

0:8:15.730 --> 0:8:24.480
P008
I feel like it was the same member of staff because as well I'd actually we're not still not quite sure to this day how I managed it.

0:8:28.10 --> 0:8:28.380
PW (LC clinic)
Right.

0:8:24.670 --> 0:8:37.290
P008
I'd actually gone back to work at this point as well, which was a bit of a daft thing to do, so probably I might well have had like appointment, perhaps in my lunch hour over the phone and things like that.

0:8:37.760 --> 0:8:38.710
PW (LC clinic)
OK. OK.

0:8:38.720 --> 0:8:42.330
PW (LC clinic)
Yeah, that makes sense and kind of in its entirety, S.

0:8:42.500 --> 0:8:43.270
PW (LC clinic)
How how long?

0:8:43.280 --> 0:8:51.90
PW (LC clinic)
Approximately, you know, it's only gonna be a rough amount of time, but how long would you say you were in contact with the long COVID clinic from start to end?

0:8:52.110 --> 0:8:54.200
P008
Umm, I think it might have been.

0:8:57.220 --> 0:8:59.400
P008
Ah, might be a couple of months.

0:9:0.400 --> 0:9:1.430
PW (LC clinic)
Couple of months. OK.

0:9:1.440 --> 0:9:1.640
PW (LC clinic)
Yeah.

0:9:1.970 --> 0:9:2.370
P008
Umm.

0:9:2.120 --> 0:9:4.990
PW (LC clinic)
And and you know a a kind of toughness.

0:9:8.130 --> 0:9:8.350
P008
Yeah.

0:9:5.0 --> 0:9:9.110
PW (LC clinic)
And if it again, you know, and if if if you can't, you know, recall it.

0:9:9.120 --> 0:9:9.990
PW (LC clinic)
That's no problem at all.

0:9:10.60 --> 0:9:16.200
PW (LC clinic)
I just wondered, you know, if you're aware of, like, what assessments you might have been given or and what and what treatments you were given over that period of time?

0:9:17.280 --> 0:9:22.810
P008
I do remember assessment wise I'm not too short.

0:9:31.120 --> 0:9:31.350
PW (LC clinic)
Yeah.

0:9:22.820 --> 0:9:37.820
P008
Maybe it would have been how far I could walk and things like that, which wasn't particularly far and still isn't particularly for, but I do remember I was given a lot of, like, treatment wise.

0:9:42.950 --> 0:9:43.250
PW (LC clinic)
OK.

0:9:37.890 --> 0:9:47.310
P008
It was mainly like exercises and tips and things like that and I'm the exercises because I used to do them sitting at my desk.

0:9:48.310 --> 0:9:48.710
PW (LC clinic)
Right.

0:9:48.320 --> 0:9:51.570
P008
Umm and I used to do it to try and distract my colleague as well.

0:9:51.580 --> 0:9:57.210
P008
I was sitting next to me and that I should remember that quite clearly, and it was they sent me out.

0:9:58.210 --> 0:9:59.820
P008
I've probably still got the email.

0:9:59.830 --> 0:10:11.870
P008
Actually, I do remember quite vividly being sent out emails and and it was things like breathing like through your stomach, like down through your chest and right into your stomach.

0:10:25.370 --> 0:10:25.880
PW (LC clinic)
OK.

0:10:25.890 --> 0:10:28.260
PW (LC clinic)
So yeah.

0:10:11.980 --> 0:10:34.850
P008
Things like when you're breathing, if you're having trouble breathing, to breathe with your mouth closed rather than open, and to do slower breaths and quieter breaths until things like that, it was kind of almost having to learn how to breathe again, because I'd had COVID and I'd had pneumonia as well.

0:10:34.980 --> 0:10:43.700
P008
On top of that, I've got asthma, so I think it was literally almost teaching me how to breathe again, which you think sounds really weird.

0:10:43.710 --> 0:10:44.30
P008
You think?

0:10:44.330 --> 0:10:44.690
PW (LC clinic)
Umm.

0:10:44.40 --> 0:10:53.220
P008
Well, I can breathe or do it everyday, but actually it what they said to me made a lot of treatment wise made a lot of sense.

0:10:53.930 --> 0:10:54.870
PW (LC clinic)
OK, did did.

0:10:54.880 --> 0:10:57.260
PW (LC clinic)
Did you feel that that worked?

0:10:57.270 --> 0:11:0.910
PW (LC clinic)
You know, do you feel confident in the treatment that that it was having some impact?

0:10:59.520 --> 0:11:6.670
P008
Yeah, I think because if I had trouble with my breeze in which I do sort of on a regular basis.

0:11:6.840 --> 0:11:10.770
P008
I still I can still remember those those key points.

0:11:14.260 --> 0:11:14.560
PW (LC clinic)
OK.

0:11:10.780 --> 0:11:24.770
P008
Now that's one of the things that has actually stayed with me, and I've it's been for all the sort of confusion and that it's still does does stay with me and it's, you know, it's our find it really useful.

0:11:28.910 --> 0:11:29.240
P008
Umm.

0:11:31.540 --> 0:11:32.530
P008
Yeah, to the side.

0:11:26.600 --> 0:11:36.640
PW (LC clinic)
That well, that, that, that's fantastic and and and and as you say you still use that you know from time to time still yeah, fantastic so you know.

0:11:38.280 --> 0:11:46.750
PW (LC clinic)
Again, in relation to kind of your contact with the Connect, did you feel supported at you know when you had interactions you did, yeah.

0:11:43.970 --> 0:11:47.960
P008
Yeah, it's not like, yeah, yeah.

0:11:47.80 --> 0:11:51.900
PW (LC clinic)
And was there anything in particular that made you feel supported that, you know, thinking back?

0:11:51.910 --> 0:11:55.970
P008
And the lady was just a sent.

0:11:55.980 --> 0:11:58.310
P008
Remember, being easy to talk to, but she was also.

0:11:59.330 --> 0:12:8.900
P008
UM, she was quite a no nonsense lady and I liked that she was very sort of direct and to the point and she knew what she was doing.

0:12:9.30 --> 0:12:11.300
P008
And you felt that this was somebody.

0:12:12.10 --> 0:12:12.660
P008
OK.

0:12:12.670 --> 0:12:15.460
P008
In amongst all of this craziness and madness.

0:12:15.990 --> 0:12:18.20
P008
Here's somebody that actually knows what you know.

0:12:25.290 --> 0:12:26.310
PW (LC clinic)
Right, right.

0:12:18.570 --> 0:12:31.140
P008
Someones got a bit of an idea of what's going on because she'd obviously, I'm guessing, drawn on her previous backgrounds from staff and yeah, yeah.

0:12:27.230 --> 0:12:33.370
PW (LC clinic)
So just having someone that was quite straightforward and gave you that gave you a sense of confidence around.

0:12:33.670 --> 0:12:34.450
P008
8 days?

0:12:34.690 --> 0:12:35.240
PW (LC clinic)
Yeah.

0:12:34.510 --> 0:12:36.70
P008
Yeah, in.

0:12:35.290 --> 0:12:36.150
PW (LC clinic)
OK, OK.

0:12:36.870 --> 0:12:37.280
PW (LC clinic)
Umm.

0:12:37.430 --> 0:12:40.330
PW (LC clinic)
And you know, you might not have had any, and that would.

0:12:40.340 --> 0:12:42.300
PW (LC clinic)
That's obviously absolutely fine, S.

0:12:42.310 --> 0:12:50.70
PW (LC clinic)
And did you feel able to voice any concerns about your diagnosis or or or, you know, in relation to the to the treatments that you were given?

0:13:5.690 --> 0:13:5.970
PW (LC clinic)
Hmm.

0:12:51.30 --> 0:13:10.710
P008
Umm, diagnosis was I don't really remember if I did voice any concerns because it was still so new and with the treatment I was given, I sort of didn't have any concerns with with thought there was, you know, I wasn't sort of concerned with that in, you know, in any way.

0:13:10.720 --> 0:13:11.450
P008
So it was.

0:13:11.780 --> 0:13:14.210
P008
It was really good and and I did come.

0:13:24.740 --> 0:13:24.980
PW (LC clinic)
Yeah.

0:13:14.220 --> 0:13:26.460
P008
I worked for the NHS for 12 years previously, so I sort of knew I had a bit of an idea of what should you know what should happen and what you know, like what's expected and it it more than my expectations.

0:13:27.80 --> 0:13:27.510
PW (LC clinic)
Right.

0:13:27.520 --> 0:13:27.750
PW (LC clinic)
OK.

0:13:27.760 --> 0:13:36.320
PW (LC clinic)
Well, that's, that's that's obviously fantastic and you probably you know I'm I'm I'm kind of kind of West guess the answer to this next question S.

0:13:36.330 --> 0:13:42.820
PW (LC clinic)
But did you feel that you know your opinions in relation to any aspects of this when you were interacting with staff?

0:13:42.830 --> 0:13:44.330
PW (LC clinic)
Did you feel that your opinion was valued?

0:13:45.420 --> 0:13:47.440
P008
Yes, because I do think.

0:13:58.360 --> 0:13:58.540
PW (LC clinic)
Hi.

0:14:6.920 --> 0:14:7.420
PW (LC clinic)
Right.

0:13:49.450 --> 0:14:9.360
P008
Again, I think I can remember writing a very quick review on their Facebook page, and I was then contacted, I think to ask if they could anonymously use it, maybe to put on the website or to but other patients.

0:14:9.370 --> 0:14:12.750
P008
I do have a a recollection of that happening as well.

0:14:13.330 --> 0:14:13.680
PW (LC clinic)
OK.

0:14:13.690 --> 0:14:15.0
PW (LC clinic)
So I mean, so very much.

0:14:15.10 --> 0:14:17.740
PW (LC clinic)
So then you know, they they actually sep003 you out for.

0:14:18.220 --> 0:14:18.440
P008
Yeah.

0:14:17.970 --> 0:14:20.30
PW (LC clinic)
Uh, your opinion that you're given, OK. OK.

0:14:20.60 --> 0:14:20.330
P008
Umm.

0:14:20.720 --> 0:14:25.640
P008
And obviously coming from that background, I know how important patient feedback is.

0:14:25.740 --> 0:14:29.790
P008
So, and that's why I think I just did it with my own accord.

0:14:29.800 --> 0:14:38.650
P008
Actually, I think I'd liked the Facebook page and I think I saw that they'd put a post up and I thought, yeah, you, you know, you've really been helping me.

0:14:38.700 --> 0:14:41.390
P008
So I thought I, you know, just as a little.

0:14:41.400 --> 0:14:41.950
P008
Thank you.

0:14:41.960 --> 0:14:45.210
P008
I'm gonna write something nice back because you know, everyone.

0:14:47.290 --> 0:14:47.490
PW (LC clinic)
Umm.

0:14:45.220 --> 0:14:48.790
P008
Here's the bad side of things in the NHS and you don't get a lot of people praising.

0:14:48.800 --> 0:14:49.650
P008
So that's why I did it.

0:14:50.500 --> 0:14:52.530
PW (LC clinic)
Yeah, I I I can agree with that for sure.

0:14:53.120 --> 0:14:53.390
P008
Umm.

0:14:53.20 --> 0:14:54.710
PW (LC clinic)
And OK.

0:15:0.570 --> 0:15:0.860
P008
Umm.

0:15:3.220 --> 0:15:3.480
P008
Umm.

0:14:55.320 --> 0:15:7.910
PW (LC clinic)
And you know, the other thing that was obviously, you know, aside from a novel, the novel virus was obviously the way in which, you know, services were were given to patients such as yourself.

0:15:6.690 --> 0:15:8.660
P008
Umm yeah.

0:15:15.760 --> 0:15:15.960
P008
Yeah.

0:15:7.920 --> 0:15:17.390
PW (LC clinic)
So I wanted about your experience of the kind of telephone Nash, you know, online or virtual and care that you receive.

0:15:17.400 --> 0:15:17.660
PW (LC clinic)
What?

0:15:17.670 --> 0:15:20.90
PW (LC clinic)
What did you make of of of that, S?

0:15:20.640 --> 0:15:27.720
P008
For me it was really good because I was getting tired and still do get tired and exhausted.

0:15:27.730 --> 0:15:28.900
P008
Very, very easily.

0:15:29.270 --> 0:15:44.940
P008
I was having a lot of appointments at the hospital anyway, and some wep003s I'd be there, sort of maybe three times a wep003, and it was nice actually to be able to do something that was in my own home.

0:15:45.990 --> 0:15:46.410
PW (LC clinic)
Right.

0:15:46.420 --> 0:15:46.940
PW (LC clinic)
OK.

0:15:47.690 --> 0:15:54.700
PW (LC clinic)
You know, had you previously, you know, prior to the pandemic, had any other sort of health related appointments carried out in that way?

0:15:56.850 --> 0:16:14.510
P008
Only possibly I think years ago and I'd had maybe the old consultation over the phone with a GP or something like that, but it it wasn't really something that was that was done.

0:16:14.520 --> 0:16:22.510
P008
So it was, it was actually nice because it meant that and if I didn't want to get dressed, I didn't have to get dressed.

0:16:23.50 --> 0:16:23.250
PW (LC clinic)
Yeah.

0:16:23.160 --> 0:16:27.940
P008
And you know, it could be done in my lunch hour if it needed to be.

0:16:28.970 --> 0:16:33.40
P008
And and it was nice just to not have to be going into a hospital setting.

0:16:37.870 --> 0:16:38.20
P008
Yeah.

0:16:41.20 --> 0:16:41.450
P008
Yeah.

0:16:33.940 --> 0:16:42.630
PW (LC clinic)
OK, I I suppose particularly in in you know in in a time where this the the virus was at large kind of thing and.

0:16:41.760 --> 0:16:43.750
P008
Yeah, it was for me.

0:16:43.760 --> 0:16:52.160
P008
It was very good and as well having been in hospital and been really poorly, it was, you know, he's you sort of thing.

0:16:52.200 --> 0:16:53.900
P008
Oh God, I never wanna have to go back there again.

0:16:53.910 --> 0:16:57.60
P008
And you're sort of being taken back there, sort of, you know, three times a wep003.

0:16:57.480 --> 0:17:0.90
P008
The fact that it was there, like, oh, we can do this over the phone.

0:17:0.100 --> 0:17:1.880
P008
It was like ohh that's really good.

0:17:2.510 --> 0:17:3.50
PW (LC clinic)
Yeah.

0:17:3.60 --> 0:17:3.400
PW (LC clinic)
Yeah.

0:17:3.410 --> 0:17:3.610
PW (LC clinic)
OK.

0:17:4.230 --> 0:17:7.790
PW (LC clinic)
UM, yeah, it's a so in.

0:17:14.660 --> 0:17:14.890
P008
Umm.

0:17:12.850 --> 0:17:23.380
PW (LC clinic)
I specific circumstances season of you know, obviously the the the fact that there was a virus circulating and so with that in mind. You know going back to the sort of. You know the time at which this occurred.

0:17:23.830 --> 0:17:24.680
PW (LC clinic)
Did you actually?

0:17:29.680 --> 0:17:29.900
P008
Yeah.

0:17:25.170 --> 0:17:33.540
PW (LC clinic)
Would it be possible to say that you actually preferred then the online kind of virtual I you know, instead of the the face to face interactions, yeah.

0:17:34.260 --> 0:17:35.100
P008
Yeah, definitely.

0:17:35.510 --> 0:17:37.800
P008
And it was for me.

0:17:37.810 --> 0:17:39.320
P008
It was a lot.

0:17:39.670 --> 0:17:42.60
P008
A lot easier because you hadn't got.

0:17:42.70 --> 0:17:46.560
P008
I mean, I don't drive, but it was, you know, having to find somebody to take me.

0:17:46.900 --> 0:17:47.300
PW (LC clinic)
Right.

0:17:46.650 --> 0:17:52.540
P008
Which would be relying on parents finding a parking space, walking to the actual clinic.

0:17:52.630 --> 0:17:56.800
P008
By the time you get there, you exhausted and it it just made it so much easier.

0:17:58.200 --> 0:17:58.620
PW (LC clinic)
Right.

0:17:58.670 --> 0:17:59.120
PW (LC clinic)
OK.

0:17:59.130 --> 0:17:59.380
PW (LC clinic)
Yeah.

0:17:59.390 --> 0:18:1.560
PW (LC clinic)
No, that sounds yeah.

0:18:1.570 --> 0:18:3.270
PW (LC clinic)
On many levels are a good thing, right?

0:18:3.690 --> 0:18:3.810
P008
Yeah.

0:18:5.340 --> 0:18:20.720
PW (LC clinic)

And just, you know in your think back at this sort of light to the entirety of your appointments with the with the long COVID clinic and we're we're all of your appointments carried out kind of in a virtual way or did you have any in person interactions?

0:18:22.580 --> 0:18:27.190
P008
With the pulmonary rehab now, it was all it was all over the phone.

0:18:27.240 --> 0:18:28.200
P008
And I think so.

0:18:28.210 --> 0:18:42.960
P008
I think I had a teams teams meeting as well, so that that was it, but I didn't have any other to any other long Cairo clinics because at the time the confusion was just put down to OU had COVID.

0:18:43.770 --> 0:18:49.320
P008
And and the tiredness was, you know you've had COVID.

0:18:49.410 --> 0:18:51.320
P008
I was also quite badly anemic as well.

0:18:54.800 --> 0:18:55.240
PW (LC clinic)
Right.

0:18:51.330 --> 0:19:0.190
P008
I ended up needing an infusion and so really, and it was just the, you know, the the primary rehab side of it.

0:19:5.30 --> 0:19:5.150
P008
Yeah.

0:19:2.210 --> 0:19:8.550
PW (LC clinic)
It's just the, so it was mainly mainly that was the the care that you received was from yeah, from the pulmonary rehab, OK.

0:19:8.200 --> 0:19:16.830
P008
And my other things like respiratory hematology were done and and I had to have like a lot of scans done as well.

0:19:16.840 --> 0:19:19.80
P008
So that was all done at hospital.

0:19:19.90 --> 0:19:24.820
P008
The only other thing that actually was done over the phone were my anticoag appointments.

0:19:26.300 --> 0:19:26.690
PW (LC clinic)
OK.

0:19:26.700 --> 0:19:27.270
PW (LC clinic)
And they were.

0:19:27.280 --> 0:19:29.160
PW (LC clinic)
So they were in, they were face to face where they.

0:19:29.580 --> 0:19:30.330
P008
No, they wrote.

0:19:31.260 --> 0:19:31.570
PW (LC clinic)
Oh, sorry.

0:19:30.340 --> 0:19:32.850
P008
They wrote the they were just like the phone.

0:19:32.860 --> 0:19:33.810
P008
Yeah, that was.

0:19:33.820 --> 0:19:36.100
P008
And that was a lot better as well.

0:19:36.580 --> 0:19:37.860
P008
And and it.

0:19:37.920 --> 0:19:39.150
P008
Yeah, it was definitely.

0:19:39.160 --> 0:19:41.520
P008
The phone call would definitely an easier way for me.

0:19:42.570 --> 0:19:43.640
PW (LC clinic)
OK, OK.

0:19:43.650 --> 0:19:43.870
PW (LC clinic)
Yeah.

0:19:44.200 --> 0:19:46.370
PW (LC clinic)
And and were there.

0:19:46.380 --> 0:20:12.710
PW (LC clinic)
Did you you know with with that method of communication, S, was there any sort of difficulties around, you know using those kind of methods and say the use of like PPE you know with with uh with there any difficulties around you know being say on a virtual call or team with me and you know would would you or would would the person that you're speaking to perhaps have been wearing PPE.

0:20:12.760 --> 0:20:20.70
PW (LC clinic)
I just wonder if that might might have made things difficult in anyways or obviously maybe not and not something you experienced.

0:20:21.370 --> 0:20:24.280
P008
And no, it wasn't really something I experienced.

0:20:25.150 --> 0:20:25.930
PW (LC clinic)
OK, OK.

0:20:26.310 --> 0:20:34.210
PW (LC clinic)
And and you know, throughout your communications, you know, did you generally feel that you were able to ask questions as things moved along?

0:20:35.300 --> 0:20:37.60
P008
I think I would have been, yeah, yeah.

0:20:38.130 --> 0:20:38.850
PW (LC clinic)
Yeah. OK.

0:20:40.270 --> 0:20:57.440
PW (LC clinic)
And I just wanted to sort of, you know if it's possible to say, I mean I again I would probably touched on this and I think I can kind of perhaps you know roughly understand where you might go with this one, but where you, you know satisfied, would you say with the care that you received at the clinic and if so why?

0:20:58.780 --> 0:20:59.210
P008
I'm.

0:20:59.300 --> 0:21:9.710
P008
Yeah, was because they were from what I can remember, because I had a lot of other appointments and I was working as well.

0:21:10.20 --> 0:21:10.830
P008
They were.

0:21:15.580 --> 0:21:15.850
PW (LC clinic)
Hmm.

0:21:11.0 --> 0:21:17.450
P008
I think they would have probably had to have been quite flexible as well with the appointments and and they I think yeah.

0:21:17.460 --> 0:21:19.580
P008
And they I always sort of had an appointment.

0:21:19.590 --> 0:21:26.680
P008
So and for them to sort of work around everything else that was going on with me was really good.

0:21:27.570 --> 0:21:28.20
PW (LC clinic)
Yeah.

0:21:28.70 --> 0:21:28.600
PW (LC clinic)
OK.

0:21:28.610 --> 0:21:32.370
PW (LC clinic)
So there was a lot of flexibility it seemed in, in, in the in, in the service.

0:21:32.380 --> 0:21:32.530
PW (LC clinic)
Yeah.

0:21:30.640 --> 0:21:33.450
P008
Yeah, yeah, yeah.

0:21:34.370 --> 0:21:43.490
PW (LC clinic)
And any other you know, any other things that you that you were particularly pleased with in you know that you've found like you know that was satisfying in terms of the service you received?

0:21:44.630 --> 0:21:47.310
P008
I think really was just uh UM.

0:21:48.250 --> 0:21:50.440
P008
I mean, to me, pulmonary rehab was new.

0:21:50.450 --> 0:21:54.20
P008
I mean, I I'd never sort of heard of it before.

0:21:54.390 --> 0:22:0.180
P008
I know now that it's quite common thing, but yeah, no, it was just it was.

0:22:0.190 --> 0:22:0.840
P008
It was just good.

0:22:3.730 --> 0:22:3.850
PW (LC clinic)
Yeah.

0:22:0.850 --> 0:22:9.50
P008
Just being able to be done on the phone and they always try to do it at, you know, a time that would that would suit me.

0:22:12.350 --> 0:22:12.630
PW (LC clinic)
Hmm.

0:22:20.320 --> 0:22:20.690
PW (LC clinic)
Right.

0:22:9.60 --> 0:22:30.810
P008
You know, the lady was always nice to talk to and the emails and that that I got sent out cause my reading didn't deteriorate immediately and that not only been sort of in the last maybe sort of, I'm not quite sure, but my comeback comes more as a possible dementia diagnosis.

0:22:31.270 --> 0:22:31.550
PW (LC clinic)
OK.

0:22:31.20 --> 0:22:32.990
P008
But my reading good hero, Emily.

0:22:33.0 --> 0:22:34.870
P008
But I also think they sent out as well.

0:22:35.260 --> 0:22:41.360
P008
There was a way to talk you through, like, slowing your breathing down when you're having an asthma attack as well.

0:22:42.600 --> 0:22:42.960
PW (LC clinic)
OK.

0:22:42.410 --> 0:22:44.760
P008
And you know, and things like that.

0:22:50.670 --> 0:22:50.950
PW (LC clinic)
Hmm.

0:22:44.770 --> 0:22:51.920
P008
So it was really just a good overall use of all the resources that they've got and it was, it was just a really good service.

0:22:52.850 --> 0:22:53.240
PW (LC clinic)
Fantastic.

0:23:10.120 --> 0:23:10.380
P008
Umm.

0:22:54.630 --> 0:23:11.530
PW (LC clinic)
You know, but we but it be possible to sort of say if there were any kind of, you know, looking back at it, would you say there's anywhere that, you know, aspects of that could have been improved in your opinion, S, or things that could obviously tell that you're quite pleased with the service that you've got?

0:23:11.570 --> 0:23:20.520
PW (LC clinic)
I just wondered whether there were things that went well, but perhaps could have been improved even more if a given change was made.

0:23:21.360 --> 0:23:26.210
P008
One no, I I think it was all you know it it was.

0:23:26.490 --> 0:23:31.740
P008
It was all fine from from my point of view, so I know I wouldn't have made any improvements to it.

0:23:32.360 --> 0:23:32.890
PW (LC clinic)
OK.

0:23:36.350 --> 0:23:38.180
P008
Umm yeah.

0:23:33.10 --> 0:23:39.410
PW (LC clinic)
And sort of like thinking about sort of going forward that's, you know, well, let's not imagine, let's not imagine another pandemic obviously.

0:23:40.210 --> 0:23:40.450
P008
Umm.

0:23:39.420 --> 0:23:53.500
PW (LC clinic)
But you know, if something like this happened again, would there be any sort of changes that you would like to see made to a service like that if they were going to provide care again at some point perhaps in the future?

0:23:53.90 --> 0:23:55.880
P008
Umm, no, I I think no.

0:23:55.890 --> 0:23:56.820
P008
I think they they did.

0:23:56.910 --> 0:24:2.600
P008
They did a brilliant job under exceptionally bad circumstances and I don't think you could have.

0:24:3.90 --> 0:24:13.800
P008
I think they were lucky because in a way, because they probably were one of the few departments that could work in the way that they did.

0:24:14.310 --> 0:24:14.520
PW (LC clinic)
Umm.

0:24:13.850 --> 0:24:20.570
P008
And you don't think, well, how can I have attained call with somebody then to assess my breathing?

0:24:21.130 --> 0:24:23.280
P008
But 8000 work?

0:24:24.370 --> 0:24:24.840
PW (LC clinic)
Yeah.

0:24:34.580 --> 0:24:34.830
P008
Umm.

0:24:24.850 --> 0:24:45.0
PW (LC clinic)
So I mean was there, do you think there was anything you know like you say with that with that in mind, you know that perhaps do you think that that that those sort of like being given those breathing techniques, would it have perhaps under ideal circumstances would that have been something that could have potentially been better if it was face to face or would you say that you didn't really know?

0:24:43.650 --> 0:24:50.110
P008
No, not site no, because it no, it was.

0:24:50.600 --> 0:24:54.270
P008
It was all explained really well and really clearly.

0:24:54.280 --> 0:24:55.620
P008
So now I think it was.

0:24:57.630 --> 0:25:4.220
P008
It was good and also I think as well at that time you're quite conscious of not wanting to come into contact with too many people.

0:25:5.210 --> 0:25:5.700
PW (LC clinic)
Yeah.

0:25:5.750 --> 0:25:9.360
PW (LC clinic)
OK, so that was a real benefit just that not having to go into public places.

0:25:7.700 --> 0:25:10.590
P008
Yeah, yeah, definitely.

0:25:10.40 --> 0:25:12.550
PW (LC clinic)
Uh, you know, in in the midst of a pandemic?

0:25:12.560 --> 0:25:13.320
PW (LC clinic)
Yeah, absolutely.

0:25:14.800 --> 0:25:16.30
PW (LC clinic)
S, I've actually covered this.

0:25:16.80 --> 0:25:17.650
PW (LC clinic)
You know, I've covered everything.

0:25:17.780 --> 0:25:18.100
P008
OK.

0:25:28.210 --> 0:25:29.760
P008
No, I think you covered everything.

0:25:17.720 --> 0:25:30.710
PW (LC clinic)
All the questions that I wanted to ask you are just wondered if you know do you was there anything that you touched on that you feel as if we haven't kind of highlighted enough anything that I'm yeah.

0:25:30.720 --> 0:25:30.910
PW (LC clinic)
OK.

0:25:30.920 --> 0:25:38.660
PW (LC clinic)
So last question then and any other comments at this stage or have you sort of do you feel if you you know with covered everything adequately so to speak?

0:25:38.600 --> 0:25:41.310
P008
And now I think everything's been been done really well.

0:25:41.320 --> 0:25:42.40
P008
Thank you.

0:25:42.870 --> 0:25:43.680
PW (LC clinic)
Thanks very much, S.

0:25:43.690 --> 0:25:48.210
PW (LC clinic)
I'll just hit stop on the record button here and just bear with me a second.

-- ----- ----- -----

P009

0:0:0.0 --> 0:0:1.130
P009
Yeah, that's just come out.

0:0:0.270 --> 0:0:2.450
PW (LC clinic)
That's is that letting you know? OK, brilliant.

0:0:3.840 --> 0:0:23.180
PW (LC clinic)
Alright, so just for the purpose of the tape, I am here with participant P009. It is the 31st of May 2023 and it's 2 minutes past 3 P009 and just for the purpose to type. Are you happy for me to be recording this conversation today?

0:0:23.750 --> 0:0:24.500
P009
Yes, I am.

0:0:25.230 --> 0:0:38.820
PW (LC clinic)
Thank you so much. OK. So you know, P009, if you happy, we just start with a very broad question. What was your experience like of being referred to the long COVID clinic in Hertfordshire?

0:0:39.630 --> 0:0:42.660
P009
So I actually referred myself.

0:0:43.190 --> 0:0:43.660
PW (LC clinic)
OK.

0:0:44.240 --> 0:0:51.520
P009
So I had COVID in the April 2020 and then.

0:1:8.270 --> 0:1:8.640
PW (LC clinic)
OK.

0:0:52.980 --> 0:1:11.810
P009
I think it was about August. I was stuck. August, September. I was still breathless, having heart palpitations and and I knew there was a clinic for people that hadn't necessarily been in hospital because of where I worked. I was aware that there was a clinic.

0:1:12.470 --> 0:1:17.440
P009
And so I spoke to my GP and got him to refer me.

0:1:30.100 --> 0:1:30.600
P009
Yeah.

0:1:18.980 --> 0:1:34.950
PW (LC clinic)
OK, so ohhh so you went to the GP being aware of and just you know, so obviously your job at the

time was senior sister in the crystal Care at Lister Hospital. OK. So you've heard about it through the list of hospital.

0:1:35.370 --> 0:1:43.400
P009
Well, still with my staff that couldn't work clinically were involved in the speaking to patients post COVID.

0:1:43.760 --> 0:1:44.280
PW (LC clinic)
Yeah.

0:1:44.670 --> 0:1:51.310
P009
And referring to and setting up this clinic, but mostly it was patients that were had been in hospital.

0:1:52.120 --> 0:1:52.550
PW (LC clinic)
Right.

0:1:52.610 --> 0:1:55.10
P009
And I've never been admitted.

0:1:55.910 --> 0:2:0.60
P009
Although I was quite poorly at the time, you know earlier on so.

0:1:59.760 --> 0:2:0.200
PW (LC clinic)
Yeah.

0:2:1.940 --> 0:2:4.900
P009
So yeah, I was just aware that this was going on.

0:2:12.730 --> 0:2:13.80
P009
Yeah.

0:2:5.560 --> 0:2:22.570
PW (LC clinic)
OK. And so it was more a case of kind of you know for want of a better phrase, you know, hearing, hearing about it on the Grapevine kind of thing or so nothing official came round to make you aware via sort of like the NHS sort of communications et cetera. It was purely word of mouth. OK.

0:2:20.690 --> 0:2:28.890
P009
No, no, I can't remember anything. I think it was just purely that because I I was aware of the service that was being started.

0:2:37.490 --> 0:2:37.940
P009
Yeah.

0:2:29.630 --> 0:2:45.700
PW (LC clinic)
Yeah, OK. And and uh, you know, is it possible? I know we're obviously thinking back to 2020, aren't we when you you kind of would have accessed it did was it you find it easy to get an appointment after you spoke to your GP?

0:2:53.240 --> 0:2:53.690
PW (LC clinic)
OK.

0:2:46.810 --> 0:2:59.370
P009
Yeah, I had. I can't remember how long it was, possibly a week I'd. I can't remember an e-mail from either an e-mail or a telephone call from the service and I was referred to the.

0:3:0.500 --> 0:3:3.160
P009
It was the physiotherapist, basically.

0:3:4.590 --> 0:3:6.470
P009
So I started now.

0:3:4.240 --> 0:3:6.790
PW (LC clinic)
Right, OK. That was the first person that you spoke to.

0:3:7.230 --> 0:3:9.360
P009
Yeah. And I did it all. It was all online.

0:3:11.310 --> 0:3:16.840
PW (LC clinic)
OK, so when when you say online it was a like a a conversation like this on a like an Ms. teams call or?

0:3:18.830 --> 0:3:20.50
PW (LC clinic)
Video call OK.

0:3:16.370 --> 0:3:22.620
P009
And yes, video yeah, video. I think it was zoom zoom then where we using so.

0:3:23.370 --> 0:3:40.0
PW (LC clinic)
OK. And you know that was that sort of something that you had you know previously had, you know prior to sort of pandemic etcetera. Had you previously had any sort of healthcare services delivered by the Internet if you like or by zoom?

0:3:45.140 --> 0:3:45.720
PW (LC clinic)
Yeah.

0:3:39.900 --> 0:3:47.50
P009
No, no, it was totally new, wasn't it, to all of us doing anything, any meetings by.

0:3:47.930 --> 0:3:48.560
P009
Same.

0:3:49.570 --> 0:3:51.470
P009
Yeah, completely easy.

0:3:52.560 --> 0:3:52.800
P009
Like.

0:3:48.950 --> 0:3:53.140
PW (LC clinic)
Yes, absolutely. So how did you find? How did you find that P009?

0:3:55.270 --> 0:3:57.840
P009
I didn't find it too bad actually, because I.

0:4:2.210 --> 0:4:2.740
PW (LC clinic)
OK.

0:3:58.770 --> 0:4:7.150
P009
Was using it at work as well and so I suppose I was a little bit more familiar with it and.

0:4:8.630 --> 0:4:10.550
P009
Yeah, it was. It was OK.

0:4:23.480 --> 0:4:23.890
P009
Umm.

0:4:30.850 --> 0:4:31.290
P009
I'm.

0:4:11.80 --> 0:4:32.190
PW (LC clinic)
And you know with with if you sort of like, umm, it can think back and sort of you know compare it to what you predominantly more used to which is sort of face to face appointments you know was

there anything that you thought was lacking or was there anything that was that was better that you preferred just sort of comparing those two mediums. I just wanted to sort of like how yeah.

0:4:32.790 --> 0:4:33.430
P009
I think it.

0:4:41.900 --> 0:4:42.700
PW (LC clinic)
Yeah.

0:5:3.830 --> 0:5:4.170
PW (LC clinic)
Umm.

0:4:34.570 --> 0:5:4.340
P009
You probably didn't feel like if you've got an appointment with the doctor or physio, whatever, you know, you've got a time limit, don't you? You've you've got 10 minutes and I think there was a little bit less of that, that pressure to get everything said and you know, I I did have notes that if you know, I need to mention this, whatever, but I didn't feel that I was pressurized, that it was, you know, we've only got 10 minutes. You've gotta say what you need to say and.

0:5:5.140 --> 0:5:6.600
P009
But on the other hand.

0:5:7.300 --> 0:5:15.970
P009
Our issue was trying to teach me breathing exercises and I did find that quite difficult doing it on a video it was.

0:5:15.720 --> 0:5:16.270
PW (LC clinic)
Right.

0:5:16.670 --> 0:5:18.300
P009
You couldn't really see.

0:5:19.40 --> 0:5:22.430
P009
What my breathing pattern was and and.

0:5:24.340 --> 0:5:27.290
P009
Yeah, I thought that that was a little bit difficult.

0:5:30.440 --> 0:5:30.810
P009
Yeah.

0:5:34.20 --> 0:5:34.490
P009
Yeah.

0:5:27.990 --> 0:5:45.880
PW (LC clinic)
So kind of a bit of a mixed bag really. So you know you you like the fact that there was less kind of like time pressure, but there was almost like this lacking of would you say sort of like, you know, just literally being able to see clearly sort of was it the demoing of the breathing techniques that was difficult to and?

0:5:45.240 --> 0:5:51.450
P009
Yeah, because she was like, and I had to. I don't know how to stand sit back from the.

0:5:56.130 --> 0:5:56.590
PW (LC clinic)
Right.

0:5:52.70 --> 0:6:0.90
P009
Computer so she could see my chest to see my breathing, which is quite hot, is quite hard to see somebody breathe.

0:6:5.730 --> 0:6:7.120
P009
Yeah, yeah.

0:6:0.730 --> 0:6:15.300
PW (LC clinic)
Yes. Yeah. And particularly if you're not in the same location as them. Yeah. Yeah. So, so that, you know, things like that you think potentially would be better to be carried out face to face, I should imagine. Yeah.

0:6:13.790 --> 0:6:19.380
P009
Yeah. Yeah. But it, you know, at the time, nobody was face to face with anything where they so.

0:6:19.990 --> 0:6:21.0
PW (LC clinic)
Yeah. Yeah. Well.

0:6:20.560 --> 0:6:22.140
P009
You're just going to stop that.

0:6:22.580 --> 0:6:36.810
PW (LC clinic)
With that in mind, P009, you know as you sort of carried on with your kind of ongoing appointments through the clinic, where was it, what were all your appointments carried out virtually or were there, was there any, you know, in person interactions?

0:6:40.970 --> 0:6:41.460
PW (LC clinic)
OK.

0:6:37.110 --> 0:6:41.750
P009
No, that was it was all uh, videoed. Yeah, it was all but.

0:6:42.740 --> 0:7:2.10
PW (LC clinic)
OK. And this is kind of more just kind of, uh, suppose you know linked to kind of the emotion of perhaps how you felt at the time, but how did you know once you had the referral once you had the appointment, how did you feel just knowing that you had that appointment for your for long COVID basically?

0:7:2.130 --> 0:7:4.480
P009
And I felt.

0:7:6.210 --> 0:7:15.980
P009
Better in the I had something to work with and so I have breathing exercises. She sent me over information.

0:7:18.230 --> 0:7:22.980
P009
So that I could download. So I did feel I had.

0:7:24.270 --> 0:7:26.360
P009
I could do something positive.

0:7:27.450 --> 0:7:27.880
PW (LC clinic)
Yeah.

0:7:27.390 --> 0:7:28.500
P009
To help myself.

0:7:31.890 --> 0:7:32.330
P009
Yeah.

0:7:28.940 --> 0:7:33.810
PW (LC clinic)
Yeah. So it felt kind of more proactive that you were kind of, you actually had something.

0:7:36.290 --> 0:7:36.840
P009
Yeah.

0:7:35.40 --> 0:7:38.130
PW (LC clinic)
The could alleviate some of the symptoms, yeah.

0:7:38.510 --> 0:7:38.910
P009
Yeah.

0:7:39.400 --> 0:7:39.950
PW (LC clinic)
OK.

0:7:40.240 --> 0:7:53.880
PW (LC clinic)
And and just thinking about sort of, you know the the, the, the treatments, was that the breathing techniques was was that predominantly that you know the main sort of treatment option that you were given where others for any other symptoms?

0:8:7.690 --> 0:8:8.170
PW (LC clinic)
OK.

0:7:55.620 --> 0:8:11.130
P009
I was given advice on vitamins, so I started taking a quite a high dose vitamin D and vitamin C and a general multivitamin.

0:8:11.890 --> 0:8:12.660
P009
And.

0:8:13.560 --> 0:8:14.250
P009
I had.

0:8:22.700 --> 0:8:23.80
PW (LC clinic)
Hmm.

0:8:15.90 --> 0:8:24.520
P009
Breathing exercises. But also I have what was it was like a a constant cough and she said it was more of a.

0:8:25.870 --> 0:8:28.20
P009
It was a habit rather than a.

0:8:28.760 --> 0:8:29.750
P009
A response.

0:8:31.10 --> 0:8:31.540
PW (LC clinic)
OK.

0:8:30.320 --> 0:8:43.0
P009
It's as such they need having a cough as such. So it says it's. It's a bit of a habit and you've got to try and suppress it. So that was quite useful trying to suppress the cough and.

0:8:45.730 --> 0:8:48.350
P009
I'm trying to think of what are the symptoms. I had it so I had.

0:8:48.420 --> 0:8:48.800
PW (LC clinic)
Hmm.

0:8:52.190 --> 0:8:52.650
PW (LC clinic)
OK.

0:8:48.430 --> 0:8:54.680
P009
And tachycardias and and she basically that was.

0:8:56.660 --> 0:9:9.220
P009
To see see what happens with that? Really. And it was more if I was going uphill and they'd had suddenly ohh, I'd done any exercise, I'd get a tachycardia that would last for.

0:9:11.740 --> 0:9:12.110
PW (LC clinic)
Right.

0:9:10.50 --> 0:9:12.890
P009
Quite a long time before it's down.

0:9:13.950 --> 0:9:14.360
PW (LC clinic)
OK.

0:9:13.670 --> 0:9:19.90
P009
And but it over time it it it got better on its own but yeah.

0:9:20.370 --> 0:9:20.980
PW (LC clinic)
OK.

0:9:20.420 --> 0:9:23.650
P009
And and and the tiredness was.

0:9:24.390 --> 0:9:30.400
P009
I think that was a combination because I was still working. So I think that was a bit combination of everything.

0:9:31.620 --> 0:9:37.890
PW (LC clinic)
OK. And were there any sort of you know for for fatigue, were there any additional sort of treatments that you or you know?

0:9:38.50 --> 0:9:43.590
PW (LC clinic)
I'm sort of interventions that you were given for the fatigue, P009.

0:9:45.380 --> 0:9:46.80
PW (LC clinic)
No. OK.

0:9:43.380 --> 0:9:48.850
P009
No, not really. Just to listen to my body and.

0:9:49.560 --> 0:9:53.40
P009
Stop if I can sort of thing, you know, rest if I can so.

0:9:53.580 --> 0:9:54.390
P009
And.

0:9:55.240 --> 0:10:3.350
P009
And how was in one of those OK situations where I was supposed to have reduced my hours at work and and in the end increased my hours at work so?

0:10:4.600 --> 0:10:5.940
P009
Yeah, yeah.

0:10:10.250 --> 0:10:10.600
P009
Umm.

0:10:17.670 --> 0:10:18.720
P009
Yes, cause I've.

0:10:24.150 --> 0:10:24.510
P009
Yeah.

0:10:3.390 --> 0:10:35.560
PW (LC clinic)

Right. OK. Yeah. So yeah, kind of a, a A suppose an odd situation to be someone that probably is called upon for more hours because of the pandemic, but was also suffering with the very illness that the pandemic revolved around. So yeah, OK, so with the treatment, the treatments that you were given, right, you know, did you feel as if like the process or the pathway that you were going down in terms of treatment was that was it explained to you were the treatment options made?

0:10:36.40 --> 0:10:45.870
PW (LC clinic)
Clear to you. You know, I just wanted to sort of like around the idea around Community communication and and and and what you thought of those aspects of of receiving care.

0:10:53.420 --> 0:10:53.800
PW (LC clinic)
Hmm.

0:10:45.30 --> 0:10:58.250
P009
And I think I think so. But but I think because I was talking to a physiotherapist. So when I sort of started to mention about my heart rate, et cetera, she did.

0:10:59.520 --> 0:11:8.620
P009
So say you always think if it doesn't settle down, you go back to your GP. There wasn't another route I could take. Speak to somebody else about it.

0:11:9.470 --> 0:11:9.900
PW (LC clinic)
Right.

0:11:10.900 --> 0:11:11.170
PW (LC clinic)
So.

0:11:9.340 --> 0:11:17.580
P009
And I think because I have knowledge, I wasn't over worried about that.

0:11:18.450 --> 0:11:18.970
PW (LC clinic)
OK.

0:11:18.870 --> 0:11:22.470
P009
And and it did improve on its own.

0:11:23.150 --> 0:11:23.830
P009
And.

0:11:38.430 --> 0:11:39.310
PW (LC clinic)
Umm yeah.

0:11:24.580 --> 0:11:41.600
P009
Overtime, but I think if I had been somebody without their knowledge or a been a bit unsure of, obviously it was really hard to get to speak to a GP as well. And I think I might have been a bit stumped.

0:11:48.960 --> 0:11:49.610
P009
Yeah.

0:11:42.430 --> 0:11:51.770
PW (LC clinic)
So did that seem a bit kind of circular? Almost like you've been referred by the GP and then you're being referred kind of back to the GP for things that OK, OK and.

0:11:57.890 --> 0:11:58.410
PW (LC clinic)
Right.

0:11:50.590 --> 0:12:1.420
P009
Yeah, I think so. I think there wasn't another route into another part of the clinic, the long COVID clinic. It was like a build dealing with the breathlessness rather than.

0:12:3.0 --> 0:12:4.730
P009
The whole picture, I think.

0:12:5.500 --> 0:12:9.390
PW (LC clinic)
Right. OK. And did I mean just, you know, did you did you end up?

0:12:10.340 --> 0:12:15.120
PW (LC clinic)
Referring self back to your Jeep, you know. Did you go back to your GP for an appointment for help with that? No. OK.

0:12:13.460 --> 0:12:28.550
P009
No, no, it did. It did settle down in time. And The funny thing was at the time I had my flu vaccine in probably the end of October, beginning of November, and that actually helped a lot of my symptoms.

0:12:29.230 --> 0:12:30.380
PW (LC clinic)
Right. OK.

0:12:30.780 --> 0:12:37.880
P009
Which was weird at the time and it was before the UM COVID vaccine came out. But yeah.

0:12:37.690 --> 0:12:51.720
PW (LC clinic)

Of course. Yes, absolutely. OK. So you know, just with that in mind, how confident did you feel, you know, in both your diagnosis and then, you know, subsequent treatments, you know, bearing in mind what we just spoke about?

0:12:53.170 --> 0:12:53.820
P009
I'm.

0:13:1.140 --> 0:13:1.670
PW (LC clinic)
Hmm.

0:12:55.170 --> 0:13:20.410
P009
Yeah, I was. I think I self diagnosed really. And because I'd had COVID went back to work, went back a little bit too soon and struggled for about a week and then just had this persistent breathlessness, especially on exercise or if I was rushing from one unit to the other because we're 2 units and.

0:13:21.780 --> 0:13:29.180
P009
And I'd have to get to the other and sit down. And and it it did take me a while before I thought, you know, this isn't right.

0:13:29.960 --> 0:13:37.20
P009
And that's when I rang the GP and said I think I've got long COVID and I know there's a clinic. Can you refer me?

0:13:37.810 --> 0:13:41.130
P009
And but the process of.

0:13:43.50 --> 0:13:48.800
P009
Of the treatments I think, yeah, I think that was it was right because the breathlessness was the most.

0:13:50.650 --> 0:13:52.420
P009
Important thing to me at the time.

0:13:52.770 --> 0:13:53.860
PW (LC clinic)
OK, OK.

0:13:53.180 --> 0:13:56.760
P009
It was the thing that triggered my request.

0:13:57.810 --> 0:13:58.510
P009
And.

0:13:59.250 --> 0:14:7.350
P009
And I, oh, I think the Jeep. I think I went for an extra. I can't remember who asked for the extra whether it was GP or the clinic.

0:14:8.560 --> 0:14:10.580
P009
And but I did have an extra at the beginning.

0:14:11.150 --> 0:14:20.770
PW (LC clinic)
OK. OK. So so kind of apart from that kind of like sort of what would you say slight bump in the road where you were asked to go back to your GP?

0:14:20.950 --> 0:14:21.470
P009
Yeah.

0:14:28.230 --> 0:14:28.550
P009
Umm.

0:14:36.480 --> 0:14:36.820
P009
Yeah.

0:14:22.90 --> 0:14:38.980
PW (LC clinic)
And and and as you say you know coming from sort of like a A you know a you know like a strong obviously backgrounds working in healthcare that didn't that didn't kind of bother you too much the everything else seemed to you understood it the treatment seemed correct, yeah. OK fantastic.

0:14:49.790 --> 0:14:50.590
P009
Yeah.

0:14:40.810 --> 0:15:5.920
PW (LC clinic)
And you know, just obviously I I totally appreciate this is a tricky ask because we're, we're gotta cast mine back good number of years now, which is which is odd and exist it's gone very quickly, but you're sort of first appoint with the clinic that you mentioned. Can you remember sort of what was involved in that first session you obviously mentioned the breathing techniques. Was there anything else in that first session?

0:15:6.460 --> 0:15:7.210
P009
Umm.

0:15:8.210 --> 0:15:16.20
P009
And came back. She I think we just discussed my symptoms and how I fell and.

0:15:17.130 --> 0:15:17.920
P009
And.

0:15:22.560 --> 0:15:22.900
PW (LC clinic)
Hmm.

0:15:19.260 --> 0:15:26.760
P009
I suppose what I hope to achieve and it was having some control over the the breathing really and.

0:15:28.310 --> 0:15:31.470
P009
And I suppose I was looking for a bit of reassurance that it was.

0:15:32.50 --> 0:15:32.450
PW (LC clinic)
Hmm.

0:15:32.630 --> 0:15:35.120
P009
I wasn't gonna be like this forever sort of thing. Yeah.

0:15:35.670 --> 0:15:52.160
PW (LC clinic)
Yeah. So that kind of brings me to another question. It's you know similar to whilst you for but you know just in terms of sort of like your your the emotional reaction so you know how on the day that you had your appointment or during the appointment, how did you feel if it's possible to say P009 as obviously?

0:15:52.230 --> 0:15:57.690
P009
I don't. I've been. I think I've been a little bit apprehensive to start with, obviously cause it's.

0:15:57.470 --> 0:15:57.840
PW (LC clinic)
Hmm.

0:15:58.520 --> 0:16:6.720
P009
Zoom and you're talking to somebody. Don't. Don't really know when you're on camera and all that. And I hate having my face on camera and.

0:16:7.590 --> 0:16:8.820
PW (LC clinic)
Yeah, you're not alone there.

0:16:8.750 --> 0:16:13.800
P009
I need to really strong Northern accent on the on the microphone and.

0:16:16.480 --> 0:16:19.250
P009
But yeah, she was, you know, and.

0:16:22.920 --> 0:16:24.100
PW (LC clinic)
Yeah, yeah.

0:16:20.220 --> 0:16:28.250
P009
Clicking the right buttons and things like that, you know, making sure you've got the right clients and all that. But no, I think other than that it was OK actually.

0:16:36.610 --> 0:16:36.950
P009
Umm.

0:16:28.690 --> 0:16:42.90
PW (LC clinic)
OK. And then so sort of like once you've had the appointment, you know and and you know you finished the call with the with the physiotherapist, you know, can you remember approximately how you might have felt, you know, once you've finally had that first appointment?

0:16:43.140 --> 0:16:45.450
P009
And yeah, I think because.

0:16:46.930 --> 0:16:49.740
P009
OK, so before because she gave me some.

0:16:51.990 --> 0:17:0.960
P009
Something that I could work with, some some exercises I could work with and be proactive and try and help myself. I think I've probably felt.

0:17:1.620 --> 0:17:3.320
P009
To advertise they achieved something.

0:17:6.260 --> 0:17:11.650
PW (LC clinic)
OK. Yeah. So you felt a bit more like you, you know, got a bit of a handle on.

0:17:14.620 --> 0:17:15.180
P009
Yeah.

0:17:19.110 --> 0:17:19.580
P009
Yeah.

0:17:13.410 --> 0:17:20.820
PW (LC clinic)
The symptoms and you know, he had something to work with, so a bit more hopeful. Would that be sort of an accurate thing to say, you know? Yeah. OK.

0:17:20.530 --> 0:17:20.940
P009
Yeah.

0:17:21.210 --> 0:17:23.540
PW (LC clinic)
And and and.

0:17:25.640 --> 0:17:25.870
P009
Yeah.

0:17:24.890 --> 0:17:35.0
PW (LC clinic)
You know, you know, we sort of mentioned the time wise. It was sort of like you know was was it did you spend you remember approximately how long you spent on on that first appointment?

0:17:35.650 --> 0:17:37.300
P009
It was probably.

0:17:38.430 --> 0:17:40.380
P009
20 minutes half an hour.

0:17:40.750 --> 0:17:42.70
PW (LC clinic)
OK, OK.

0:17:44.470 --> 0:17:45.150
PW (LC clinic)
Yeah.

0:17:42.580 --> 0:17:51.930
P009
Apartment breaks out, but I think it was. I can't either talked for more than half an hour, so I think 20 minutes. Half an hour would probably be about right.

0:17:59.940 --> 0:18:1.790
P009
Yeah, there's a lot happened since then.

0:17:52.540 --> 0:18:3.150
PW (LC clinic)
OK. Yeah. No. And and really, you know totally appreciate that we've you know it's it's it's a long time back. So you know I'm sure it's quite difficult to recall recall these things. Yeah, exactly. Yeah.

0:18:3.470 --> 0:18:16.680
PW (LC clinic)
Umm. And you know, sort of like from start to finish in terms of you know ongoing appointments approximately over what sort of time you know time span did you did you have contact with the long COVID clinic?

0:18:20.550 --> 0:18:21.390
PW (LC clinic)
Every two weeks.

0:18:15.870 --> 0:18:24.70
P009
I think I have an appointment every two weeks for and I probably had four or.

0:18:25.60 --> 0:18:26.530
P009
I have appointments.

0:18:27.100 --> 0:18:28.710
PW (LC clinic)
4-5 appointments every two weeks so.

0:18:27.470 --> 0:18:42.360
P009
And before right before. So yeah. And I can remember, we always arranged what date time and date and for the next one. And I think I'm sure there were, there weren't weakly. I think they were fortnightly.

0:18:43.60 --> 0:18:43.530
PW (LC clinic)
OK.

0:18:43.550 --> 0:18:46.800
P009
They're not four or five sessions, I think.

0:18:50.720 --> 0:18:51.240
P009
Yeah.

0:18:52.220 --> 0:18:52.730
P009
Yeah.

0:18:47.210 --> 0:18:52.840
PW (LC clinic)
So quite to sort of like maybe 2-3 months around about, around about that in total. Yeah. OK.

0:18:54.290 --> 0:18:54.630
P009
Yeah.

0:18:53.690 --> 0:19:3.720
PW (LC clinic)
And and we we've mentioned some of the assessments that that, that that you've had obviously mentioned the your next ray sort of like you had a.

0:19:8.330 --> 0:19:8.680
P009
Yeah.

0:19:4.360 --> 0:19:17.110
PW (LC clinic)
You know, if somebody passed you in terms of, you know, you're breathing technique, et cetera, was it is anything that we haven't mentioned that you know any other assessments or treatments that you were you were provided P009.

0:19:18.170 --> 0:19:18.760
P009
Uh.

0:19:19.70 --> 0:19:20.460
P009
No, not that I can remember.

0:19:20.830 --> 0:19:21.450
PW (LC clinic)
OK.

0:19:21.750 --> 0:19:36.300
PW (LC clinic)
And and you know, so initially you spoke to a physiotherapist with subsequent appointments. Did you did you tend to always speak to the same person or did was there kind of a variety of people that you spoke to throughout that time?

0:19:35.710 --> 0:19:39.180
P009
No, it was always the same. It was always the same girl, yeah.

0:19:38.950 --> 0:19:40.270
PW (LC clinic)
Same number staff, OK.

0:19:40.280 --> 0:19:40.520
P009
Yeah.

0:19:41.510 --> 0:19:45.180
PW (LC clinic)
Alright, so a so just kind of like.

0:19:46.290 --> 0:19:55.200
PW (LC clinic)
Considering sort of, you know interactions with staff and and and you know, how did you feel in terms of you know, did you feel supported in your interactions?

0:19:56.150 --> 0:19:58.200
P009
And yes, yes, definitely.

0:19:57.320 --> 0:19:58.450
PW (LC clinic)
Yeah. OK.

0:20:0.420 --> 0:20:6.290
PW (LC clinic)
And, you know, was was that down to the that individual member of staff or, you know, did you?

0:20:5.620 --> 0:20:10.290
P009
Yeah, I think so. Because she said, you know, I could e-mail her at any time and.

0:20:21.920 --> 0:20:22.260
PW (LC clinic)
Hmm.

0:20:25.300 --> 0:20:25.740
PW (LC clinic)
Right.

0:20:11.790 --> 0:20:26.260
P009
If I had any problems and I think I after she thought assigned me off, I think I did. Then did send her an e-mail of a few weeks later and to say that I I was feeling much better as well so.

0:20:27.300 --> 0:20:29.710
PW (LC clinic)
OK, so it was nice to have that sort of level of contact.

0:20:29.990 --> 0:20:30.560
P009
Yeah.

0:20:31.350 --> 0:20:51.250
PW (LC clinic)
OK. And you know, did you feel that you, you know, I mean, it might be the case obviously that you you you didn't have any reason to sort of voice any concerns, but did you feel that you you know that you did or you would have been able to kind of voice any concerns about the diagnosis and any of the subsequent treatment that you may have received?

0:20:50.910 --> 0:20:51.620
P009
Umm.

0:20:55.660 --> 0:20:55.930
PW (LC clinic)
Umm.

0:20:53.520 --> 0:20:58.450
P009
I'm not sure actually because I I only had contact with her. I don't know.

0:20:59.430 --> 0:21:14.560
P009
If there was, if she gave me any information about anybody else within the team, now I I do know some of new some of the people that were setting it up so I could have gone to them. But that was only because of where I worked. So.

0:21:15.140 --> 0:21:15.980
P009
And.

0:21:17.240 --> 0:21:20.990
P009
I can't remember if she in the initial e-mail.

0:21:21.680 --> 0:21:23.980
P009
They gave me any other contact.

0:21:25.440 --> 0:21:27.40
PW (LC clinic)
OK, OK.

0:21:27.170 --> 0:21:30.970
PW (LC clinic)
And so, you know, in terms of sort of.

0:21:36.900 --> 0:21:37.270
P009
You.

0:21:47.350 --> 0:21:47.880
P009
Yeah.

0:21:32.100 --> 0:21:53.80
PW (LC clinic)
Having that sort of support, then perhaps you know there was a kind of. Did you feel as if there was something that could have been improved there in terms of kind of, you know, you've been given this diagnosis or you self diagnose and then you had subsequent treatment that you were trying out like the breathing techniques and so is it the case that you kind of felt as if?

0:21:53.950 --> 0:21:56.800
PW (LC clinic)
There would have been a, you know, there was a need, if you like, for.

0:22:3.0 --> 0:22:3.670
P009
Yeah.

0:21:58.120 --> 0:22:4.660
PW (LC clinic)
You know, the ability to have a bit more of like a feedback sort of you know session with, with, with members of staff. Hmm.

0:22:4.540 --> 0:22:6.10
P009
Yeah, or even.

0:22:8.250 --> 0:22:13.160
P009
To have either had a conversation with a, a magic, or.

0:22:14.70 --> 0:22:21.900
P009
And an e-mail. Even just maybe just to reassure me about a I do remember that my.

0:22:26.390 --> 0:22:26.820
PW (LC clinic)
Hmm.

0:22:23.20 --> 0:22:27.0
P009
Erratic heart rate was quite a concern to me.

0:22:27.560 --> 0:22:27.990
PW (LC clinic)
Yeah.

0:22:27.880 --> 0:22:32.430
P009
And and I just maybe if I'd had access to.

0:22:33.650 --> 0:22:36.370
P009
Talked to a medic within the clinic rather than.

0:22:36.680 --> 0:22:38.610
P009
And physio.

0:22:39.450 --> 0:22:39.920
PW (LC clinic)
OK.

0:22:39.170 --> 0:22:41.210
P009
Uh, yeah.

0:22:44.760 --> 0:22:45.200
P009
Yeah.

0:22:42.0 --> 0:22:46.710
PW (LC clinic)
That might that might have been some that could have delayed some of the some of the fears.

0:22:47.180 --> 0:22:47.610
P009
Yeah.

0:22:47.20 --> 0:22:55.980
PW (LC clinic)
Umm and, you know, did you feel as if you're your opinion that you know that you may have raised or you know if we just sort of think.

0:22:56.130 --> 0:23:5.710
PW (LC clinic)
And in terms of, you know, you might not have raised any kind of like strong opinions about things, but did you feel as if you could if that makes sense, you know, do you feel as if your opinion was valued?

0:23:7.590 --> 0:23:9.350
P009
I can't remember, to be honest.

0:23:10.90 --> 0:23:10.670
P009
Uh.

0:23:9.660 --> 0:23:10.730
PW (LC clinic)
Yeah, yeah.

0:23:13.50 --> 0:23:16.190
P009
And I said, I suppose as it was all new.

0:23:17.420 --> 0:23:18.110
P009
And.

0:23:19.0 --> 0:23:21.560
P009
I don't know whether I'd have had an.

0:23:26.370 --> 0:23:26.810
PW (LC clinic)
Umm.

0:23:23.140 --> 0:23:29.980
P009
And opiniones such to suppose it's you you don't know what to expect so.

0:23:29.890 --> 0:23:30.280
PW (LC clinic)
Yeah.

0:23:30.750 --> 0:23:32.640
P009
Anything you get was a bonus.

0:23:33.490 --> 0:23:33.920
PW (LC clinic)
Right.

0:23:33.360 --> 0:23:34.190
P009
Rather than.

0:23:35.50 --> 0:23:50.340
P009
And being critical of something or be, you know, having well, I was expecting this to happen because I had no expectations. I didn't know what was gonna happen. So I suppose that's quite difficult to reflect back on.

0:23:55.380 --> 0:23:55.760
P009
Yeah.

0:23:50.860 --> 0:23:58.280
PW (LC clinic)
Yeah. So much like you would have needed to have prior experience of this kind of situation before. Yeah, that makes sense. OK.

0:23:59.500 --> 0:24:9.360
PW (LC clinic)
And so like, just to, you know, totally broad question again about you know your experience of the clinic, you know overall would you say that you were satisfied with the care that you received?

0:24:10.40 --> 0:24:14.410
PW (LC clinic)
And if so, you know, why would that? Why would that? Why would that be?

0:24:15.850 --> 0:24:21.300
P009
Yeah, I suppose I I I was satisfied. Like I keep saying it was, it was only that.

0:24:21.640 --> 0:24:25.270
P009
And I suppose because of the heart rate thing that.

0:24:26.90 --> 0:24:26.480
PW (LC clinic)
Hmm.

0:24:25.970 --> 0:24:29.390
P009
And how they sort of ended with?

0:24:37.450 --> 0:24:37.940
PW (LC clinic)
Yeah.

0:24:30.290 --> 0:24:41.450
P009
If it doesn't improve, go back to GP and I thought well end up back in the same situation, don't I? And and that's I suppose that's the only thing that would have.

0:24:42.490 --> 0:24:43.470
P009
Made it more.

0:24:44.290 --> 0:24:50.820
P009
And satisfying, I suppose, as if I'd been referred within the clinic, I suppose.

0:24:51.340 --> 0:24:52.250
PW (LC clinic)
Yeah, cause.

0:24:52.370 --> 0:24:57.460
PW (LC clinic)
That, you know, I I'm obviously correct me if I'm wrong, P009, but it kind of.

0:25:18.440 --> 0:25:19.10
P009
And.

0:24:58.700 --> 0:25:19.210
PW (LC clinic)
It's did it feel a bit odd that you've been referred to this specialist clinic if you like that then the main you know, I don't wanna put words in your mouth, but I'm sort of like getting the impression that this tachycardia the symptoms of that were potentially the symptom that you were most concerned about. Would that be, would that be fair?

0:25:21.360 --> 0:25:23.410
P009
I was concerned about it but it.

0:25:29.420 --> 0:25:30.600
PW (LC clinic)
As well, yeah, yeah.

0:25:49.710 --> 0:25:50.50
PW (LC clinic)
Umm.

0:25:25.300 --> 0:25:51.550
P009
I was concerned about my and my breathing as well because it yeah, I did get very short of breath at times and had to have to stop and sit down for 10 minutes or so. And then my heart will kick in. And so I think it was a combination and I and I suppose I thought if I could get the breathing under control, the heart would follow and which it did eventually, but it.

0:25:52.410 --> 0:25:53.290
P009
Not Australia away.

0:25:53.900 --> 0:25:55.200
PW (LC clinic)
Yeah. OK.

0:25:55.460 --> 0:26:5.280
PW (LC clinic)
And so, yeah, so so kind of would it be fair to say you know, majority of things that you experienced, you were satisfied with, but there were just kind of like?

0:26:5.360 --> 0:26:5.590
PW (LC clinic)
And.

0:26:25.10 --> 0:26:25.380
P009
Yeah.

0:26:29.240 --> 0:26:29.680
P009
Yeah.

0:26:7.100 --> 0:26:32.350
PW (LC clinic)
You know, just going back on, you know what you've mentioned so far today, which is obviously the breathing, you know, the learning the breathing techniques would have been better off face to face and having some kind of like onward referral to a. So let's say a specialist who would be able to look at that, those symptoms around sort of tachycardia, et cetera, that would have been things that really could have perhaps been improved.

0:26:34.530 --> 0:26:35.0
PW (LC clinic)
OK.

0:26:32.900 --> 0:26:35.530
P009
Yeah, I think so. I think that's fair to say.

0:26:45.650 --> 0:26:45.880
P009
No.

0:26:35.830 --> 0:26:47.390
PW (LC clinic)
Yeah. OK. Any any other sort of you know other than what we've mentioned there? Were there any other improvements that you think could have been made at all, P009 and it could be you know any aspect of it?

0:26:49.0 --> 0:26:51.850
P009
And no, I don't think so.

0:26:52.740 --> 0:26:58.560
P009
And before you know, like we say, the 2020, we didn't know anything, did we and it was.

0:26:59.980 --> 0:27:4.10
P009
It was new to the was a new service. It was.

0:27:4.670 --> 0:27:20.300
P009
They're new patients with and everybody's long COVID is different. And it's. Yeah, no, I don't. I don't think I could have said anything else needed to be improved at that time. I think it's an ongoing thing, isn't it as?

0:27:20.540 --> 0:27:21.20
PW (LC clinic)
Yeah.

0:27:21.30 --> 0:27:21.640
P009
And.

0:27:22.630 --> 0:27:23.10
PW (LC clinic)
Yeah.

0:27:22.400 --> 0:27:26.670
P009
As new knowledge was gained, then it was.

0:27:28.620 --> 0:27:31.220
P009
He changed approaches and things.

0:27:31.790 --> 0:27:39.40
PW (LC clinic)
Yeah. So you would kind of like you appreciated the fact that it was like kind of a moving feast, if that makes sense. You know that. Yeah. OK.

0:27:36.960 --> 0:27:43.130
P009
Yeah. Yeah, cause you know, physical carried we everything evolved.

0:27:43.930 --> 0:27:47.830
P009
Every month we were doing something different, so yeah.

0:27:46.440 --> 0:27:51.200
PW (LC clinic)
Yeah. And you probably you probably had a, you know compared to you know.

0:27:58.320 --> 0:27:58.720
P009
Yeah.

0:28:5.790 --> 0:28:6.160
P009
Yeah.

0:28:10.940 --> 0:28:11.490
P009
Yeah.

0:27:52.400 --> 0:28:15.910
PW (LC clinic)
Patients that would have come through the service that that don't have any experience of work in the NHS, you know that I I suppose you have more of a kind of understanding of of what that's like to be sort of in a situation. Well you were working in it yourself, weren't you and I presume came across situations where which were novel and all the rest of it. So yeah, that makes sense. And you know, just the benefits of hindsight.

0:28:17.270 --> 0:28:35.240
PW (LC clinic)
Were there any changes that that you think you know if, heaven forbid, anything like this ever happened again that, you know, any change that you think you know? Ohh. Actually, in hindsight, you know, if if the service had this or it did, it would be kind of an improvement. As I say, if anything like this happens a bit ever happened again.

0:28:36.20 --> 0:28:38.280
P009
At at just think if.

0:28:43.910 --> 0:28:44.260
PW (LC clinic)
Hmm.

0:28:42.120 --> 0:28:51.150
P009
A face to face meeting. And I know that's not always possible and we didn't have anybody face to face for, like, nearly two years, but.

0:28:58.390 --> 0:28:58.820
PW (LC clinic)
Yeah.

0:28:51.410 --> 0:28:58.930
P009
And I think, yeah, face to face because it's always just more comfortable, isn't it? And.

0:29:0.740 --> 0:29:6.780
PW (LC clinic)
And do you think sort of you know because just sort of like expanding on what you mentioned earlier about?

0:29:9.910 --> 0:29:10.300
P009
Hmm.

0:29:17.240 --> 0:29:17.700
P009
Yeah.

0:29:7.510 --> 0:29:33.620
PW (LC clinic)
Getting these the the breathing technique right and you know also the physiotherapist that was, you know, trying to obviously look at what you were doing there was that complication around that. Do you think that sort of having a face to face appointment would have taken would have delayed more of your fears around your symptoms, etc. Just having that physical sort of experience of somebody actually being in the room with you and seeing you and?

0:29:33.410 --> 0:29:33.930
P009
Yeah.

0:29:34.750 --> 0:29:35.770
P009
Yeah, I think so.

0:29:34.900 --> 0:29:37.270
PW (LC clinic)
You know, with that? Yeah. OK.

0:29:37.360 --> 0:29:37.680
P009
Yeah.

0:29:39.190 --> 0:30:0.270
PW (LC clinic)
And OK, fine. Final question at P009. Really is you know with we've covered kind of like a fair amount of ground today and I just wonder if there's things that you you know that have come up that you had a chance to expand on and basically if you got any other sort of comments around sort of the topic of your experience with the long COVID clinic.

0:30:0.370 --> 0:30:0.900
P009
And.

0:30:2.90 --> 0:30:6.710
P009
I don't think so. I think at the if I remember rightly at the time.

0:30:20.640 --> 0:30:20.970
PW (LC clinic)
Umm.

0:30:7.190 --> 0:30:29.0
P009
And you generally only seeing patients that have been in hospital, but there were quite a few patients that haven't even been in hospital. He developed long COVID. And I just I think people wouldn't have been aware that there was a clinic set up and I think.

0:30:29.960 --> 0:30:34.470
P009
I was possibly only able to access it because of where I worked.

0:30:35.270 --> 0:30:42.260
PW (LC clinic)
Right. So you, you you have concerns around sort of the notion that there be a lot of people out there that didn't receive any care?

0:30:42.590 --> 0:30:44.30
P009
Yeah, yeah.

0:30:43.480 --> 0:30:45.680
PW (LC clinic)
It's purely purely because they would have known about it.

0:30:47.300 --> 0:30:48.240
PW (LC clinic)
Hmm.

0:30:46.260 --> 0:30:50.470
P009
That's right. Yeah, because it was all novel, wasn't it? And it was all.

0:30:50.820 --> 0:30:51.500
P009
And.

0:30:53.280 --> 0:30:54.200
P009
People that.

0:30:55.230 --> 0:31:14.400
P009
Had COVID didn't go into hospital and then it was months later, people saying, oh, there's this thing called long COVID. That's some people are still suffering with effects afterwards and I don't think those people would if they haven't been in hospital. I think they've missed. They're missed out.

0:31:21.340 --> 0:31:21.880
P009
Yeah.

0:31:14.830 --> 0:31:24.580
PW (LC clinic)
Yeah. So, yeah, you'd have. You'd have a lot of potentially, a lot of people that would just have to would have just been left with with no, no options around them.

0:31:35.480 --> 0:31:35.990
PW (LC clinic)
Yeah.

0:31:23.90 --> 0:31:38.520
P009
Yeah, because I don't even know if G at the time because like I say, this was very early on at the time where the GP's were aware that they could refer patients that haven't been in hospital because I know my GP.

0:31:39.260 --> 0:31:50.710
P009
I had quite a good relationship with him and he was like, yeah, P009, that's what you want. I will do that. But I don't know whether he was aware that it was generally only for.

0:31:52.190 --> 0:31:53.160
P009
Impatience.

0:32:2.440 --> 0:32:2.970
P009
Umm.

0:32:6.210 --> 0:32:6.680
P009
Yeah.

0:31:54.240 --> 0:32:19.80
PW (LC clinic)
Right. So you know, yeah, if the if the, you know, NHS staff members aren't aware of the the the clinic, it's probably highly unlikely that many patients are going to especially if they don't work work within the NHS. So there could be a lot more to be done around kind of making people aware under such circumstances that there is some help available yeah.

0:32:21.920 --> 0:32:22.500
PW (LC clinic)
Yeah.

0:32:18.290 --> 0:32:28.800
P009
That, that, that might have changed over time as well. So that might be more available now, more known about than it was then.

0:32:30.180 --> 0:32:30.510
P009
Yeah.

0:32:38.610 --> 0:32:39.170
P009
Yeah.

0:32:28.930 --> 0:32:40.10
PW (LC clinic)
More highlighted, yes, you might have. Yeah. It's a tricky thing, isn't it? I suppose with GP's you might, you know, GP surgeries, you might get information on posters and leaflets, et cetera. And when that was all.

0:32:43.930 --> 0:32:44.330
P009
Umm.

0:32:50.360 --> 0:32:50.840
P009
Yeah.

0:32:40.610 --> 0:32:55.350
PW (LC clinic)
And stopped. If you like, you know you. It does make you wonder what what would the channels then be of making patients aware that there are services available? Yeah, it's. I think that kind of like the the question that comes out of what you're saying, really, P009 is kind of.

0:32:56.430 --> 0:32:57.530
PW (LC clinic)
You know.

0:32:56.700 --> 0:33:1.380
P009
How? How was it? How will people know that there is that service?

0:33:1.310 --> 0:33:1.730
PW (LC clinic)
Hmm.

0:33:2.680 --> 0:33:3.450
P009
You know.

0:33:2.500 --> 0:33:3.630
PW (LC clinic)
Yeah, exactly.

0:33:4.360 --> 0:33:10.730
P009
Unless she you were picked up as as having been an impatient, then you wouldn't have known about it.

0:33:11.330 --> 0:33:12.550
PW (LC clinic)
Yeah, I think it's a really.

0:33:13.370 --> 0:33:20.30
PW (LC clinic)
Really important point because especially this you know lots of work around the idea of you know who gets access to healthcare.

0:33:20.610 --> 0:33:21.70
P009
Umm.

0:33:20.570 --> 0:33:21.770
PW (LC clinic)
Uh and?

0:33:22.470 --> 0:33:23.600
PW (LC clinic)
Well, you know.

0:33:34.560 --> 0:33:35.20
P009
Yeah.

0:33:39.430 --> 0:33:39.720
P009
Yeah.

0:33:23.680 --> 0:33:42.360
PW (LC clinic)
And your yourself and other people like, well, you know myself as well, you know, working within the NHS, you kind of you could argue that that that that there's a privileged position there isn't there of just you know hearing of these things of from from your work so yeah a really important question.

0:33:42.500 --> 0:33:51.710
PW (LC clinic)
And yeah, OK. Thank you as much, P009. And anything else that that we that that you know that we haven't touched on.

0:33:52.520 --> 0:33:54.590
P009
No, I don't think so. I think we've covered it.

0:33:55.250 --> 0:33:58.550
PW (LC clinic)
Fantastic. What I'll do is I'm just gonna press stop.

0:33:59.690 --> 0:34:1.70
PW (LC clinic)
On the recording.

-- ----- ----- -----

P011

0:0:0.0 --> 0:0:1.890
PW (LC clinic)
OK, just give it a SEC.

0:0:3.380 --> 0:0:6.690
DB
Oh, someone started transcription pick.

0:0:6.710 --> 0:0:9.300
DB
Starting recording and transcription like this come up.

0:0:8.910 --> 0:0:9.540
PW (LC clinic)
Brilliant.

0:0:9.910 --> 0:0:10.520
PW (LC clinic)
Fantastic.

0:0:10.530 --> 0:0:11.760
PW (LC clinic)
OK, so it's working great.

0:0:9.960 --> 0:0:12.100
DB
Yeah, who?

0:0:12.450 --> 0:0:16.280
PW (LC clinic)
OK, so the date today is the 15th of June.

0:0:16.290 --> 0:0:23.140
PW (LC clinic)
It is 8 minutes past four and I'm here with participant P011. Umm.

0:0:23.960 --> 0:0:27.500
PW (LC clinic)
And don't you happy for me to be recording this conversation today?

0:0:28.20 --> 0:0:29.80
DB
Yes, absolutely, yeah.

0:0:29.660 --> 0:0:30.150
PW (LC clinic)
Fantastic.

0:0:30.160 --> 0:0:31.10
PW (LC clinic)
Thank you so much.

0:0:31.940 --> 0:0:32.430
PW (LC clinic)
OK.

0:0:32.440 --> 0:0:35.990
PW (LC clinic)
So just sort of start off with like a very broad question.

0:0:36.0 --> 0:0:40.820
PW (LC clinic)
Dawn, what was your experience of being referred to the long COVID clinic?

0:0:42.570 --> 0:0:45.920
DB
I I I was quite happy to have that.

0:0:46.870 --> 0:0:48.890
DB
I do feel that it's needed.

0:0:50.70 --> 0:0:50.290
PW (LC clinic)
Yeah.

0:0:49.990 --> 0:0:55.370
DB
Umm, you know to to find out what what's next and what's going on?

0:0:55.380 --> 0:0:57.220
DB
There's so much happens with long COVID.

0:0:57.230 --> 0:0:58.320
DB
It's just unbelievable.

0:0:59.550 --> 0:0:59.880
PW (LC clinic)
It.

0:0:59.950 --> 0:1:0.270
PW (LC clinic)
Yeah.

0:1:0.280 --> 0:1:2.140
PW (LC clinic)
What in terms of what?

0:0:59.780 --> 0:1:2.480
DB
Yeah, I that's the.

0:1:2.150 --> 0:1:3.170
PW (LC clinic)
What it means is kind of thing.

0:1:3.40 --> 0:1:4.150
DB
What was meant?

0:1:6.70 --> 0:1:6.470
PW (LC clinic)
Right.

0:1:14.90 --> 0:1:14.410
PW (LC clinic)
Yeah.

0:1:4.160 --> 0:1:18.750
DB
The stuff that goes wrong with you and trying to understand it all and why it's happening and you know you didn't put yourself in this position and it it's mentally and physically debilitating.

0:1:20.50 --> 0:1:20.720
PW (LC clinic)
Absolutely.

0:1:20.730 --> 0:1:21.0
PW (LC clinic)
Yeah.

0:1:21.10 --> 0:1:24.150
PW (LC clinic)
So, you know, was it was it would you say that?

0:1:24.340 --> 0:1:24.690
PW (LC clinic)
How?

0:1:24.700 --> 0:1:24.890
PW (LC clinic)
How?

0:1:24.900 --> 0:1:26.210
PW (LC clinic)
How did you feel once you had you?

0:1:26.700 --> 0:1:34.300
PW (LC clinic)
You know, the first appointment was kind of arranged and I just wanted to sort of like how you felt at the point at which you knew that you were gonna have an appointment with the with the long COVID clinic.

0:1:35.110 --> 0:1:49.260
DB

Well, I had a like a A a meeting with a long coat with people previously, but I kind of felt that they talked at us and not to us and it was just like they were reading the script.

0:1:50.360 --> 0:1:51.600
PW (LC clinic)
OK so.

0:1:51.290 --> 0:1:52.630
DB
I didn't feel that.

0:1:52.730 --> 0:1:58.210
DB
I mean when I tried to put a little bit of input in, I felt like I was being shut down.

0:1:59.340 --> 0:2:15.230
PW (LC clinic)
OK, so you know in terms of sort of like you know, did so then if I ask you this question, you know Dawn, did you feel that it was possible for you to kind of like voice your concerns about any kind of like, you know treatments or diagnosis that were being given you?

0:2:16.140 --> 0:2:16.510
DB
What?

0:2:16.600 --> 0:2:17.20
DB
What?

0:2:18.720 --> 0:2:18.990
PW (LC clinic)
Hmm.

0:2:17.180 --> 0:2:30.120
DB
Not really, no, because the people, the people that were in the meeting kind of thing and the other recipient, umm, bed, no one actually really said anything, but no one encouraged anybody to say anything.

0:2:30.330 --> 0:2:31.850
DB
And I found that really weird.

0:2:32.700 --> 0:2:33.30
PW (LC clinic)
OK.

0:2:33.40 --> 0:2:34.570
PW (LC clinic)
And what was this a particular?

0:2:32.830 --> 0:2:35.330
DB
Umm, I spoke about well.

0:2:35.120 --> 0:2:35.600
PW (LC clinic)
Was it?

0:2:37.940 --> 0:2:38.100
DB
Yeah.

0:2:36.100 --> 0:2:38.450
PW (LC clinic)
Was it a group situation, this one door?

0:2:39.0 --> 0:2:39.870
DB
Hmm. Yes.

0:2:40.310 --> 0:2:45.230
PW (LC clinic)
And you remember what it which, which sort of like which part of the service it was, was it for?

0:2:46.790 --> 0:2:47.320
PW (LC clinic)
Breathing.

0:2:47.330 --> 0:2:50.680
PW (LC clinic)
Was it pulmonary rehab or can you remember what it was for that one?

0:2:51.80 --> 0:2:52.690
DB
Remember to be honest with you.

0:2:53.250 --> 0:2:53.750
PW (LC clinic)
OK.

0:2:53.340 --> 0:2:54.750
DB
Uh, it was just.

0:3:2.540 --> 0:3:2.820
PW (LC clinic)
OK.

0:2:54.760 --> 0:3:7.280
DB
It was basically about how to how to cope with long COVID the fatigue and or things like that, but really was one of those there no one was interacting.

0:3:8.90 --> 0:3:8.600
PW (LC clinic)
Right.

0:3:8.610 --> 0:3:10.720
PW (LC clinic)
So it was quite a it felt quite passive.

0:3:11.450 --> 0:3:12.510
DB
Ohh absolutely.

0:3:12.520 --> 0:3:13.120
DB
You know, they.

0:3:12.890 --> 0:3:13.150
PW (LC clinic)
Yeah.

0:3:16.330 --> 0:3:16.780
PW (LC clinic)
Yeah.

0:3:13.130 --> 0:3:16.940
DB
I wake some people up here at this rate, but I'm.

0:3:17.890 --> 0:3:20.760
DB
But no, I I did feel it was a waste.

0:3:21.580 --> 0:3:22.50
PW (LC clinic)
Right.

0:3:22.210 --> 0:3:22.830
PW (LC clinic)
OK.

0:3:22.900 --> 0:3:23.920
PW (LC clinic)
So you did?

0:3:22.490 --> 0:3:24.850
DB
So why should I pay the bill?

0:3:25.470 --> 0:3:25.830
PW (LC clinic)
Yeah.

0:3:25.840 --> 0:3:26.140
PW (LC clinic)
Did you?

0:3:26.150 --> 0:3:26.560
PW (LC clinic)
Did you?

0:3:26.570 --> 0:3:36.10
PW (LC clinic)
Did you feel as if the sort of environment was such that you didn't feel comfortable to to to, to speak about your experiences or ask questions etcetera?

0:3:36.830 --> 0:3:41.700
DB
Well, when I saw that no one else is doing anything, I thought maybe it's just we've gotta listen.

0:3:42.530 --> 0:3:43.350
PW (LC clinic)
Right, I see.

0:3:42.810 --> 0:3:45.750
DB
I I kind of said my little bits.

0:3:48.430 --> 0:3:48.670
PW (LC clinic)
Yeah.

0:3:45.760 --> 0:3:49.30
DB
Well, everybody can do their own thing kind of thing, you know, and then moved.

0:3:52.350 --> 0:3:52.590
PW (LC clinic)
OK.

0:3:49.390 --> 0:3:53.960
DB
And I thought, OK, obviously hear what I've got to say.

0:3:54.620 --> 0:3:55.250
PW (LC clinic)
Yeah.

0:3:55.260 --> 0:3:55.540
PW (LC clinic)
Yeah.

0:3:55.550 --> 0:3:55.730
PW (LC clinic)
OK.

0:3:55.740 --> 0:4:2.810
PW (LC clinic)
And was this now was this was this done because I know that you know, there's there's many ways in which sort of like services were provided.

0:4:2.820 --> 0:4:4.630
PW (LC clinic)
Was this a face to face event?

0:4:4.800 --> 0:4:5.950
PW (LC clinic)
Donna was this carried out?

0:4:5.960 --> 0:4:8.700
PW (LC clinic)
Sort of on on a platform like we're using now.

0:4:17.500 --> 0:4:17.820
PW (LC clinic)
OK.

0:4:9.210 --> 0:4:21.400
DB
Now it's like we're using now, but we we all had our faces up so that the video was on you know some people put it and some people didn't, but it was just no interaction and it was just so bland.

0:4:21.410 --> 0:4:24.200
DB
And I thought this is a complete waste of time.

0:4:24.830 --> 0:4:25.270
PW (LC clinic)
Right.

0:4:25.280 --> 0:4:25.760
PW (LC clinic)
OK.

0:4:25.830 --> 0:4:30.610
PW (LC clinic)
So it would be fair to say that you didn't particularly feel supported during this event.

0:4:29.770 --> 0:4:31.470
DB
No, it was babe.

0:4:33.270 --> 0:4:33.520
PW (LC clinic)
OK.

0:4:31.480 --> 0:4:37.90
DB
Anything at all, you know how to cope with the cough and things like that.

0:4:37.100 --> 0:4:40.640
DB
And and just no one seemed to particularly interested in it.

0:4:41.20 --> 0:4:42.720
DB
And I'm not sure if it was the speaker.

0:4:43.870 --> 0:4:44.250
PW (LC clinic)
Right.

0:4:49.580 --> 0:4:49.840
PW (LC clinic)
Yeah.

0:4:43.970 --> 0:4:51.820
DB
Uh, you know, I've done lots of speaking when I was when I was at work, you know, at forms and things like that.

0:4:56.310 --> 0:4:56.640
PW (LC clinic)
Yeah.

0:4:51.830 --> 0:4:58.990
DB
And just thought, God, you know, I need to get up there and tell them, you know, say to him anybody questions anything that worked for you.

0:4:59.70 --> 0:5:0.640
DB
Guess get some input here.

0:5:6.400 --> 0:5:7.60
PW (LC clinic)
Yep, absolutely.

0:5:1.30 --> 0:5:10.960
DB
You know, cause everybody's got different ways of how they deal with it and dealt with that and still deal at the my work for them might not work for everybody.

0:5:16.400 --> 0:5:16.720
PW (LC clinic)
Hmm.

0:5:10.970 --> 0:5:17.80
DB
But if it works for one out of 10, then bang, you know you've you've helped one person, one person.

0:5:18.660 --> 0:5:19.540
PW (LC clinic)
Yeah, yeah.

0:5:17.90 --> 0:5:21.110
DB
It's better than nothing, but the nothing at all.

0:5:21.120 --> 0:5:26.370
DB
And when I suggested cause we're vegan, we do a lot of our own medication.

0:5:31.700 --> 0:5:32.140
PW (LC clinic)
Right.

0:5:26.380 --> 0:5:37.340
DB
As such, I say that very loosely, by way of herbal remedies I and cleanses I'm and we do ******** cleanses sometimes.

0:5:37.600 --> 0:5:41.570
DB
Well, it mean when I had this mighty cough, the judge cause couldn't get rid of.

0:5:42.230 --> 0:5:48.920
DB
I'm and we started the lemon juice and celery juice and then it was herbal mixtures.

0:5:49.600 --> 0:5:49.860
PW (LC clinic)
Yeah.

0:5:49.550 --> 0:6:2.50
DB
And then we did a hard dog ******** cleanse on it with the what we ate as well, like having broth every day with certain vegetables and which are the ones that help you live in your lungs, etcetera.

0:6:2.290 --> 0:6:2.770
PW (LC clinic)
Right.

0:6:3.20 --> 0:6:5.990
DB
Did that and within nine days my coffee gone.

0:6:7.60 --> 0:6:7.360
PW (LC clinic)
Well.

0:6:6.280 --> 0:6:9.900
DB
Now this has been weeks, but then antibiotics galore.

0:6:10.820 --> 0:6:11.820
PW (LC clinic)
Yes. Yeah.

0:6:11.780 --> 0:6:13.710
DB
But tried to put across.

0:6:14.20 --> 0:6:23.480
DB
You know, maybe if people tried, you know, something other than antibiotics or if you wanna get rid of a cough.

0:6:23.490 --> 0:6:34.420
DB
This this worked for me, but you know you know, try if you like, but no, that was that was a, it was like, well, OK, it all work for some people, but I don't know if everybody has to do their own thing.

0:6:34.430 --> 0:6:36.650
DB
I thought, yeah.

0:6:36.850 --> 0:6:37.850
PW (LC clinic)
OK, OK.

0:6:37.860 --> 0:6:38.800
PW (LC clinic)
So.

0:6:39.220 --> 0:6:55.360
PW (LC clinic)
So yeah, so so if you could sum up sort of like a, you know what you would say was lacking from it, dawn, what would it, what would that have been was it was it you think it was the the way that the presentation was umm delivered or was it the content?

0:6:54.170 --> 0:6:59.320
DB
The person who there was a person doing it just seemed that she was reading from a script.

0:7:0.40 --> 0:7:0.880
PW (LC clinic)
Right. OK.

0:7:0.620 --> 0:7:4.890
DB
That was there was no right, OK, this is what we're about, guys, you know?

0:7:5.140 --> 0:7:8.730
DB
And embracing them all, there was none of that.

0:7:9.360 --> 0:7:9.600
PW (LC clinic)
OK.

0:7:9.170 --> 0:7:11.150
DB
Absolutely flat.

0:7:11.860 --> 0:7:12.270
PW (LC clinic)
OK.

0:7:12.280 --> 0:7:13.120
PW (LC clinic)
So yeah.

0:7:11.840 --> 0:7:17.170
DB
And when the save anything out and no one really said anything except.

0:7:16.770 --> 0:7:17.220
PW (LC clinic)
Right.

0:7:20.350 --> 0:7:20.800
DB
Yeah.

0:7:17.230 --> 0:7:23.750
PW (LC clinic)
So as you say, it felt pretty sterile and and and did you sort of mention that thought it was really a waste of time.

0:7:23.760 --> 0:7:29.360
PW (LC clinic)
You didn't feel as if there was anything that you could take away from it that was, you know, that you could say would have helped.

0:7:29.810 --> 0:7:31.230
DB
I think I sent the thing back.

0:7:31.240 --> 0:7:32.360
DB
I can't remember how to be honest.

0:7:32.370 --> 0:7:33.580
DB
You cause unfortunate with COVID.

0:7:33.590 --> 0:7:34.180
DB
You remember we go.

0:7:35.340 --> 0:7:35.620
PW (LC clinic)
Yeah.

0:7:34.190 --> 0:7:41.280
DB
So I have the place and without looking back on it and I can't remember what it would be called.

0:7:43.860 --> 0:7:44.160
PW (LC clinic)
Hmm.

0:7:41.290 --> 0:7:52.280
DB
Now I'd have to have a good old search, but you know, I just said I I didn't feel that it was beneficial to me at that point.

0:7:52.630 --> 0:7:55.620
DB
I felt that no one was included as such.

0:7:55.770 --> 0:7:58.780
DB
I kind of, you know, tried to just do it lightly.

0:7:59.480 --> 0:7:59.740
PW (LC clinic)
Yeah.

0:7:59.490 --> 0:8:1.800
DB
Rather complete waste of bloody time.

0:8:2.190 --> 0:8:2.790
PW (LC clinic)
Right.

0:8:2.250 --> 0:8:4.130
DB
You know, the woman should have.

0:8:4.170 --> 0:8:4.980
DB
Yeah, up to her game.

0:8:4.990 --> 0:8:5.610
DB
A bit embraced.

0:8:5.620 --> 0:8:7.810
DB
Everybody you know, I don't wanna tell him how to do it.

0:8:14.750 --> 0:8:14.990
PW (LC clinic)
Yeah.

0:8:8.410 --> 0:8:15.160
DB
I just tried for my own opinion, you know, gave her a little insight, but nothing.

0:8:15.630 --> 0:8:16.0
DB
I don't.

0:8:18.150 --> 0:8:18.570
PW (LC clinic)
Right.

0:8:16.10 --> 0:8:20.380
DB
I don't wanna rock the boat and get on the teams again.

0:8:21.130 --> 0:8:22.980
DB
Yeah, enough.

0:8:22.810 --> 0:8:29.100
PW (LC clinic)
And so, you know, DBjust sort of like, I mean, it might well be, you know, this is 1 component to it.

0:8:29.110 --> 0:8:36.620
PW (LC clinic)
But do you think that perhaps the online kind of method with regards to that, was that part of the problem?

0:8:36.630 --> 0:8:44.140
PW (LC clinic)
Would it have been something that you think perhaps could have been better delivered in a face to face situation or you think about that?

0:8:43.500 --> 0:9:0.700
DB
I think good, but I also think it's nice to hear other peoples opinions, other peoples what they've gone through you know so you get an understanding because you're kind of think of course I don't know anybody else that had it as bad as I did.

0:9:1.830 --> 0:9:2.200
PW (LC clinic)
Right.

0:9:1.580 --> 0:9:3.960
DB
You know, So what can what?

0:9:6.580 --> 0:9:7.10
PW (LC clinic)
I say.

0:9:3.970 --> 0:9:10.410
DB
Can anybody else tell me, you know, they've not been through that they won't understand.

0:9:10.420 --> 0:9:13.430
DB
All your good looks like a really bad cold can get that bed for a week.

0:9:13.560 --> 0:9:14.120
DB
OK, hang on.

0:9:14.130 --> 0:9:15.630
DB
The metals in three months and nearly dead.

0:9:16.20 --> 0:9:17.710
DB
You know there?

0:9:16.540 --> 0:9:17.820
PW (LC clinic)
Right. So.

0:9:22.470 --> 0:9:22.870
PW (LC clinic)
OK.

0:9:17.720 --> 0:9:25.130
DB
There's a one end of the spectrum and the other end of the spectrum, you know, and I just don't think that.

0:9:27.780 --> 0:9:32.370
DB
That meeting cause I've in all honesty, when I came out of hospital, I felt I've been dumped and left.

0:9:33.500 --> 0:9:33.720
PW (LC clinic)
Mm-hmm.

0:9:33.910 --> 0:9:35.250
DB
Yeah, I still couldn't walk properly.

0:9:36.120 --> 0:9:41.980
DB
Umm I I walked in enough to get out of that hospital because I just didn't wanna be there any longer.

0:9:42.390 --> 0:9:42.610
PW (LC clinic)
Yeah.

0:9:42.650 --> 0:9:44.220
DB
I three months was quite enough.

0:9:44.230 --> 0:9:44.590
DB
Thank you.

0:9:45.400 --> 0:9:45.830
PW (LC clinic)
Yes.

0:9:46.20 --> 0:9:46.830
PW (LC clinic)
Yeah, absolutely.

0:9:45.740 --> 0:9:49.220
DB
It it we know endless.

0:9:49.230 --> 0:9:57.650
DB
Cannulas going in cause my veins collapse and they got robbery and they got valved and God knows what else, you know.

0:9:57.660 --> 0:9:59.900
DB
And then less than 10% chance of survival.

0:10:0.850 --> 0:10:1.830
DB
You know, I'm a fighter.

0:10:8.400 --> 0:10:8.680
PW (LC clinic)
Right.

0:10:1.840 --> 0:10:11.900
DB
That's the way I am, but it was it was when you come out there, was it, you know, and you really are still quite drugged up.

0:10:12.850 --> 0:10:13.390
DB
You don't.

0:10:14.880 --> 0:10:15.260
PW (LC clinic)
Hmm.

0:10:13.400 --> 0:10:18.270
DB
You're not in the real world when you come out, you're you're kind of.

0:10:18.460 --> 0:10:22.240
DB
I mean, I was really my daughter for for you know, three or four days.

0:10:22.250 --> 0:10:22.950
DB
And then I went to.

0:10:23.180 --> 0:10:24.930
DB
I need to go home to mom.

0:10:24.940 --> 0:10:25.210
DB
You can't.

0:10:26.150 --> 0:10:26.990
DB
What account should?

0:10:27.0 --> 0:10:29.340
DB
You can't get upstairs, or so I can go or fours.

0:10:29.670 --> 0:10:32.240
DB
What it was like climbing Mount Everest, you know?

0:10:32.740 --> 0:10:33.660
PW (LC clinic)
Yeah, yeah.

0:10:32.250 --> 0:10:35.710
DB
But I got there mostly get it, you know.

0:10:36.630 --> 0:10:38.760
DB
But it was really hard.

0:10:38.850 --> 0:10:40.700
DB
And there was no support.

0:10:45.560 --> 0:10:46.950
PW (LC clinic)
OK, so this this.

0:10:40.770 --> 0:10:47.340
DB
I I found absolutely nothing when I came out and I think that, yeah.

0:10:47.290 --> 0:10:47.690
PW (LC clinic)
Yeah.

0:10:47.700 --> 0:10:51.410
PW (LC clinic)
So just to ask you, dawn, so this sort of presentation, would it be fair to say so?

0:10:51.420 --> 0:10:52.850
PW (LC clinic)
I wanna make sure that I'm getting it right.

0:11:11.970 --> 0:11:12.200
DB
Right.

0:10:52.860 --> 0:11:13.760
PW (LC clinic)
You know, you obviously along with many others, but you had quite a different experience quite obviously from what you're saying it quite a traumatic experience of going through COVID and to have this presentation, it didn't really it wasn't, would it be fair to say it wasn't really tailored towards your experience or you know it was more of a generalized?

0:11:14.900 --> 0:11:17.510
DB
Yeah, but well, this is what you do when you have a cough.

0:11:17.520 --> 0:11:19.190
DB
This what you do if you get tired.

0:11:19.200 --> 0:11:20.290
DB
You have little tip.

0:11:21.310 --> 0:11:21.510
PW (LC clinic)
Yeah.

0:11:20.340 --> 0:11:24.860
DB
You know, just literally just touching the surface.

0:11:25.690 --> 0:11:27.640
PW (LC clinic)
OK, up to that point.

0:11:27.650 --> 0:11:30.270
PW (LC clinic)
Dawn, had you ever previously been delivered?

0:11:30.280 --> 0:11:39.170
PW (LC clinic)
You know any health services by umm, you know via the Internet, if you like, you know with the the virtual platforms.

0:11:43.340 --> 0:11:43.700
PW (LC clinic)
Right.

0:11:43.710 --> 0:11:44.150
PW (LC clinic)
OK.

0:11:40.390 --> 0:11:44.530
DB
That that was the only one I'd had, but on the.

0:11:44.260 --> 0:12:8.710
PW (LC clinic)
And I mean, what did you think of the actual the idea of, you know, if we if it's possible to sort of like, you know, separate out the, the the kind of like the the bad experience that you had with the with with that group work, you know, what did you think of just the very idea or the medium of being delivered health services via and if a virtual platform?

0:12:9.280 --> 0:12:10.660
DB
I mean, I think it's a good idea.

0:12:11.50 --> 0:12:11.320
PW (LC clinic)
Hmm.

0:12:10.740 --> 0:12:19.590
DB
You know you can sit get 10 people in rather than one person each time we send it means you've got a six months to 12 months waiting list.

0:12:30.360 --> 0:12:30.580
PW (LC clinic)
Umm.

0:12:20.200 --> 0:12:36.670
DB
If you didn't get sort of 10 people or 6 or 10 people in, you know one lot, but encouraging them to speak up to say some, you know, well, what was your, what was, how did you feel about it, you know, called him by name, not just saying.

0:12:36.680 --> 0:12:41.130
DB
Does anybody got anything to say and all like, look, no, no, no.

0:12:42.0 --> 0:12:42.210
PW (LC clinic)
Yeah.

0:12:41.180 --> 0:12:43.740
DB
You know, the first they're not.

0:12:44.20 --> 0:12:45.410
DB
I mean, I can talk freely.

0:12:51.320 --> 0:12:51.560
PW (LC clinic)
Umm.

0:12:57.460 --> 0:12:57.810
PW (LC clinic)
Right.

0:12:45.420 --> 0:13:1.720
DB
They should know, but you know, at the end of the day, these people aren't used to talking on thing and like you could see there was sort of like not sure what to say here and you know, I wanted to intervene a bit, but I thought that's not my place.

0:13:2.840 --> 0:13:3.100
PW (LC clinic)
Yeah.

0:13:2.600 --> 0:13:5.450
DB
And will these people think what the hell does she cheers?

0:13:5.460 --> 0:13:16.200
DB
Talking like you know, but I just felt there was more needed in, in encouraging the people to come forward and say something that.

0:13:16.150 --> 0:13:18.120
PW (LC clinic)
Did you do you think that could have been proved?

0:13:18.330 --> 0:13:30.690
PW (LC clinic)
It could be improved by perhaps it being face to face, you know, if the circumstances allowed obviously would have been something that you think could have been better in a face to face situation.

0:13:33.180 --> 0:13:33.510
PW (LC clinic)
Hmm.

0:13:31.300 --> 0:13:40.570
DB
It's better for some people, people that aren't able to talk openly and I'll shy for one of another word.

0:13:41.130 --> 0:13:41.470
PW (LC clinic)
Hmm.

0:13:41.200 --> 0:13:42.990
DB
Uh, you know which is in May.

0:13:43.90 --> 0:13:47.630
DB
So I I'm quite happy with her. You know, like we're doing now.

0:13:48.80 --> 0:13:52.130
DB
Other people would prefer just to discuss what their problem is 1 to one?

0:13:53.190 --> 0:13:53.540
PW (LC clinic)
Right.

0:13:53.750 --> 0:14:0.90
DB
Ohh, it's purely down to the the person themselves if they're able to discuss that.

0:14:0.900 --> 0:14:1.390
PW (LC clinic)
Yeah.

0:14:1.460 --> 0:14:7.610
PW (LC clinic)
So so for you personally, dawn, you wouldn't have minded either whether it was face to face or online, you wouldn't particularly have a preference.

0:14:7.180 --> 0:14:9.250
DB
The not absolutely not.

0:14:11.910 --> 0:14:12.290
PW (LC clinic)
OK.

0:14:9.260 --> 0:14:15.730
DB
You know, I can I I'm happy with either if that I'm used to talk into 500 plus people.

0:14:16.300 --> 0:14:18.570
PW (LC clinic)
Right. OK.

0:14:15.740 --> 0:14:21.350
DB
So that's not a, you know, so, but other people aren't.

0:14:21.360 --> 0:14:29.930
DB
And and some of them on there that were in this, I think there was about eight of us, I think yes, I'm, you know, just didn't come up with anything.

0:14:30.820 --> 0:14:31.700
PW (LC clinic)
Right. OK.

0:14:31.730 --> 0:14:33.290
DB
I reading this thing.

0:14:33.300 --> 0:14:33.840
DB
Uh, it wouldn't?

0:14:33.850 --> 0:14:34.60
DB
No.

0:14:34.100 --> 0:14:34.680
DB
OK.

0:14:34.730 --> 0:14:35.600
DB
My next meeting.

0:14:35.610 --> 0:14:36.400
DB
Anyone. OK?

0:14:36.410 --> 0:14:38.240
DB
No, and it just was so flat.

0:14:38.940 --> 0:14:39.370
PW (LC clinic)
OK.

0:14:43.690 --> 0:14:43.930
DB
Yeah.

0:14:39.580 --> 0:14:47.420
PW (LC clinic)
So fairly sort of like a robotic sort of delivery and and and and that that made you feel kind of like not connected to what was going on perhaps?

0:14:47.940 --> 0:14:48.850
DB
Yeah, exactly.

0:14:48.860 --> 0:14:50.770
DB
I mean, I was a bit like, didn't it?

0:14:50.820 --> 0:14:53.130
DB
Trying to get on this thing again, you know.

0:14:53.510 --> 0:14:53.720
PW (LC clinic)
Umm.

0:14:53.140 --> 0:14:56.920
DB
But I mean, I was like, but you know and ohh.

0:14:56.930 --> 0:14:58.890
DB
Welcome them like, OK, you know.

0:14:59.670 --> 0:15:1.380
DB
OK, good.

0:15:1.870 --> 0:15:2.230
PW (LC clinic)
Right.

0:15:1.720 --> 0:15:4.360
DB
But it it was just flat, you know?

0:15:4.610 --> 0:15:4.850
PW (LC clinic)
Yeah.

0:15:12.910 --> 0:15:13.150
PW (LC clinic)
Yeah.

0:15:17.240 --> 0:15:17.450
PW (LC clinic)
Yeah.

0:15:4.370 --> 0:15:18.780
DB
And no, was putting in the input and that was quite sad really, because obviously some of those were struggling, otherwise it wouldn't be on on there, you know, and to help the people that are struggling with it, some people got harder than others.

0:15:19.970 --> 0:15:20.380
PW (LC clinic)
Yeah.

0:15:20.430 --> 0:15:22.670
PW (LC clinic)
OK, so plenty of room for improvement.

0:15:23.610 --> 0:15:23.830
DB
Yeah.

0:15:23.0 --> 0:15:27.290
PW (LC clinic)
I'm going down, going back to sort like the beginning of your journey, if you like.

0:15:32.100 --> 0:15:32.240
DB
Yeah.

0:15:27.300 --> 0:15:35.320
PW (LC clinic)
If we call it that, you know, going through the the the Hertfordshire Long COVID clinic, what was it like getting an appointment in the in the first instance?

0:15:37.550 --> 0:15:39.960
DB
Uh, I I don't think you know.

0:15:39.970 --> 0:15:44.730
DB
I I don't think I've actually been to a clinic as such just on this bit.

0:15:45.560 --> 0:15:46.100
PW (LC clinic)
OK, OK.

0:15:46.110 --> 0:15:51.60
PW (LC clinic)
So sort of like a, you know, you've done a lot of like the online stuff, did you get?

0:15:50.620 --> 0:15:54.130
DB
Well, if I of only had that one.

0:15:55.70 --> 0:15:55.720
PW (LC clinic)
Uh, you only had that.

0:15:55.730 --> 0:15:58.890
PW (LC clinic)
Did you speak to anybody before being referred to that dawn?

0:16:0.260 --> 0:16:2.950
DB
I think I spoke to my doctor.

0:16:4.70 --> 0:16:4.350
PW (LC clinic)
OK.

0:16:5.240 --> 0:16:8.490
DB
And they said right, you know, there was a long COVID clinic.

0:16:11.100 --> 0:16:11.400
PW (LC clinic)
OK.

0:16:8.500 --> 0:16:12.380
DB
I went out and I had no idea and a hat there.

0:16:12.390 --> 0:16:14.380
DB
I thought it was a long COVID clinic.

0:16:14.470 --> 0:16:28.670
DB
I didn't consider myself in long COVID at don't think that's probably why I didn't didn't notice, but as on on you know, because I I felt better when I came out after COVID umm.

0:16:28.920 --> 0:16:31.240
DB
And for probably the first.

0:16:32.880 --> 0:16:33.360
DB
Yeah.

0:16:34.620 --> 0:16:34.890
PW (LC clinic)
Hmm.

0:16:34.610 --> 0:16:37.500
DB
And then everything started to go wrong.

0:16:38.570 --> 0:16:39.430
PW (LC clinic)
OK, OK.

0:16:39.390 --> 0:16:42.610
DB
You know, things started happening and I'm thinking, what's this about them?

0:16:42.620 --> 0:16:43.600
DB
What's going on now?

0:16:43.610 --> 0:16:44.540
DB
Oh, God sake.

0:16:52.600 --> 0:16:52.970
PW (LC clinic)
Hmm.

0:16:44.670 --> 0:16:56.470
DB
And you know, it's really depressing when you've been someone that's always been healthy, fit, you know, can dance tonight and all of a sudden, like, you're like sleeping in the afternoon.

0:16:56.480 --> 0:16:58.200
DB
And and I've never done that in my life.

0:16:59.70 --> 0:16:59.540
PW (LC clinic)
Right.

0:16:59.670 --> 0:17:0.380
PW (LC clinic)
OK.

0:17:1.70 --> 0:17:1.920
PW (LC clinic)
So just going.

0:17:2.670 --> 0:17:2.790
DB
And.

0:17:1.930 --> 0:17:5.640
PW (LC clinic)
But so you it was your GP that referred you with that, would that be correct to?

0:17:5.10 --> 0:17:5.830
DB
Yeah, yeah.

0:17:5.650 --> 0:17:6.230
PW (LC clinic)
Yeah, OK.

0:17:6.860 --> 0:17:7.640
PW (LC clinic)
Uh.

0:17:8.220 --> 0:17:10.890
PW (LC clinic)
And can you remember, I mean, I know we've got a cast.

0:17:10.900 --> 0:17:12.690
PW (LC clinic)
Our minds back DBand and it's totally fine.

0:17:12.700 --> 0:17:17.190
PW (LC clinic)
Obviously if it's, you know, two different, remember just cause the amount of time in the way.

0:17:17.200 --> 0:17:24.510
PW (LC clinic)
But can you remember approximately or did it seem like it took a long time to get your sort of first appointment or was it fairly quick sort of thing?

0:17:23.960 --> 0:17:26.160
DB
A long time for everything came through.

0:17:25.240 --> 0:17:30.460
PW (LC clinic)
So, right, OK, any you know, any idea of an approximate amount of time? DBdon't.

0:17:30.470 --> 0:17:30.860
PW (LC clinic)
No worries.

0:17:30.870 --> 0:17:32.410
PW (LC clinic)
If if it's, you know, not possible to say.

0:17:31.690 --> 0:17:32.790
DB
Have no idea.

0:17:32.800 --> 0:17:34.830
DB
I've got no idea on on time.

0:17:35.490 --> 0:17:35.730
PW (LC clinic)
OK.

0:17:35.470 --> 0:17:35.860
DB
Umm.

0:17:36.50 --> 0:17:39.340
DB
And that's part of what my biggest problem is.

0:17:40.410 --> 0:17:42.320
DB
You know of remembering things.

0:17:42.330 --> 0:17:51.560
DB
Everything I now have to write down trying to remember days, times and and things I've got to do alarms on my my phone is constant.

0:17:51.770 --> 0:17:55.640
DB
You know things I've got to remember and I've never been like that.

0:17:56.280 --> 0:17:56.700
PW (LC clinic)
Right.

0:17:55.990 --> 0:17:57.500
DB
Ever say something?

0:17:57.890 --> 0:17:59.460
DB
That was three weeks ago that happened.

0:17:59.910 --> 0:18:1.470
DB
And this is what happened to you.

0:18:1.610 --> 0:18:3.420
DB
The, the, the and now I go.

0:18:3.670 --> 0:18:4.230
DB
Oh, no idea.

0:18:5.770 --> 0:18:6.80
PW (LC clinic)
Right.

0:18:5.440 --> 0:18:8.970
DB
We're not clone the look back on everything and go alright.

0:18:8.980 --> 0:18:9.500
DB
OK, hang on.

0:18:9.510 --> 0:18:10.700
DB
Yeah, I've got that bit there.

0:18:11.90 --> 0:18:16.760
DB
It's that is the most well, one of the most debilitating parts of all of this.

0:18:21.90 --> 0:18:21.430
PW (LC clinic)
Right.

0:18:17.10 --> 0:18:28.90
DB
That has really, really got to me and I find it grating to be so debilitated with, with remembering things.

0:18:29.390 --> 0:18:30.40
PW (LC clinic)
Yeah, absolutely.

0:18:30.50 --> 0:18:30.760
PW (LC clinic)
And DBwas that.

0:18:30.930 --> 0:18:35.790
PW (LC clinic)
Was that something you got help with at the at the time you have you got any ongoing sort of support for that?

0:18:36.430 --> 0:18:37.560
DB
Nope, nothing.

0:18:37.890 --> 0:18:38.400
DB
That's there.

0:18:38.140 --> 0:18:38.420
PW (LC clinic)
OK.

0:18:38.410 --> 0:18:39.860
DB
You dumped and left.

0:18:39.900 --> 0:18:43.160
DB
You know, no one told you this was what's gonna happen.

0:18:43.170 --> 0:18:44.580
DB
No one, I mean, I don't know.

0:18:44.590 --> 0:18:46.930
DB
They didn't know a lot, but I knew more than I knew.

0:18:48.420 --> 0:18:48.660
PW (LC clinic)
Yeah.

0:18:48.320 --> 0:18:53.0
DB
And I've just find out on the way I'm thinking of.

0:18:51.840 --> 0:18:56.510
PW (LC clinic)
So yeah, so dawn, but just to pick that up a bit more.

0:18:56.520 --> 0:19:4.490
PW (LC clinic)
So you know was at the beginning of it, sort of. You know of your first contact with the clinic. Did you did.

0:19:4.500 --> 0:19:15.400
PW (LC clinic)
You know you have any recollection of anybody explaining the process or the pathway through the assessment and what was gonna happen or is that something that just as far as you concerned?

0:19:16.170 --> 0:19:18.10
PW (LC clinic)
And it didn't happen.

0:19:18.20 --> 0:19:19.250
PW (LC clinic)
So therefore you were you.

0:19:19.260 --> 0:19:20.220
PW (LC clinic)
You were fairly in the dark.

0:19:20.230 --> 0:19:26.200
PW (LC clinic)
I just wanted if you know, did you have anything like an explanation of what was gonna happen if you went through the the long COVID clinic, anything like that?

0:19:27.330 --> 0:19:28.280
DB
Uh, no.

0:19:28.350 --> 0:19:29.60
DB
I just.

0:19:29.150 --> 0:19:29.470
PW (LC clinic)
Right.

0:19:29.70 --> 0:19:30.820
DB
I just didn't say right.

0:19:30.830 --> 0:19:32.40
DB
You know you're in long coat.

0:19:32.50 --> 0:19:33.560
DB
There was a long COVID clinic at all.

0:19:33.570 --> 0:19:33.920
DB
OK.

0:19:33.930 --> 0:19:35.220
DB
Then do you want to join it?

0:19:35.230 --> 0:19:37.90
DB
Well, yeah, OK, let's see what it's about.

0:19:38.10 --> 0:19:38.640
PW (LC clinic)
OK.

0:19:44.330 --> 0:19:44.670
DB
Hmm.

0:19:38.990 --> 0:19:48.320
PW (LC clinic)
And and apart from this, the the online sort of a session that you had, you know where were you given any other treatments or any other options for for, for care?

0:19:49.460 --> 0:19:50.530
DB
No, nothing.

0:19:50.650 --> 0:19:51.350
PW (LC clinic)
No. OK.

0:19:50.620 --> 0:19:55.510
DB
The only thing I was one of the nurses there, I don't even know who she was.

0:19:55.520 --> 0:20:3.690
DB
Now, she said, it might help you to come in and see where you were in the hospital so.

0:20:3.760 --> 0:20:3.960
PW (LC clinic)
Right.

0:20:11.990 --> 0:20:12.520
PW (LC clinic)
Right.

0:20:12.570 --> 0:20:13.720
PW (LC clinic)
OK, what's?

0:20:3.780 --> 0:20:22.500
DB
But well, the fact that my memory is gone and I was in a coma for seven weeks, that is actually didn't help me because I the only thing it did was one of the doctors came up and I said to him, I know you can't do it said because you were in a coma, I said.

0:20:22.510 --> 0:20:23.530
DB
But I recognize your voice.

0:20:25.690 --> 0:20:26.170
PW (LC clinic)
Right.

0:20:25.360 --> 0:20:31.320
DB
And he said wait and that was at the on that bit there I'd gone all through these other bits.

0:20:31.330 --> 0:20:32.140
DB
So they were saying, right?

0:20:32.150 --> 0:20:32.640
DB
You were here.

0:20:32.650 --> 0:20:33.510
DB
You were here and I'm thinking.

0:20:33.520 --> 0:20:34.760
DB
Well, I've got a clue where I was.

0:20:45.660 --> 0:20:45.920
PW (LC clinic)
Yeah.

0:20:35.510 --> 0:20:50.960
DB
You know they're they're there and it wasn't till we got to the the this one room which had just looked like Star Wars, you know, everything's cloak machines everywhere as you were tied up to all of this and that doctor came along.

0:20:51.610 --> 0:20:55.0
DB
And I after I heard his voice, something triggered.

0:21:0.870 --> 0:21:1.190
PW (LC clinic)
Hmm.

0:20:55.530 --> 0:21:7.380
DB
I don't know what it was, but I've got really emotional and said sorry, I've got to go because I to tears, but I've never, never shed so many tears since happiness.

0:21:10.780 --> 0:21:10.960
DB
No.

0:21:17.190 --> 0:21:18.200
DB
It remembered.

0:21:8.210 --> 0:21:19.600
PW (LC clinic)
So you don't you don't remember him per say, but there's something obviously, that, that that was, you know, part of your memory that was, uh, you know active whilst you were, yeah.

0:21:21.800 --> 0:21:22.200
PW (LC clinic)
Wow.

0:21:19.490 --> 0:21:28.950
DB
I remembered his voice doing the the the when I was in the coma, I heard foreign.

0:21:29.480 --> 0:21:41.580
DB
Well, I'm in this camera with this hallucinations and in the hallucinations was part of someone asking to take my blood because they torture apparently.

0:21:41.740 --> 0:21:45.850
DB
And someone else was, uh, doing something else.

0:21:45.860 --> 0:21:51.930
DB
And you know, and and I was on a slope at some stage and then I'm fighting to save a monkey in a fish.

0:21:51.940 --> 0:21:54.90
DB
And I can remember all of that.

0:21:54.250 --> 0:21:55.580
DB
But I also remember someone going.

0:21:55.590 --> 0:21:57.10
DB
We're just gonna whip your lips.

0:21:57.240 --> 0:21:58.970
DB
We're just gonna wipe your eyes.

0:21:59.680 --> 0:22:0.40
PW (LC clinic)
Right.

0:22:4.150 --> 0:22:4.450
PW (LC clinic)
Hmm.

0:21:59.300 --> 0:22:7.720
DB
So that a lot of Filipinos there to I was at a Japanese restaurant, but these Filipinos, obviously it's just.

0:22:8.420 --> 0:22:9.530
DB
Another language.

0:22:9.970 --> 0:22:10.270
PW (LC clinic)
Yeah.

0:22:9.640 --> 0:22:14.500
DB
So you know, it would sounded to me like they were Japanese, but we was just a foreign language.

0:22:15.60 --> 0:22:15.440
PW (LC clinic)
Wow.

0:22:16.90 --> 0:22:22.700
DB
So I I heard that so it proves people can hear during a coma.

0:22:23.550 --> 0:22:24.360
PW (LC clinic)
Absolutely.

0:22:24.370 --> 0:22:27.390
PW (LC clinic)
Crikey, what an experienced DByou've been through there.

0:22:26.60 --> 0:22:30.800
DB
Yeah, I put that together until about two years later.

0:22:32.380 --> 0:22:35.430
DB
It took that long for me to realize.

0:22:35.860 --> 0:22:36.460
DB
Hang on a minute.

0:22:37.820 --> 0:22:39.50
DB
Maybe that's to do with it.

0:22:39.740 --> 0:22:41.120
PW (LC clinic)
So that, yeah, that those. Yeah.

0:22:39.840 --> 0:22:45.440
DB
And I, Claire, you need the help you when you come out, you need someone to say.

0:22:45.450 --> 0:22:46.300
DB
Right, OK.

0:22:46.370 --> 0:22:47.720
DB
Do you remember anything?

0:22:51.610 --> 0:22:51.970
PW (LC clinic)
Hmm.

0:22:48.230 --> 0:22:53.50
DB
And they put the two and two together for you to go, right.

0:22:53.60 --> 0:22:53.820
DB
That's it.

0:22:54.50 --> 0:22:55.660
DB
That's another thing off your mic.

0:22:55.670 --> 0:22:57.700
DB
Should thinking why there a minute?

0:22:57.950 --> 0:22:59.960
DB
I remember that I was that to do with that.

0:22:59.970 --> 0:23:4.940
DB
With that, you know, and you kind of think when you're in that comma, what's that screaming and shouting?

0:23:4.950 --> 0:23:6.120
DB
Cause it my dream.

0:23:6.130 --> 0:23:6.530
DB
I was.

0:23:6.540 --> 0:23:7.940
DB
I was fighting everybody.

0:23:9.20 --> 0:23:9.250
PW (LC clinic)
Umm.

0:23:8.270 --> 0:23:12.830
DB
You know, I was actually screaming this and it's all these little things that you want to know.

0:23:13.870 --> 0:23:17.80
DB
Umm, when you've been in there that long?

0:23:17.150 --> 0:23:20.640
DB
I mean, I've I parted from when my daughter was 12.

0:23:21.270 --> 0:23:22.680
DB
She's now 35.

0:23:23.420 --> 0:23:26.410
PW (LC clinic)
Umm, right.

0:23:23.250 --> 0:23:28.0
DB
I live for once and then wants to have wants for lung infection.

0:23:28.810 --> 0:23:29.190
PW (LC clinic)
Right.

0:23:37.910 --> 0:23:38.190
PW (LC clinic)
Umm.

0:23:29.770 --> 0:23:53.930
DB
Nothing ever since or hip hip reserves in, but nothing, and then suddenly to be that much in there, you know, and it's such a depressing place to be at any given stage, let alone when you you can't see anybody and you your family can't come in and you know my daughter was facetiming me all the time and apparently I'm talking to her and I don't remember a thing of that.

0:23:54.810 --> 0:23:55.750
PW (LC clinic)
Wow, OK.

0:23:55.450 --> 0:23:59.300
DB
But you know, do you want that help when you come out?

0:23:59.310 --> 0:24:5.290
DB
So the long COVID bit needs to be from the start, not two years down the line.

0:24:6.440 --> 0:24:7.10
PW (LC clinic)
I see.

0:24:7.20 --> 0:24:7.870
PW (LC clinic)
So yeah.

0:24:7.880 --> 0:24:18.250
PW (LC clinic)
OK, so there was just that sort of like disjunct between following on the care, you know, from the hospital to into the the community based clinic.

0:24:18.500 --> 0:24:20.870
PW (LC clinic)
Yeah, that makes sense, dawn.

0:24:20.880 --> 0:24:21.70
PW (LC clinic)
How?

0:24:24.550 --> 0:24:24.710
DB
The.

0:24:21.80 --> 0:24:24.950
PW (LC clinic)
How come you know the very idea of long COVID as a condition?

0:24:25.160 --> 0:24:28.160
PW (LC clinic)
Do you feel confident in the diagnosis you've been given?

0:24:30.550 --> 0:24:33.520
DB
I don't, to be honest with you, there's so much that's gone wrong.

0:24:33.530 --> 0:24:38.570
DB
I've just had a things through today telling me what I've got wrong and it's scary.

0:24:39.690 --> 0:24:39.980
PW (LC clinic)
Hmm.

0:24:39.770 --> 0:24:40.300
DB
Absolutely.

0:24:40.310 --> 0:24:46.810
DB
Very case I never had this problem before and in all honesty they put me in a COVID wall and I didn't have COVID.

0:24:47.760 --> 0:24:48.140
PW (LC clinic)
Right.

0:24:49.750 --> 0:24:50.950
DB
And that's how I got COVID.

0:24:52.280 --> 0:24:52.740
PW (LC clinic)
OK.

0:24:54.90 --> 0:24:55.700
DB
So that makes me quite angry.

0:24:57.600 --> 0:25:4.570
DB
The that could have happened if I had three false false, false tests saying no.

0:25:10.900 --> 0:25:11.370
PW (LC clinic)
Right.

0:25:4.580 --> 0:25:13.380
DB
I hadn't got it, but they put me straight in the cover to all, but I didn't go in with COVID, and that makes me angry that they did that.

0:25:19.400 --> 0:25:19.860
PW (LC clinic)
Right. So.

0:25:14.400 --> 0:25:23.180
DB
Umm, you know, just for the sake of putting someone in another COVID board, you know, and I theories on it, whether they're right or wrong.

0:25:23.910 --> 0:25:29.290
DB
Umm on why they put people in code towards why they did so many tests, etcetera, etcetera.

0:25:29.760 --> 0:25:30.370
PW (LC clinic)
Yeah, it's a.

0:25:30.420 --> 0:25:34.150
PW (LC clinic)
There's a lot of there's a definitely a lot of stones left unturned.

0:25:34.640 --> 0:25:34.950
PW (LC clinic)
Door.

0:25:34.960 --> 0:25:35.240
PW (LC clinic)
Not yes.

0:25:34.950 --> 0:25:37.380
DB
So yeah, and I am awake.

0:25:38.530 --> 0:25:38.980
PW (LC clinic)
Right.

0:25:39.50 --> 0:25:39.430
PW (LC clinic)
Yeah.

0:25:39.440 --> 0:25:40.950
PW (LC clinic)
OK, I'm with you and.

0:25:41.510 --> 0:25:43.280
DB
So and that sort of thing.

0:25:43.880 --> 0:25:46.130
DB
Umm, you know, I I just.

0:25:46.140 --> 0:25:47.150
DB
It makes me very angry.

0:25:47.160 --> 0:25:53.450
DB
In fact, I actually have emailed them today to the hospital to say I want an explanation as to why you did it.

0:25:54.60 --> 0:25:57.580
DB
Why didn't you just think about me and just say, well, let's see how she gets on the next few days.

0:25:58.480 --> 0:25:58.830
PW (LC clinic)
Hmm.

0:25:58.340 --> 0:26:1.630
DB
If I'd have been sorted out there, there's all she got the symptoms.

0:26:4.930 --> 0:26:5.250
PW (LC clinic)
Right.

0:26:6.310 --> 0:26:6.690
PW (LC clinic)
OK.

0:26:8.150 --> 0:26:8.790
PW (LC clinic)
So yeah.

0:26:1.640 --> 0:26:10.560
DB
I didn't have the symptoms at all, but you know this is this is what's happened after all this.

0:26:10.570 --> 0:26:12.860
DB
And it's now sort of three years down the line.

0:26:12.870 --> 0:26:13.220
DB
And I'm.

0:26:13.230 --> 0:26:14.220
DB
I'm like, why isn't?

0:26:14.230 --> 0:26:15.350
DB
I mean, this isn't right.

0:26:22.150 --> 0:26:22.350
PW (LC clinic)
Umm.

0:26:15.360 --> 0:26:26.790
DB
This is so much going on and I think cause since last August I've been absolutely nothing but unwell and when I hear into it I'm starting to get really angry about it so.

0:26:26.640 --> 0:26:26.980
PW (LC clinic)
OK.

0:26:28.450 --> 0:26:33.10
PW (LC clinic)
So something that you're thinking about following up basically, yeah, quite strongly by sounds of things done.

0:26:31.600 --> 0:26:33.980
DB
Yeah. Yeah, absolutely.

0:26:33.990 --> 0:26:34.560
DB
Yeah.

0:26:33.70 --> 0:26:35.380
PW (LC clinic)
Yeah. OK.

0:26:34.630 --> 0:26:37.680
DB
Yeah, because I feel it was unnecessary.

0:26:39.220 --> 0:26:39.950
DB
You understand that?

0:26:39.960 --> 0:26:41.530
DB
Didn't know quite a lot was going on.

0:26:41.540 --> 0:26:46.670
DB

But you know you don't dump someone in somewhere that they haven't got something and then give it to him.

0:26:47.590 --> 0:26:48.110
PW (LC clinic)
Yeah.

0:26:48.170 --> 0:27:3.510
PW (LC clinic)
Well, it's, you know, I can I can really obviously see you know from well exactly what you're saying really went into hospital and not with COVID and then you whatever you went in with initially is enough.

0:27:5.500 --> 0:27:5.660
DB
Yeah.

0:27:3.520 --> 0:27:9.440
PW (LC clinic)
Right, you should be dealing with that and the result of going somewhere where essentially thing your your health should be being improved.

0:27:9.450 --> 0:27:12.10
PW (LC clinic)
You actually get worse by contracting COVID.

0:27:13.30 --> 0:27:13.210
DB
Umm.

0:27:12.20 --> 0:27:22.140
PW (LC clinic)
Absolutely, dawn, you know, sorry to sort of like a swing us back to kind of more more let's say, more mundane questions, shall we about the clinic.

0:27:22.250 --> 0:27:30.980
PW (LC clinic)
But I just wanted to sort of like if it's possible and again I totally appreciate that we're trying to sort of cast our minds back into a good few years ago.

0:27:30.990 --> 0:27:37.850
PW (LC clinic)
But the very first appointment that you had with the clinic, you remember all that involved you have some form of.

0:27:39.710 --> 0:27:50.890
PW (LC clinic)
Did you go through some some form of, you know, a questionnaire or anything like that where they might have asked you sort of details of what you've been experiencing symptom wise and stuff like that was you remember much about your first?

0:27:50.380 --> 0:27:52.810
DB
I think I think you remember something.

0:27:52.820 --> 0:27:54.10
DB
You just brought it to my mind.

0:27:54.20 --> 0:28:7.400
DB
Actually I seem to remember feeling something in from someone I don't know who I can't remember now on what was going on and I think I actually wrote like, you know, war and peace.

0:28:7.410 --> 0:28:21.800
DB
I think you know to put it out and explain to them what it was so that I got the right not treatment but right questions or right person dealing with it.

0:28:22.320 --> 0:28:22.580
PW (LC clinic)
Yeah.

0:28:23.260 --> 0:28:24.470
DB
And obviously it didn't.

0:28:25.770 --> 0:28:26.190
PW (LC clinic)
Right.

0:28:24.680 --> 0:28:27.340
DB
But they go, umm, you know?

0:28:27.490 --> 0:28:27.780
DB
Oops.

0:28:27.790 --> 0:28:31.980
DB
Wrong, but yeah, like getting rid of her.

0:28:32.90 --> 0:28:37.770
DB
She's trouble, but it's sit with the quiet ones that don't ask questions.

0:28:38.190 --> 0:28:42.30
DB
But yeah, but some you know, it's.

0:28:43.330 --> 0:28:49.80
DB
I remember feeling something in umm, but I have no idea what it was, no.

0:28:48.850 --> 0:28:50.540
PW (LC clinic)
OK, OK.

0:28:50.610 --> 0:29:8.800
PW (LC clinic)
Is it possible to say sort of like you know just receiving that first appointment and know that knowing that you're having it, you know, I just wondered if that sort of how you how you felt at the time about actually getting appointment that was, you know gonna be focused on uh, you know, suffering with long COVID did it make you feel at a particular way just to have the appointment?

0:29:9.740 --> 0:29:10.880
DB
Well, I just thought, oh, right.

0:29:10.890 --> 0:29:13.250
DB
OK, let's see what this has got to offer.

0:29:14.140 --> 0:29:14.500
PW (LC clinic)
OK.

0:29:14.30 --> 0:29:14.790
DB
You know, is it?

0:29:14.890 --> 0:29:16.860
DB
Is this gonna benefit me?

0:29:17.30 --> 0:29:17.290
PW (LC clinic)
OK.

0:29:16.970 --> 0:29:18.400
DB
I they gonna.

0:29:18.410 --> 0:29:23.450
DB
I'm I'm trying to think what questions I would ask, you know what they would be asking.

0:29:24.850 --> 0:29:25.960
DB
That was a basement.

0:29:28.70 --> 0:29:31.160
PW (LC clinic)
And you remember, can you remember who?

0:29:31.170 --> 0:29:37.660
PW (LC clinic)

The perhaps the first person was with, you know, once you've been referred to the long COVID clinic that you, you have spoken to no problem.

0:29:37.670 --> 0:29:39.930
PW (LC clinic)
If you can't remember because you know I understand we're talking.

0:29:39.190 --> 0:29:41.0
DB
Well, a little bit to meeting thing.

0:29:41.750 --> 0:29:42.40
PW (LC clinic)
Yeah.

0:29:42.50 --> 0:29:43.870
PW (LC clinic)
So once you got referred from your doctors.

0:29:42.960 --> 0:29:47.530
DB
It was like the lady that did the team meeting thing.

0:29:48.80 --> 0:29:48.340
PW (LC clinic)
Right.

0:29:48.350 --> 0:29:49.280
PW (LC clinic)
That was the first person.

0:29:49.290 --> 0:29:51.340
PW (LC clinic)
Yeah, right. OK.

0:29:50.100 --> 0:29:51.430
DB
Yeah, yeah.

0:29:51.500 --> 0:30:0.40
DB
I I just literally filled this form in and pretty sure it's a form of filled in about the long COVID and that was it.

0:30:0.50 --> 0:30:3.130
DB
And then obviously I get a teams thing come up.

0:30:3.230 --> 0:30:4.110
DB
OK, fine.

0:30:5.800 --> 0:30:6.60
PW (LC clinic)
OK.

0:30:6.490 --> 0:30:9.740
DB
That's about the only thing actually that I can remember.

0:30:10.280 --> 0:30:10.780
PW (LC clinic)
Yeah.

0:30:10.830 --> 0:30:11.940
PW (LC clinic)
Yeah and.

0:30:11.80 --> 0:30:12.580
DB
I'm crazy trying.

0:30:12.590 --> 0:30:13.490
DB
That's I'm a problem.

0:30:13.500 --> 0:30:13.970
DB
It's all gone.

0:30:15.840 --> 0:30:16.690
PW (LC clinic)
And what?

0:30:16.700 --> 0:30:17.310
PW (LC clinic)
How long does that last?

0:30:17.320 --> 0:30:17.850
PW (LC clinic)
About an hour.

0:30:17.860 --> 0:30:18.770
PW (LC clinic)
Was it something like that or?

0:30:22.760 --> 0:30:23.120
PW (LC clinic)
Right.

0:30:19.630 --> 0:30:24.220
DB
Umm, I don't think even that really might be about, you know.

0:30:24.70 --> 0:30:24.500
PW (LC clinic)
OK.

0:30:25.270 --> 0:30:29.70
PW (LC clinic)
So yeah, so.

0:30:25.280 --> 0:30:29.850
DB
So it's about like getting in but not normal for me.

0:30:29.860 --> 0:30:30.470
DB
Normal for me.

0:30:31.880 --> 0:30:39.800
PW (LC clinic)
So more more importantly down then, maybe this is kind of you know after you had that that first you know and we might have touched it a bit because it might be the same.

0:30:39.810 --> 0:30:44.130
PW (LC clinic)
But how did you feel immediately after that initial appointment?

0:30:44.140 --> 0:30:48.350
PW (LC clinic)
If you like the time when you did the group the virtual group.

0:30:49.510 --> 0:30:50.990
DB
I thought bloody waste of time.

0:30:51.890 --> 0:30:52.310
PW (LC clinic)
Right.

0:30:52.390 --> 0:30:53.190
PW (LC clinic)
OK. Yeah.

0:30:51.0 --> 0:30:54.170
DB
That was the exactly myself.

0:30:55.750 --> 0:30:56.160
PW (LC clinic)
Yeah.

0:30:54.180 --> 0:30:56.170
DB
What waste time? What?

0:30:57.120 --> 0:30:57.850
DB
Back in my life.

0:30:58.300 --> 0:31:0.480
DB
Yeah, but.

0:30:56.230 --> 0:31:3.860
PW (LC clinic)
And so after after that after, after that dawn, did you have any ongoing contact with him or it was literally that was that.

0:31:3.910 --> 0:31:6.830
PW (LC clinic)
Yeah, that one session then, and then you didn't see anybody again, is it?

0:31:6.840 --> 0:31:8.570
PW (LC clinic)
Would that be a fair thing to say?

0:31:8.30 --> 0:31:9.720
DB
That was one second, they said.

0:31:9.730 --> 0:31:12.910
DB
They taken me off the long COVID thing because I hadn't answered something I said.

0:31:12.920 --> 0:31:14.740
DB
I've never had anything they said.

0:31:14.750 --> 0:31:18.300
DB
We sent it to you, I said look, I'm looking through all my emails now and I've got nothing.

0:31:19.410 --> 0:31:20.510
DB
Absolutely nothing comes through.

0:31:21.200 --> 0:31:21.770
PW (LC clinic)
Right.

0:31:21.840 --> 0:31:22.400
PW (LC clinic)
And what what?

0:31:22.410 --> 0:31:23.240
PW (LC clinic)
What was it they looking for?

0:31:22.610 --> 0:31:29.610
DB
Whether but they said that they had sent for another long COVID chat.

0:31:30.720 --> 0:31:31.80
PW (LC clinic)
Right.

0:31:30.410 --> 0:31:33.910
DB
Umm, but I got I had nothing come through.

0:31:33.920 --> 0:31:42.670
DB
So they said as soon as you didn't give your consent and and join would take you off the long COVID so ring them and said, what's that about?

0:31:43.160 --> 0:31:46.170
DB
Haven't had anything they said we sent it on such and such a day.

0:31:46.180 --> 0:31:46.640
DB
That's alright.

0:31:46.650 --> 0:31:49.730
DB
Let me look through all my emails and I look through that date.

0:31:49.740 --> 0:31:50.750
DB
So I said, I've got nothing.

0:31:50.820 --> 0:31:51.950
DB
Absolutely nothing come true.

0:31:52.360 --> 0:31:53.590
DB
I've looked at my junk.

0:31:53.680 --> 0:31:55.530
DB
Nothing in there at all, I said.

0:31:55.540 --> 0:32:1.350
DB
So either it's just as it's falling in the big black hole somewhere, or you know no one.

0:32:1.360 --> 0:32:3.50
DB
Someone's got to send it, I said.

0:32:3.60 --> 0:32:8.910
DB
But I haven't got nothing I said, so I want it to be back on the on COVID thing that because.

0:32:8.550 --> 0:32:11.0
PW (LC clinic)
So that was the that was the end of that really.

0:32:11.10 --> 0:32:15.110
PW (LC clinic)
Then was it or did you get put back back, you know, on into the clinic or?

0:32:14.670 --> 0:32:17.210
DB
The eventually they put me back on.

0:32:17.880 --> 0:32:18.320
PW (LC clinic)
OK.

0:32:19.950 --> 0:32:20.100
DB
No.

0:32:18.330 --> 0:32:20.780
PW (LC clinic)
And then did you have any more contact?

0:32:21.100 --> 0:32:21.510
PW (LC clinic)
No.

0:32:21.740 --> 0:32:23.800
PW (LC clinic)
OK alright.

0:32:24.910 --> 0:32:26.160
DB
I'll pick up in blacklisted.

0:32:29.410 --> 0:32:31.670
PW (LC clinic)
So I mean this question sort of.

0:32:34.250 --> 0:32:34.540
PW (LC clinic)
Yeah.

0:32:34.550 --> 0:32:36.800
PW (LC clinic)
We've kind of touched it really, but I'll ask you anyway.

0:32:37.10 --> 0:32:37.190
DB
Yeah.

0:32:36.850 --> 0:32:41.130
PW (LC clinic)
Dawn, you know, was communication of your assessment and treatment make clear.

0:32:45.40 --> 0:32:50.160
DB
In what way with with the teams or from the doctor?

0:32:50.830 --> 0:32:53.830
PW (LC clinic)
Yeah, it really any any of it really what you know, I suppose I suppose was there.

0:32:54.640 --> 0:32:58.480
PW (LC clinic)
Uh, it seems like there was quite a lot of miscommunication there.

0:32:58.490 --> 0:33:1.110
PW (LC clinic)
Is it is probably a better route to go down, isn't it?

0:33:1.120 --> 0:33:1.510
PW (LC clinic)
You know.

0:33:1.520 --> 0:33:3.500
PW (LC clinic)
So umm and.

0:33:1.750 --> 0:33:9.900
DB
Yeah, very little location about when, from when I came out, there was very little, if any, communication about anything.

0:33:11.360 --> 0:33:11.700
PW (LC clinic)
Right.

0:33:11.220 --> 0:33:15.580
DB
You know, I find, as I say, dumped and left to get on with it.

0:33:16.340 --> 0:33:16.770
PW (LC clinic)
Hmm.

0:33:16.460 --> 0:33:17.570
DB
Uh, you know?

0:33:17.580 --> 0:33:21.430
DB
And that was it, basically, yeah.

0:33:21.90 --> 0:33:24.300
PW (LC clinic)
So it would be fair to say that you you didn't really have.

0:33:24.390 --> 0:33:25.730
PW (LC clinic)
It sounds like, you know.

0:33:25.810 --> 0:33:28.550
PW (LC clinic)
Did you feel as if you had any opportunity to ask questions or?

0:33:29.340 --> 0:33:30.490
DB
Which be honest with you.

0:33:30.540 --> 0:33:31.830
DB
I really wasn't.

0:33:36.370 --> 0:33:36.780
PW (LC clinic)
Hmm.

0:33:31.880 --> 0:33:39.730
DB
I mean, it's taken it took me a good couple of years for all to sink in, you know, even seeing the pictures.

0:33:39.740 --> 0:33:44.380
DB
Because my daughter FaceTime, but took a picture at each time of me.

0:33:44.290 --> 0:33:48.270
PW (LC clinic)
Yeah, this is during your hospital, yeah.

0:33:45.590 --> 0:33:49.90
DB
And yeah, let's see what she said.

0:33:54.690 --> 0:33:54.900
PW (LC clinic)
Umm.

0:33:49.100 --> 0:33:57.400
DB
Mom, you're never gonna believe when I tell you what happened, you won't believe it, but could not coma you.

0:33:57.450 --> 0:34:1.940
DB
You won't believe it, so I need to take this picture to show you just how ill you were.

0:34:1.950 --> 0:34:4.580
DB
So you understand, Christian.

0:34:4.800 --> 0:34:5.180
DB
Knows me.

0:34:5.190 --> 0:34:6.90
DB
I'll be going UHB.

0:34:7.180 --> 0:34:9.580
PW (LC clinic)
Yeah. Wow.

0:34:6.100 --> 0:34:10.680
DB
Alright, right now, you know, that's what.

0:34:10.730 --> 0:34:12.160
DB
That's why I am.

0:34:12.230 --> 0:34:14.660
DB
I'm because I I haven't been that ill before.

0:34:14.670 --> 0:34:19.50
DB
I've never been in a coma in my life and I didn't even know I was in it.

0:34:19.980 --> 0:34:20.370
PW (LC clinic)
Right.

0:34:20.80 --> 0:34:25.60
DB
You know, I lost probably a good three weeks of not having a clue what was going on.

0:34:26.980 --> 0:34:30.880
DB
And I was in there three months, but actually probably longer than than three weeks.

0:34:31.370 --> 0:34:33.780
DB
You know it must be 4-5 weeks before I knew.

0:34:33.790 --> 0:34:35.900
DB
I mean, when I woke up, I just thought I was in the next day.

0:34:36.880 --> 0:34:37.250
PW (LC clinic)
Wow.

0:34:37.300 --> 0:34:37.640
PW (LC clinic)
OK.

0:34:37.650 --> 0:34:38.30
PW (LC clinic)
What?

0:34:38.40 --> 0:34:41.210
PW (LC clinic)
What a horrible thing to go through DBand obviously.

0:34:41.810 --> 0:34:44.990
DB
Well, actually I think it was worse for my family.

0:34:45.940 --> 0:34:49.330
DB
Umm, you know, they rang my daughter 3:00 o'clock and said look, you better get ready.

0:34:49.340 --> 0:34:50.490
DB
Your mum's not gonna make it.

0:34:51.490 --> 0:34:52.800
DB
And I said, well, what's the chances?

0:34:52.810 --> 0:34:54.920
DB
They said less than 10% this, she said.

0:34:54.930 --> 0:34:56.380
DB
Well, someone's gotta survive 10%.

0:34:56.390 --> 0:34:58.660
DB
You don't know my mum. And here I am.

0:34:58.890 --> 0:35:6.860
DB
But you know, Even so, with with all of that, I would never have believed that I'd actually been the ill.

0:35:7.690 --> 0:35:8.100
PW (LC clinic)
Right.

0:35:10.940 --> 0:35:11.880
DB
Yeah, yeah.

0:35:8.110 --> 0:35:12.910
PW (LC clinic)
So seeing that picture was really quite a I I I opening, you know, yeah.

0:35:14.60 --> 0:35:24.850
DB
Lately, when I look at it and I'll think it's even look like me, you know, I'll look at a bloated right out case they couldn't get the the liquid off my lungs or something, you know.

0:35:30.90 --> 0:35:30.400
PW (LC clinic)
Hmm.

0:35:24.860 --> 0:35:34.150
DB
And The thing is, I called the 1113 weeks previous at the start and such an I am drenching something is wrong.

0:35:34.280 --> 0:35:35.670
DB
Can't find anything wrong with you.

0:35:36.0 --> 0:35:41.910
DB
Bloods are OK that that the, the, whatever that actually didn't take bloods, they just said we checked all your vitals and everything. Fine.

0:35:42.200 --> 0:35:43.700
DB
Ohh it's something's not right.

0:35:44.590 --> 0:35:49.970
DB
Second week at the same look, I'm still really trenching of night time absolutely soaked.

0:35:50.680 --> 0:35:52.150
DB
There's I don't can't find anything wrong.

0:35:52.380 --> 0:35:54.870
DB
Third week it was my pulse was 146.

0:35:54.960 --> 0:35:55.130
DB
I went.

0:35:55.140 --> 0:35:56.230
DB
It was it, good or bad?

0:35:56.320 --> 0:35:57.160
DB
It's catastrophic.

0:35:57.170 --> 0:36:0.700
DB
We're gonna move here now, and that was the start of the whole thing.

0:36:1.990 --> 0:36:11.410
DB
So it's it's when people get ill that it's making sure that they they get the treatment for the first week.

0:36:11.420 --> 0:36:15.830
DB
If I had got that treatment first week, I wouldn't have been in the state I'm in now.

0:36:17.190 --> 0:36:17.880
PW (LC clinic)
Yeah.

0:36:18.70 --> 0:36:18.840
PW (LC clinic)
So it could have been.

0:36:18.850 --> 0:36:20.370
PW (LC clinic)
It could, yeah, but.

0:36:18.80 --> 0:36:22.410
DB
This good bit of possibly possibly.

0:36:22.640 --> 0:36:22.880
PW (LC clinic)
Yeah.

0:36:22.470 --> 0:36:26.20
DB
I'm not a doctor and I'm not quite sure, but possibly that could have been avoided.

0:36:27.20 --> 0:36:27.220
PW (LC clinic)
Yeah.

0:36:27.560 --> 0:36:28.970
DB
You know all this?

0:36:32.590 --> 0:36:32.880
PW (LC clinic)
Umm.

0:36:29.60 --> 0:36:38.450
DB
What I'm going through now and that's the bit that makes me really cross, yeah, so but going on from that, you know the pictures and they give seven day diary.

0:36:40.940 --> 0:36:47.340
DB
And that's seven day diary they tell you who's looking after you and what they've said to you and what you're like that day.

0:36:47.310 --> 0:36:47.810
PW (LC clinic)
Right.

0:36:48.700 --> 0:36:51.220
DB
I it's really, really emotional.

0:36:52.130 --> 0:36:52.420
DB
Really.

0:36:52.370 --> 0:36:54.20
PW (LC clinic)
Yeah, absolutely.

0:36:53.870 --> 0:36:59.0
DB
Yeah, I mean you, I don't know what I was doing that day, but they tell me, you know, you opened your eyes.

0:36:59.10 --> 0:37:0.590
DB
You're smiling, you know.

0:37:0.600 --> 0:37:4.330
DB
And actually one of our friends was a nurse and she she was looking after me at one stage.

0:37:4.840 --> 0:37:5.220
PW (LC clinic)
Right.

0:37:4.900 --> 0:37:6.560
DB
So that was really nice.

0:37:6.570 --> 0:37:7.930
DB
I mean, I used to do karate.

0:37:8.300 --> 0:37:13.720
DB
OK, there now and I'm like what you know, and I just haven't been doing that much.

0:37:13.730 --> 0:37:15.330
DB
I missed tournaments and things like that.

0:37:16.480 --> 0:37:18.410
DB
Yeah, because I just haven't got the energy.

0:37:19.300 --> 0:37:19.990
PW (LC clinic)
Yeah.

0:37:20.80 --> 0:37:20.540
DB
You know, I'm.

0:37:20.140 --> 0:37:21.820
PW (LC clinic)
And that's dawn, is that status?

0:37:21.830 --> 0:37:26.80
PW (LC clinic)
You know what you're saying is it's still something that is, is that a symptom that stayed the same?

0:37:27.780 --> 0:37:28.40
PW (LC clinic)
Umm.

0:37:26.860 --> 0:37:28.170
DB
Yeah, it's raw.

0:37:28.910 --> 0:37:29.250
PW (LC clinic)
Hmm.

0:37:28.560 --> 0:37:31.830
DB
Really raw, you know? Umm.

0:37:36.770 --> 0:37:36.940
DB
Yes.

0:37:29.910 --> 0:37:41.850
PW (LC clinic)
OK, Dawn, this might be a good a better you know, so this is actually just to let you know time wise you know this is the last section here and I think this is gonna be but definitely would have touched on some of it dawn.

0:37:42.200 --> 0:37:42.380
DB
Yeah.

0:37:41.860 --> 0:37:51.730
PW (LC clinic)
But just to kind of like ask you I suppose more formally, if you like, you know, so this bit is about, you know what what you think you know could have been better.

0:37:51.780 --> 0:37:55.310
PW (LC clinic)
So what kind of improvements do you think could be made?

0:37:55.320 --> 0:38:2.520
PW (LC clinic)
I mean obviously mentioned that that, you know there was that sort of you felt like you've been dumped after coming out of hospital.

0:38:2.840 --> 0:38:3.70
DB
Umm.

0:38:16.590 --> 0:38:16.790
DB
Yeah.

0:38:28.0 --> 0:38:28.160
DB
Yeah.

0:38:2.650 --> 0:38:33.270
PW (LC clinic)
So sort of like a bit more sort of what would you say a joint up joining up that care, so we kind of mentioned that as a as an issue, right that there was this sort of like you know you were left and then you only picked up the the clinic later on what what other kind of things you know in hindsight now looking back could have been improved obviously we mentioned the presentation as well that you thought was lacking should we say yeah, so we've mentioned those two just anything else that you think you know actually this this could be?

0:38:39.190 --> 0:38:39.470
PW (LC clinic)
Hmm.

0:38:33.830 --> 0:38:44.460
DB
When when people come out that they think, I think there should be a follow up, even if it's just something like this, half dozen people that right, OK is there anything we can do for any of you?

0:38:44.470 --> 0:38:45.970
DB
Is there anything you need?

0:38:46.170 --> 0:38:49.720
DB
I mean I, you know, I was struggling to get up the stairs and then someone said I think it's.

0:38:49.790 --> 0:38:50.660
DB
I can't remember who it was.

0:38:50.670 --> 0:38:53.530
DB
I suddenly got a phone call from this person.

0:38:53.540 --> 0:38:54.460
DB
I can't remember who it was.

0:39:0.550 --> 0:39:0.790
PW (LC clinic)
Yeah.

0:38:54.470 --> 0:39:2.460
DB
Again, who said we think we need to perhaps give you some aids four in the House.

0:39:3.510 --> 0:39:3.790
PW (LC clinic)
OK.

0:39:4.260 --> 0:39:12.350
DB
Ohh, got that because obviously you can't stand you're on crutches the whole time or walking stick and you're literally have chairs everywhere to sit down on quick.

0:39:13.500 --> 0:39:14.760
DB
So he gave me a store.

0:39:14.860 --> 0:39:15.290
DB
Gave me that.

0:39:15.300 --> 0:39:19.370
DB
Don't be getting the bath because he couldn't get in there until my daughter came round.

0:39:19.380 --> 0:39:23.830
DB
She used to come out dated and so I could get in the shower and make sure I didn't slip.

0:39:24.170 --> 0:39:24.460
PW (LC clinic)
Wow.

0:39:24.180 --> 0:39:25.770
DB
You know, it's like that.

0:39:25.960 --> 0:39:31.330
DB
I I've got a an on street upstairs with a shower, but I couldn't get up those stairs as well.

0:39:37.580 --> 0:39:37.900
PW (LC clinic)
Right.

0:39:31.720 --> 0:39:40.310
DB
I could just about make the first lot of stairs, but I couldn't get the second step to get up to do the shower, so I had to try and get my leg in the bath.

0:39:41.230 --> 0:39:41.570
PW (LC clinic)
Yeah.

0:39:40.320 --> 0:39:46.950
DB
Kind of thing, which is just that I couldn't have a bath because I couldn't get out because I had no strength.

0:39:46.700 --> 0:39:47.30
PW (LC clinic)
Right.

0:39:47.40 --> 0:39:48.0
PW (LC clinic)
So yeah.

0:39:55.670 --> 0:39:56.520
PW (LC clinic)
And any assistance.

0:40:1.640 --> 0:40:2.270
PW (LC clinic)
Yeah.

0:40:2.340 --> 0:40:2.940
PW (LC clinic)
Yeah. OK.

0:39:47.690 --> 0:40:12.290
DB
So it's it's all those things that are happening and that was going on for a while before I've got any uh OHS to say ohh you know we think you need some assistance coming out so you know that was that was obviously a great help to me and as soon as they were done obviously I gave him back and said look you know don't leave this anymore.

0:40:14.30 --> 0:40:14.250
PW (LC clinic)
Umm.

0:40:17.470 --> 0:40:18.90
PW (LC clinic)
Right. OK.

0:40:12.340 --> 0:40:23.520
DB
8 to cope without these, but you know that should have been right at the beginning when OK, A came out in crutches, which Claire came out in a wheelchair because I couldn't walk that far.

0:40:24.150 --> 0:40:27.390
DB
So you know, seeing that there should have been immediate, right.

0:40:31.460 --> 0:40:31.770
PW (LC clinic)
Right.

0:40:27.400 --> 0:40:32.920
DB
OK, let's get some stuff into her house to help her, you know? Here's the.

0:40:31.780 --> 0:40:35.90
PW (LC clinic)
So there there was a delay that the delay really was an issue.

0:40:33.970 --> 0:40:35.870
DB
Yeah, yeah.

0:40:35.180 --> 0:40:38.170
PW (LC clinic)
It in both respects the initial delay.

0:40:48.110 --> 0:40:49.160
DB
Yes, that's it.

0:40:49.170 --> 0:40:49.250
DB
Yeah.

0:40:38.180 --> 0:40:49.290
PW (LC clinic)
I suppose you know between coming out of hospital and then you first point the clinic and then obviously delay with things like this, which sounds like would it be an occupational health that you spoke to?

0:40:49.300 --> 0:40:49.870
PW (LC clinic)
Yeah.

0:40:49.910 --> 0:40:50.300
PW (LC clinic)
Yeah.

0:40:50.340 --> 0:40:51.620
PW (LC clinic)
OK. OK.

0:40:49.940 --> 0:40:53.780
DB
Yeah, I mean, I was very quick and getting them in the next day.

0:40:53.790 --> 0:40:54.540
DB
I mean, I had it.

0:40:54.690 --> 0:40:55.770
DB
What I needed here?

0:40:56.500 --> 0:40:58.790
DB
Umm, you know, and they they were really good.

0:40:59.100 --> 0:40:59.810
DB
Absolutely.

0:40:59.820 --> 0:41:1.550
DB
I mean, the guy was was spot on.

0:41:2.80 --> 0:41:2.440
PW (LC clinic)
Right.

0:41:1.900 --> 0:41:6.420
DB
Only young could have been managed 20th, but he was really on the ball.

0:41:7.380 --> 0:41:7.780
PW (LC clinic)
Right.

0:41:7.860 --> 0:41:13.860
PW (LC clinic)
OK, so when it arrived it was great, but it was just the weight in between, yeah.

0:41:14.390 --> 0:41:19.460
DB
Yeah, I mean, I didn't know I could get these things because often have a pin in that situation.

0:41:19.790 --> 0:41:20.50
PW (LC clinic)
Yeah.

0:41:19.470 --> 0:41:21.220
DB
So you don't know what you ask for?

0:41:22.40 --> 0:41:29.10
DB
Umm, you know or or what's gonna come come out like you just was lack of communication really.

0:41:29.700 --> 0:41:38.350
PW (LC clinic)
Umm any other any other points DByou know any other things that you think could have improved it could have been improved in relation to the service?

0:41:39.690 --> 0:41:41.700
DB
I think they could have had a counselor.

0:41:41.710 --> 0:41:43.560
DB
I did see a counselor.

0:41:43.570 --> 0:41:43.820
DB
One.

0:41:43.830 --> 0:41:44.410
DB
What was that on?

0:41:44.420 --> 0:41:47.650
DB
Cause I had chemo before I saw the Council on chemo.

0:41:48.600 --> 0:41:48.970
DB
Uh.

0:41:50.560 --> 0:41:56.560
DB
Or can't remember if I saw someone I spoke to someone on long COVID I can't remember to be honest with you.

0:41:57.120 --> 0:41:57.440
PW (LC clinic)
Hmm.

0:41:57.640 --> 0:41:59.230
DB
But definitely choose me.

0:42:2.770 --> 0:42:3.280
PW (LC clinic)
Are you OK?

0:42:2.760 --> 0:42:4.210
DB
Definitely. Yeah.

0:42:4.220 --> 0:42:4.300
DB
Yeah.

0:42:5.140 --> 0:42:5.300
DB
No.

0:42:3.290 --> 0:42:6.0
PW (LC clinic)
Dawn, did you wanna grab a glass of water or anything or?

0:42:6.690 --> 0:42:7.120
DB
Or coffee.

0:42:7.130 --> 0:42:8.670
DB
It's fine, it's just it gradually.

0:42:9.960 --> 0:42:10.140
PW (LC clinic)
Yeah.

0:42:8.680 --> 0:42:10.850
DB
It just gets me now.

0:42:10.860 --> 0:42:14.650
DB
It's the I've got.

0:42:14.660 --> 0:42:17.910
DB
What we were saying now, sorry, it's a bit like a get.

0:42:16.620 --> 0:42:18.290
PW (LC clinic)
I'm yeah.

0:42:18.420 --> 0:42:24.270
PW (LC clinic)
It was just kind of like any other things that you think could have been, you know, could could be improved about about the service.

0:42:24.680 --> 0:42:24.820
DB
Yeah.

0:42:24.420 --> 0:42:25.590
PW (LC clinic)
So we've mentioned, yeah.

0:42:26.180 --> 0:42:34.230
DB
Yeah, I think counseling would have been a good thing at the beginning to say.

0:42:34.240 --> 0:42:34.500
DB
Right.

0:42:34.510 --> 0:42:34.830
DB
OK.

0:42:34.840 --> 0:42:35.750
DB
How do you feel?

0:42:36.50 --> 0:42:37.690
DB
This is what can happen now.

0:42:42.270 --> 0:42:42.490
PW (LC clinic)
Yeah.

0:42:45.120 --> 0:42:45.430
PW (LC clinic)
Yeah.

0:43:9.750 --> 0:43:10.880
PW (LC clinic)
Hmm hmm.

0:43:25.380 --> 0:43:25.580
PW (LC clinic)
Yeah.

0:42:38.160 --> 0:43:27.510
DB
So you it's kind of letting you know in advance. What could happen. And if it doesn't bang. It's a bonus. You know, but just I don't know they didn't know an awful lot, but then you more than I knew as I said, You know, so there could have been formed me and said look you know this is what you you do this is what you we need to get you, your your your aids Etcetera. And if you do these exercises on something I mean, I did as much as I possibly could to walk around and and and get up and down the stairs. I was just determined to do it, but I can see the other side of people that aren't as determined you know, I'd I'd young grandkids. I wanted to still be able to see them and you know when I came out when I first woke up at the hospital. I I just thought I'd never walk again. So I was determined to get that done and when 7 days.

0:43:31.340 --> 0:43:32.110
PW (LC clinic)
Fantastic though.

0:43:27.520 --> 0:43:35.430
DB
I'm actually get out of the wheelchair with crutches so but that that was ******** copy on this hard.

0:43:34.740 --> 0:43:35.860
PW (LC clinic)
I should imagine, yeah.

0:43:35.810 --> 0:43:39.710
DB
But I do brilliant physios, both called Alex, 2 girls.

0:43:40.230 --> 0:43:41.270
DB
Amazing.

0:43:41.800 --> 0:43:42.650
DB
Amazing.

0:43:43.160 --> 0:43:47.520
DB
They really, you know, I wanted to do to do it for them because they were so nice.

0:43:48.620 --> 0:43:48.950
DB
I want to.

0:43:48.680 --> 0:43:49.210
PW (LC clinic)
And where were they?

0:43:49.220 --> 0:43:50.70
PW (LC clinic)
Where were they from, dawn?

0:43:50.80 --> 0:43:50.320
PW (LC clinic)
Where?

0:43:50.330 --> 0:43:50.490
PW (LC clinic)
Where?

0:43:50.500 --> 0:43:51.960
PW (LC clinic)
Where did you get your the physios from?

0:43:51.250 --> 0:43:53.40
DB
Mr Lister, funnier.

0:43:53.510 --> 0:43:53.950
PW (LC clinic)
Right.

0:43:53.960 --> 0:43:56.30
PW (LC clinic)
So you so and and was that.

0:43:56.110 --> 0:44:0.700
PW (LC clinic)
That's obviously something that was separate to the to the actual sort of like official long COVID clinic.

0:44:0.710 --> 0:44:0.880
PW (LC clinic)
Was it?

0:44:0.890 --> 0:44:1.430
PW (LC clinic)
That was something.

0:44:1.300 --> 0:44:1.870
DB
Yeah, I was.

0:44:1.960 --> 0:44:4.340
DB
I was staying in hospital at the time when I came out.

0:44:4.70 --> 0:44:4.870
PW (LC clinic)
Right, right.

0:44:5.340 --> 0:44:6.790
DB
No, no physio at all.

0:44:8.550 --> 0:44:8.860
PW (LC clinic)
Right.

0:44:6.800 --> 0:44:9.240
DB
When I came out, no.

0:44:8.870 --> 0:44:10.730
PW (LC clinic)
So you know, great while you're in there.

0:44:10.740 --> 0:44:12.810
PW (LC clinic)
But then, as you say is this adds to?

0:44:14.450 --> 0:44:14.690
DB
Yeah.

0:44:12.820 --> 0:44:15.40
PW (LC clinic)
Why you felt sort of dumped, you know? Sort of.

0:44:15.150 --> 0:44:15.810
PW (LC clinic)
Yeah. OK.

0:44:15.760 --> 0:44:16.710
DB
Yeah, there's people said.

0:44:16.720 --> 0:44:18.320
DB
Didn't you get your physio went or no?

0:44:19.540 --> 0:44:19.790
PW (LC clinic)
Umm.

0:44:19.460 --> 0:44:21.170
DB
Yeah, yeah, that was that.

0:44:21.600 --> 0:44:22.310
DB
You know you're up.

0:44:22.320 --> 0:44:24.480
DB
You're out if you go kind of thing.

0:44:24.50 --> 0:44:25.500
PW (LC clinic)
Yeah. OK.

0:44:34.320 --> 0:44:34.550
PW (LC clinic)
Umm.

0:44:25.100 --> 0:44:37.470
DB
I'm but Physio again after as I saying I was going up the stairs on all fours and I was as weak as a kitten, so trying to come down again. Again.

0:44:37.480 --> 0:44:37.680
DB
You.

0:44:37.690 --> 0:44:41.780
DB
You're coming down on all fours backwards now.

0:44:42.160 --> 0:44:42.410
PW (LC clinic)
Where?

0:44:41.790 --> 0:44:46.60
DB
I live alone, which is, you know, also a bit scary at times and.

0:44:45.930 --> 0:44:46.640
PW (LC clinic)
Yeah.

0:44:46.690 --> 0:44:47.950
PW (LC clinic)
Yeah, it makes you feel vulnerable.

0:44:47.960 --> 0:44:48.360
PW (LC clinic)
I should imagine.

0:44:48.960 --> 0:44:49.990
DB
Very vulnerable.

0:44:50.0 --> 0:44:50.510
DB
Yeah.

0:44:50.880 --> 0:44:51.110
PW (LC clinic)
Umm.

0:44:50.960 --> 0:44:52.540
DB
You know, I think of yourself.

0:44:52.550 --> 0:44:52.700
DB
What?

0:44:52.710 --> 0:44:53.10
DB
OK.

0:44:53.20 --> 0:44:54.950
DB
Can I get to to get that cup of tea?

0:45:2.230 --> 0:45:2.670
PW (LC clinic)
Right.

0:44:55.280 --> 0:45:3.760
DB
And with the day you get it, you know you only get half a cup of tea because you don't wanna slap it everywhere cause you're shaking and you do get really bad shakes.

0:45:4.800 --> 0:45:5.100
DB
Umm.

0:45:5.520 --> 0:45:10.250
DB
And because your muscles and your nerves have all gone, so they've all gotta build up against you.

0:45:10.270 --> 0:45:10.600
PW (LC clinic)
Yeah.

0:45:10.260 --> 0:45:13.530
DB
Really makes you can't hold a cup of tea without shaking.

0:45:13.680 --> 0:45:15.190
DB
It's like a Parkinson's, you know?

0:45:16.480 --> 0:45:16.730
PW (LC clinic)
Yeah.

0:45:15.200 --> 0:45:23.300
DB
You know, but you know, when you get to that stage, you've got 3/4 of a cup and you haven't stopped it all over the floor.

0:45:23.310 --> 0:45:25.240
DB
It's it's like it's such a relief.

0:45:25.970 --> 0:45:26.230
PW (LC clinic)
Umm.

0:45:27.170 --> 0:45:28.450
DB
That thing, you know that you're not.

0:45:29.220 --> 0:45:30.870
DB
You not expecting you.

0:45:34.170 --> 0:45:35.410
PW (LC clinic)
Yeah, it's so.

0:45:30.880 --> 0:45:37.580
DB
You just gotta try and cope with it yourself that then it is really a struggle.

0:45:45.220 --> 0:45:45.460
DB
Umm.

0:45:38.420 --> 0:45:46.950
PW (LC clinic)
So with the, you know what you're saying is like, you know, there could have been that the just the psychological support through this kind of period would have been really beneficial.

0:45:47.240 --> 0:45:47.970
PW (LC clinic)
Dawn, you think?

0:45:47.980 --> 0:45:48.80
PW (LC clinic)
Yeah.

0:45:48.110 --> 0:45:49.640
DB
Yeah, yeah.

0:45:49.40 --> 0:45:49.720
PW (LC clinic)
Yeah. OK.

0:45:49.790 --> 0:45:51.920
DB
It When you're cause.

0:45:52.0 --> 0:45:54.320
DB
Well, obviously I live in my own and I don't do tears.

0:45:54.490 --> 0:45:55.620
DB
None of us in the family.

0:45:55.630 --> 0:45:56.700
DB
Me, my sister never did it.

0:45:57.480 --> 0:45:57.730
PW (LC clinic)
Hmm.

0:46:2.470 --> 0:46:2.680
PW (LC clinic)
Umm.

0:45:57.960 --> 0:46:3.450
DB
But you know, I'd sit here and I've just been tears it.

0:46:3.910 --> 0:46:5.380
DB
How long is this going on for?

0:46:6.420 --> 0:46:6.640
PW (LC clinic)
Yeah.

0:46:6.430 --> 0:46:11.780
DB
And again, if you had that counselling, you could go to and say to them, I think I did get one.

0:46:11.790 --> 0:46:14.430
DB
I'm not sure I've got something in the back of my mind's going.

0:46:16.190 --> 0:46:16.710
PW (LC clinic)
Right.

0:46:14.440 --> 0:46:18.960
DB
You saw someone but nothing else after that.

0:46:20.150 --> 0:46:20.410
PW (LC clinic)
OK.

0:46:20.140 --> 0:46:23.210
DB
Umm, I can't quite remember to be honest with you.

0:46:23.220 --> 0:46:29.890
DB
It's as I say, you know, it's it's part when you gonna come 1/2 your brain cuts off and doesn't come back.

0:46:29.530 --> 0:46:30.250
PW (LC clinic)
Yeah.

0:46:56.310 --> 0:46:56.570
DB
Is it?

0:47:12.410 --> 0:47:12.590
DB
Yeah.

0:46:30.290 --> 0:47:17.150
PW (LC clinic)
So, so again, you know just and I know that you know this isn't something you said explicitly down, but the fact that there are those kind of issues you know coming out as you say you've been through that whole traumatic experience, you come out and then well I'm picking up is you know it's it's gonna be difficult for someone that's been through what you've been through to then try and potentially remember you know yeah could there be more support could there be more support around sort of like that you know this is some way of of letting you know what we're doing and when the next appointment is and all the rest of it you know did it feel a bit like oh there's no central place from which you can get information about what's happening next and what have you yeah did it feel a bit sort of so again DBsorry.

0:47:20.150 --> 0:47:20.400
PW (LC clinic)
Hmm.

0:47:14.740 --> 0:47:20.680
DB
That .1 I feel there should be one contact point, not the doctor.

0:47:20.690 --> 0:47:20.940
DB
Don't.

0:47:29.120 --> 0:47:29.330
PW (LC clinic)
Umm.

0:47:20.950 --> 0:47:31.800
DB

This is yeah, not the this and not back, but one contact point where you can say right you know, I'm DBhave, I got really thing coming up because I if I put it round down somewhere or written.

0:47:31.810 --> 0:47:33.160
DB
It somewhere off? I've got a letter.

0:47:33.410 --> 0:47:37.980
DB
It's and that it's really, really so hard to to deal with.

0:47:40.490 --> 0:47:40.830
PW (LC clinic)
Right.

0:47:38.250 --> 0:47:45.570
DB
I struggle with that daily and sometimes I get really crossing myself and cross with other people who go well.

0:47:45.580 --> 0:47:46.300
DB
I just told you that.

0:47:46.940 --> 0:47:48.810
PW (LC clinic)
And it's a typical long COVID symptom, isn't it?

0:47:49.490 --> 0:47:50.400
DB
Absolutely.

0:47:48.820 --> 0:47:51.550
PW (LC clinic)
So umm yeah, yeah.

0:47:50.410 --> 0:47:52.400
DB
Yeah, in my bed.

0:47:52.410 --> 0:47:53.800
DB
Me, but I've already told you that, mom.

0:47:54.250 --> 0:47:59.560
DB
Why I care and then I'm like, well, best, you know, she got it alright.

0:47:59.630 --> 0:48:1.120
DB
But my son in law is different.

0:48:1.290 --> 0:48:2.420
DB
He's got the other son.

0:48:2.430 --> 0:48:7.50
DB
He understands a bit more because he said, you know, you're worth more to me at life than you are dead.

0:48:7.60 --> 0:48:7.840
DB
So I've gotta keep you alive.

0:48:10.840 --> 0:48:16.760
DB
It and you know, when I was ill not so long ago with this cough and that my daughter sent me mum.

0:48:16.770 --> 0:48:20.900
DB
You need to come and stay here because I'm not having you all for another three weeks and then going to hospital.

0:48:21.110 --> 0:48:22.690
DB
I can't help you if you're going to hospital.

0:48:23.330 --> 0:48:24.310
PW (LC clinic)
Umm yes.

0:48:23.490 --> 0:48:24.570
DB
My friend.

0:48:24.990 --> 0:48:27.970
DB
So I went and stayed with her for nearly three weeks.

0:48:28.620 --> 0:48:31.980
PW (LC clinic)
Wow, OK, good, good, good family support then dawn. Yeah.

0:48:29.700 --> 0:48:33.140
DB
The alcohol absolute well, yeah.

0:48:33.150 --> 0:48:42.520
DB
I mean, you know, we are a close family, but you know she's she's very much on the ball with herbal remedies and what you need.

0:48:42.530 --> 0:48:45.740
DB
And she knows a lot of people that are also into that.

0:48:45.750 --> 0:48:47.190
DB
So they all give their input.

0:48:48.220 --> 0:48:48.580
PW (LC clinic)
Brilliant.

0:48:48.100 --> 0:48:49.490
DB
Umm, how about?

0:48:49.500 --> 0:48:53.110
DB
Except I know people poo poo it, but hate it works for me, you know?

0:48:58.140 --> 0:48:58.380
PW (LC clinic)
Umm.

0:48:53.120 --> 0:49:1.980
DB
And rather than just keep taking drugs all the time that aren't working, I would drive, try, Plan BI haven't got a plan C yet.

0:49:1.990 --> 0:49:7.180
DB
That could be six that face our plan to say so, you know.

0:49:8.90 --> 0:49:10.490
DB
But my my son in law would say to me right, OK.

0:49:11.50 --> 0:49:12.360
DB
And you'll tell me something.

0:49:12.490 --> 0:49:13.640
DB
And he look at me and you go.

0:49:13.650 --> 0:49:14.800
DB
I didn't quite get there, did it?

0:49:14.810 --> 0:49:15.480
DB
Let's try it again.

0:49:16.70 --> 0:49:20.510
DB
And he can see from my face that just it's just come past me.

0:49:21.180 --> 0:49:21.380
PW (LC clinic)
Yeah.

0:49:21.850 --> 0:49:22.120
DB
I'm.

0:49:22.130 --> 0:49:22.700
DB
I'm just.

0:49:30.480 --> 0:49:31.340
PW (LC clinic)
Yeah, yeah.

0:49:22.750 --> 0:49:32.410
DB
I don't take it in and again, that's not me and I could stand a week when that happens, it is so the tating.

0:49:32.420 --> 0:49:35.310
DB
So frustrating, you know?

0:49:35.360 --> 0:49:38.910
DB
And but he's so patient with it, which is a good thing, my daughter.

0:49:38.710 --> 0:49:39.190
PW (LC clinic)
Absolutely.

0:49:38.920 --> 0:49:41.750
DB
Not that you know, she.

0:49:41.890 --> 0:49:44.690
DB
She's got two kids and me to contend with it.

0:49:45.140 --> 0:49:46.30
DB
Yeah, yeah.

0:49:46.100 --> 0:49:46.550
PW (LC clinic)
So.

0:49:46.560 --> 0:49:48.100
PW (LC clinic)
So yeah, dawn, definitely something.

0:49:48.640 --> 0:49:51.750
PW (LC clinic)
Seems like you know there could be.

0:49:52.40 --> 0:49:53.930
PW (LC clinic)
I'm just saying this off top of my head, but just.

0:49:55.360 --> 0:50:0.170
PW (LC clinic)
Imagine like an app or something which has got all the details of who you've spoken to so far.

0:50:0.360 --> 0:50:2.130
PW (LC clinic)
Contact details what's happening there?

0:50:2.140 --> 0:50:8.190
PW (LC clinic)
Some kind of central place where you've got access to essentially what's going on and what have you.

0:50:6.560 --> 0:50:9.320
DB
Yeah, put your little code in.

0:50:12.710 --> 0:50:13.20
PW (LC clinic)
Yeah.

0:50:13.380 --> 0:50:16.420
DB
Or next appointments and you know, just a little bit of insight.

0:50:13.80 --> 0:50:17.810
PW (LC clinic)
Yeah, absolutely.

0:50:17.900 --> 0:50:18.270
PW (LC clinic)
Yeah.

0:50:18.320 --> 0:50:18.490
PW (LC clinic)
No.

0:50:18.500 --> 0:50:18.870
PW (LC clinic)
Great.

0:50:18.380 --> 0:50:18.880
DB
You're not.

0:50:18.910 --> 0:50:20.40
PW (LC clinic)
Great point, dawn.

0:50:19.160 --> 0:50:25.590
DB
You could do as well that I could sit and think ohh you must just write that deck and I'll type it into there.

0:50:27.740 --> 0:50:28.20
PW (LC clinic)
Hmm.

0:50:25.700 --> 0:50:30.190
DB
Which also reminds me I can go anytime and look at what?

0:50:30.990 --> 0:50:33.770
DB
Well, I thought of that like after that session.

0:50:34.710 --> 0:50:36.20
DB
I could have gone into that and gone.

0:50:36.30 --> 0:50:37.150
DB
My OK didn't.

0:50:38.190 --> 0:50:40.280
DB
You know, didn't think that was much help, blah blah blah.

0:50:40.370 --> 0:50:45.130
DB
So when someone does ask you or wants to look at that, you can give permission to look at it.

0:50:45.700 --> 0:50:45.900
PW (LC clinic)
Yeah.

0:50:45.880 --> 0:50:48.70
DB
Uh, now I would wouldn't bother me at all.

0:50:48.740 --> 0:50:49.230
DB
Umm.

0:50:49.600 --> 0:51:2.750
DB
And they can see everything that you've gone and done and any comments that I feel on there and you know they can address that or think well, well, she's having another moment that would do her, you know, so it's.

0:51:4.240 --> 0:51:4.570
PW (LC clinic)
No.

0:51:4.580 --> 0:51:5.270
PW (LC clinic)
Great, great.

0:51:5.400 --> 0:51:6.270
PW (LC clinic)
Great point, dawn.

0:51:6.280 --> 0:51:7.150
PW (LC clinic)
Thank you so much.

0:51:12.470 --> 0:51:12.580
DB
Yeah.

0:51:7.620 --> 0:51:13.150
PW (LC clinic)
So I just now we're coming up on the hour and I actually only have one question left, which isn't even really a question.

0:51:13.240 --> 0:51:24.150
PW (LC clinic)
So it's just obviously touched a lot of things and they might well have been something you've mentioned that you sort of thought, you know, thinking back I didn't get a chance to sort of like go into that in any you know, in, in, in the depth of the light.

0:51:24.160 --> 0:51:24.400
PW (LC clinic)
So is.

0:51:24.410 --> 0:51:32.990
PW (LC clinic)
Is there anything that we that we've gone over that you sort of think you know I didn't get a chance to say X or Y you know anything?

0:51:32.320 --> 0:51:34.400
DB
You you may not say something.

0:51:36.780 --> 0:51:36.990
DB
Really.

0:51:36.410 --> 0:51:37.340
PW (LC clinic)
I wouldn't say it's.

0:51:39.740 --> 0:51:39.920
DB
No.

0:51:37.570 --> 0:51:42.330
PW (LC clinic)
I wouldn't say it's a a major characteristic DBof yours, just from just from our short time together.

0:51:45.220 --> 0:51:45.690
PW (LC clinic)
Bless you.

0:51:45.700 --> 0:51:46.90
PW (LC clinic)
OK, well.

0:51:45.510 --> 0:51:48.730
DB
I do these long COVID things for other people.

0:51:56.210 --> 0:51:56.330
PW (LC clinic)
Yeah.

0:51:51.530 --> 0:51:58.510
DB
Yeah, I OK, guys, you know what's happened to you and you know, and just get them all talking.

0:51:58.660 --> 0:52:5.770
DB
And The thing is, if you talked about it and you talk to each other, you'll find that other people will start to talk.

0:52:5.920 --> 0:52:10.380
DB
And they'll also find they'll talk to other people outside that team as well.

0:52:10.900 --> 0:52:11.180
PW (LC clinic)
Yeah.

0:52:11.190 --> 0:52:11.820
PW (LC clinic)
So you can create.

0:52:17.890 --> 0:52:18.170
PW (LC clinic)
Yeah.

0:52:11.210 --> 0:52:22.240
DB
People say you you'll create in that that thing of them wanting to say something about it and you know that mentally that is gotta be such a help.

0:52:29.540 --> 0:52:29.800
DB
But.

0:52:34.160 --> 0:52:34.340
DB
Yeah.

0:52:22.990 --> 0:52:34.880
PW (LC clinic)
Yeah, stepping out of that sort of a sort of passive, I'm on my own, sort of like a, you know, without any support, just stepping over that line into and creating a a support network, which would be great from something.

0:52:34.890 --> 0:52:37.870
PW (LC clinic)
It's it's great if that could work from someone that wouldn't it.

0:52:38.250 --> 0:52:39.240
PW (LC clinic)
That would be great when it done.

0:52:38.160 --> 0:52:42.110
DB
Uh, but, you know, people could you could say, right.

0:52:42.120 --> 0:52:44.930
DB
OK, once a month, we're just gonna have 1/2 hour meeting.

0:52:44.940 --> 0:52:45.920
DB
Join me on that.

0:52:46.80 --> 0:52:47.30
DB
Everyone doesn't join.

0:52:47.40 --> 0:52:47.470
DB
That's fine.

0:52:48.650 --> 0:52:48.870
PW (LC clinic)
Yeah.

0:52:47.480 --> 0:52:49.750
DB
Who are OK the next months?

0:52:49.820 --> 0:52:50.600
DB
Ohh, hang on a minute.

0:52:50.610 --> 0:52:52.50
DB
We've got more here, they've said.

0:52:52.60 --> 0:52:57.570
DB
I think about this, you know, and you could just say to them, even if it wasn't a month, two months, three months or whatever.

0:52:57.620 --> 0:52:59.320
DB
But I think, Pete, three months is too long.

0:52:59.940 --> 0:53:0.320
PW (LC clinic)
Hmm.

0:53:8.340 --> 0:53:8.640
PW (LC clinic)
Yeah.

0:53:0.630 --> 0:53:11.0
DB
I think if there was a monthly thing that people could, and they're gonna speak more and more and more because things happen in that month, you know, probably you got something else going wrong.

0:53:11.10 --> 0:53:11.380
DB
Do you think?

0:53:11.410 --> 0:53:11.760
DB
Oh ****.

0:53:13.60 --> 0:53:13.820
PW (LC clinic)
Yeah, yeah.

0:53:11.770 --> 0:53:14.40
DB
Now what you know and I said, I don't.

0:53:14.190 --> 0:53:15.260
DB
I'm sick of being.

0:53:15.270 --> 0:53:15.800
DB
I'm well.

0:53:15.810 --> 0:53:18.520
DB
I'm sick of just falling asleep on the sofa.

0:53:21.640 --> 0:53:21.880
PW (LC clinic)
Umm.

0:53:18.530 --> 0:53:25.760
DB
I'm sick of going to bed at 8:00 o'clock, so I'm so tired, you know, and then waking up at 5:00 in the morning.

0:53:27.970 --> 0:53:28.930
PW (LC clinic)
Yeah, yeah.

0:53:25.770 --> 0:53:30.640
DB
I'm like Ohhh God it's too early, you know, but I can't help because I got to go to bed.

0:53:31.340 --> 0:53:34.600
DB
But then also to tell people to listen to their bodies.

0:53:35.110 --> 0:53:36.150
DB
I never did that.

0:53:36.600 --> 0:53:36.860
PW (LC clinic)
Yeah.

0:53:36.230 --> 0:53:42.210
DB
I never you my body until I got the cancer and my friend said to me, you need to listen to your body.

0:53:47.490 --> 0:53:48.120
PW (LC clinic)
Fantastic dawn.

0:53:42.600 --> 0:53:48.120
DB
And she was absolutely right that anyone has ever said to me, and I took it on board so.

0:53:48.130 --> 0:53:48.360
PW (LC clinic)
So.

0:53:48.370 --> 0:53:48.600
PW (LC clinic)
So.

0:53:48.670 --> 0:53:59.840
PW (LC clinic)
So yeah, some way of creating that sort of like group, whether it be virtually whether it be face to face a essentially doesn't matter, I guess from what we said earlier, but just somewhere where these kind of things can develop.

0:53:59.850 --> 0:54:5.280
PW (LC clinic)
And then people can build confidence to be able to speak and bring their own sort of experience to the group.

0:54:5.630 --> 0:54:5.800
DB
Yeah.

0:54:5.350 --> 0:54:11.680
PW (LC clinic)
And then you know, and then they might well be things that can be discussed and advice between people and, you know, this is good.

0:54:11.690 --> 0:54:18.910
PW (LC clinic)
Try this out and so just to create sort of a supportive network that can facilitate, you know recovery I guess would be.

0:54:19.80 --> 0:54:19.750
DB
We're at six.

0:54:19.760 --> 0:54:25.580
DB
Yeah, I mean, it is all about getting the recovery and and and getting yourself your own well being.

0:54:26.310 --> 0:54:26.590
PW (LC clinic)
Hmm.

0:54:26.910 --> 0:54:29.780
DB
You know, it's listening to your body and helping yourself.

0:54:29.790 --> 0:54:31.240
DB
Don't rely on the medication.

0:54:31.250 --> 0:54:33.350
DB
Don't rely on the doctors and everything else.

0:54:33.410 --> 0:54:35.250
PW (LC clinic)
Yeah. Umm.

0:54:33.430 --> 0:54:42.10
DB
Help health the that gives them self satisfaction that they're feeling better by doing something or talking to someone about it.

0:54:42.270 --> 0:54:47.890
DB
It's a weight off their minds and it's it's just gonna be mentally.

0:54:47.900 --> 0:54:55.770
DB
It's gonna be much healthier than than not doing anything, not saying anything and not knowing where to go ask.

0:54:56.140 --> 0:54:56.510
PW (LC clinic)
Yeah.

0:54:56.520 --> 0:55:0.530
PW (LC clinic)
So gaining a bit of control over the situation, really dawn, which you say, yeah.

0:55:0.620 --> 0:55:1.380
PW (LC clinic)
Yeah. OK.

0:54:58.780 --> 0:55:1.500
DB
Yeah, yeah, yeah. What?

0:55:3.390 --> 0:55:3.610
PW (LC clinic)
Yeah.

0:55:4.790 --> 0:55:5.380
PW (LC clinic)
Fantastic.

0:55:5.390 --> 0:55:10.970
PW (LC clinic)
Dawn, I'm just gonna and hit stop on the recorder.
