# Supplementary material for: A mixed-methods analysis of the implementation of a new community long-COVID service during the 2020 pandemic: Learning from practice
Source: PLoS One. 2026 Jun 26;21(6):e0313367. doi: 10.1371/journal.pone.0313367 (PMC13308792; doi:10.1371/journal.pone.0313367)
Supplement: Attachment — Submitted filename: Response to reviewers 13Aug25.pdf [file pone.0313367.s011.pdf]

Letter to Editor and Reviewers

Dear Editor Dr Mohammed Abutaleb and Reviewers,

Thank you for your request to revise our paper to which we responded via a letter dated 03 April 2025. We want to reassure the reviewers every comment was taken into consideration and responded to in our previous rebuttal letter dated 31 Dec 2024 (below) and in the second version of the paper.

We wonder if reviewer #1 considered the revised version of our article and figures/table, since the listing of original and revised documents may have been misleading. All the changes they suggested during the first review were made and clearly explained in the revised version with changes highlighted.

In particular we refer to reviewer #1 comments, hereby between ** **, our reply is found after >>>:

**RESULTS
- Table 1 is not visually appealing. It contains grid lines, which are not recommended for a table.
- I suggest removing the age variable from the table and describing it only in the paragraph above. Thus, I recommend that the table only includes variables with N and %.
- All tables need to be better formatted to be more visually pleasant to the reader. I emphasize that tables should not have closed grid lines. Perhaps what the authors are calling a table should be called a figure/chart. It is better to check with the journal's editing guidelines.
- The table titles also need to be revised, as they do not include the location and date/period of data collection.**

>>> **Response (from December rebuttal letter)**: thank you for this suggestion, table 1 and 3 were changed to figure 1 (line 285 of Manuscript revison3) with infographics to provide a clearer description of the patients' population and figure 4 (line 554) showing barriers and facilitators to care and service implementation. We checked the editing guidelines of the journal, other manuscripts published by PLOSONE, and addressed this comment accordingly.

>>> **Additional comment:** Table 1 (line 390) is now referring to a different part of the results: TDF domains and subdomains across patients and HCPs.
We changed the format of the table removing grid lines, we thought that was done when the paper is edited by the journal for publishing, we will wait for your advice on this. We did add the location and date of data collection in the title.

**DISCUSSION
- In the first paragraph of the discussion, there is no need to repeat the results, as they are already described above.
- I understand that the first two items of the Discussion still fit into the Results section. The authors indeed discuss in the item "Comparison with Other Studies."**

>>> **Response (rebuttal letter):** thank you for these comments, we have reviewed the relevant sections of results and discussion to avoid duplication, and use the statement of findings (line 529: Statement of principal findings) to summarise the results at the end of the results section, we believe this is good practice and also suggested by publishing guidelines.

We understand that, when the previously submitted PDF was downloaded, the original submission appeared first, therefore creating confusion.
Please, let us know if, in your opinion, the above-mentioned changes, found in the revised version of the paper and new table/figures, are in accordance with the reviewer's comments.

Best regards
Stefanie Williams 13/08/2025

Original Revision Rebuttal Letter

Dr Stefanie L. Williams
University of Hertfordshire
College Lane, Hatfield, AL10 9AB

31st December 2024

Dear Editor,

We are pleased to resubmit our manuscript titled "A mixed-methods analysis of the implementation of a new community long-COVID service during the 2020 pandemic: learning from practice" for consideration by PLOS ONE.

We are thankful to reviewers for their helpful suggestions.

We hereby respond to the request for revision, and we provide a point-by-point rebuttal to editing and reviewers' requests.

Journal Requirements:

When submitting your revision, we need you to address these additional requirements.

1. Please ensure that your manuscript meets PLOS ONE's style requirements, including those for file naming.

Response: we changed the file names as per the journal requirements.

2. We note that the grant information you provided in the 'Funding Information' and 'Financial Disclosure' sections do not match.

When you resubmit, please ensure that you provide the correct grant numbers for the awards you received for your study in the 'Funding Information' section.

Response: as requested, we revised the information provided in the sections 'Funding Information' and 'Financial Disclosure'.

3. Thank you for stating the following in the Competing Interests section:

“HM received funding for a long-COVID GP Fellowship, to undertake clinical work and research, from NHS East and North Hertfordshire CCG. HM’s role within the study was funded by the LOCOMOTION study funding (National Institute for Health and Care Research, grant ref: COV-LT2-0016). HM received payment and travel expenses for long-COVID-related presentations and expert opinion. The remaining authors have declared that no competing interests exist.”

Please confirm that this does not alter your adherence to all PLOS ONE policies on sharing data and materials, by including the following statement: "This does not alter our adherence to PLOS ONE policies on sharing data and materials.” (as detailed online in our guide for authors http://journals.plos.org/plosone/s/competing-interests). If there are restrictions on sharing of data and/or materials, please state these. Please note that we cannot proceed with consideration of your article until this information has been declared.

Please include your updated Competing Interests statement in your cover letter; we will change the online submission form on your behalf.

Response: As requested, our original competing interests statement has been updated:

HM received funding for a long-COVID GP Fellowship, to undertake clinical work and research, from NHS East and North Hertfordshire CCG. HM’s role within the study was funded by the LOCOMOTION study funding (National Institute for Health and Care Research, grant ref: COV-LT2-0016). HM received payment and travel expenses for long-COVID-related presentations and expert opinion. This does not alter our adherence to PLOS ONE policies on sharing data and materials. The remaining authors have declared that no competing interests exist.

4. We note that you have indicated that there are restrictions to data sharing for this study. For studies involving human research participant data or other sensitive data, we encourage authors to share de-identified or anonymized data. However, when data cannot be publicly shared for ethical reasons, we allow authors to make their data sets available upon request. For information on unacceptable data access restrictions, please see http://journals.plos.org/plosone/s/data-availability#loc-unacceptable-data-access-restrictions.

Before we proceed with your manuscript, please address the following prompts:

a) If there are ethical or legal restrictions on sharing a de-identified data set, please explain them in detail (e.g., data contain potentially identifying or sensitive patient information, data are owned by a third-party organization, etc.) and who has imposed them (e.g., a Research Ethics Committee or Institutional Review Board, etc.). Please also provide contact information for a data access committee, ethics committee, or other institutional body to which data requests may be sent.

b) If there are no restrictions, please upload the minimal anonymized data set necessary to replicate your study findings to a stable, public repository and provide us with the relevant URLs, DOIs, or accession numbers. Please see http://www.bmj.com/content/340/bmj.c181.long for guidelines on how to de-

identify and prepare clinical data for publication. For a list of recommended repositories, please see https://journals.plos.org/plosone/s/recommended-repositories. You also have the option of uploading the data as Supporting Information files, but we would recommend depositing data directly to a data repository if possible.

Please update your Data Availability statement in the submission form accordingly.

Response: we uploaded the minimal anonymized data set necessary to replicate study findings as supplementary material: the quantitative dataset with anonymised data, and the transcript of interviews with patients. The clinic was small and led by one medical director and one general practitioner, therefore interviews with staff cannot be uploaded, otherwise readers may be able to recognise members of staff, with breach of confidentiality.

Reviewers' comments:

Reviewer's Responses to Questions

Comments to the Author

1. Is the manuscript technically sound, and do the data support the conclusions?

The manuscript must describe a technically sound piece of scientific research with data that supports the conclusions. Experiments must have been conducted rigorously, with appropriate controls, replication, and sample sizes. The conclusions must be drawn appropriately based on the data presented.

Reviewer #1: Partly

Reviewer #2: Yes

Reviewer #3: Yes

No action required.

2. Has the statistical analysis been performed appropriately and rigorously?

Reviewer #1: Yes

Reviewer #2: Yes

Reviewer #3: Yes

No action required.

3. Have the authors made all data underlying the findings in their manuscript fully available?

The PLOS Data policy requires authors to make all data underlying the findings described in their manuscript fully available without restriction, with rare exception (please refer to the Data Availability Statement in the manuscript PDF file). The data should be provided as part of the manuscript or its supporting information, or deposited to a public repository. For example, in addition to summary statistics, the data points behind means, medians and variance measures should be available. If there are restrictions on publicly sharing data—e.g. participant privacy or use of data from a third party—those must be specified.

Reviewer #1: Yes

Reviewer #2: Yes

Reviewer #3: Yes

No action required.

4. Is the manuscript presented in an intelligible fashion and written in standard English?

PLOS ONE does not copyedit accepted manuscripts, so the language in submitted articles must be clear, correct, and unambiguous. Any typographical or grammatical errors should be corrected at revision, so please note any specific errors here.

Reviewer #1: Yes

Reviewer #2: Yes

Reviewer #3: Yes

No action required.

5. Review Comments to the Author

Please use the space provided to explain your answers to the questions above. You may also include additional comments for the author, including concerns about dual publication, research ethics, or publication ethics. (Please upload your review as an attachment if it exceeds 20,000 characters)

Reviewer #1: A mixed-methods analysis of the implementation of a new long-term community service during the 2020 pandemic: learning from practice.
ABSTRACT Well-structured.
INTRODUCTION The authors divided the introduction into 4 subsections in which they explain the

problem to be addressed, the organization of health services that serve patients with CL, explain the approach used with the TDF and then the objectives of the study.

METHODS The study was carried out using mixed methods, with the quantitative part being the collection of secondary data from patients and the qualitative approach based on a semi-structured questionnaire applied to patients and health professionals.

- local specifications; Response: this is provided in lines 142-146
- specify the study population; Response: this is provided in lines 163-164
- specify the period in which the study was completed; Response: this is provided in line 167

- the authors specify the analysis of quantitative data (descriptive statistics, standard deviation and brightness tests between variables, as well as the qualitative data were decoded and detailed. Subsequently, data triangulation was performed. - ethical precepts were followed

No action required.

RESULTS - table 1 is not visually appealing. It contains note lines, which is not found in a table - I suggest removing the variable from the table and leaving it described only in the paragraph above; the table only contains variables with N and % - all tables need to be better formatted to be more visually appealing to the reader, tables cannot have closed note lines, perhaps what the authors reserve for a table is to be defined, called a figure/chart. It is best to check with the newspaper's editing standards; - the table titles also need to be reviewed, since they do not contain local data/data collection period;

Response: thank you for this suggestion, table 1 and 3 were changed to figure 1 with infographics to provide a clearer description of the patients' population and figure 4 showing barriers and facilitators to care and service implementation. We checked the editing guidelines of the journal, other manuscripts published by PLOSONE, and addressed this comment accordingly.

DISCUSSION - in the first paragraph of the discussion there is no need to repeat the results, this is already described above; - I understand that the first two items of the Discussion still fit into the Results. The authors, in fact, discuss the item Comparison with other studies;

Response: thank you for these comments, we have reviewed the relevant sections of results and discussion to avoid duplication and use the statement of findings to summarise the results.

- I suggest reviewing the need to separate the discussion into subtitles;

Response: we would like to explain that this choice was made to improve the readability of the section and to make it easier for readers to find information. We have deleted some subheadings.

- the authors point out the weaknesses of the study, as well as highlighting the need for future studies.

No action required.

Reviewer #2: The manuscript establishes a strong rationale for investigating long-COVID care pathways, given the condition's rising prevalence and complexity. However, the authors can ensure that the introduction further clearly defines key terms, such as "long-COVID" and "Theoretical Domains Framework" (TDF), for readers who may not be so familiar, by elaborating on the gaps in existing literature that this study aims to address, which would help highlight the study's uniqueness and importance.
Also, further methodological details could enhance replicability, such as deeply clarifying the data collection procedures, analysis methods (both qualitative and quantitative), and any steps taken to ensure reliability and validity.
Furthermore, the results provide valuable insights into patient needs, such as rehabilitation requirements and referrals to specialists. However, if feasible, the authors could provide more context or comparisons with similar studies to demonstrate how the findings align with or differ from the broader literature.

Response: we are grateful to the reviewer for these comments. We reviewed the introduction and relevant sections in accordance with these suggestions.

Overall, the presentation of quantitative results, table and tables is clear, further improving readability and making trends or key data points more immediately apparent to readers.
No action required.

Implications and Recommendations
The manuscript could be strengthened by offering more concrete recommendations based on the findings. For instance, if "funding constraints" were a barrier, the authors could suggest specific policy or funding changes to support long-COVID services. Thus, highlighting actionable strategies that other clinics or healthcare providers could adopt would further enhance the study's practical relevance.
Also, discussion on the limitations have helped to add balance to the interpretation, thus, helping the clarifications and generalizability of findings to other settings or populations.
Lastly, could the authors further mention (if available) any unanswered questions or emerging needs related to long-COVID care as observed during the study?

Response: thanks for these comments that greatly helped in improving the relevance of the paper to current practice challenges.

Reviewer #3: Dear Authors,
I have thoroughly reviewed the manuscript.
At this point, some minor revisions are still worth addressing before recommending your manuscript for publication.

Minor comments:
1—On page 10, line 231, please leave out the 'causality' exploration as it is outside the scope of the analysis, not supported by the proposed methodological framework, and not derived from your results.

Response: thank you for this suggestion that we have taken into consideration.

2- Please discuss the potential equity concerns regarding your results observing patients of the Long-Covid pathway to be predominantly female, white British people from non-deprived areas. You might reflect, for instance, if and how the design and implementation of the new care pathway might be biased due to accessibility issues.
You already hint at your interest in further exploring this issue in your "Future research" section. Still, you must reflect on whether your results are derived from the current pathway implementation (or some artefact of your sample, etc.)

Response: thank you for this suggestion that we have taken into consideration.

Please address all correspondence concerning this manuscript to me at s.williams35@herts.ac.uk.

Thank you for your consideration of this revised manuscript.

Sincerely,

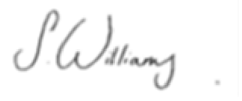

Dr Stefanie Williams
