# Supplementary material for: A mixed-methods analysis of the implementation of a new community long-COVID service during the 2020 pandemic: Learning from practice
Source: PLoS One. 2026 Jun 26;21(6):e0313367. doi: 10.1371/journal.pone.0313367 (PMC13308792; doi:10.1371/journal.pone.0313367)
Supplement: Attachment — Submitted filename: Comments.docx [file pone.0313367.s010.docx]

1- Make the background/ introduction more detailed for better contextualization (cite different other studies)
2- Use more than one resource to support your claim in lines (93, 94,95)

“ Healthcare professionals (HCPs) have reported a lack of existing referral pathways, service capacity pressures, lack of medical staff within LC service provision, resource issues, gaps in knowledge and lack of confidence in managing the condition as barriers to the effective implementation of community rehabilitation for LC [13].”

3- Admit your Reflexivity and address ( the bias that could be introduced such as selection bias/ Interviewer / observer bias and its impact on your results in line 172, . Also mention the percentage of (116/218) “Of 218 patients referred into the clinic, 116 provided consent by phone for their data to be analysed for research purposes. Their data were transferred to the data”
4- Admit your Reflexivity and potential biases in line 188, 189

“PW’s prior working relationship with clinicians may have influenced data collection dynamics, potentially shaping how information was recorded or shared ( e.g., response bias). However, PW had no prior relationship with patients, which reduced the risk of bias in patient–researcher interactions.”

5- Your statement on patient and public engagement is generic

You can rewrite it with the emphasis on “ **Strengths of your statement**

- You highlight that the **study aligns with patient priorities** (assessment, management, navigating care).
- You acknowledge **HCP involvement** in shaping aims and plans.
- You commit to **sharing findings with participants** and seeking their advice on dissemination.

6- Male: **34 (29.31%)/** Female: **82 (70.69%)** That’s more than **twice as many females as males** in the sample. How could this bias your findings?
7- This is a very confusing figure :

8- I think you'd better restructure your discussion for better reading ( select 2-3 emerging themes) make them as subthemes, and merge the other studies under each subtheme where it fits. It would read better.
9- Can you restructure the **Strengths and weaknesses of the study to subthemes because it's confusing**
10- **Nice Future research, but could be shortened.**
