# Supplementary material for: A mixed-methods analysis of the implementation of a new community long-COVID service during the 2020 pandemic: Learning from practice
Source: PLoS One. 2026 Jun 26;21(6):e0313367. doi: 10.1371/journal.pone.0313367 (PMC13308792; doi:10.1371/journal.pone.0313367)
Supplement: Attachment — Submitted filename: Response to reviewers.docx [file pone.0313367.s013.docx]

**Letter to Editor and Reviewers**

Dr Stefanie L. Williams

University of Hertfordshire

College Lane, Hatfield, AL10 9AB

16th February, 2026

Dear Editor,

We are pleased to resubmit our manuscript titled “A mixed-methods analysis of the implementation of a new community long-COVID service during the 2020 pandemic: learning from practice” for consideration by PLOS ONE.

We are thankful to reviewers #3 and #4 for their helpful suggestions.

We hereby respond to the request for revision [in blue], and we provide a point-by-point rebuttal to editing and reviewers’ requests.

Journal Requirements:

If the reviewer comments include a recommendation to cite specific previously published works, please review and evaluate these publications to determine whether they are relevant and should be cited. There is no requirement to cite these works unless the editor has indicated otherwise. 

Please review your reference list to ensure that it is complete and correct. If you have cited papers that have been retracted, please include the rationale for doing so in the manuscript text, or remove these references and replace them with relevant current references. Any changes to the reference list should be mentioned in the rebuttal letter that accompanies your revised manuscript. If you need to cite a retracted article, indicate the article’s retracted status in the References list and also include a citation and full reference for the retraction notice.

We have reviewed our reference list to ensure that it is complete and correct. Changes to the reference list are mentioned below.

Reviewers' comments:

Reviewer's Responses to Questions

**Comments to the Author**

1. If the authors have adequately addressed your comments raised in a previous round of review and you feel that this manuscript is now acceptable for publication, you may indicate that here to bypass the “Comments to the Author” section, enter your conflict of interest statement in the “Confidential to Editor” section, and submit your "Accept" recommendation.

Reviewer #3: All comments have been addressed

Reviewer #4: (No Response)

No action required.

2. Is the manuscript technically sound, and do the data support the conclusions?

The manuscript must describe a technically sound piece of scientific research with data that supports the conclusions. Experiments must have been conducted rigorously, with appropriate controls, replication, and sample sizes. The conclusions must be drawn appropriately based on the data presented.

Reviewer #3: Yes

Reviewer #4: Yes

No action required.

3. Has the statistical analysis been performed appropriately and rigorously?

Reviewer #3: Yes

Reviewer #4: Yes

No action required.

4. Have the authors made all data underlying the findings in their manuscript fully available?

The [PLOS Data policy](http://www.plosone.org/static/policies.action#sharing) requires authors to make all data underlying the findings described in their manuscript fully available without restriction, with rare exception (please refer to the Data Availability Statement in the manuscript PDF file). The data should be provided as part of the manuscript or its supporting information, or deposited to a public repository. For example, in addition to summary statistics, the data points behind means, medians and variance measures should be available. If there are restrictions on publicly sharing data—e.g. participant privacy or use of data from a third party—those must be specified.

Reviewer #3: Yes

Reviewer #4: Yes

No action required.

5. Is the manuscript presented in an intelligible fashion and written in standard English?

PLOS ONE does not copyedit accepted manuscripts, so the language in submitted articles must be clear, correct, and unambiguous. Any typographical or grammatical errors should be corrected at revision, so please note any specific errors here.

Reviewer #3: Yes

Reviewer #4: Yes

No action required.

6. Review Comments to the Author

Please use the space provided to explain your answers to the questions above. You may also include additional comments for the author, including concerns about dual publication, research ethics, or publication ethics. (Please upload your review as an attachment if it exceeds 20,000 characters)

Reviewer #3: (No Response)

Reviewer #4: Thank you for the opportunity to review this manuscript. The study addresses an important topic and presents useful findings. However, the introduction could provide more context, the methods need clarification, and the discussion would benefit from a clearer structure. Please address my comments to improve clarity and overall quality.

Comments provided as an attachment:

1- Make the background/ introduction more detailed for better contextualization (cite different other studies)

We added reference to recently published articles as requested to contextualize our study. Specifically, we added reference # 6, 7, 13, 14, 15, 16, 17, 19, 22, 23, 24, 27, 28, 35 (see list below)

2- Use more than one resource to support your claim in lines (93, 94,95)

“Healthcare professionals (HCPs) have reported a lack of existing referral pathways, service capacity pressures, lack of medical staff within LC service provision, resource issues, gaps in knowledge and lack of confidence in managing the condition as barriers to the effective implementation of community rehabilitation for LC [13].”

We added reference to published articles that support our statement.

3- Admit your Reflexivity and address the bias that could be introduced such as selection bias/ Interviewer / observer bias and its impact on your results in line 172, [the one with 116 in it]

Also mention the percentage of (116/218) “Of 218 patients referred into the clinic, 116 provided consent by phone for their data to be analysed for research purposes. Their data were transferred to the data”

4- Admit your Reflexivity and potential biases in line 188, 189

“PW’s prior working relationship with clinicians may have influenced data collection dynamics, potentially shaping how information was recorded or shared (e.g., response bias). However, PW had no prior relationship with patients, which reduced the risk of bias in patient–researcher interactions.”

We combined comments #3 and #4 since they reflect a similar suggestion.

We discussed reflexivity and selection/interviewer/observer/response bias in the text as suggested. We added reference # 41.

5- Your statement on patient and public engagement is generic.

You can rewrite it with the emphasis on “Strengths of your statement

- You highlight that the study aligns with patient priorities (assessment, management, navigating care).
- You acknowledge HCP involvement in shaping aims and plans.
- You commit to sharing findings with participants and seeking their advice on dissemination.

We have rewritten the paragraph taking into account the useful suggestions.

6- Male: 34 (29.31%)/ Female: 82 (70.69%) That’s more than twice as many females as males in the sample. How could this bias your findings?

We added a reflection on this disparity and its impact on findings.

7- This is a very confusing figure (2).

We have replaced this figure with one that we hope will be clearer.

8- I think you'd better restructure your discussion for better reading (select 2-3 emerging themes) make them as subthemes, and merge the other studies under each subtheme where it fits. It would read better.

We have rewritten the discussion taking into account the useful suggestions.

9- Can you restructure the Strengths and weaknesses of the study to subthemes because it's confusing.

We have rewritten this section taking into account the useful suggestion.

10- Nice Future research, but could be shortened.

We have shortened this section as suggested.

-----------------------------------------------

Please address all correspondence concerning this manuscript to me at s.williams35@herts.ac.uk.

Thank you for your consideration of this revised manuscript.

Sincerely,

Dr Stefanie Williams

New references list:

6, 7, 13, 14, 15, 16, 17, 19, 22, 23, 24, 27, 28, 35

6.	Sivan M, Heightman M, Mitchell JL, et al. Assessing long COVID symptom burden and functional outcomes in patients attending specialist clinics in England. J Infect. 2022;84(4):418–425. doi:10.1016/j.jinf.2021.12.033

7.	Whittaker HR, Gulea C, Koteci A, et al. Trajectories of health-related quality of life and functional limitations among people referred to long COVID clinics in England. BMJ Open. 2024;14(11):e088538. doi:10.1136/bmjopen-2024-088538

13.	Aiyar S, Heightman M, Darzi A, Banerjee A, the LOCOMOTION Consortium. LOCOMOTION: A national, multi-site study to optimise long COVID services across the NHS. BMJ Open. 2023;13(8):e072943. doi:10.1136/bmjopen-2023-072943

14.	Darbyshire J, Greenhalgh T, Bakerly ND, Balasundaram K, Baley S, Ball M, et al. Improving quality in adult long COVID services: Findings from the LOCOMOTION quality improvement collaborative. Clin Med. 2024;24(5):100237. doi:10.1016/j.clinme.2024.100237

15.	Routen A, O’Mahoney LL, Ayoubkhani D, et al. How long is long COVID? Evaluation of long-term health status in individuals discharged from a specialist community long COVID service. J Clin Med. 2024;13(19):5817. doi:10.3390/jcm13195817

16.	Ladds E, Rushforth A, Wieringa S, et al. Persistent symptoms after COVID-19: qualitative study of 114 “long COVID” patients and draft quality principles for services. BMC Health Serv Res. 2020;20:1144. doi:10.1186/s12913-020-06001-y

17.	Greenhalgh T, Darbyshire J, Ladds E, Van Dael J, Rayner C. Working knowledge, uncertainty and ontological politics: An ethnography of UK long COVID clinics. Sociol Health Illn. 2024;46(8):1881–1900. doi:10.1111/1467-9566.13819

19.	McLeod M, Sivan M, Russell AM, et al. Exploring perceptions of community rehabilitation for people with long COVID: A qualitative study. BMC Health Serv Res. 2024;24:512. doi:10.1186/s12913-024-10854-9

22.	Bek LM, Ista E, Berentschot JC, Hellemons ME, Aerts JGJV, Ribbers GM, Heijenbrok-Kal MH, van den Berg-Emons HJG. Healthcare professionals’ perspective on the organization of COVID-19 post-hospital aftercare: perspective, barriers and facilitators. Int J Healthc Manag. 2024:1–16.

23.	Razai MS, Oakeshott P, Kankam H, et al. Mitigating ethnic disparities in COVID-19 and long COVID. Lancet. 2021;397(10292):671–673. doi:10.1016/S0140-6736(21)00324-9

24.	Mir, G., Mullard, J., Parkin, A., Lee, C., Clarke, J., De Kock, J. H., Prociuk, D., Darbyshire, J. L., Evans, S., & Sivan, M. (2025). Addressing inequalities in long COVID healthcare: A mixed-methods study on building inclusive services. Health Expectations, 28(4), e70336. doi:10.1111/hex.70336

27.	Russell, A. M., Sivan, M., McLeod, M., et al. (2023). Understanding models of community-based long COVID rehabilitation services: A qualitative study. BMC Health Services Research, 23, 1213. doi: 10.1186/s12913-023-10128-1

28.	Gulea C, Whittaker HR, Koteci A, et al. Health service utilisation and economic outcomes associated with long COVID in England and Scotland. Lancet Reg Health Eur. 2024;39:100862. doi:10.1016/j.lanepe.2024.100862

35.	Wong CH, Yeoh EK, Zhong CC, Chung VC, Nilsen P, Wong EL. Barriers and Facilitators to Receiving the COVID-19 Vaccination and Development of Theoretically-Informed Implementation Strategies for the Public: Qualitative Study in Hong Kong. Vaccines (Basel). 2022;10. doi:10.3390/VACCINES10050764
