# Supplementary material for: A mixed-methods analysis of the implementation of a new community long-COVID service during the 2020 pandemic: Learning from practice
Source: PLoS One. 2026 Jun 26;21(6):e0313367. doi: 10.1371/journal.pone.0313367 (PMC13308792; doi:10.1371/journal.pone.0313367)
Supplement: Attachment — Submitted filename: comments 2.docx [file pone.0313367.s012.docx]

**Reviewer Comments:**

Thank you for addressing my previous comments; the revisions are appreciated. Overall, this is a well-conducted study and the authors should be commended for their work. The following suggestions are offered to further strengthen the manuscript:

**Discussion:** The strengths and limitations sections are currently presented separately with subtitles. I recommend removing these subtitles and integrating the content more concisely into the body of the Discussion, as is conventional in most journals. Similarly, the "Comparison with Other Studies" subtitle should be removed, with the comparative content woven naturally into the Discussion narrative.

**Recommendations/Conclusion:** The study recommendations section is overly lengthy as a standalone section. I suggest incorporating the key recommendations into the Discussion where relevant, and consolidating the remainder into a dedicated **Conclusion** section. This Conclusion section should also include a brief paragraph on **Recommendations for Future Research**, which would provide a more focused and impactful ending to the manuscript.

These revisions would improve the overall flow and conciseness of the paper without losing any substantive content.
