# Supplementary material for: A mixed-methods analysis of the implementation of a new community long-COVID service during the 2020 pandemic: Learning from practice
Source: PLoS One. 2026 Jun 26;21(6):e0313367. doi: 10.1371/journal.pone.0313367 (PMC13308792; doi:10.1371/journal.pone.0313367)
Supplement: Attachment — Submitted filename: Response_to_reviewers_auresp_4.docx [file pone.0313367.s014.docx]

**Letter to Editor and Reviewers**

Dr Stefanie L. Williams

University of Hertfordshire

College Lane, Hatfield, AL10 9AB

1^st^ May, 2026

Dear Editor,

We are pleased to resubmit our manuscript titled “A mixed-methods analysis of the implementation of a new community long-COVID service during the 2020 pandemic: learning from practice” for consideration by PLOS ONE.

We are thankful to reviewers #4 for their suggestions and to Marianne Clemence, Division Editor. Her response to our enquiry was very helpful: ***I have reviewed the most recent peer review of your manuscript, and I recognize that the most recent requests from Reviewer 4 relate to formatting, length and style, which are not related to the PLOS One publication criteria. These requests would therefore be considered optional.***

We hereby respond to the request for revision [in blue], and we provide a point-by-point rebuttal to editing and reviewers’ requests.

Journal Requirements:

If the reviewer comments include a recommendation to cite specific previously published works, please review and evaluate these publications to determine whether they are relevant and should be cited. There is no requirement to cite these works unless the editor has indicated otherwise. 

Please review your reference list to ensure that it is complete and correct. If you have cited papers that have been retracted, please include the rationale for doing so in the manuscript text, or remove these references and replace them with relevant current references. Any changes to the reference list should be mentioned in the rebuttal letter that accompanies your revised manuscript. If you need to cite a retracted article, indicate the article’s retracted status in the References list and also include a citation and full reference for the retraction notice.

We have reviewed our reference list to ensure that it is complete and correct.

We have deleted reference number 50 (added by mistake), 56 and 57 according to the shortening of text suggested by the reviewer.

Reviewers' comments:

Reviewer's Responses to Questions

**Comments to the Author**

1. If the authors have adequately addressed your comments raised in a previous round of review and you feel that this manuscript is now acceptable for publication, you may indicate that here to bypass the “Comments to the Author” section, enter your conflict of interest statement in the “Confidential to Editor” section, and submit your "Accept" recommendation.

Reviewer #4: (No Response)

No action required.

2. Is the manuscript technically sound, and do the data support the conclusions?

The manuscript must describe a technically sound piece of scientific research with data that supports the conclusions. Experiments must have been conducted rigorously, with appropriate controls, replication, and sample sizes. The conclusions must be drawn appropriately based on the data presented.

Reviewer #4: Yes

No action required.

3. Has the statistical analysis been performed appropriately and rigorously?

Reviewer #4: Yes

No action required.

4. Have the authors made all data underlying the findings in their manuscript fully available?

The [PLOS Data policy](http://www.plosone.org/static/policies.action#sharing) requires authors to make all data underlying the findings described in their manuscript fully available without restriction, with rare exception (please refer to the Data Availability Statement in the manuscript PDF file). The data should be provided as part of the manuscript or its supporting information, or deposited to a public repository. For example, in addition to summary statistics, the data points behind means, medians and variance measures should be available. If there are restrictions on publicly sharing data—e.g. participant privacy or use of data from a third party—those must be specified.

Reviewer #4: Yes

No action required.

5. Is the manuscript presented in an intelligible fashion and written in standard English?

PLOS ONE does not copyedit accepted manuscripts, so the language in submitted articles must be clear, correct, and unambiguous. Any typographical or grammatical errors should be corrected at revision, so please note any specific errors here.

Reviewer #4: Yes

No action required.

6. Review Comments to the Author

Please use the space provided to explain your answers to the questions above. You may also include additional comments for the author, including concerns about dual publication, research ethics, or publication ethics. (Please upload your review as an attachment if it exceeds 20,000 characters)

Reviewer #4: Thank you for addressing my previous comments; the revisions are appreciated. Overall, this is a well-conducted study and the authors should be commended for their work.

Thank you to review #4 for this encouraging message.

The following suggestions are offered to further strengthen the manuscript:

Discussion: The strengths and limitations sections are currently presented separately with subtitles. I recommend removing these subtitles and integrating the content more concisely into the body of the Discussion, as is conventional in most journals.

We have considered this suggestion carefully, we also asked a third opinion by an academic who is involved in reviewing papers for the REF (Research Excellence Framework (REF) is the UK's system for assessing the excellence of research in UK higher education institutions) - most papers do have a separate strengths and limitations, as also seen here

https://doi.org/10.1371/journal.pone.0347997

https://journals.plos.org/plosone/article?id=10.1371/journal.pone.0315486

https://journals.plos.org/plosone/article?id=10.1371/journal.pone.0289245

We have made the decision to keep the section separated for clarity but removing the headings as suggested.

Similarly, the "Comparison with Other Studies" subtitle should be removed, with the comparative content woven naturally into the Discussion narrative.

We have made this change and we are grateful for the suggestion.

Recommendations/Conclusion: The study recommendations section is overly lengthy as a standalone section. I suggest incorporating the key recommendations into the Discussion where relevant, and consolidating the remainder into a dedicated Conclusion section.

We have shortened the list of recommendations for the implementation and delivery of new LC clinics, we kept it as separate section for readers to find it quickly when looking for practical tips. We deleted references number 56 and 57.

This Conclusion section should also include a brief paragraph on Recommendations for Future Research, which would provide a more focused and impactful ending to the manuscript.

We created a separate conclusion section with the addition of the recommendations for future research.

These revisions would improve the overall flow and conciseness of the paper without losing any substantive content.

Thank you for the suggestions which improved the flow and conciseness of the paper.

-----------------------------------------------

Please address all correspondence concerning this manuscript to me at s.williams35@herts.ac.uk.

Thank you for your consideration of this revised manuscript.

Sincerely,

Dr Stefanie Williams
