# Supplementary material for: A mixed-methods analysis of the implementation of a new community long-COVID service during the 2020 pandemic: Learning from practice
Source: PLoS One. 2026 Jun 26;21(6):e0313367. doi: 10.1371/journal.pone.0313367 (PMC13308792; doi:10.1371/journal.pone.0313367)
Supplement: Peer review history

# Peer review: Decision Letter 0

**DOI:** 10.1371/journal.pone.0313367.r001

**Authors:** Mohammed Abutaleb

14 Nov 2024

PONE-D-24-46697

A mixed-methods analysis of the implementation of a new community long-COVID service during the 2020 pandemic: learning from practice.

PLOS ONE

Dear Dr. Williams,

Thank you for submitting your manuscript to PLOS ONE. After careful consideration, we feel that it has merit but does not fully meet PLOS ONE’s publication criteria as it currently stands. Therefore, we invite you to submit a revised version of the manuscript that addresses the points raised during the review process.

Please submit your revised manuscript by Dec 29 2024 11:59PM. If you will need more time than this to complete your revisions, please reply to this message or contact the journal office at plosone@plos.org. When you're ready to submit your revision, log on to https://www.editorialmanager.com/pone/ and select the ’submissions Needing Revision' folder to locate your manuscript file.

Please include the following items when submitting your revised manuscript:

- A rebuttal letter that responds to each point raised by the academic editor and reviewer(s). You should upload this letter as a separate file labeled 'Response to Reviewers'.
- A marked-up copy of your manuscript that highlights changes made to the original version. You should upload this as a separate file labeled 'Revised Manuscript with Track Changes'.
- An unmarked version of your revised paper without tracked changes. You should upload this as a separate file labeled 'Manuscript'.

If you would like to make changes to your financial disclosure, please include your updated statement in your cover letter. Guidelines for resubmitting your figure files are available below the reviewer comments at the end of this letter.

If applicable, we recommend that you deposit your laboratory protocols in protocols.io to enhance the reproducibility of your results. Protocols.io assigns your protocol its own identifier (DOI) so that it can be cited independently in the future. For instructions see: https://journals.plos.org/plosone/s/submission-guidelines#loc-laboratory-protocols. Additionally, PLOS ONE offers an option for publishing peer-reviewed Lab Protocol articles, which describe protocols hosted on protocols.io. Read more information on sharing protocols at https://plos.org/protocols?utm_medium=editorial-email&utm_source=authorletters&utm_campaign=protocols.

We look forward to receiving your revised manuscript.

Kind regards,

Mohammed Hussain Abutaleb, PhD

Academic Editor

PLOS ONE

Journal Requirements:

When submitting your revision, we need you to address these additional requirements.

1. Please ensure that your manuscript meets PLOS ONE’s style requirements, including those for file naming. The PLOS ONE style templates can be found at

https://journals.plos.org/plosone/s/file?id=wjVg/PLOSOne_formatting_sample_main_body.pdf and

https://journals.plos.org/plosone/s/file?id=ba62/PLOSOne_formatting_sample_title_authors_affiliations.pdf

2. We note that the grant information you provided in the ‘Funding Information’ and ‘Financial Disclosure’ sections do not match.

When you resubmit, please ensure that you provide the correct grant numbers for the awards you received for your study in the ‘Funding Information’ section.

3. Thank you for stating the following in the Competing Interests section:

“HM received funding for a long-COVID GP Fellowship, to undertake clinical work and research, from NHS East and North Hertfordshire CCG. HM’s role within the study was funded by the LOCOMOTION study funding (National Institute for Health and Care Research, grant ref: COV-LT2-0016). HM received payment and travel expenses for long-COVID-related presentations and expert opinion. The remaining authors have declared that no competing interests exist.”

Please confirm that this does not alter your adherence to all PLOS ONE policies on sharing data and materials, by including the following statement: "This does not alter our adherence to  PLOS ONE policies on sharing data and materials.” (as detailed online in our guide for authors http://journals.plos.org/plosone/s/competing-interests).  If there are restrictions on sharing of data and/or materials, please state these. Please note that we cannot proceed with consideration of your article until this information has been declared.

Please include your updated Competing Interests statement in your cover letter; we will change the online submission form on your behalf.

4. We note that you have indicated that there are restrictions to data sharing for this study. For studies involving human research participant data or other sensitive data, we encourage authors to share de-identified or anonymized data. However, when data cannot be publicly shared for ethical reasons, we allow authors to make their data sets available upon request. For information on unacceptable data access restrictions, please see http://journals.plos.org/plosone/s/data-availability#loc-unacceptable-data-access-restrictions.

Before we proceed with your manuscript, please address the following prompts:

a) If there are ethical or legal restrictions on sharing a de-identified data set, please explain them in detail (e.g., data contain potentially identifying or sensitive patient information, data are owned by a third-party organization, etc.) and who has imposed them (e.g., a Research Ethics Committee or Institutional Review Board, etc.). Please also provide contact information for a data access committee, ethics committee, or other institutional body to which data requests may be sent.

b) If there are no restrictions, please upload the minimal anonymized data set necessary to replicate your study findings to a stable, public repository and provide us with the relevant URLs, DOIs, or accession numbers. Please see http://www.bmj.com/content/340/bmj.c181.long for guidelines on how to de-identify and prepare clinical data for publication. For a list of recommended repositories, please see https://journals.plos.org/plosone/s/recommended-repositories. You also have the option of uploading the data as Supporting Information files, but we would recommend depositing data directly to a data repository if possible.

Please update your Data Availability statement in the submission form accordingly.

[Note: HTML markup is below. Please do not edit.]

Reviewers' comments:

Reviewer’s Responses to Questions

**Comments to the Author**

1. Is the manuscript technically sound, and do the data support the conclusions?

The manuscript must describe a technically sound piece of scientific research with data that supports the conclusions. Experiments must have been conducted rigorously, with appropriate controls, replication, and sample sizes. The conclusions must be drawn appropriately based on the data presented.

Reviewer #1: Partly

Reviewer #2: Yes

Reviewer #3: Yes

**********

2. Has the statistical analysis been performed appropriately and rigorously?

Reviewer #1: Yes

Reviewer #2: Yes

Reviewer #3: Yes

**********

3. Have the authors made all data underlying the findings in their manuscript fully available?

The PLOS Data policy requires authors to make all data underlying the findings described in their manuscript fully available without restriction, with rare exception (please refer to the Data Availability Statement in the manuscript PDF file). The data should be provided as part of the manuscript or its supporting information, or deposited to a public repository. For example, in addition to summary statistics, the data points behind means, medians and variance measures should be available. If there are restrictions on publicly sharing data—e.g. participant privacy or use of data from a third party—those must be specified.

Reviewer #1: Yes

Reviewer #2: Yes

Reviewer #3: Yes

**********

4. Is the manuscript presented in an intelligible fashion and written in standard English?

PLOS ONE does not copyedit accepted manuscripts, so the language in submitted articles must be clear, correct, and unambiguous. Any typographical or grammatical errors should be corrected at revision, so please note any specific errors here.

Reviewer #1: Yes

Reviewer #2: Yes

Reviewer #3: Yes

**********

5. Review Comments to the Author

Please use the space provided to explain your answers to the questions above. You may also include additional comments for the author, including concerns about dual publication, research ethics, or publication ethics. (Please upload your review as an attachment if it exceeds 20,000 characters)

Reviewer #1: A mixed-methods analysis of the implementation of a new long-term community service during the 2020 pandemic: learning from practice.

ABSTRACT Well-structured.

INTRODUCTION The authors divided the introduction into 4 subsections in which they explain the problem to be addressed, the organization of health services that serve patients with CL, explain the approach used with the TDF and then the objectives of the study.

METHODS The study was carried out using mixed methods, with the quantitative part being the collection of secondary data from patients and the qualitative approach based on a semi-structured questionnaire applied to patients and health professionals.

- local specifications;

- specify the study population;

- specify the period in which the study was completed; - the authors specify the analysis of quantitative data (descriptive statistics, standard deviation and brightness tests between variables, as well as the qualitative data were decoded and detailed. Subsequently, data triangulation was performed. - ethical precepts were followed RESULTS - table 1 is not visually appealing. It contains note lines, which is not found in a table - I suggest removing the variable from the table and leaving it described only in the paragraph above; the table only contains variables with N and % - all tables need to be better formatted to be more visually appealing to the reader, tables cannot have closed note lines, perhaps what the authors reserve for a table is to be defined, called a figure/chart. It is best to check with the newspaper’s editing standards; - the table titles also need to be reviewed, since they do not contain local data/data collection period;

DISCUSSION - in the first paragraph of the discussion there is no need to repeat the results, this is already described above;

- I understand that the first two items of the Discussion still fit into the Results. The authors, in fact, discuss the item Comparison with other studies;

- I suggest reviewing the need to separate the discussion into subtitles;

- the authors point out the weaknesses of the study, as well as highlighting the need for future studies.

Reviewer #2: The manuscript establishes a strong rationale for investigating long-COVID care pathways, given the condition’s rising prevalence and complexity. However, the authors can ensure that the introduction further clearly defines key terms, such as "long-COVID" and "Theoretical Domains Framework" (TDF), for readers who may not be so familiar, by elaborating on the gaps in existing literature that this study aims to address, which would help highlight the study’s uniqueness and importance.

Also, further methodological details could enhance replicability, such as deeply clarifying the data collection procedures, analysis methods (both qualitative and quantitative), and any steps taken to ensure reliability and validity.

Furthermore, the results provide valuable insights into patient needs, such as rehabilitation requirements and referrals to specialists. However, if feasible, the authors could provide more context or comparisons with similar studies to demonstrate how the findings align with or differ from the broader literature.

Overall, the presentation of quantitative results, table and tables is clear, further improving readability and making trends or key data points more immediately apparent to readers.

Implications and Recommendations

The manuscript could be strengthened by offering more concrete recommendations based on the findings. For instance, if "funding constraints" were a barrier, the authors could suggest specific policy or funding changes to support long-COVID services. Thus, highlighting actionable strategies that other clinics or healthcare providers could adopt would further enhance the study’s practical relevance.

Also, discussion on the limitations have helped to add balance to the interpretation, thus, helping the clarifications and generalizability of findings to other settings or populations.

Lastly, could the authors further mention (if available) any unanswered questions or emerging needs related to long-COVID care as observed during the study?

Reviewer #3: Dear Authors,

I have thoroughly reviewed the manuscript.

At this point, some minor revisions are still worth addressing before recommending your manuscript for publication.

Minor comments:

1—On page 10, line 231, please leave out the ‘causality’ exploration as it is outside the scope of the analysis, not supported by the proposed methodological framework, and not derived from your results.

2- Please discuss the potential equity concerns regarding your results observing patients of the Long-Covid pathway to be predominantly female, white British people from non-deprived areas. You might reflect, for instance, if and how the design and implementation of the new care pathway might be biased due to accessibility issues.

You already hint at your interest in further exploring this issue in your “Future research” section. Still, you must reflect on whether your results are derived from the current pathway implementation (or some artefact of your sample, etc.)

Kind regards,

**********

6. PLOS authors have the option to publish the peer review history of their article (what does this mean?). If published, this will include your full peer review and any attached files.

If you choose “no”, your identity will remain anonymous but your review may still be made public.

**Do you want your identity to be public for this peer review?**  For information about this choice, including consent withdrawal, please see our Privacy Policy.

Reviewer #1: **Yes:**  Maria Eugenia Firmino Brunello (Brunello, MEF)

Reviewer #2: **Yes:**  ALA Oluwafemi Ajoyemi

Reviewer #3: **Yes:**  Francisco Estupiñán-Romero

**********

[NOTE: If reviewer comments were submitted as an attachment file, they will be attached to this email and accessible via the submission site. Please log into your account, locate the manuscript record, and check for the action link "View Attachments". If this link does not appear, there are no attachment files.]

While revising your submission, please upload your figure files to the Preflight Analysis and Conversion Engine (PACE) digital diagnostic tool, https://pacev2.apexcovantage.com/. PACE helps ensure that figures meet PLOS requirements. To use PACE, you must first register as a user. Registration is free. Then, login and navigate to the UPLOAD tab, where you will find detailed instructions on how to use the tool. If you encounter any issues or have any questions when using PACE, please email PLOS at figures@plos.org. Please note that Supporting Information files do not need this step.

# Author comment: Author response to Decision Letter 1

**DOI:** 10.1371/journal.pone.0313367.r002

3 Jan 2025

The point by point response to reviewer and editor comments is included in the file named 'Response to reviewers'

Attachment Submitted filename: Response to reviewers.pdf

# Peer review: Decision Letter 1

**DOI:** 10.1371/journal.pone.0313367.r003

**Authors:** Mohammed Abutaleb, Mohammed Abutaleb

26 Mar 2025

PONE-D-24-46697R1

A mixed-methods analysis of the implementation of a new community long-COVID service during the 2020 pandemic: learning from practice.

PLOS ONE

Dear Dr. Williams,

Thank you for submitting your manuscript to PLOS ONE. After careful consideration, we feel that it has merit but does not fully meet PLOS ONE’s publication criteria as it currently stands. Therefore, we invite you to submit a revised version of the manuscript that addresses the points raised during the review process.

Please submit your revised manuscript by May 10 2025 11:59PM. If you will need more time than this to complete your revisions, please reply to this message or contact the journal office at plosone@plos.org. When you're ready to submit your revision, log on to https://www.editorialmanager.com/pone/ and select the ’submissions Needing Revision' folder to locate your manuscript file.

Please include the following items when submitting your revised manuscript:

- A rebuttal letter that responds to each point raised by the academic editor and reviewer(s). You should upload this letter as a separate file labeled 'Response to Reviewers'.
- A marked-up copy of your manuscript that highlights changes made to the original version. You should upload this as a separate file labeled 'Revised Manuscript with Track Changes'.
- An unmarked version of your revised paper without tracked changes. You should upload this as a separate file labeled 'Manuscript'.

-->If you would like to make changes to your financial disclosure, please include your updated statement in your cover letter. Guidelines for resubmitting your figure files are available below the reviewer comments at the end of this letter.

If applicable, we recommend that you deposit your laboratory protocols in protocols.io to enhance the reproducibility of your results. Protocols.io assigns your protocol its own identifier (DOI) so that it can be cited independently in the future. For instructions see: https://journals.plos.org/plosone/s/submission-guidelines#loc-laboratory-protocols. Additionally, PLOS ONE offers an option for publishing peer-reviewed Lab Protocol articles, which describe protocols hosted on protocols.io. Read more information on sharing protocols at https://plos.org/protocols?utm_medium=editorial-email&utm_source=authorletters&utm_campaign=protocols.

We look forward to receiving your revised manuscript.

Kind regards,

Mohammed Abutaleb, PhD

Academic Editor

PLOS ONE

Journal Requirements:

Please review your reference list to ensure that it is complete and correct. If you have cited papers that have been retracted, please include the rationale for doing so in the manuscript text, or remove these references and replace them with relevant current references. Any changes to the reference list should be mentioned in the rebuttal letter that accompanies your revised manuscript. If you need to cite a retracted article, indicate the article’s retracted status in the References list and also include a citation and full reference for the retraction notice.

[Note: HTML markup is below. Please do not edit.]

Reviewers' comments:

Reviewer’s Responses to Questions

**Comments to the Author**

1. If the authors have adequately addressed your comments raised in a previous round of review and you feel that this manuscript is now acceptable for publication, you may indicate that here to bypass the “Comments to the Author” section, enter your conflict of interest statement in the “Confidential to Editor” section, and submit your "Accept" recommendation.

Reviewer #1: (No Response)

Reviewer #2: All comments have been addressed

Reviewer #3: All comments have been addressed

**********

2. Is the manuscript technically sound, and do the data support the conclusions?

The manuscript must describe a technically sound piece of scientific research with data that supports the conclusions. Experiments must have been conducted rigorously, with appropriate controls, replication, and sample sizes. The conclusions must be drawn appropriately based on the data presented.

Reviewer #1: Partly

Reviewer #2: Yes

Reviewer #3: Yes

**********

3. Has the statistical analysis been performed appropriately and rigorously?

Reviewer #1: Yes

Reviewer #2: Yes

Reviewer #3: N/A

**********

4. Have the authors made all data underlying the findings in their manuscript fully available?

The PLOS Data policy requires authors to make all data underlying the findings described in their manuscript fully available without restriction, with rare exception (please refer to the Data Availability Statement in the manuscript PDF file). The data should be provided as part of the manuscript or its supporting information, or deposited to a public repository. For example, in addition to summary statistics, the data points behind means, medians and variance measures should be available. If there are restrictions on publicly sharing data—e.g. participant privacy or use of data from a third party—those must be specified.

Reviewer #1: Yes

Reviewer #2: Yes

Reviewer #3: Yes

**********

5. Is the manuscript presented in an intelligible fashion and written in standard English?

PLOS ONE does not copyedit accepted manuscripts, so the language in submitted articles must be clear, correct, and unambiguous. Any typographical or grammatical errors should be corrected at revision, so please note any specific errors here.

Reviewer #1: Yes

Reviewer #2: Yes

Reviewer #3: Yes

**********

6. Review Comments to the Author

Please use the space provided to explain your answers to the questions above. You may also include additional comments for the author, including concerns about dual publication, research ethics, or publication ethics. (Please upload your review as an attachment if it exceeds 20,000 characters)

Reviewer #1: In the previous version, notes were made that were not accepted by the authors and I think that it would be appropriate for the fluidity of reading and understanding of the present work.

**RESULTS**

- Table 1 is not visually appealing. It contains grid lines, which are not recommended for a table.

- I suggest removing the age variable from the table and describing it only in the paragraph above. Thus, I recommend that the table only includes variables with N and %.

- All tables need to be better formatted to be more visually pleasant to the reader. I emphasize that tables should not have closed grid lines. Perhaps what the authors are calling a table should be called a figure/chart. It is better to check with the journal’s editing guidelines.

- The table titles also need to be revised, as they do not include the location and date/period of data collection.

**DISCUSSION**

- In the first paragraph of the discussion, there is no need to repeat the results, as they are already described above.

- I understand that the first two items of the Discussion still fit into the Results section. The authors indeed discuss in the item "Comparison with Other Studies."

The present study provides rich and important information on a current topic and highlights the need for greater knowledge about LC. However, I believe the authors should review and better structure the way the study is presented, according to previous comments. I reinforce that the results are important, but the presentation needs improvement.

Reviewer #2: All the points and "issues" I raised in the last round of review have been addressed and detailed out appropriately.

Reviewer #3: (No Response)

**********

7. PLOS authors have the option to publish the peer review history of their article (what does this mean?). If published, this will include your full peer review and any attached files.

If you choose “no”, your identity will remain anonymous but your review may still be made public.

**Do you want your identity to be public for this peer review?**  For information about this choice, including consent withdrawal, please see our Privacy Policy.

Reviewer #1: No

Reviewer #2: **Yes**

Reviewer #3: No

**********

[NOTE: If reviewer comments were submitted as an attachment file, they will be attached to this email and accessible via the submission site. Please log into your account, locate the manuscript record, and check for the action link "View Attachments". If this link does not appear, there are no attachment files.]

While revising your submission, please upload your figure files to the Preflight Analysis and Conversion Engine (PACE) digital diagnostic tool, https://pacev2.apexcovantage.com/. PACE helps ensure that figures meet PLOS requirements. To use PACE, you must first register as a user. Registration is free. Then, login and navigate to the UPLOAD tab, where you will find detailed instructions on how to use the tool. If you encounter any issues or have any questions when using PACE, please email PLOS at figures@plos.org. Please note that Supporting Information files do not need this step.

# Author comment: Author response to Decision Letter 2

**DOI:** 10.1371/journal.pone.0313367.r004

27 Aug 2025

Dear Editor Dr Mohammed Abutaleb and Reviewers,

Thank you for your request to revise our paper to which we responded via a letter dated 03 April 2025.

We want to reassure the reviewers every comment was taken into consideration and responded to in our previous rebuttal letter dated 31 Dec 2024 (attached) and in the second version of the paper.

We wonder if reviewer #1 considered the revised version of our article and figures/table, since the listing of original and revised documents may have been misleading. All the changes they suggested during the first review were made and clearly explained in the revised version with changes highlighted.

In particular we refer to reviewer #1 comments, hereby between ** **, our reply is found after >>>:

**RESULTS

- Table 1 is not visually appealing. It contains grid lines, which are not recommended for a table.

- I suggest removing the age variable from the table and describing it only in the paragraph above. Thus, I recommend that the table only includes variables with N and %.

- All tables need to be better formatted to be more visually pleasant to the reader. I emphasize that tables should not have closed grid lines. Perhaps what the authors are calling a table should be called a figure/chart. It is better to check with the journal’s editing guidelines.

- The table titles also need to be revised, as they do not include the location and date/period of data collection.**

>>> Response (from December rebuttal letter): thank you for this suggestion, table 1 and 3 were changed to figure 1 (line 285 of Manuscript revison3) with infographics to provide a clearer description of the patients’ population and figure 4 (line 554) showing barriers and facilitators to care and service implementation. We checked the editing guidelines of the journal, other manuscripts published by PLOSONE, and addressed this comment accordingly.

>>> Additional comment: Table 1 (line 390) is now referring to a different part of the results: TDF domains and subdomains across patients and HCPs.

We changed the format of the table removing grid lines, we thought that was done when the paper is edited by the journal for publishing, we will wait for your advice on this. We did add the location and date of data collection in the title.

**DISCUSSION

- In the first paragraph of the discussion, there is no need to repeat the results, as they are already described above.

- I understand that the first two items of the Discussion still fit into the Results section. The authors indeed discuss in the item "Comparison with Other Studies."**

>>> Response (rebuttal letter): thank you for these comments, we have reviewed the relevant sections of results and discussion to avoid duplication, and use the statement of findings (line 529: Statement of principal findings) to summarise the results at the end of the results section, we believe this is good practice and also suggested by publishing guidelines.

We understand that, when the previously submitted PDF was downloaded, the original submission appeared first, therefore creating confusion.

Please, let us know if, in your opinion, the above-mentioned changes, found in the revised version of the paper and new table/figures, are in accordance with the reviewer’s comments.

Best regards

Stefanie Williams

Attachment Submitted filename: Response to reviewers 13Aug25.pdf

# Peer review: Decision Letter 2

**DOI:** 10.1371/journal.pone.0313367.r005

**Authors:** Mohammed Abutaleb, Mohammed Abutaleb, Ilse Bloom

16 Dec 2025

PONE-D-24-46697R2

A mixed-methods analysis of the implementation of a new community long-COVID service during the 2020 pandemic: learning from practice.

PLOS One

Dear Dr. Williams,

Thank you for submitting your manuscript to PLOS ONE. After careful consideration, we feel that it has merit but does not fully meet PLOS ONE’s publication criteria as it currently stands. Therefore, we invite you to submit a revised version of the manuscript that addresses the points raised during the review process.

The manuscript has been further evaluated, and the reviewer comments are available in the attached file.

The reviewer has requested more contextual detail in the Introduction, greater methodological detail, notably regarding potential sources of bias, and some alterations to the structure of the Discussion section.

Could you please carefully revise the manuscript to address all comments raised?

Please submit your revised manuscript by Jan 30 2026 11:59PM. If you will need more time than this to complete your revisions, please reply to this message or contact the journal office at plosone@plos.org. When you're ready to submit your revision, log on to https://www.editorialmanager.com/pone/ and select the ’submissions Needing Revision' folder to locate your manuscript file.

Please include the following items when submitting your revised manuscript:

- A letter that responds to each point raised by the academic editor and reviewer(s). You should upload this letter as a separate file labeled 'Response to Reviewers'.
- A marked-up copy of your manuscript that highlights changes made to the original version. You should upload this as a separate file labeled 'Revised Manuscript with Track Changes'.
- An unmarked version of your revised paper without tracked changes. You should upload this as a separate file labeled 'Manuscript'.

If you would like to make changes to your financial disclosure, please include your updated statement in your cover letter. Guidelines for resubmitting your figure files are available below the reviewer comments at the end of this letter.

If applicable, we recommend that you deposit your laboratory protocols in protocols.io to enhance the reproducibility of your results. Protocols.io assigns your protocol its own identifier (DOI) so that it can be cited independently in the future. For instructions see: https://journals.plos.org/plosone/s/submission-guidelines#loc-laboratory-protocols. Additionally, PLOS ONE offers an option for publishing peer-reviewed Lab Protocol articles, which describe protocols hosted on protocols.io. Read more information on sharing protocols at https://plos.org/protocols?utm_medium=editorial-email&utm_source=authorletters&utm_campaign=protocols.

We look forward to receiving your revised manuscript.

Kind regards,

Ilse Bloom

Staff Editor

PLOS One

Journal Requirements:

If the reviewer comments include a recommendation to cite specific previously published works, please review and evaluate these publications to determine whether they are relevant and should be cited. There is no requirement to cite these works unless the editor has indicated otherwise.

Please review your reference list to ensure that it is complete and correct. If you have cited papers that have been retracted, please include the rationale for doing so in the manuscript text, or remove these references and replace them with relevant current references. Any changes to the reference list should be mentioned in the rebuttal letter that accompanies your revised manuscript. If you need to cite a retracted article, indicate the article’s retracted status in the References list and also include a citation and full reference for the retraction notice.

[Note: HTML markup is below. Please do not edit.]

Reviewers' comments:

Reviewer’s Responses to Questions

**Comments to the Author**

1. If the authors have adequately addressed your comments raised in a previous round of review and you feel that this manuscript is now acceptable for publication, you may indicate that here to bypass the “Comments to the Author” section, enter your conflict of interest statement in the “Confidential to Editor” section, and submit your "Accept" recommendation.

Reviewer #3: All comments have been addressed

Reviewer #4: (No Response)

**********

2. Is the manuscript technically sound, and do the data support the conclusions?

The manuscript must describe a technically sound piece of scientific research with data that supports the conclusions. Experiments must have been conducted rigorously, with appropriate controls, replication, and sample sizes. The conclusions must be drawn appropriately based on the data presented.

Reviewer #3: Yes

Reviewer #4: Yes

**********

3. Has the statistical analysis been performed appropriately and rigorously?

Reviewer #3: Yes

Reviewer #4: Yes

**********

4. Have the authors made all data underlying the findings in their manuscript fully available?

The PLOS Data policy requires authors to make all data underlying the findings described in their manuscript fully available without restriction, with rare exception (please refer to the Data Availability Statement in the manuscript PDF file). The data should be provided as part of the manuscript or its supporting information, or deposited to a public repository. For example, in addition to summary statistics, the data points behind means, medians and variance measures should be available. If there are restrictions on publicly sharing data—e.g. participant privacy or use of data from a third party—those must be specified.

Reviewer #3: Yes

Reviewer #4: Yes

**********

5. Is the manuscript presented in an intelligible fashion and written in standard English?

PLOS ONE does not copyedit accepted manuscripts, so the language in submitted articles must be clear, correct, and unambiguous. Any typographical or grammatical errors should be corrected at revision, so please note any specific errors here.

Reviewer #3: Yes

Reviewer #4: Yes

**********

6. Review Comments to the Author

Please use the space provided to explain your answers to the questions above. You may also include additional comments for the author, including concerns about dual publication, research ethics, or publication ethics. (Please upload your review as an attachment if it exceeds 20,000 characters)

Reviewer #3: (No Response)

Reviewer #4: Thank you for the opportunity to review this manuscript. The study addresses an important topic and presents useful findings. However, the introduction could provide more context, the methods need clarification, and the discussion would benefit from a clearer structure. Please address my comments to improve clarity and overall quality.

**********

7. PLOS authors have the option to publish the peer review history of their article (what does this mean?). If published, this will include your full peer review and any attached files.

If you choose “no”, your identity will remain anonymous but your review may still be made public.

**Do you want your identity to be public for this peer review?**  For information about this choice, including consent withdrawal, please see our Privacy Policy.

Reviewer #3: No

Reviewer #4: **Yes:**  Reem Yehia Kamel Elsayed

**********

[NOTE: If reviewer comments were submitted as an attachment file, they will be attached to this email and accessible via the submission site. Please log into your account, locate the manuscript record, and check for the action link "View Attachments". If this link does not appear, there are no attachment files.]

To ensure your figures meet our technical requirements, please review our figure guidelines: https://journals.plos.org/plosone/s/figures

You may also use PLOS’s free figure tool, NAAS, to help you prepare publication quality figures: https://journals.plos.org/plosone/s/figures#loc-tools-for-figure-preparation.

NAAS will assess whether your figures meet our technical requirements by comparing each figure against our figure specifications.

Attachment Submitted filename: Comments.docx

# Author comment: Author response to Decision Letter 3

**DOI:** 10.1371/journal.pone.0313367.r006

16 Feb 2026

Dear Editor,

We are pleased to resubmit our manuscript titled “A mixed-methods analysis of the implementation of a new community long-COVID service during the 2020 pandemic: learning from practice” for consideration by PLOS ONE.

We are thankful to reviewers #3 and #4 for their helpful suggestions.

We hereby respond to the request for revision, and we provide a point-by-point rebuttal to editing and reviewers’ requests.

Journal Requirements:

If the reviewer comments include a recommendation to cite specific previously published works, please review and evaluate these publications to determine whether they are relevant and should be cited. There is no requirement to cite these works unless the editor has indicated otherwise.

Please review your reference list to ensure that it is complete and correct. If you have cited papers that have been retracted, please include the rationale for doing so in the manuscript text, or remove these references and replace them with relevant current references. Any changes to the reference list should be mentioned in the rebuttal letter that accompanies your revised manuscript. If you need to cite a retracted article, indicate the article’s retracted status in the References list and also include a citation and full reference for the retraction notice.

We have reviewed our reference list to ensure that it is complete and correct. Changes to the reference list are mentioned below.

Reviewers' comments:

Reviewer’s Responses to Questions

Comments to the Author

1. If the authors have adequately addressed your comments raised in a previous round of review and you feel that this manuscript is now acceptable for publication, you may indicate that here to bypass the “Comments to the Author” section, enter your conflict of interest statement in the “Confidential to Editor” section, and submit your "Accept" recommendation.

Reviewer #3: All comments have been addressed

Reviewer #4: (No Response)

No action required.

2. Is the manuscript technically sound, and do the data support the conclusions?

The manuscript must describe a technically sound piece of scientific research with data that supports the conclusions. Experiments must have been conducted rigorously, with appropriate controls, replication, and sample sizes. The conclusions must be drawn appropriately based on the data presented.

Reviewer #3: Yes

Reviewer #4: Yes

No action required.

3. Has the statistical analysis been performed appropriately and rigorously?

Reviewer #3: Yes

Reviewer #4: Yes

No action required.

4. Have the authors made all data underlying the findings in their manuscript fully available?

The PLOS Data policy requires authors to make all data underlying the findings described in their manuscript fully available without restriction, with rare exception (please refer to the Data Availability Statement in the manuscript PDF file). The data should be provided as part of the manuscript or its supporting information, or deposited to a public repository. For example, in addition to summary statistics, the data points behind means, medians and variance measures should be available. If there are restrictions on publicly sharing data—e.g. participant privacy or use of data from a third party—those must be specified.

Reviewer #3: Yes

Reviewer #4: Yes

No action required.

5. Is the manuscript presented in an intelligible fashion and written in standard English?

PLOS ONE does not copyedit accepted manuscripts, so the language in submitted articles must be clear, correct, and unambiguous. Any typographical or grammatical errors should be corrected at revision, so please note any specific errors here.

Reviewer #3: Yes

Reviewer #4: Yes

No action required.

6. Review Comments to the Author

Please use the space provided to explain your answers to the questions above. You may also include additional comments for the author, including concerns about dual publication, research ethics, or publication ethics. (Please upload your review as an attachment if it exceeds 20,000 characters)

Reviewer #3: (No Response)

Reviewer #4: Thank you for the opportunity to review this manuscript. The study addresses an important topic and presents useful findings. However, the introduction could provide more context, the methods need clarification, and the discussion would benefit from a clearer structure. Please address my comments to improve clarity and overall quality.

Comments provided as an attachment:

1- Make the background/ introduction more detailed for better contextualization (cite different other studies)

We added reference to recently published articles as requested to contextualize our study. Specifically, we added reference # 6, 7, 13, 14, 15, 16, 17, 19, 22, 23, 24, 27, 28, 35 (see letter attached)

2- Use more than one resource to support your claim in lines (93, 94,95)

“Healthcare professionals (HCPs) have reported a lack of existing referral pathways, service capacity pressures, lack of medical staff within LC service provision, resource issues, gaps in knowledge and lack of confidence in managing the condition as barriers to the effective implementation of community rehabilitation for LC [13].”

We added reference to published articles that support our statement.

3- Admit your Reflexivity and address the bias that could be introduced such as selection bias/ Interviewer / observer bias and its impact on your results in line 172, [the one with 116 in it]

Also mention the percentage of (116/218) “Of 218 patients referred into the clinic, 116 provided consent by phone for their data to be analysed for research purposes. Their data were transferred to the data”

4- Admit your Reflexivity and potential biases in line 188, 189

“PW’s prior working relationship with clinicians may have influenced data collection dynamics, potentially shaping how information was recorded or shared (e.g., response bias). However, PW had no prior relationship with patients, which reduced the risk of bias in patient–researcher interactions.”

We combined comments #3 and #4 since they reflect a similar suggestion.

We discussed reflexivity and selection/interviewer/observer/response bias in the text as suggested. We added reference # 41.

5- Your statement on patient and public engagement is generic.

You can rewrite it with the emphasis on “Strengths of your statement

• You highlight that the study aligns with patient priorities (assessment, management, navigating care).

• You acknowledge HCP involvement in shaping aims and plans.

• You commit to sharing findings with participants and seeking their advice on dissemination.

We have rewritten the paragraph taking into account the useful suggestions.

6- Male: 34 (29.31%)/ Female: 82 (70.69%) That’s more than twice as many females as males in the sample. How could this bias your findings?

We added a reflection on this disparity and its impact on findings.

7- This is a very confusing figure (2).

We have replaced this figure with one that we hope will be clearer.

8- I think you’d better restructure your discussion for better reading (select 2-3 emerging themes) make them as subthemes, and merge the other studies under each subtheme where it fits. It would read better.

We have rewritten the discussion taking into account the useful suggestions.

9- Can you restructure the Strengths and weaknesses of the study to subthemes because it’s confusing.

We have rewritten this section taking into account the useful suggestion.

10- Nice Future research, but could be shortened.

We have shortened this section as suggested.

-----------------------------------------------

Please address all correspondence concerning this manuscript to me at s.williams35@herts.ac.uk.

Thank you for your consideration of this revised manuscript.

Sincerely,

Dr Stefanie Williams

Attachment Submitted filename: Response to reviewers.docx

# Peer review: Decision Letter 3

**DOI:** 10.1371/journal.pone.0313367.r007

**Authors:** Mohammed Abutaleb, Mohammed Abutaleb, Ilse Bloom, Ilse Bloom

17 Mar 2026

PONE-D-24-46697R3

A mixed-methods analysis of the implementation of a new community long-COVID service during the 2020 pandemic: learning from practice.

PLOS One

Dear Dr. Williams,

Thank you for submitting your manuscript to PLOS ONE. After careful consideration, we feel that it has merit but does not fully meet PLOS ONE’s publication criteria as it currently stands. Therefore, we invite you to submit a revised version of the manuscript that addresses the points raised during the review process.

The manuscript has been further evaluated by one reviewer, and the reviewer comments can be found below.

Could you please carefully revise the manuscript to address all comments raised?

Please submit your revised manuscript by May 01 2026 11:59PM. If you will need more time than this to complete your revisions, please reply to this message or contact the journal office at plosone@plos.org. When you're ready to submit your revision, log on to https://www.editorialmanager.com/pone/ and select the ’submissions Needing Revision' folder to locate your manuscript file.

Please include the following items when submitting your revised manuscript:

- A letter that responds to each point raised by the academic editor and reviewer(s). You should upload this letter as a separate file labeled 'Response to Reviewers'.
- A marked-up copy of your manuscript that highlights changes made to the original version. You should upload this as a separate file labeled 'Revised Manuscript with Track Changes'.
- An unmarked version of your revised paper without tracked changes. You should upload this as a separate file labeled 'Manuscript'.

If you would like to make changes to your financial disclosure, please include your updated statement in your cover letter. Guidelines for resubmitting your figure files are available below the reviewer comments at the end of this letter.

If applicable, we recommend that you deposit your laboratory protocols in protocols.io to enhance the reproducibility of your results. Protocols.io assigns your protocol its own identifier (DOI) so that it can be cited independently in the future. For instructions see: https://journals.plos.org/plosone/s/submission-guidelines#loc-laboratory-protocols. Additionally, PLOS ONE offers an option for publishing peer-reviewed Lab Protocol articles, which describe protocols hosted on protocols.io. Read more information on sharing protocols at https://plos.org/protocols?utm_medium=editorial-email&utm_source=authorletters&utm_campaign=protocols.

We look forward to receiving your revised manuscript.

Kind regards,

Ilse Bloom

Staff Editor

PLOS One

Journal Requirements:

1. If the reviewer comments include a recommendation to cite specific previously published works, please review and evaluate these publications to determine whether they are relevant and should be cited. There is no requirement to cite these works unless the editor has indicated otherwise.

2. Please review your reference list to ensure that it is complete and correct. If you have cited papers that have been retracted, please include the rationale for doing so in the manuscript text, or remove these references and replace them with relevant current references. Any changes to the reference list should be mentioned in the rebuttal letter that accompanies your revised manuscript. If you need to cite a retracted article, indicate the article’s retracted status in the References list and also include a citation and full reference for the retraction notice.

[Note: HTML markup is below. Please do not edit.]

Reviewers' comments:

Reviewer’s Responses to Questions

**Comments to the Author**

1. If the authors have adequately addressed your comments raised in a previous round of review and you feel that this manuscript is now acceptable for publication, you may indicate that here to bypass the “Comments to the Author” section, enter your conflict of interest statement in the “Confidential to Editor” section, and submit your "Accept" recommendation.

Reviewer #4: (No Response)

**********

2. Is the manuscript technically sound, and do the data support the conclusions?

The manuscript must describe a technically sound piece of scientific research with data that supports the conclusions. Experiments must have been conducted rigorously, with appropriate controls, replication, and sample sizes. The conclusions must be drawn appropriately based on the data presented.

Reviewer #4: Yes

**********

3. Has the statistical analysis been performed appropriately and rigorously?

Reviewer #4: Yes

**********

4. Have the authors made all data underlying the findings in their manuscript fully available?

The PLOS Data policy requires authors to make all data underlying the findings described in their manuscript fully available without restriction, with rare exception (please refer to the Data Availability Statement in the manuscript PDF file). The data should be provided as part of the manuscript or its supporting information, or deposited to a public repository. For example, in addition to summary statistics, the data points behind means, medians and variance measures should be available. If there are restrictions on publicly sharing data—e.g. participant privacy or use of data from a third party—those must be specified.

Reviewer #4: Yes

**********

5. Is the manuscript presented in an intelligible fashion and written in standard English?

PLOS ONE does not copyedit accepted manuscripts, so the language in submitted articles must be clear, correct, and unambiguous. Any typographical or grammatical errors should be corrected at revision, so please note any specific errors here.

Reviewer #4: Yes

**********

6. Review Comments to the Author

Please use the space provided to explain your answers to the questions above. You may also include additional comments for the author, including concerns about dual publication, research ethics, or publication ethics. (Please upload your review as an attachment if it exceeds 20,000 characters)

Reviewer #4: Thank you for addressing my previous comments; the revisions are appreciated. Overall, this is a well-conducted study and the authors should be commended for their work. The following suggestions are offered to further strengthen the manuscript:

Discussion: The strengths and limitations sections are currently presented separately with subtitles. I recommend removing these subtitles and integrating the content more concisely into the body of the Discussion, as is conventional in most journals. Similarly, the "Comparison with Other Studies" subtitle should be removed, with the comparative content woven naturally into the Discussion narrative.

Recommendations/Conclusion: The study recommendations section is overly lengthy as a standalone section. I suggest incorporating the key recommendations into the Discussion where relevant, and consolidating the remainder into a dedicated Conclusion section. This Conclusion section should also include a brief paragraph on Recommendations for Future Research, which would provide a more focused and impactful ending to the manuscript.

These revisions would improve the overall flow and conciseness of the paper without losing any substantive content.

**********

7. PLOS authors have the option to publish the peer review history of their article (what does this mean?). If published, this will include your full peer review and any attached files.

If you choose “no”, your identity will remain anonymous but your review may still be made public.

**Do you want your identity to be public for this peer review?**  For information about this choice, including consent withdrawal, please see our Privacy Policy.

Reviewer #4: No

**********

[NOTE: If reviewer comments were submitted as an attachment file, they will be attached to this email and accessible via the submission site. Please log into your account, locate the manuscript record, and check for the action link "View Attachments". If this link does not appear, there are no attachment files.]

To ensure your figures meet our technical requirements, please review our figure guidelines: https://journals.plos.org/plosone/s/figures

You may also use PLOS’s free figure tool, NAAS, to help you prepare publication quality figures: https://journals.plos.org/plosone/s/figures#loc-tools-for-figure-preparation.

NAAS will assess whether your figures meet our technical requirements by comparing each figure against our figure specifications.

Attachment Submitted filename: comments 2.docx

# Author comment: Author response to Decision Letter 4

**DOI:** 10.1371/journal.pone.0313367.r008

1 May 2026

Dear Editor,

We are pleased to resubmit our manuscript titled “A mixed-methods analysis of the implementation of a new community long-COVID service during the 2020 pandemic: learning from practice” for consideration by PLOS ONE.

We are thankful to reviewers #4 for their suggestions and to Marianne Clemence, Division Editor. Her response to our enquiry was very helpful: I have reviewed the most recent peer review of your manuscript, and I recognize that the most recent requests from Reviewer 4 relate to formatting, length and style, which are not related to the PLOS One publication criteria. These requests would therefore be considered optional.

We hereby respond to the request for revision [***], and we provide a point-by-point rebuttal to editing and reviewers’ requests.

Journal Requirements:

If the reviewer comments include a recommendation to cite specific previously published works, please review and evaluate these publications to determine whether they are relevant and should be cited. There is no requirement to cite these works unless the editor has indicated otherwise.

Please review your reference list to ensure that it is complete and correct. If you have cited papers that have been retracted, please include the rationale for doing so in the manuscript text, or remove these references and replace them with relevant current references. Any changes to the reference list should be mentioned in the rebuttal letter that accompanies your revised manuscript. If you need to cite a retracted article, indicate the article’s retracted status in the References list and also include a citation and full reference for the retraction notice.

***

We have reviewed our reference list to ensure that it is complete and correct.

We have deleted reference number 50 (added by mistake), 56 and 57 according to the shortening of text suggested by the reviewer.

Reviewers' comments:

Reviewer’s Responses to Questions

Comments to the Author

1. If the authors have adequately addressed your comments raised in a previous round of review and you feel that this manuscript is now acceptable for publication, you may indicate that here to bypass the “Comments to the Author” section, enter your conflict of interest statement in the “Confidential to Editor” section, and submit your "Accept" recommendation.

Reviewer #4: (No Response)

*** No action required.

2. Is the manuscript technically sound, and do the data support the conclusions?

The manuscript must describe a technically sound piece of scientific research with data that supports the conclusions. Experiments must have been conducted rigorously, with appropriate controls, replication, and sample sizes. The conclusions must be drawn appropriately based on the data presented.

Reviewer #4: Yes

*** No action required.

3. Has the statistical analysis been performed appropriately and rigorously?

Reviewer #4: Yes

***No action required.

4. Have the authors made all data underlying the findings in their manuscript fully available?

The PLOS Data policy requires authors to make all data underlying the findings described in their manuscript fully available without restriction, with rare exception (please refer to the Data Availability Statement in the manuscript PDF file). The data should be provided as part of the manuscript or its supporting information, or deposited to a public repository. For example, in addition to summary statistics, the data points behind means, medians and variance measures should be available. If there are restrictions on publicly sharing data—e.g. participant privacy or use of data from a third party—those must be specified.

Reviewer #4: Yes

***No action required.

5. Is the manuscript presented in an intelligible fashion and written in standard English?

PLOS ONE does not copyedit accepted manuscripts, so the language in submitted articles must be clear, correct, and unambiguous. Any typographical or grammatical errors should be corrected at revision, so please note any specific errors here.

Reviewer #4: Yes

***No action required.

6. Review Comments to the Author

Please use the space provided to explain your answers to the questions above. You may also include additional comments for the author, including concerns about dual publication, research ethics, or publication ethics. (Please upload your review as an attachment if it exceeds 20,000 characters)

Reviewer #4: Thank you for addressing my previous comments; the revisions are appreciated. Overall, this is a well-conducted study and the authors should be commended for their work.

***Thank you to review #4 for this encouraging message.

The following suggestions are offered to further strengthen the manuscript:

Discussion: The strengths and limitations sections are currently presented separately with subtitles. I recommend removing these subtitles and integrating the content more concisely into the body of the Discussion, as is conventional in most journals.

***We have considered this suggestion carefully, we also asked a third opinion by an academic who is involved in reviewing papers for the REF (Research Excellence Framework (REF) is the UK’s system for assessing the excellence of research in UK higher education institutions) - most papers do have a separate strengths and limitations, as also seen here

https://doi.org/10.1371/journal.pone.0347997

https://journals.plos.org/plosone/article?id=10.1371/journal.pone.0315486

https://journals.plos.org/plosone/article?id=10.1371/journal.pone.0289245

We have made the decision to keep the section separated for clarity but removing the headings as suggested.

Similarly, the "Comparison with Other Studies" subtitle should be removed, with the comparative content woven naturally into the Discussion narrative.

***We have made this change and we are grateful for the suggestion.

Recommendations/Conclusion: The study recommendations section is overly lengthy as a standalone section. I suggest incorporating the key recommendations into the Discussion where relevant, and consolidating the remainder into a dedicated Conclusion section.

***We have shortened the list of recommendations for the implementation and delivery of new LC clinics, we kept it as separate section for readers to find it quickly when looking for practical tips. We deleted references number 56 and 57.

This Conclusion section should also include a brief paragraph on Recommendations for Future Research, which would provide a more focused and impactful ending to the manuscript.

***We created a separate conclusion section with the addition of the recommendations for future research.

These revisions would improve the overall flow and conciseness of the paper without losing any substantive content.

***Thank you for the suggestions which improved the flow and conciseness of the paper.

-----------------------------------------------

Please address all correspondence concerning this manuscript to me at s.williams35@herts.ac.uk.

Thank you for your consideration of this revised manuscript.

Sincerely,

Dr Stefanie Williams

Attachment Submitted filename: Response_to_reviewers_auresp_4.docx

# Editor report: Decision Letter 4

**DOI:** 10.1371/journal.pone.0313367.r009

**Authors:** Mohammed Abutaleb, Mohammed Abutaleb, Ilse Bloom, Ilse Bloom, Marianne Clemence

19 May 2026

A mixed-methods analysis of the implementation of a new community long-COVID service during the 2020 pandemic: learning from practice.

PONE-D-24-46697R4

Dear Dr. Williams,

We’re pleased to inform you that your manuscript has been judged scientifically suitable for publication and will be formally accepted for publication once it meets all outstanding technical requirements.

Within one week, you’ll receive an e-mail detailing the required amendments. When these have been addressed, you’ll receive a formal acceptance letter and your manuscript will be scheduled for publication.

An invoice will be generated when your article is formally accepted. Please note, if your institution has a publishing partnership with PLOS and your article meets the relevant criteria, all or part of your publication costs will be covered. Please make sure your user information is up-to-date by logging into Editorial Manager at Editorial Manager® and clicking the ‘Update My Information' link at the top of the page. For questions related to billing, please contact billing support.

If your institution or institutions have a press office, please notify them about your upcoming paper to help maximize its impact. If they’ll be preparing press materials, please inform our press team as soon as possible -- no later than 48 hours after receiving the formal acceptance. Your manuscript will remain under strict press embargo until 2 pm Eastern Time on the date of publication. For more information, please contact onepress@plos.org.

Kind regards,

Marianne Clemence

Staff Editor

PLOS One

Additional Editor Comments (optional):

Reviewers' comments:

# Editor report: Acceptance letter

**DOI:** 10.1371/journal.pone.0313367.r010

**Authors:** Mohammed Abutaleb, Mohammed Abutaleb, Ilse Bloom, Ilse Bloom, Marianne Clemence

PONE-D-24-46697R4

PLOS One

Dear Dr. Williams,

I'm pleased to inform you that your manuscript has been deemed suitable for publication in PLOS One. Congratulations! Your manuscript is now being handed over to our production team.

At this stage, our production department will prepare your paper for publication. This includes ensuring the following:

* All references, tables, and figures are properly cited

* All relevant supporting information is included in the manuscript submission,

* There are no issues that prevent the paper from being properly typeset

You will receive further instructions from the production team, including instructions on how to review your proof when it is ready. Please keep in mind that we are working through a large volume of accepted articles, so please give us a few days to review your paper and let you know the next and final steps.

Lastly, if your institution or institutions have a press office, please let them know about your upcoming paper now to help maximize its impact. If they'll be preparing press materials, please inform our press team within the next 48 hours. Your manuscript will remain under strict press embargo until 2 pm Eastern Time on the date of publication. For more information, please contact onepress@plos.org.

You will receive an invoice from PLOS for your publication fee after your manuscript has reached the completed accept phase. If you receive an email requesting payment before acceptance or for any other service, this may be a phishing scheme. Learn how to identify phishing emails and protect your accounts at https://explore.plos.org/phishing.

If we can help with anything else, please email us at customercare@plos.org.

Thank you for submitting your work to PLOS ONE and supporting open access.

Kind regards,

PLOS ONE Editorial Office Staff

on behalf of

Dr Marianne Clemence

Staff Editor

PLOS One
